# Trifluoromethylthiolation of Tryptophan and Tyrosine Derivatives: A Tool for Enhancing the Local Hydrophobicity of Peptides

**DOI:** 10.1021/acs.joc.3c01373

**Published:** 2023-09-06

**Authors:** Jure Gregorc, Nathalie Lensen, Grégory Chaume, Jernej Iskra, Thierry Brigaud

**Affiliations:** † Chair of Organic Chemistry, Faculty of Chemistry and Chemical Technology, University of Ljubljana, Večna pot 113, Ljubljana 1000, Slovenia; ‡ CY Cergy Paris Université, CNRS, BioCIS, Cergy Pontoise 95000, France; § Université Paris-Saclay, CNRS, BioCIS, Orsay 91400, France

## Abstract

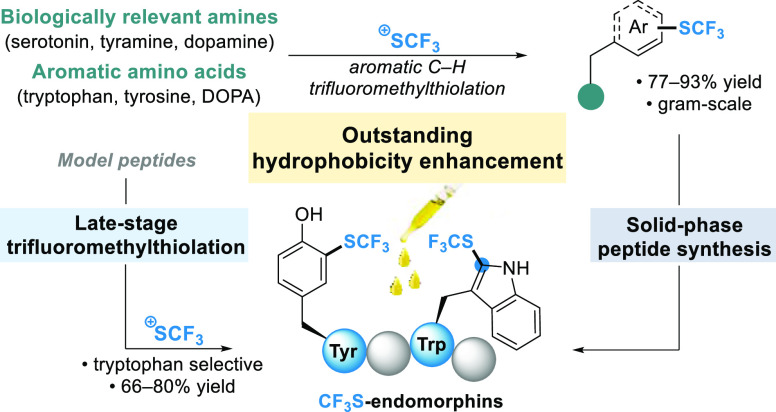

The incorporation of fluorinated groups into peptides significantly affects their biophysical properties. We report herein the synthesis of Fmoc-protected trifluoromethylthiolated tyrosine (CF_3_S-Tyr) and tryptophan (CF_3_S-Trp) analogues on a gram scale (77–93% yield) and demonstrate their use as highly hydrophobic fluorinated building blocks for peptide chemistry. The developed methodology was successfully applied to the late-stage regioselective trifluoromethylthiolation of Trp residues in short peptides (66–80% yield) and the synthesis of various CF_3_S-analogues of biologically active monoamines. To prove the concept, Fmoc-(CF_3_S)Tyr and -Trp were incorporated into the endomorphin-1 chain (**EM-1**) and into model tripeptides by solid-phase peptide synthesis. A remarkable enhancement of the local hydrophobicity of the trifluoromethylthiolated peptides was quantified by the chromatographic hydrophobicity index determination method, demonstrating the high potential of CF_3_S-containing amino acids for the rational design of bioactive peptides.

## Introduction

The introduction of fluorine atoms into biomolecules has become a well-established strategy in drug development, as they can be used to favorably improve or modulate their physicochemical and biological properties.^1^ This approach is now widely exploited in the pharmaceutical industry, with 20–25% of marketed drugs containing at least one fluorine atom.^2^ Concurrently, peptides have emerged as a unique class of therapeutic agents in recent years. Over the past decade, their development has steadily increased, and therapeutic peptides now account for a significant portion of the pharmaceutical market.^3^ Moreover, tailor-made amino acids are becoming privileged scaffolds even in small-molecule drugs (which account for over 30% of pharmaceuticals).^4^ These paradigm shifts in medicinal chemistry call for further efforts to advance the synthesis of diverse fluorinated amino acids (F-AAs) for expanding the peptide design toolbox.^5^

In particular, the incorporation of F-AAs into peptides is a powerful tool for modulating various parameters, such as local hydrophobicity,^6^ pK_a_ values of proximal functionalities,^1b^ membrane permeability,^7^ metabolic stability,^8^ and inducing conformational constraints and self-assembly properties.^9^ Furthermore, fluorine atoms are used as highly sensitive probes for ^19^F NMR spectroscopy in biological media.^9b,10^ The most commonly investigated F-AAs contain either a fluorine atom or the CF_3_ group.^11^ However, other fluorinated groups, especially motifs bearing chalcogen atoms, remain underexplored in the context of amino acid chemistry.^12^ Among them, the trifluoromethylthio group (CF_3_S) is of particular interest as it has one of the highest lipophilicity parameters (Hansch-Leo parameter; π = 1.44),^13^ a strong electron-withdrawing effect (Hammett constant σ_m_ = 0.40, σ_p_ = 0.50),^14^ and a favorable pharmacological profile.^15^ Therefore, the synthesis of trifluoromethylsulfanylated amino acids (CF_3_S-AAs) and their incorporation into peptides appears to be a promising strategy to improve their physicochemical properties. In particular, the local hydrophobicity and membrane permeability could be significantly increased, improving the drug profile of the peptides.^15,16^

**Scheme 1:**
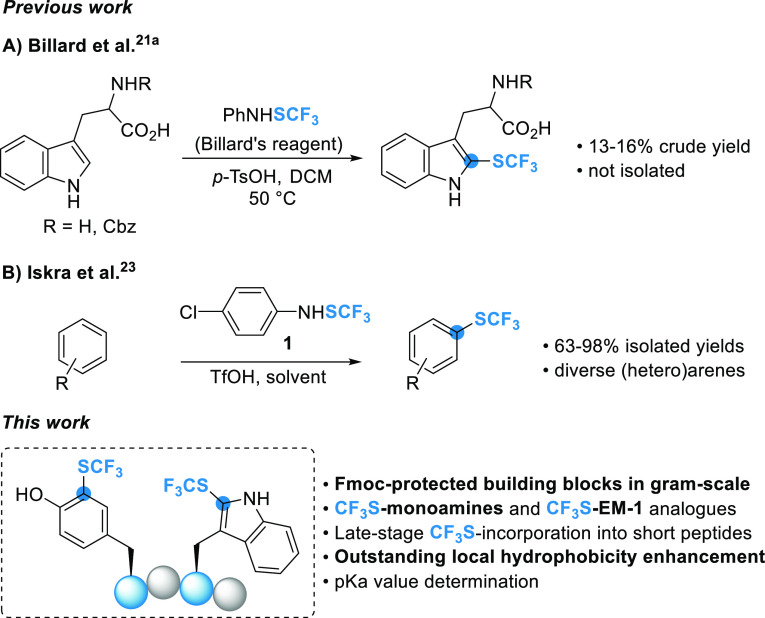
Previous Reports on Direct Aromatic Trifluoromethylthiolation of Trp Derivatives or (Hetero)arenes Using the Trifluoromethanesulfenamide Reagents and Our Work Reported Herein

Most of the CF_3_S-AAs reported so far involve the aliphatic AAs, in particular, the trifluoromethylcysteine (TfmCys)^16^ and trifluoromethionine (TFM) analogues.^17^ They proved to be useful as sensitive ^19^F NMR reporters to probe protein–protein interactions (PPIs),^10a^ as well as for their ability to enhance the local hydrophobicity of model peptides.^6b^ A CF_3_S-cysteine (CF_3_S-Cys) analogue was also prepared by direct trifluoromethylthiolation of the corresponding thiol.^18^ Although there are numerous developed methods^15a,16,19^ and a plethora of shelf-stable and efficient reagents^20^ for aromatic trifluoromethylthiolation, the synthesis of aromatic CF_3_S-containing amino acids (CF_3_S-AAs) is still in its infancy and has been very scarcely reported to date. A few years ago, Billard and co-workers demonstrated the reactivity of various substituted indoles toward electrophilic trifluoromethylthiolation using their first- and second-generation trifluoromethanesulfenamide reagents.^21^ While the reaction was effective in introducing the CF_3_S group at the C2-position of unprotected indole propionic acid and tryptamine, very limited conversion (*ca.* 15%) was observed with Cbz-protected and unprotected Trp residues, and the desired products were not isolated (Scheme 1A).^21a^ Nevertheless, the reaction conditions proved to be sensitive to the nature of the aromatic ring and are mainly limited to electron-rich aromatic compounds. Very recently, the synthesis of *p*-SCF_3_ phenylalanine (Phe) through a photoredox-mediated Ni-catalyzed trifluoromethylthiolation pathway was reported.^22^ To the best of our knowledge, CF_3_S incorporation into tyrosine derivatives has not been reported so far.

As the demand for original fluorinated compounds continues to increase, the development of robust methods that allow efficient access to aromatic CF_3_S-AAs on a gram scale is of great importance. We have previously demonstrated that the *p*-chloro analogue **1** of the first-generation Billard’s^21^ reagent is more stable and promotes efficient electrophilic Friedel-Crafts trifluoromethylthiolation of diverse (hetero)arenes (Scheme 1B).^23^ Herein, we have investigated the Friedel-Crafts trifluoromethylthiolation with reagent **1** for the preparation of a series of aromatic CF_3_S-AAs (Scheme 1). We first report the synthesis of trifluoromethylthiolated tryptophan (CF_3_S-Trp) and tyrosine (CF_3_S-Tyr) amino acids, as well as their related monoamine analogues, such as trace amines and catecholamines. As an extension, the feasibility of the late-stage functionalization (LSF) was investigated on a series of Trp- and/or Tyr-containing short peptides. In addition, solid-phase peptide synthesis (SPPS) synthesis of fluorinated analogues of the opioid agonist endomorphin-1 (**EM-1**) was performed using the ready-to-use Fmoc-protected CF_3_S-Trp and CF_3_S-Tyr building blocks. Finally, we report a significant enhancement of the local hydrophobicity and acidity originating from the CF_3_S group.

## Results and Discussion

The electrophilic trifluoromethylthiolation reaction was first studied on tryptophan derivatives **2a–c** (Table 1).

**Table 1:**
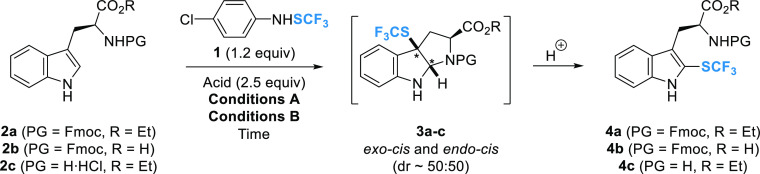
Trifluoromethylthiolation of Tryptophan Derivatives **2a–c**

entry	substrate	acid	conditions	3/4 ratio^a^	yield (%)^b^
1	**2a**	TsOH	**A**, 24 h	only **3a**	29
2^c^	**2a**	TfOH	**A**, 24 h	14:1	71
3	**2a**	TfOH	**A**, 24 h	2:1	92
4	**2a**	TfOH	**B**, 6 h	only **4a**	90
5	**2a**	BF_3_·OEt_2_	**A**, 24 h	2.1:1	44
6	**2a**	BF_3_·OEt_2_	**B**, 24 h	1:2.2	58
7	**2a**	BF_3_·OEt_2_	**B**, 48 h	1:2.7	73
8^d^	**2a**	BF_3_·OEt_2_	**B**, 48 h	only **4a**	78
9	**2b**	BF_3_·OEt_2_	**B**, 24 h	only **4b**	84
10^e^	**2b**	BF_3_·OEt_2_	**B**, 24 h	only **4b**	93
11	**2c**	TfOH	**A**, 30 min	only **4c**	96

a PG-Trp-OR **2a–c** (0.10 mmol, 1.0 equiv), ArNHSCF_3_**1** (1.2 equiv), acid (2.5 equiv), conditions **A** (DCM; 1 mL, 0.1 M; rt) or conditions **B** (DCE; 1 mL, 0.1 M; 50 °C), 0.5–48 h. **3***vs***4** ratios determined by ^19^F NMR analysis of the crude reaction mixture.

b Isolated yield after purification of **3** and **4** or the inseparable mixture thereof.

c TfOH (1.0 equiv).

d BF_3_·OEt_2_ (2 × 2.5 equiv, second addition after 24 h).

e Reaction performed on a gram scale (4.0 mmol).

The Fmoc-protecting group was selected to provide direct access to readily available building blocks for SPPS. In addition, the Fmoc group is known to tolerate the general acidic conditions used in our previous report.^23^ Trifluoromethylthiolation was first attempted on Fmoc-Trp-OEt **2a** by applying the conditions of Billard *et al.*(21a) with TsOH (2.5 equiv) as an activator in DCM at rt (conditions **A**) (Table 1, entry 1). Instead of the expected product **4a**, the formation of CF_3_S-hexahydropyrrolo[2,3-*b*]indole **3a** was observed in a low yield (29%) as a separable *ca.* 1:1 mixture of *endo*-*cis* and *exo*-*cis* diastereomers (see the Supporting Information for structural elucidation).^24^ The formation of the intermediate compound **3a** is consistent with the previously reported cascade trifluoromethylthiolation-cyclization of N-protected tryptamines.^25^ Similar to Qing *et al.*,^25a^ we observed in the AA-series that **3a** undergoes ring opening after a prolonged time in acidic media, yielding the expected CF_3_S-Trp **4a**. To increase the activation of the electrophile and the rate of the ring-opening process, we decided to replace TsOH with a stronger acid, namely, triflic acid (TfOH). The use of only 1.0 equiv of TfOH resulted in an inseparable 14:1 mixture of **3a** and **4a** in a 71% yield (Table 1, entry 2). Addition of TfOH in excess (2.5 equiv) increased both the selectivity for product **4a** (**3a/4a** = *ca.* 2:1) and the isolated yield of **3a** and **4a** (92%) (Table 1, entry 3). Increasing the reaction temperature to 50 °C in DCE (conditions **B**) allowed the exclusive formation of **4a** in a 90% isolated yield in a shorter reaction time (Table 1, entry 4). The reaction also proved effective under Lewis acid activation. An initial experiment with BF_3_·OEt_2_ under conditions **A** gave a mixture of **3a** and **4a** in a ratio of 2.1:1 (Table 1, entry 5). Application of conditions **B** resulted in an opposite 1:2.2 ratio of **3a**/**4a** (Table 1, entry 6). Increasing the reaction time to 48 h improved the yield but had a limited effect on the ring-opening step (Table 1, entry 7). Finally, a second addition of 2.5 equiv of BF_3_·OEt_2_ after 24 h proved effective for complete conversion to **4a** (Table 1, entry 8). It is noteworthy that trifluoromethylthiolation under Lewis acid activation gave a cleaner conversion compared to TfOH activation. To gain direct access to ready-to-use building blocks for SPPS, we decided to investigate the trifluoromethylthiolation of Fmoc-Trp-OH **2b**. Compared to the ester series (Table 1, entry 7), the reaction on Fmoc-protected acid **2b** using 2.5 equiv of BF_3_·OEt_2_ under conditions **B** resulted in a complete conversion to the expected CF_3_S-Trp **4b** after 24 h (Table 1, entry 9). With the optimized reaction conditions in hand, the trifluoromethylthiolation of **2b** was carried out on a gram scale, yielding approximately 2 g of the desired product **4b** (93% yield, Table 1, entry 10). The enantiopurity of **4b** was analyzed by chiral HPLC, which showed no epimerization at the C_α_ position (see the Supporting Information, Chapter 5). Next, we investigated the effect of the N-protection on the reaction outcome. Trifluoromethylthiolation starting from N-unprotected ethyl ester **2c** under TfOH activation proceeded much faster (30 min) compared to **2a** (Table 1, entry 3), and the corresponding CF_3_S-Trp **4c** was isolated in a 96% yield (Table 1, entry 11). This result highlights the key role of the nature of the amino group in the ring-opening step.

**Scheme 2:**
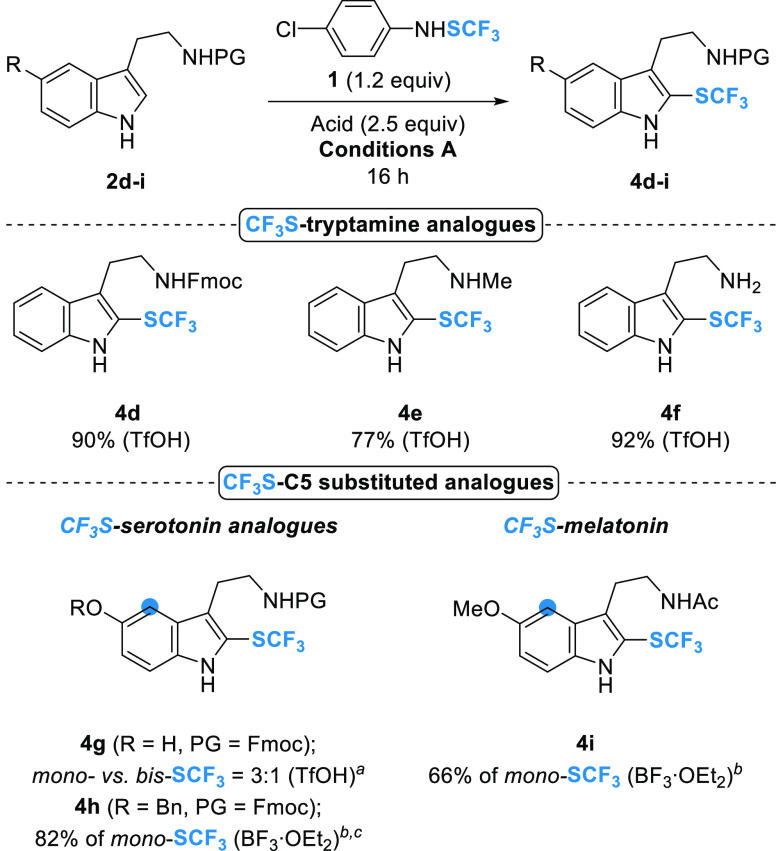
Synthesis of CF_3_S-Tryptamine Derivatives **4d–i** (Isolated Yields) **2d–i** (0.1 mmol, 1.0 equiv), ArNHSCF_3_**1** (1.2 equiv), acid (2.5 equiv; in parentheses), conditions **A** (DCM; 1 mL, 0.1 M; rt), 16 h. ArNHSCF_3_**1** (1.05 equiv); ratio determined by ^19^F NMR of the crude product. ^b^ArNHSCF_3_**1** (1.05 equiv), BF_3_·OEt_2_ (5.0 equiv), 24 h. ^c^conditions **B** (DCE; 1 mL, 0.1 M; 50 °C).

We then decided to extend the scope of the reaction to biologically important tryptamine-based trace amines (Scheme 2).

Several CF_3_S-tryptamine derivatives have already been described in the literature using the trifluoromethanesulfenamide reagent in the presence of TsOH.^25a^ N-substituted tryptamines favored the formation of the CF_3_S-pyrroloindoline intermediates,^25a^ while in the case of the unprotected tryptamine **2f**, only the corresponding CF_3_S-tryptamine **4f** has been isolated in very low yield.^21a^ Herein, the use of reagent **1** in the presence of TfOH allowed us to selectively obtain Fmoc-protected and *N*-methyl tryptamine analogues **4d** and **4e** (90 and 77% isolated yields, respectively), and to significantly improve the isolated yield (92%) of the unprotected CF_3_S-tryptamine **4f** (Scheme 2). CF_3_S introduction was then attempted on serotonin and melatonin, two important biologically relevant representatives of the C5-functionalized tryptamine derivatives. TfOH activation of Fmoc- protected serotonin **2g** resulted in a *ca.* 3:1 mixture of *mono*-SCF_3_ (at the C2-indole position) and *bis*-SCF_3_ (at the C2- and C4-indole positions) products. We assumed that the formation of the *bis*-SCF_3_ compound arises due to the presence of the hydroxyl group at the C5-position, which causes a considerable increase in the electron density of the indole. Therefore, we decided to decrease the activation of the indole by protecting the free hydroxyl with a benzyl group (see Table S1 for optimization data). In addition, the BF_3_·OEt_2_ activation was preferred to avoid the possible deprotection of the benzyl group by TfOH. Under these conditions, only the *mono*-SCF_3_ serotonin derivative **4h** was obtained in high yield (Scheme 2). When applied to melatonin **2i**, which has a methoxy group at the C5-indole position, these conditions gave the expected *mono*-SCF_3_ melatonin **4i** in a 66% yield (Scheme 2).

We further explored the scope and limitations of our method with less electron-rich aromatic tyrosine (Tyr) derivatives (Table 2).

**Table 2:**
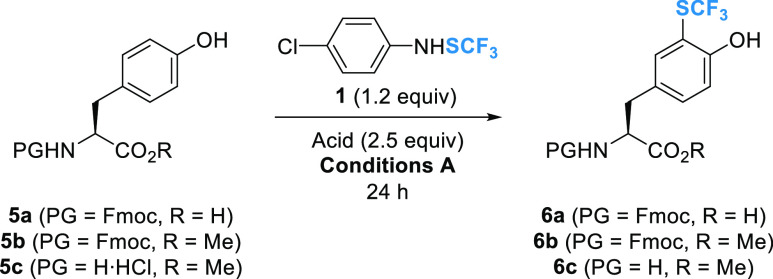
Trifluoromethylthiolation of Tyrosine Derivatives **5a–c**

entry	substrate	acid	conversion (%)^a^	yield (%)^b^
1	**5a**	BF_3_·OEt_2_	7 (25)^c^	n.d.
2	**5a**	TfOH	93	n.d.
3^d^	**5a**	TfOH	>99	**6a** (79)
4^d^^,^^e^	**5a**	TfOH	>99	**6a** (77)
5^f^	**5b**	TfOH	>99	**6b** (93)
6	**5c**	TfOH	<1	n.d.

a PG-Tyr-OR **5a–c** (0.10 mmol, 1.0 equiv), ArNHSCF_3_**1** (1.2 equiv), acid (2.5 equiv), conditions **A** (DCM; 1 mL, 0.1 M; rt). Conversion to **6a** determined by ^1^H NMR analysis (DMSO-*d*_6_) of the crude reaction mixture.

b n.d.: not determined.

c Conditions **B** (DCE; 1 mL, 0.1 M; 50 °C), 24 h.

d 1.5 equiv of ArNHSCF_3_**1**.

e Reaction performed on a gram scale (4.0 mmol).

f ArNHSCF_3_**1** (2.5 equiv), TfOH (2 × 2.5 equiv, second addition after 18 h).

To access readily available building blocks for SPPS, Fmoc-Tyr-OH **5a** was first subjected to the previously optimized conditions for Trp. The trifluoromethylthiolation of **5a** using BF_3_·OEt_2_ at rt (conditions **A**) and at 50 °C (conditions **B**) proceeded with a very low conversion (Table 2, entry 1). These results were attributed to the lower reactivity of the phenolic moiety compared to the indole. Replacing BF_3_·OEt_2_ with TfOH significantly improved the reaction conversion (Table 2, entry 2). Moreover, increasing the amount of reagent **1** to 1.5 equiv resulted in complete conversion, and Fmoc-protected CF_3_S-Tyr **6a** was isolated in a 79% yield (Table 2, entry 3). A gram-scale trifluoromethylthiolation of **5a** yielded *ca.* 1.6 g of the enantiomerically pure Fmoc-protected building block **6a** in 77% isolated yield (Table 2, entry 4). It is worthwhile to note that the phenol group of this amino acid should be orthogonally protected by a *t*-butyl group for incorporation into longer peptide sequences. In the case of the Fmoc-Tyr-OMe substrate **5b**, harsher conditions (2.5 equiv of **1**, 2 × 2.5 equiv of TfOH) were required to achieve quantitative CF_3_S-incorporation and the ester analogue **6b** was isolated in an excellent yield of 93% (Table 2, entry 5). In contrast to the observation in the Trp series, the trifluoromethylthiolation of **5c** without Fmoc protection of the N-terminus did not proceed (Table 2, entry 6).

**Scheme 3:**
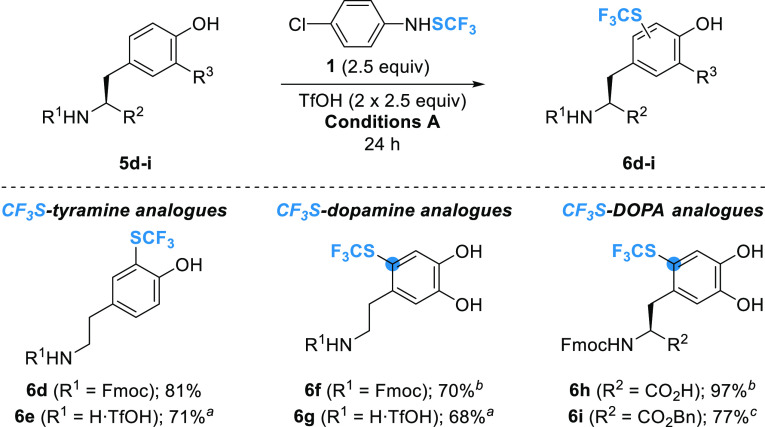
Synthesis of CF_3_S-Tyramine, -Dopamine, and -DOPA Derivatives **6d–i** Substrate **5d–i** (0.10 mmol, 1.0 equiv), ArNHSCF_3_**1** (0.25 mmol, 2.5 equiv), TfOH (2 × 2.5 equiv, second addition after 18 h), conditions **A** (DCM, 0.1 M, rt), 24 h; isolated yields in parentheses. 0.20 mmol scale (1.0 equiv). ^b^ArNHSCF_3_**1** (1.1 equiv), TfOH (2.5 equiv), overnight. ^c^ArNHSCF_3_**1** (1.2 equiv), BF_3_·OEt_2_ (5.0 equiv), conditions **B** (DCE; 1 mL, 0.1 M; 50 °C), 24 h.

Encouraged by the novel reactivity of tyrosine-based substrates, we investigated the CF_3_S-functionalization of tyramine, dopamine, and DOPA derivatives **5d–i** (Scheme 3).

As with the Tyr series (see Table 2, entry 5), harsher conditions had to be applied to obtain Fmoc-protected and unprotected CF_3_S-tyramines **6d** and **6e** in good yields (Scheme 3). In the case of the dopamine and DOPA analogues, standard conditions (1.2 equiv of **1**, 2.5 equiv of TfOH) are sufficient to achieve quantitative CF_3_S incorporation in the electron-rich catechol motif. However, a change in regioselectivity was observed because of the *para*-directing effect of the additional hydroxyl group (Scheme 3). This allowed straightforward synthesis of Fmoc-protected and unprotected CF_3_S-dopamines **6f** and **6g** as single regioisomers in 70 and 68% yields, respectively (Scheme 3). When applied to Fmoc-protected DOPA **5h**, the reaction gave the corresponding 2–SCF_3_ derivative **6h**. Purification of **6h** by flash chromatography showed that the product was not stable on silica after a prolonged time. Nevertheless, **6h** could be isolated in satisfactory purity only by acidic workup. On the other hand, quantitative conversion of Fmoc-DOPA-OBn **5i** could be achieved under BF_3_·OEt_2_ activation (5.0 equiv, conditions **B**) to afford the corresponding **6i** in a 77% yield (Scheme 3). The scope of the method was then tentatively extended to phenylalanine substrates (Phe), but no reaction occurred. The reactivity of the phenyl ring of Phe is low, and trifluoromethylthiolation occurs on the aniline ring of reagent **1**. Such limitations were also demonstrated in our previous report on the trifluoromethylthiolation of arenes.^23^

**Scheme 4:**
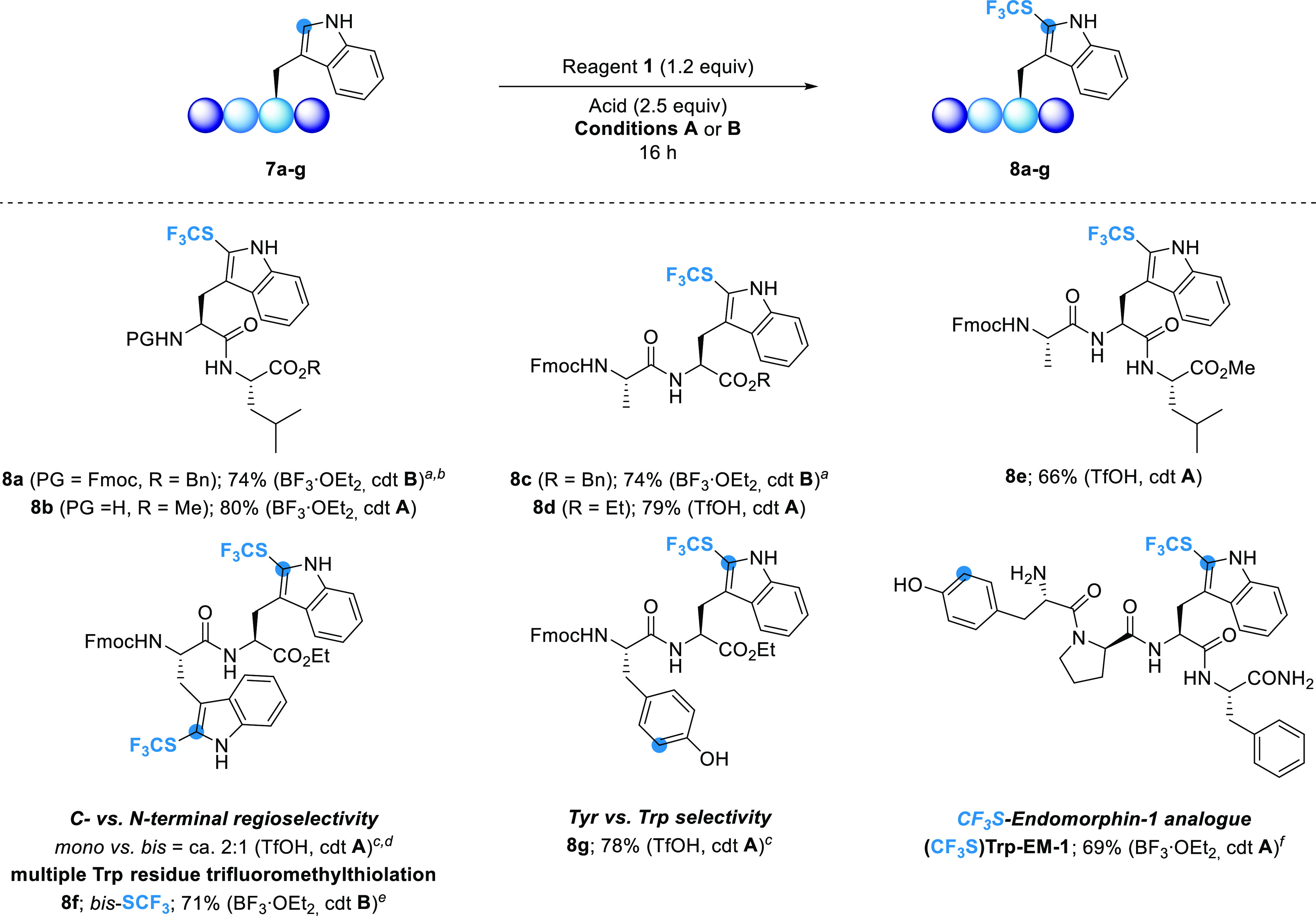
Late-Stage Trifluoromethylthiolation of Trp Residue-Containing Peptides **7a**–**g** Peptide **7a–g** (0.10 mmol, 1.0 equiv), ArNHSCF_3_**1** (1.2 equiv). acid (2.5 equiv), conditions **A** (DCM; 1 mL, 0.1 M; rt) or conditions **B** (DCE; 1 mL, 0.1 M; 50 °C), overnight. BF_3_·OEt_2_ (2 × 2.5 equiv). ^b^48 h. ^c^ArNHSCF_3_**1** (1.05 equiv). ^d^Ratios estimated by ^19^F NMR analysis. ^e^ArNHSCF_3_**1** (2.2 equiv), BF_3_·OEt_2_ (2 × 5.0 equiv), 48 h. ^f^**TFA·EM-1** (10 mg scale, 0.014 mmol, 1.0 equiv), BF_3_·OEt_2_ (20 equiv), DCM (0.05 M), rt, 24 h; Conversion determined by UPLC-MS analysis.

LSF is a powerful diversification tool for the synthesis of peptides featuring unnatural AAs with modified properties or biological activity. Because of its high reactivity, the Trp residue is often targeted for such modifications.^26^ We therefore investigated the feasibility of the method for LSF on a series of Trp- and/or Tyr-containing short peptides (Scheme 4).

Late-stage trifluoromethylthiolation was first investigated on a series of peptides with a Trp residue at the N- or C-terminal position (**7a–b** and **7c–d**) and in the middle position of the peptide chain (**7e**). LSF of Fmoc-protected peptides was carried out using BF_3_·OEt_2_ activation under the optimized conditions found for the Fmoc-Trp ester **2a** (see Table 1, entry 8). CF_3_S-peptide **8a** with the Fmoc-Trp residue at the N-terminal position was obtained in a 74% yield after 48 h (Scheme 4). LSF of the peptide **7b**, with the free N-terminal amino group, was first performed under TfOH activation, which was the condition successfully used for the unprotected Trp ester **2c** (see Table 1, Entry 11). However, the reaction resulted in a complex reaction mixture. Upon BF_3_·OEt_2_ activation at room temperature, a clean conversion was observed, and peptide **8b** was obtained in an 80% yield. Trifluoromethylthiolation of peptide **7c** with the Trp residue at the *C*-terminal position gave the corresponding dipeptide **8c** in a 74% yield after 16 h. It appears that the reaction proceeds much faster when the Trp residue is at the *C*-terminal position. LSF of Fmoc-protected peptides also worked efficiently under TfOH activation. Peptides **8d** and **8e**, having the Trp residue at the *C*-terminus and in the middle position of the peptide chain, were obtained in good yields (Scheme 4). Because of the difference in kinetics observed with peptides **8a** and **8c**, we decided to investigate the regioselectivity of the trifluoromethylthiolation on the Trp–Trp dipeptide **7f**. CF_3_S incorporation under TfOH activation gave a mixture of *mono*- and *bis*-SCF_3_ dipeptide **8f** in a ratio of *ca.* 2:1 (Scheme 4). Therefore, we have chosen the reaction conditions to selectively obtain the *bis*-SCF_3_ dipeptide **8f**. Under TfOH activation, the reaction gave a complex mixture, whereas, under BF_3_·OEt_2_ activation, the dipeptide **8f** was isolated in a 71% yield (Scheme 4). The chemoselectivity aspect of the late-stage SCF_3_ incorporation, based on the difference in reactivity between Trp and Tyr residues, has also been investigated. Complete chemoselectivity in favor of the Trp residue was observed for the model dipeptide **7g**. The reaction resulted in a quantitative conversion when 1.05 equiv of **1** was used and peptide **8g** was obtained in a 78% yield (Scheme 4). A model Ala-Tyr dipeptide **7h** was also tested for selective LSF of the Tyr residue. Unfortunately, the required harsher conditions (as in Table 2, entry 5) were not tolerated by the peptide substrate, resulting in a complex reaction mixture. To highlight the generalization of LS trifluoromethylthiolation to longer peptides of interest, we investigated the reaction on the analgesic peptide endomorphin-1 (**EM-1**). This substrate was selected as a relevant biologically active peptide target because it contains both Tyr and Trp residues. As expected, by applying the LSF conditions using a large excess of BF_3_·OEt_2_ (20 equiv), only the formation of **(CF**_**3**_**S)Trp-EM-1** was observed in 69% conversion, as determined by UPLC-MS and NMR analysis (Scheme 4).

As a complementary strategy to LSF, we decided to investigate the synthesis of the **(CF**_**3**_**S)Trp-EM-1** analogue by SPPS. The **(CF**_**3**_**S)Trp-EM-1** was obtained in a 26% yield using standard protocols and coupling reagents (HATU/DIPEA) (Table 3).

**Table 3:**
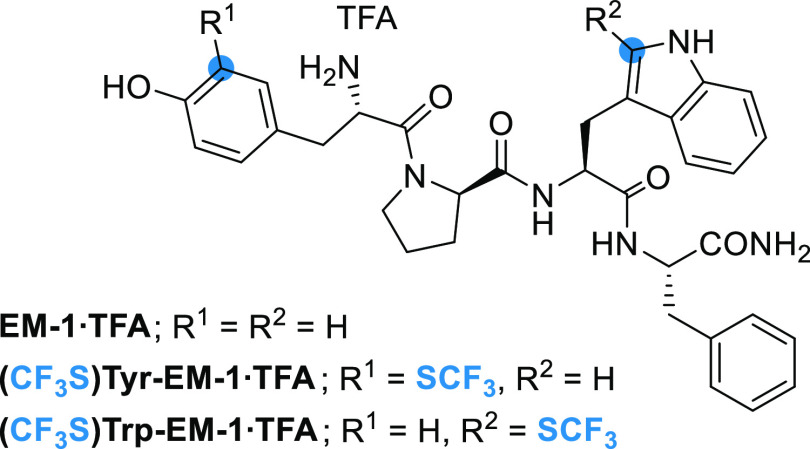
SPPS of **EM-1** Analogues

peptide	yield (%)	*trans vs cis*^a^	t*R* (min)^b^
**EM-1**	24	66:34	8.1
**(CF**_**3**_**S)Trp-EM-1**	26	71:29	10.9
**(CF**_**3**_**S)Tyr-EM-1**	19	71:29	11.6

a Conformer ratio of the peptidyl-prolyl amide bond determined by ^1^H and/or ^19^F NMR spectroscopy in MeOD-*d*_3_ (see the Supporting Information, Chapter 4.2.).

b RP-HPLC analysis (20% → 60% MeCN + 0.1% TFA in H_2_O + 0.1% TFA).

**Figure 1:**
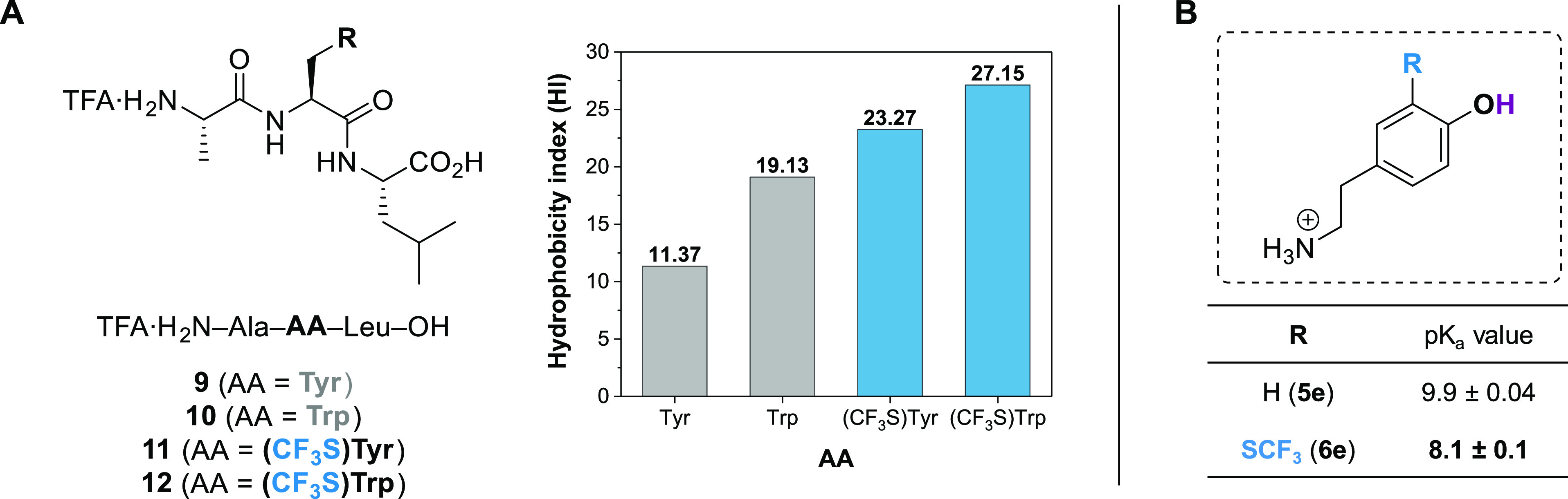
Biophysical property evaluation. (A) HI for non-fluorinated tripeptides **9**–**10** and the CF_3_S-tripeptides **11**–**12**; (B) phenolic moiety pK_a_ values of tyramine **5e** and CF_3_S-tyramine **6e** triflate salts in H_2_O/D_2_O = 90:10 by ^1^H NMR spectroscopy.

Compared to endomorphin-1 (**EM-1**), the two fluorinated analogues were obtained in comparable yields and have similar *cis*/*trans* ratios of the peptidyl-prolyl amide bond. These results indicate that Fmoc-(CF_3_S)Trp-OH **4b** and Fmoc-(CF_3_S)Tyr-OH **6a** are suitable building blocks for SPPS and that their incorporation into **EM-1** does not significantly affect the peptide conformation (Table 3). Interestingly, the retention times (t*R*) determined by reverse-phase HPLC were significantly longer for both CF_3_S-containing peptides compared to the parent **EM-1** (*ca.* 3 min). This result supports the expected enhancement of local hydrophobicity. To achieve an accurate determination of the local hydrophobicity enhancement imparted by the CF_3_S group in a peptide context, we decided to measure the chromatographic hydrophobicity indexes of a series of four H_2_N-Ala-AA-Leu-OH tripeptides containing the respective Tyr, Trp, and (CF_3_S)Tyr or the (CF_3_S)Trp residue at the AA position (Figure 1).

The hydrophobicity index (HI) shift ΔHI (HI_(CF3S)AA_ – HI_AA_) is an assessment of the contribution of the CF_3_S substituent to the increase in local hydrophobicity. This method, developed by Valkó *et al.*,^27^ has already been successfully applied to measure the increased local hydrophobicity of peptides containing fluorinated AAs.^6b,28^ This short peptide sequence was chosen to prevent the formation of secondary structures that could affect hydrophobicity (see the Supporting Information, Chapter 6, for details). As shown in Figure 1, CF_3_S-containing peptides **11** and **12** have significantly higher HI compared to their non-fluorinated counterparts **9** and **10**. These results corroborate the significant increase in RP-HPLC retention times measured with fluorinated **EM-1** analogues. As observed in the non-fluorinated series (peptides **9** and **10**), the (CF_3_S)Trp-containing peptide **12** exhibits a higher HI than the (CF_3_S)Tyr peptide **11**. It is noteworthy that the replacement of the Tyr residue with its CF_3_S-Tyr analogue provides a higher local enhancement of the hydrophobicity (ΔHI = HI_(CF3S)Tyr_ – HI_Tyr_*ca.* 12 units) compared to the Trp series (ΔHI = HI_(CF3S)Trp_ – HI_Trp_*ca.* 8 units). These results showed that (CF_3_S)Tyr and (CF_3_S)Trp are the most hydrophobic AAs determined so far by this method.^6b^

Finally, we assessed the influence of the CF_3_S group on the acidity of the vicinal phenol function in a simplified tyramine model carrying an *ortho* CF_3_S. This functionality is highly important for peptide/protein active site interactions. The pK_a_ values of the tyramine **5e** and the CF_3_S-tyramine **6e** triflate salts were determined by ^1^H NMR spectroscopy in H_2_O/D_2_O 90:10 (see the Supporting Information, Chapter 7, for more details).^29^ It is noteworthy that in the case of CF_3_S-tyramine **6e**, the pK_a_ value could also be determined by ^19^F NMR spectroscopy. As expected, the CF_3_S has a major impact on the pK_a_ value with a 100-fold increase of the acidity of the vicinal hydroxyl group (pK_a,(CF3S)Tyr_ = 8.1, pK_a,Tyr_ = 9.9, Figure 1). The values of the pK_a_ of CF_3_S- and CF_3_-phenols are comparable (8.25^30^ and 8.42^31^ for 2-CF_3_ phenols in H_2_O), which is consistent with their similar electron-withdrawing nature (Hammet substituent constant: CF_3_: σ_m_ = 0.43, σ_p_ = 0.54; CF_3_S: σ_m_ = 0.40, σ_p_ = 0.50).^14^

## Conclusions

In this work, we have developed an efficient method for the aromatic trifluoromethylthiolation of tryptophan and tyrosine residues. Reactions were carried out using the electrophilic trifluoromethanesulfenamide reagent **1** in the presence of TfOH or BF_3_·OEt_2_ as activating acids. Several trifluoromethylthiolated tryptophan analogues (CF_3_S-Trp) and their biologically active monoamine derivatives, protected or unprotected, were prepared in high yields following a thorough methodological study. We also demonstrated the scope of our method by preparing the Fmoc-(CF_3_S)Trp-OH **4b** and Fmoc-(CF_3_S)Tyr-OH **6a** building blocks that are ready-to-use for SPPS on a gram scale. The late-stage CF_3_S-functionalization of short peptides showed total regioselectivity in favor of the Trp residue over other aromatic side chains and was successfully applied to the opioid agonist endomorphin-1 (**EM-1**). The synthesis of two trifluoromethylthiolated analogues of **EM-1** was achieved by SPPS using standard protocols and Fmoc-protected building blocks **4b** and **6a**. Finally, we investigated the effect of the incorporation of the CF_3_S group into aromatic Trp and Tyr residues on the physicochemical properties of the peptide framework. We demonstrated that the CF_3_S-containing peptides exhibited a significant enhancement of the local hydrophobicity compared with their non-fluorinated counterparts. In this regard, the CF_3_S-Trp is more hydrophobic than the CF_3_S-Tyr. Moreover, the incorporation of a CF_3_S group adjacent to the phenol ring of tyramine significantly increases its acidity. Because of the key role played by Trp and Tyr residues in protein–protein interactions and the great biological importance of Trp- and Tyr-derived monoamines, the reported CF_3_S-containing analogues represent a new class of compounds endowed with unique properties.^32^ Their ability to dramatically increase local hydrophobicity and modulate the pK_a_ of adjacent functional groups makes them very attractive for medicinal chemistry applications. The ease of implementation of the reported trifluoromethylthiolation should inspire further developments in the design of bioactive peptides.

## Data Availability

The data underlying this study are available in the published article and its online Supporting Information.

## Supporting Information Available

Experimental procedures, characterization data, copies of NMR spectra for all new compounds, HPLC traces of endomorphin-1 analogues, HPLC enantiopurity analysis of compounds **4b** and **6a**, HI determinations, and pKa determinations of compounds **5e** and **6e** (PDF)
