# Supplementary material for: Trifluoromethylthiolation of Tryptophan and Tyrosine Derivatives: A Tool for Enhancing the Local Hydrophobicity of Peptides
Source: J Org Chem. 2023 Sep 6;88(18):13169–77. doi: 10.1021/acs.joc.3c01373 (PMC10507666; doi:10.1021/acs.joc.3c01373)
Supplement: Supplementary file 1 — jo3c01373_si_001.pdf [file jo3c01373_si_001.pdf]

Trifluoromethylthiolation of Tryptophan and Tyrosine Derivatives: A Tool for Enhancing the Local Hydrophobicity of Peptides

Jure Gregorc,[a,b] Nathalie Lensen,[b] Grégory Chaume,*[,b] Jernej Iskra,*[,a] Thierry Brigaud*[,b]

[a]University of Ljubljana, Faculty of Chemistry and Chemical Technology, Večna pot 113, 1000 Ljubljana, Slovenia

[b]CY Cergy Paris Université, CNRS, BioCIS, 95000 Cergy Pontoise, France

Université Paris-Saclay, CNRS, BioCIS, 91400 Orsay, France

email:

gregory.chaume@cyu.fr

jernej.iskra@fkkt.uni-lj.si

thierry.brigaud@cyu.fr

Supporting information

Table of Contents

1. General information S3
2. Preparation of starting materials S5
3. Trifluoromethylthiolation reactions S18
   3.1. General trifluoromethylthiolation procedure S18
   3.2. Synthesis and structural elucidation of (CF_3S)-hexahydropyrrolo[2,3-*b*]indole **3a** S18
   3.3. Trifluoromethylthiolation of tryptophan and derivatives **2a-f** S23
   3.4. Trifluoromethylthiolation of C5-substituted tryptamines **2g-i** S28
   3.5. Trifluoromethylthiolation of tyrosine derivatives **5a-i** S31
   3.6. Late-stage trifluoromethylthiolation of Trp and Tyr containing di- and tripeptides **7a-g** S38
4. Synthesis of trifluoromethylthiolated endomorphin-1 analogues S44
   4.1. Late-stage trifluoromethylthiolation of endomorphin-1 .... S44
   4.2. SPPS of endomorphin-1 analogues S48
5. Enantiopurity analysis S54
6. Hydrophobicity index determination S57
7. pK_a determination S66
8. Copies of NMR spectra S73
   8.1. NMR spectra of starting materials S73
   8.2. NMR spectra of new compounds S94
9. References S143

1. General information

All chemicals and solvents used were purchased from commercial sources and were used without prior purification (Sigma-Aldrich, Fluorochem, Carbosynth, Iris Biotech GmbH). Solvents used for reactions, extractions and column chromatography were of technical grade. Anhydrous solvents for peptide coupling reactions (*N*,*N*-dimethylformamide (DMF) and tetrahydrofuran (THF) were stored over 4 Å molecular sieves (20% *V*/*V*). Water for RP-LC analysis was purified using a Millipore MilliQ water purification system. RP-LC solvents were of HPLC grade. Organic extracts were dried over technical grade anhydrous Na_2SO_4. Room temperature (rt) refers to 20–25 °C. For reactions carried out at higher temperatures, the reaction vessels were placed in a heating block.

^1H, ^{13}C and ^{19}F NMR spectra were recorded using a Bruker Avance III 500 MHz spectrometer (operating at 500 MHz for ^1H, 126 MHz for ^{13}C and 471 MHz for ^{19}F at 296 K) or Bruker Avance Neo 400 MHz spectrometer (operating at 400 MHz for ^1H, 101 MHz for ^{13}C and 376 MHz for ^{19}F at 296 K). NMR experiments were processed and analyzed using the MestReNova program. ^1H and ^{13}C chemical shifts are reported as δ scale in ppm relative to the residual signals of $CHCl_3$ (δ = 7.26 ppm and 77.16 ppm, respectively), DMSO-d_5 (δ = 2.50 ppm and 39.52 ppm, respectively), CHD_2OD (δ = 3.31 ppm and 49.00 ppm, respectively), and acetone-d_5 (δ = 2.05 ppm and 29.84 ppm, respectively). CCl_3F and C_6F_6 were used as an external reference for ^{19}F NMR spectra (respective chemical shift to the corresponding solvent).[1] Coupling constants (*J*) are given in hertz (Hz). Abbreviations for NMR data are s (singlet), bs (broad singlet), d (doublet), t (triplet), q (quartet), m (multiplet) and multiples thereof. NMR signal assignments and conformational populations were confirmed using 2D ^1H correlation spectroscopy (COSY), 2D ^1H nuclear Overhauser effect spectroscopy (NOESY), 2D ^1H–^{13}C heteronuclear multiple-bond correlation spectroscopy (HMBC) and 2D ^1H–^{13}C heteronuclear single quantum coherence spectroscopy (HSQC). The solvent signal in ^1H and 2D spectra of EM-1 analogues in MeOD-d_3 was suppressed by a Watergate sequence.

High-resolution mass spectra (HRMS) were acquired using an Agilent 6224 Accurate Mass TOF LC/MS or an Agilent QTOF 6530 (source ESI, resolution 12000). Infrared spectra (IR) were recorded using a Perkin-Elmer Spectrum BX FTIR with frequencies given in cm^{-1}. Melting points were determined using a Kofler micro hot stage and are uncorrected. The specific angles of rotation of optically pure compounds were measured using a Perkin-Elmer 241MC polarimeter (sodium D-line–589 nm, room temperature) at concentrations given in g/100 mL of the indicated solvent.

Solid-phase peptide synthesis (SPPS) was carried out manually or with the Liberty Blue 2.0 Microwave peptide synthesizer, using the standard Fmoc-strategy SPPS. Peptide syntheses were monitored using a reverse-phase (RP) ultra performance liquid chromatography coupled mass spectrometer (UPLC-MS). The analyses were performed on a Waters Acquity UPLC instrument equipped with a Luna Omega PS-C18 column (1.5 µm, 2.1 x 50 mm) and coupled to a single quadrupole ESI-MS (Micromass ZQ) under the following conditions: flow rate of 0.5 ml/min at room temperature; linear gradient of mobile phases mQ H_2O + 0.1% TFA and MeCN + 0.1% TFA; detection at 210 nm and 254 nm. RP high-performance liquid chromatography

(HPLC) preparative purification of peptides was performed using an Agilent 1260 Infinity II HPLC system equipped with a DAD detector under the following conditions: Luna Omega PS-C18 column, 5 µm, 100 Å, 250 x 10 mm; flow rate of 4.0 mL/min; injection volume 100–850 µL, linear gradient of mobile phases mQ H_2O + 0.1% TFA and MeCN + 0.1% TFA; detection at 215 nm and 250 nm. RP-HPLC analysis for determination of peptide purity and hydrophobicity index was performed on an Agilent 1200 analytical HPLC system equipped with a DAD detector. The RP-HPLC conditions were as follows: Zorbax Eclipse XDB-C18 column, 5 µm, 100 Å, 150 x 4.6 mm; flow rate of 1.0 mL/min; injection volume 10–25 µL; linear gradient of mobile phases mQ H_2O + 0.1% TFA, MeCN + 0.1% TFA, 50 mM ammonium acetate in mQ H_2O or MeCN; detection at 210 nm and 254 nm. Enantiopurity analysis of model AAs (**4b** and **6a**) was performed using the same HPLC instrument under the following normal phase conditions: Chiralpak IA-3 column; flow rate of 1.0 mL/min; mobile phase: *n*-hexane and isopropanol (*i*PrOH) + 0.1% TFA; detection at 254 nm.

Column chromatography (CC) was performed on silica gel (silica gel 60, particle size: 0.035–0.070 mm). Reactions were monitored by thin-layer chromatography (TLC) (Merck, silica gel 60 F_{254}, 0.25 nm) using mixtures of ethyl acetate (EtOAc), petroleum ether (PE), *n*-hexane (*n*-hex), cyclohexane (cy-hex), dichloromethane (DCM) or methanol (MeOH). Visualization was accomplished by irradiation with a UV lamp and/or staining with phosphomolybdic acid or ninhydrin followed by heating.

2. Preparation of starting materials

Compounds **(*S*)-2b** (Fluorochem Ltd.), **(*R*)-2b** (Fluorochem Ltd.), **2c** (Fluorochem Ltd.), **2e** (Sigma Aldrich), **2f** (Fluorochem Ltd.), **2i** (Fluorochem Ltd.), **5a** (Fluorochem Ltd.), **5c** (Fluorochem Ltd.), **5d** (Sigma Aldrich), **5f** (Alfa Aesar) and **5h** (Fluorochem Ltd.) were bought from commercial sources and used as is. The preparation of reagent **1** and the remaining starting substrates is described below.

N-(4-chlorophenyl)-*S*-(trifluoromethyl)thiohydroxylamine (1)

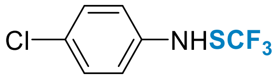

A dry 250 mL round-bottom flask was charged with diisopropylethylamine (7 ml, 40 mmol, 1.0 equiv) and DCM (80 mL). The resulting mixture was cooled to –25 °C before the dropwise addition of diethylaminosulfur trifluoride (5.9 mL, 47 mmol, 1.2 equiv) over 30 min, followed by the dropwise addition of trifluoromethyltrimethylsilane (6.5 mL, 44 mmol, 1.1 equiv) over 20 min. After an additional hour of stirring at –20 °C, 4-chloroaniline (5.1 mg, 50 mmol, 1.0 equiv) was added at 0 °C in several portions over 5 h. The reaction mixture was warmed to room temperature and was kept stirring overnight. Afterwards, the reaction mixture was washed with distilled water. The aqueous phase was extracted with DCM (2 x 80 mL) and the combined organics were dried over anhydrous Na_2SO_4, filtered and concentrated *in vacuo*. The crude residue was purified by silica gel CC (mobile phase: cyclohexane), which afforded the SCF_3 reagent **1**. The spectroscopic data is in agreement with the literature.[2]

Yield: 6.62 g (29.1 mmol, 73%) of yellow-orange oil.

^{1}H NMR (400 MHz, $CDCl_3$): δ = 7.23 (d, J = 9.0 Hz, 2H), 7.02 (d, J = 9.0 Hz, 2H), 5.11 (s, 1H).

^{13}C{^{1}H} NMR (126 MHz, $CDCl_3$): δ = 143.8, 129.4 (q, J = 317.4 Hz), 129.4, 127.0, 116.5.

^{19}F NMR (376 MHz, $CDCl_3$): δ = –52.76.

N-Fmoc-L-tryptophan ethyl ester (2a)

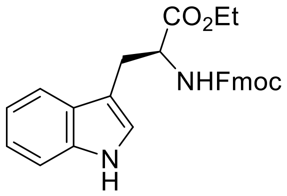

Prepared by adapting a literature procedure.[3] A 100 mL round-bottom flask was charged with ethyl L-tryptophan hydrochloride (1.05 g, 3.9 mmol, 1.0 equiv) and 10% Na_2CO_3 (aq.)/THF solution (2:1, 45 mL). The resulting mixture was stirred at 0 °C for 5 min, preceeding the dropwise addition of Fmoc-OSu (1.45 g, 4.3 mmol, 1.1 equiv) solution in 15 mL of THF. The reaction mixture was vigorously stirred overnight at room temperature. The residual THF was removed *in vacuo* and the mixture was extracted with EtOAc (3 x 30 mL). The combined organic layer was dried over anhydrous Na_2SO_4, filtered and concentrated *in vacuo*. The crude product was purified by silica gel CC (dry loading, mobile phase: EtOAc/cyclohexane 1:3 → 1:1; R_f = 0.10) which afforded the pure product **2a**.

Yield: 1.504 g (3.31 mmol, 85%; mixture of rotamers: 85:15) of white foamy solid.

mp 136–139 °C

$[\alpha]_D^{20}$ = +14.9 (*c* 1.50, DCM)

^{1}H NMR (500 MHz, $CDCl_3$) of the major rotamer: *δ* = 8.11 (s, 1H), 7.77 (d, *J* = 7.6 Hz, 2H), 7.61–7.53 (m, 3H), 7.40 (ddd, *J* = 7.6, 4.7, 2.8 Hz, 2H), 7.36 (d, *J* = 8.1, 1H), 7.33–7.28 (m, 2H), 7.23–7.18 (m, 1H), 7.16–7.11 (m, 1H), 6.96 (d, *J* = 2.4, 1H), 5.39 (d, *J* = 8.4, 1H), 4.74 (dt, *J* = 8.4, 5.5 Hz, 1H), 4.45–4.34 (m, 2H), 4.21 (t, *J* = 7.2 Hz, 1H), 4.19–4.08 (m, 2H), 3.34 (d, *J* = 5.5 Hz, 2H), 1.22 (t, *J* = 7.1 Hz, 3H).

^{13}C{^{1}H} NMR (126 MHz, $CDCl_3$): *δ* = 172.1, 155.9, 144.0, 143.9, 141.4, 141.4, 136.2, 127.8, 127.2, 125.3, 125.3, 122.9, 122.4, 120.1, 120.1, 119.9, 118.8, 111.4, 110.2, 67.1, 61.7, 54.7, 47.3, 28.1, 14.2.

IR (neat, cm^{-1}): 3381, 3324, 1729, 1698, 1525, 1442, 1265, 1225, 1077, 1039, 753, 738, 669, 561.

HRMS (ESI) m/z: $[M + H]^+$ Calcd for $C_{28}H_{27}N_2O_4$ 455.1965; Found: 455.1967.

N-Fmoc-tryptamine (2d)

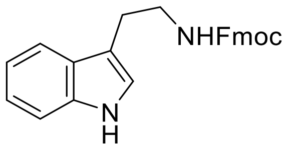

Prepared following a literature procedure[4] from tryptamine (80.1 mg, 0.50 mmol, 1.0 equiv), Fmoc-Cl (181.1 mg, 0.70 mmol, 1.4 equiv), Et_3N (83.6 µL, 0.6 mmol, 1.2 equiv), DCM (4 mL), 0 °C → rt, overnight. Purified by silica gel CC (mobile phase: EtOAc/*n*-hexane 1:4 → 1:2) to afford pure **2d**. The spectroscopic data is in agreement with the literature.[5]

Yield: 179 mg (0.47 mmol, 94%; mixture of rotamers: 84:16) of yellowish solid.

^{1}H NMR (500 MHz, $CDCl_3$) of the major rotamer: *δ* = 8.06 (s, 1H), 7.77 (d, J = 7.6 Hz, 2H), 7.63 (d, J = 8.0 Hz, 1H), 7.57 (d, J = 7.5 Hz, 2H), 7.44–7.39 (m, 2H), 7.38 (d, J = 8.1 Hz, 1H), 7.33–7.29 (m, 2H), 7.25–7.20 (m, 1H), 7.17–7.12 (m, 1H), 6.99 (d, J = 2.4 Hz, 1H), 4.87 (t, J = 6.1 Hz, 1H), 4.42 (d, J = 7.0 Hz, 2H), 4.22 (t, J = 7.0 Hz, 1H), 3.60–3.51 (m, 2H), 2.99 (t, J = 6.8 Hz, 2H).

^{13}C{^{1}H} NMR (126 MHz, $CDCl_3$): *δ* = 156.5, 144.1, 141.4, 136.5, 127.8, 127.2, 125.2, 122.3, 122.2, 120.1, 119.6, 118.9, 113.0, 111.4, 66.6, 47.4, 41.4, 25.9.

N-Fmoc-serotonin (2g)

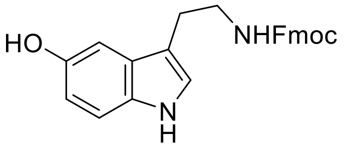

A 25 mL round-bottom flask was charged with serotonin hydrochloride (106 mg, 0.50 mmol, 1.0 equiv) and DCM (4 mL). The resulting mixture was stirred at 0 °C, Et_3N was added (105 µL, 0.75 mmol, 1.5 equiv), followed by a dropwise addition of Fmoc-OSu (169 mg, 0.50 mmol,

1.0 equiv) solution in DCM (2 mL). The reaction mixture was stirred overnight at room temperature and the volatile components were evaporated *in vacuo*. Then, 10 mL of H_2O was added and extracted with EtOAc three times. The combined organic layers were washed with brine, then dried over anhydrous Na_2SO_4, filtered and concentrated *in vacuo*. The crude product was purified by silica gel CC (dry loading, mobile phase: EtOAc/cyclohexane 1:2) which afforded the pure product **2g**.

Yield: 145 mg (0.36 mmol, 73%; mixture of rotamers: 83:17) of yellowish foamy solid.

mp 90–93 °C

^{1}H NMR (400 MHz, MeOD-*d₄*) of the major rotamer: δ = 7.77 (d, J = 7.6 Hz, 2H), 7.61 (d, J = 7.5 Hz, 2H), 7.37 (t, J = 7.5 Hz, 2H), 7.31–7.25 (m, 2H), 7.16 (d, J = 8.7 Hz, 1H), 6.98 (s, 1H), 6.96 (d, J = 2.3 Hz, 1H), 6.67 (dd, J = 8.6, 2.4 Hz, 1H), 4.31 (d, J = 6.9 Hz, 2H), 4.17 (t, J = 7.0 Hz, 1H), 3.41–3.33 (m, 2H), 2.85 (t, J = 7.4 Hz, 2H).

^{13}C{^{1}H} NMR (101 MHz, MeOD-d_4): δ = 158.9, 151.1, 145.3, 142.6, 133.1, 129.5, 128.7, 128.1, 126.2, 124.2, 120.9, 112.6, 112.4, 112.3, 103.5, 67.6, 48.5, 42.6, 26.8.

IR (neat, cm^{-1}): 3336, 3061, 2927, 2854, 1691, 1523, 1450, 1246, 1226, 1186, 1134, 759, 737, 620.

HRMS (ESI) m/z: $[M + H]^+$ Calcd for $C_{25}H_{23}N_2O_3$ 399.1703; Found: 399.1704.

_O_-benzyl-_N_-Fmoc-serotonin (2h)

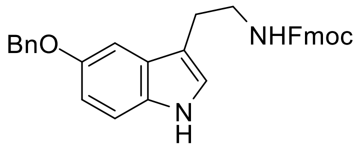

A 25 mL round-bottom flask was charged with *O*-benzylserotonin TFA salt (256 mg, 0.70 mmol, 1.0 equiv) and DCM (6 mL). The resulting mixture was stirred at 0 °C, Et_3N was added (124 µL, 0.89 mmol, 1.3 equiv), followed by a dropwise addition of Fmoc-OSu (299 mg, 0.89 mmol, 1.3 equiv) solution in DCM (4 mL). The reaction mixture was stirred overnight at room temperature. The reaction mixture was dilluted with DCM and washed with aq. 1 M HCl solution three times. The organic layer was then washed with brine, dried over anhydrous Na_2SO_4, filtered and concentrated *in vacuo*. The crude product was purified by silica gel CC (mobile phase: DCM) which afforded the pure product **2h**.

Yield: 247 mg (0.51 mmol, 72%) of white foamy solid.

mp 66–69 °C

^{1}H NMR (400 MHz, $CDCl_3$): δ = 7.92 (s, 1H), 7.76 (dt, J = 7.6, 0.9 Hz, 2H), 7.56 (d, J = 7.5 Hz, 2H), 7.46 (d, J = 7.5 Hz, 2H), 7.43–7.35 (m, 4H), 7.35–7.24 (m, 4H), 7.15 (d, J = 2.4 Hz, 1H), 6.99 (d, J = 2.5 Hz, 1H), 6.96 (dd, J = 8.8, 2.4 Hz, 1H), 5.10 (s, 2H), 4.85 (s, 1H), 4.41 (d, J = 7.0 Hz, 2H), 4.21 (t, J = 7.0 Hz, 1H), 3.57–3.48 (m, 2H), 2.95 (t, J = 6.8 Hz, 2H).

^{13}C{^{1}H} NMR (101 MHz, $CDCl_3$): δ = 156.5, 153.4, 144.1, 141.4, 137.7, 131.8, 128.6, 127.9, 127.8, 127.2, 125.2, 123.1, 120.1, 113.2, 112.8, 112.1, 102.4, 71.1, 66.7, 47.4, 41.2, 25.9 (2 signals missing or overlaping).

IR (neat, cm^{-1}): 3417, 3320, 3061, 3033, 2935, 1699, 1517, 1481, 1451, 1243, 1220, 1025, 1184, 759, 739, 697.

HRMS (ESI) m/z: $[M + H]^+$ Calcd for $C_{32}H_{29}N_2O_3$ 489.2173; Found: 489.2174.

N-Fmoc-D-tyrosine ((*R*)-5a)

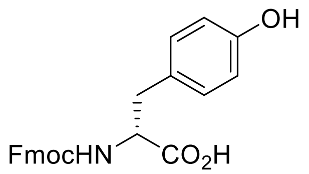

Prepared following a literature procedure[6] from D-tyrosine (2.23 g, 12.3 mmol, 1.0 equiv), Fmoc-OSu (4.16 g, 12.3 mmol, 1.0 equiv), $NaHCO_3$ (1.16 g, 13.8 mmol, 1.1 equiv), H_2O/acetone 1:1 (100 mL), rt, overnight. Purified by silica gel CC (mobile phase: DCM/MeOH 9/1) to afford pure **(*D*)-5a**. The spectroscopic data is in agreement with the literature.[7]

Yield: 1.62 g (4.03 mmol, 33%) of white foamy solid.

^{1}H NMR (400 MHz, DMSO-*d₆*): δ = 12.65 (s, 1H), 9.21 (s, 1H), 7.88 (d, *J* = 7.5 Hz, 2H), 7.70–7.62 (m, 3H), 7.41 (ddd, *J* = 7.5, 3.2, 1.1 Hz, 2H), 7.31 (ddd, *J* = 10.2, 7.5, 1.2 Hz, 2H), 7.06 (d, *J* = 8.5 Hz, 2H), 6.66 (d, *J* = 8.4 Hz, 2H), 4.24–4.15 (m, 3H), 4.08 (ddd, *J* = 10.3, 8.4, 4.4 Hz, 1H), 2.96 (dd, *J* = 13.8, 4.4 Hz, 1H), 2.75 (dd, *J* = 13.9, 10.3 Hz, 1H).

^{13}C{^{1}H} NMR (101 MHz, DMSO-d_6): δ = 173.5, 155.9, 155.9, 143.8, 143.8, 140.7, 130.0, 128.0, 127.6, 127.1, 125.3, 125.3, 120.1, 115.0, 65.6, 55.9, 46.6, 35.7.

N-Fmoc-L-tyrosine methyl ester (5b)

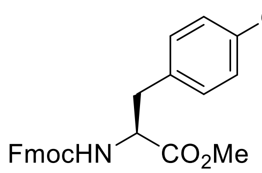

Prepared from L-tyrosine methyl ester hydrochloride (463 mg, 2.0 mmol, 1.0 equiv), Fmoc-Cl (569 mg, 2.2 mmol, 1.1 equiv), $NaHCO_3$ (504 mg, 6.0 mmol, 3.0 equiv), H_2O/dioxane 1:1 (10 mL), 0 °C → rt, overnight. Purified by silica gel CC (dry loading, mobile phase: EtOAc/petroleum ether 1:4 → 1:1) to afford pure **5b**. The spectroscopic data is in agreement with the literature.[8]

Yield: 751 mg (1.80 mmol, 90%; mixture of rotamers: 85:15) of white solid.

^{1}H NMR (500 MHz, $CDCl_3$) of the major rotamer: δ = 7.77 (d, *J* = 7.6 Hz, 2H), 7.59–7.54 (m, 2H), 7.44–7.38 (m, 2H), 7.35–7.28 (m, 2H), 6.94 (d, *J* = 8.3 Hz, 2H), 6.74 (d, *J* = 8.3 Hz, 2H), 5.25 (d, *J* = 8.4 Hz, 1H), 4.99 (s, 1H), 4.63 (dt, J = 8.4, 5.7 Hz, 1H), 4.44 (dd, J = 10.7, 7.2 Hz, 1H), 4.35 (dd, J = 10.7, 6.9 Hz, 1H), 4.21 (t, J = 7.1 Hz, 1H), 3.73 (s, 3H), 3.07 (dd, J = 14.0, 5.7 Hz, 1H), 3.02 (dd, J = 14.0, 5.9 Hz, 1H).

^{13}C{^{1}H} NMR (126 MHz, $CDCl_3$): δ = 172.3, 155.8, 155.1, 143.8, 141.4, 130.6, 127.9, 127.5, 127.2, 125.2, 125.2, 120.1, 120.1, 115.7, 67.2, 55.1, 52.6, 47.3, 37.6.

***N*-Fmoc-tyramine (5d)**

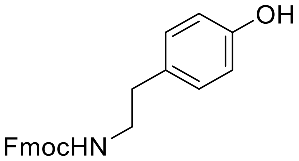

Prepared following a literature procedure[9] from tyramine (69 mg, 0.50 mmol, 1.0 equiv), Fmoc-Cl (142 mg, 0.55 mmol, 1.1 equiv), $NaHCO_3$ (42 mg, 0.50 mmol, 1.0 equiv), H_2O/THF 1:1 (6 mL), 0 °C → rt, overnight. Purified by silica gel CC (dry loading, mobile phase: EtOAc/*n*-hexane 1:2) to afford pure **5d**. The spectroscopic data is in agreement with the literature.[10]

Yield: 138 mg (0.38 mmol, 77%; mixture of rotamers: 89:11) of white solid.

^{1}H NMR (500 MHz, DMSO-*d*$_6$) of the major rotamer: δ = 9.16 (s, 1H), 7.89 (d, *J* = 7.5 Hz, 2H), 7.67 (d, *J* = 7.5 Hz, 2H), 7.46–7.39 (m, 2H), 7.36–7.30 (m, 3H), 6.96 (d, *J* = 8.4 Hz, 2H), 6.66 (d, *J* = 8.4 Hz, 2H), 4.28 (d, *J* = 7.0 Hz, 2H), 4.20 (t, *J* = 6.9 Hz, 1H), 3.13 (dt, *J* = 8.0, 6.0 Hz, 2H), 2.58 (t, *J* = 7.4 Hz, 2H).

^{13}C{^{1}H} NMR (126 MHz, DMSO-*d*$_6$): δ = 156.0, 155.6, 143.9, 140.7, 129.5, 127.6, 127.1, 125.2, 120.1, 115.1, 65.2, 46.8, 42.2, 34.6.

***N*-Fmoc-dopamine (5f)**

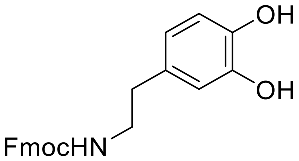

Prepared as **5b** from dopamine hydrochloride (569 mg, 3.0 mmol, 1.0 equiv), Fmoc-Cl (854 mg, 3.3 mmol, 1.1 equiv), $NaHCO_3$ (504 mg, 6.0 mmol, 2.0 equiv), H_2O/THF 1:1 (36 mL), 0 °C → rt, overnight. Purified by silica gel CC (dry loading, mobile phase: EtOAc/*n*-hexane 1:3) to afford pure **5f**. The spectroscopic data is in agreement with the literature.[11]

Yield: 949 mg (2.53 mmol, 84%; mixture of rotamers: 88:12) of white solid.

^{1}H NMR (500 MHz, DMSO-*d*$_6$) of the major rotamer: δ = 8.69 (s, 2H), 7.89 (d, *J* = 7.4 Hz, 2H), 7.67 (d, *J* = 7.5 Hz, 2H), 7.44–7.39 (m, 2H), 7.35–7.31 (m, 3H), 6.62 (d, *J* = 7.9 Hz, 1H), 6.57 (d, *J* = 2.1 Hz, 1H), 6.42 (dd, *J* = 8.1, 2.1 Hz, 1H), 4.28 (d, *J* = 7.0 Hz, 2H), 4.20 (t, *J* = 6.9 Hz, 1H), 3.11 (dt, *J* = 8.4, 6.0 Hz, 2H), 2.55–2.51 (m, 2H).

^{13}C{^{1}H} NMR (126 MHz, DMSO-*d*$_6$): δ = 156.0, 145.1, 143.9, 143.6, 140.7, 130.0, 129.0, 127.6, 127.3, 127.1, 125.2, 121.4, 120.1, 119.3, 116.0, 115.5, 109.8, 65.3, 46.8, 42.3, 35.0.

***N*-Fmoc-L-DOPA (5h)**

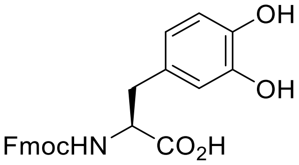

Prepared following a literature procedure[12] from L-DOPA (1.00 g, 5.07 mmol, 1.0 equiv), Fmoc-OSu (1.71 mg, 5.07 mmol, 1.0 equiv), Na_2CO_3 (1.00 g, 9.43 mmol, 1.9 equiv), H_2O/dioxane 1:1 (20 mL), 0 °C → rt, overnight. Purified by silica gel CC (mobile phase: DCM/MeOH 10:1) to afford pure **5h**. The spectroscopic data is in agreement with the literature.[12]

Yield: 1.52 g (3.62 mmol, 71%) of white foamy solid.

^{1}H NMR (400 MHz, DMSO-*d*$_6$): δ = 12.67 (s, 1H), 8.75 (s, 2H), 7.88 (d, *J* = 7.5 Hz, 2H), 7.69–7.62 (m, 3H), 7.41 (ddd, *J* = 7.5, 3.6, 1.1 Hz, 2H), 7.36–7.27 (m, 2H), 6.66 (d, *J* = 2.1 Hz, 1H), 6.62 (d, *J* = 8.0 Hz, 1H), 6.51 (dd, *J* = 8.1, 2.1 Hz, 1H), 4.23–4.16 (m, 3H), 4.07 (ddd, *J* = 10.2, 8.4, 4.5 Hz, 1H), 2.89 (dd, *J* = 13.8, 4.6 Hz, 1H), 2.69 (dd, *J* = 13.9, 10.2 Hz, 1H).

^{13}C{^{1}H} NMR (101 MHz, DMSO-*d*$_6$): δ = 173.6, 156.0, 144.9, 143.8, 143.8, 140.7, 128.7, 127.6, 127.1, 125.4, 125.3, 120.1, 119.8, 116.4, 115.3, 65.7, 55.9, 46.6, 36.0.

N-Fmoc-L-DOPA benzyl ester (5i)

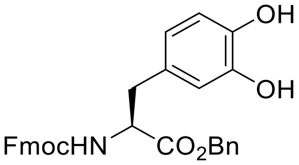

Prepared from L-DOPA benzyl ester TFA salt (620 mg, 1.54 mmol, 1.0 equiv), Fmoc-OSu (520 mg, 1.54 mmol, 1.0 equiv), $NaHCO_3$ (285.7 mg, 3.08 mmol, 2.0 equiv), H_2O/acetone 1:2 (15 mL), 0 °C → rt, overnight. Purified by silica gel CC (mobile phase: EtOAc/cyclohexane 2:3) to afford pure **5i**. The spectroscopic data is in agreement with the literature.[13]

Yield: 458 mg (0.95 mmol, 62%; mixture of rotamers: 86:14) of yellowish solid.

^{1}H NMR (400 MHz, MeOD-*d*$_4$) of the major rotamer: δ = 7.78 (d, *J* = 7.6 Hz, 2H), 7.61–7.57 (m, 2H), 7.37 (dd, *J* = 8.3, 6.9 Hz, 2H), 7.31–7.24 (m, 7H), 6.67 (d, *J* = 2.2 Hz, 1H), 6.66 (d, *J* = 8.1 Hz, 1H), 6.51 (dd, *J* = 8.0, 2.1 Hz, 1H), 5.11 (s, 2H), 4.40 (dd, *J* = 8.8, 6.1 Hz, 1H), 4.28–4.24 (m, 2H), 4.15 (t, *J* = 7.2 Hz, 1H), 2.98 (dd, *J* = 13.8, 6.1 Hz, 1H), 2.83 (dd, *J* = 13.8, 8.8 Hz, 1H).

^{13}C{^{1}H} NMR (101 MHz, MeOD-*d*$_4$): δ = 173.6, 158.4, 146.3, 145.3, 145.2, 145.1, 142.5, 137.1, 129.5, 129.2, 128.8, 128.2, 126.3, 126.3, 121.7, 120.9, 117.2, 116.3, 68.1, 67.9, 57.5, 48.3, 38.1.

General procedure for solution-phase peptide coupling. A round-bottom flask equipped with a magnetic stirr bar, was charged with the *N*-protected amino acid or peptide (1.0 equiv), and dissolved in THF (previously distilled and stored over 4 Å molecular sieves) under an argon atmosphere. The solution was cooled in an ice bath before adding *N*-methylmorpholine (NMM) (1.05 equiv), followed by a dropwise addition of isobutyl chloroformate (IBCF) (1.1 equiv). The resulting mixture was stirred in an ice bath for 5 min, then the solution of *C*-protected amino acid or peptide in dry DMF (stored over 4 Å molecular sieves) was added dropwise. After the addition, it was treated with NMM (1.05 equiv). The reaction mixture was warmed to room temperature and stirred overnight. The mixture was diluted with EtOAc and distilled H_2O. The resulting aqueous phase was extracted three times with EtOAc. The

combined organics were treated with a series of washes with sat. $NaHCO_3$ (aq.), distilled H_2O, 1 M HCl (aq.), distilled H_2O, and lastly with brine. The organic phase was dried over anhydrous Na_2SO_4, filtered, and the volatiles were removed *in vacuo*. The crude product was purified by silica gel CC to obtain the pure peptide, or it was used for further transformations without purification if indicated.

Fmoc-L-Trp-L-Leu-OBn (7a)

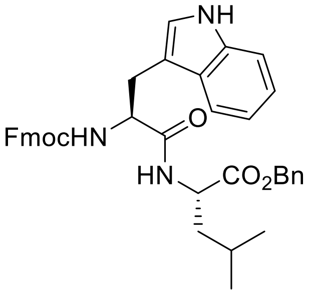

Prepared according to the general procedure from Fmoc-L-tryptophan **2b** (213 mg, 0.54 mmol, 1.1 equiv), L-leucine benzyl ester TFA salt (168 mg, 0.50 mmol, 1.0 equiv), NMM (2 x 58 µL, 1.10 mmol, 2.2 equiv), IBCF (71 µL, 0.55 mmol, 1.1 equiv), THF (5 mL), DMF (5 mL), 0 °C → rt, overnight. Following the general work-up procedure, **7a** was purified by silica gel CC (mobile phase: EtOAc/*n*-hex 1:1).

Yield: 218 mg (0.37 mmol, 69%) of yellow solid.

mp 100–103 °C

^{1}H NMR (400 MHz, $CDCl_3$): δ = 7.99 (s, 1H), 7.77 (d, *J* = 7.5 Hz, 2H), 7.72 (d, *J* = 8.1 Hz, 1H), 7.60–7.53 (m, 2H), 7.45–7.28 (m, 10H), 7.23–7.17 (m, 1H), 7.17–7.11 (m, 1H), 6.98 (s, 1H), 6.09 (d, *J* = 8.1 Hz, 1H), 5.62 (d, *J* = 7.7 Hz, 1H), 5.09 (s, 2H), 4.62–4.50 (m, 2H), 4.47–4.32 (m, 2H), 4.20 (t, *J* = 7.1 Hz, 1H), 3.34 (dd, *J* = 14.8, 5.1 Hz, 1H), 3.15 (dd, *J* = 14.5, 7.8 Hz, 1H), 1.53 (dq, *J* = 12.6, 6.5 Hz, 1H), 1.40 (ddq, *J* = 12.1, 7.1 Hz, 2H), 0.83 (d, *J* = 7.3 Hz, 3H), 0.81 (d, *J* = 6.2 Hz, 3H).

^{13}C{^{1}H} NMR (101 MHz, $CDCl_3$): δ = 172.3, 171.1, 156.1, 144.0, 143.9, 141.4, 136.3, 135.5, 128.7, 128.6, 128.4, 127.8, 127.4, 127.2, 125.2, 123.6, 122.4, 120.1, 120.1, 120.0, 119.0, 111.3, 67.1, 55.5, 51.1, 47.2, 41.6, 28.8, 24.8, 22.7, 22.1.

IR (neat, cm^{-1}): 3433, 3299, 3062, 2955, 1730, 1686, 1650, 1532, 1451, 1387, 1324, 1265, 1248, 1142, 1101, 1083, 1032, 968, 755, 733, 697, 621.

HRMS (ESI) m/z: $[M + H]^+$ Calcd for $C_{39}H_{40}N_3O_5$ 630.2962; Found: 630.2953.

Boc-L-Trp-L-Leu-OMe (7b')

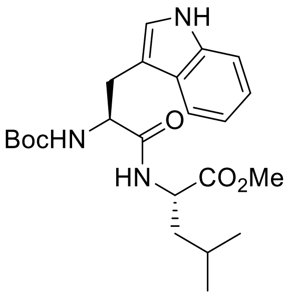

Prepared according to the general procedure from Boc-L-tryptophan (913 mg, 3.0 mmol, 1.0 equiv), L-leucine methyl ester hydrochloride (545 mg, 3.0 mmol, 1.0 equiv), NMM (2 x 346 µL, 6.3 mmol, 2.1 equiv), IBCF (428 µL, 3.3 mmol, 1.1 equiv), THF (10 mL), DMF (10 mL), 0 °C → rt, overnight. Following the general work-up procedure, **7b'** was purified by silica gel CC (mobile phase: EtOAc/*n*-hex 1:1). The spectroscopic data is in agreement with the literature.[14]

Yield: 840 mg (1.95 mmol, 65%) of yellowish foamy solid.

^{1}H NMR (500 MHz, $CDCl_3$): δ = 8.20 (s, 1H), 7.66 (d, *J* = 7.9 Hz, 1H), 7.35 (dd, *J* = 8.1, 1.0 Hz, 1H), 7.19 (ddd, *J* = 8.1, 6.9, 1.2 Hz, 1H), 7.12 (ddd, *J* = 8.0, 7.0, 1.1 Hz, 1H), 7.09 (s, 1H), 6.20 (d, *J* = 8.1 Hz, 1H), 5.18 (s, 1H), 4.52 (td, *J* = 8.5, 5.2 Hz, 1H), 4.44 (s, 1H), 3.64 (s, 3H), 3.29 (dd, *J* = 14.6, 5.4 Hz, 1H), 3.18 (dd, *J* = 14.7, 7.5 Hz, 1H), 1.55–1.34 (m, 12H), 0.85 (d, *J* = 7.1 Hz, 3H), 0.84 (d, *J* = 6.4 Hz, 3H).

^{13}C{^{1}H} NMR (126 MHz, $CDCl_3$): δ = 173.0, 171.5, 155.6, 136.3, 127.6, 123.4, 122.3, 119.9, 119.0, 111.3, 110.8, 80.2, 55.2, 52.3, 50.9, 41.7, 28.4, 28.2, 24.7, 22.8, 22.0.

TFA·H_2N-L-Trp-L-Leu-OMe (7b)

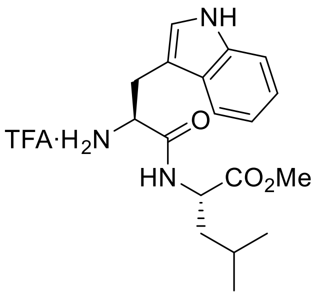

Prepared from Boc-L-Trp-L-Leu-OMe **7b'** (625 mg, 1.45 mmol, 1.0 equiv), trifluoroacetic acid (TFA; 2.5 mL, 32 mmol, 22 equiv), DCM (5 mL), 0 °C → rt, 12 h. Isolation: After the reaction completion (monitored by TLC analysis), the solvent was removed *in vacuo*. The residual TFA was co-evaporated with DCM 3-times. 7b was purified by silica gel CC (mobile phase: DCM/MeOH 10:1). The ^{1}H spectroscopic data is in agreement with the literature.[14a]

Yield: 499 mg (1.12 mmol, 77%) of yellow oil.

^{1}H NMR (500 MHz, $CDCl_3$): δ = 8.62 (s, 1H), 7.55 (d, *J* = 7.9 Hz, 1H), 7.42 (d, *J* = 7.5 Hz, 1H), 7.32 (d, *J* = 8.1 Hz, 1H), 7.15 (d, *J* = 2.4 Hz, 1H), 7.09 (t, *J* = 7.4 Hz, 1H), 7.04 (t, *J* = 7.4 Hz, 1H), 4.39 (q, *J* = 7.1, 5.6 Hz, 1H), 4.24 (t, *J* = 7.0 Hz, 1H), 3.63 (s, 3H), 3.36 (dd, *J* = 15.0, 6.2 Hz, 1H), 3.23 (dd, *J* = 14.9, 7.6 Hz, 1H), 1.49 (td, *J* = 19.3, 16.9, 6.7 Hz, 3H), 0.81 (d, *J* = 5.9 Hz, 3H), 0.78 (d, *J* = 5.8 Hz, 3H).

$^{13}C\{^1H\}$ NMR (126 MHz, $CDCl_3$): δ = 173.0, 168.8, 136.5, 126.9, 125.0, 122.5, 119.9, 118.4, 111.8, 107.4, 54.2, 52.6, 51.6, 40.7, 27.5, 24.7, 22.6, 21.7.

Fmoc-L-Ala-L-Trp-OBn (7c)

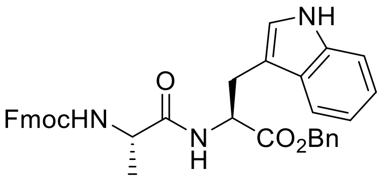

Prepared according to the general procedure from Fmoc-L-alanine (934 mg, 3.0 mmol, 1.2 equiv), L-tryptophan benzyl ester TFA salt (988 mg, 2.41 mmol, 1.0 equiv), NMM (2 x 346 µL, 6.3 mmol, 2.6 equiv), IBCF (428 µL, 3.3 mmol, 1.4 equiv), THF (5 mL), DMF (5 mL), 0 °C → rt, overnight. Following the general work-up procedure, **7c** was purified by silica gel CC (mobile phase: EtOAc/*n*-hex 1:1).

Yield: 950 mg (1.62 mmol, 67%) of yellow solid.

mp 145–149 °C

^1H NMR (400 MHz, $CDCl_3$): δ = 7.95 (s, 1H), 7.78 (d, *J* = 7.6 Hz, 2H), 7.59–7.54 (m, 2H), 7.48 (d, *J* = 7.9 Hz, 1H), 7.44–7.38 (m, 2H), 7.36–7.28 (m, 5H), 7.25–7.21 (m, 3H), 7.16–7.10 (m, 1H), 7.10–7.01 (m, 1H), 6.74 (d, *J* = 2.3 Hz, 1H), 6.62 (d, *J* = 7.9 Hz, 1H), 5.34 (d, *J* = 7.9 Hz, 1H), 5.07 (d, *J* = 2.5 Hz, 2H), 4.97 (dt, *J* = 7.9, 5.4 Hz, 1H), 4.40–4.31 (m, 1H), 4.31–4.20 (m, 2H), 4.17 (t, *J* = 7.1 Hz, 1H), 3.31 (dd, *J* = 5.5, 2.5 Hz, 2H), 1.30 (d, *J* = 7.0 Hz, 3H).

$^{13}C\{^1H\}$ NMR (101 MHz, $CDCl_3$): δ = 172.1, 171.5, 155.9, 144.0, 141.4, 136.1, 135.3, 128.7, 128.6, 127.9, 127.7, 127.2, 125.3, 125.2, 123.2, 122.3, 120.1, 119.8, 118.6, 111.4, 109.6, 67.4, 67.2, 53.1, 50.5, 47.1, 27.6, 18.8.

IR (neat, cm^{-1}): 3454, 3296, 3061, 2964, 2935, 1737, 1689, 1645, 1528, 1450, 1336, 1245, 1081, 1045, 754, 730, 696, 646, 589, 544.

HRMS (ESI) m/z: $[M + H]^+$ Calcd for $C_{36}H_{34}N_3O_5$ 588.2493; Found: 588.2495.

Fmoc-L-Ala-L-Trp-OEt (7d)

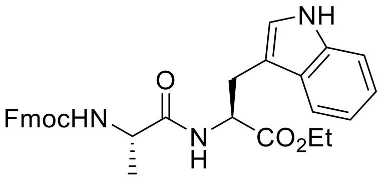

Prepared according to the general procedure from Fmoc-L-alanine (623 mg, 2.0 mmol, 1.0 equiv), L-Trp ethyl ester hydrochloride (538 mg, 2.0 mmol, 1.0 equiv), NMM (2 x 231 µL, 4.2 mmol, 2.1 equiv), IBCF (285 µL, 2.2 mmol, 1.1 equiv), THF (5 mL), DMF (5 mL), 0 °C → rt, overnight. Following the general work-up procedure, **7d** was purified by silica gel CC (mobile phase: EtOAc/*n*-hex 1:1).

Yield: 393 mg (0.75 mmol, 37%; mixture of rotamers: 86:14) of yellowish solid.

mp 139–143 °C

^{1}H NMR (500 MHz, $CDCl_3$) of the major rotamer: δ = 8.03 (s, 1H), 7.78 (d, J = 7.6 Hz, 2H), 7.59–7.54 (m, 2H), 7.51 (d, J = 7.9 Hz, 1H), 7.44–7.39 (m, 2H), 7.34–7.29 (m, 2H), 7.26 (d, J = 8.1 Hz, 1H), 7.16–7.11 (m, 1H), 7.09–7.04 (m, 1H), 6.92 (d, J = 2.4 Hz, 1H), 6.58 (d, J = 7.9 Hz, 1H), 5.34 (d, J = 7.9 Hz, 1H), 4.89 (dt, J = 7.9, 5.5 Hz, 1H), 4.38–4.33 (m, 1H), 4.28–4.21 (m, 2H), 4.17 (t, J = 7.1 Hz, 1H), 4.15–4.06 (m, 2H), 3.36–3.25 (m, 2H), 1.32 (d, J = 7.0 Hz, 3H), 1.21 (t, J = 7.2 Hz, 3H).

$^{13}C\{^{1}H\}$ NMR (126 MHz, $CDCl_3$): δ = 172.0, 171.7, 156.0, 144.0, 141.4, 136.1, 127.9, 127.7, 127.2, 125.3, 125.2, 123.1, 122.3, 120.1, 119.7, 118.6, 111.4, 109.8, 67.2, 61.7, 53.1, 50.5, 47.1, 27.6, 18.9, 14.2.

IR (neat, cm^{-1}): 3451, 3294, 2978, 1731, 1688, 1645, 1527, 1449, 1334, 1246, 1200, 1120, 1103, 1078, 1031, 860, 755, 729, 689, 646, 621.

HRMS (ESI) m/z: $[M + H]^+$ Calcd for $C_{31}H_{32}N_3O_5$ 526.2336; Found: 526.2329.

Fmoc-L-Ala-L-Trp-L-Leu-OMe (7e)

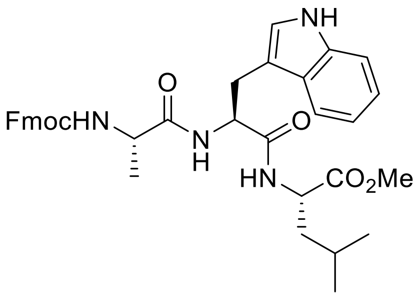

Prepared according to the general procedure from Fmoc-L-alanine (311 mg, 1.0 mmol, 1.0 equiv), TFA·H_2N-L-Trp-L-Leu-OMe **7b** (445 mg, 1.0 mmol, 1.0 equiv), NMM (2 x 115 µL, 2.1 mmol, 2.1 equiv), IBCF (143 µL, 1.1 mmol, 1.1 equiv), THF (5 mL), DMF (5 mL), 0 °C → rt, overnight. Following the general work-up procedure, **7e** was purified by silica gel CC (mobile phase: EtOAc/*n*-hex 1:1).

Yield: 252 mg (0.40 mmol, 40%; mixture of rotamers: 90:10) of yellowish solid.

mp 162–164 °C

^{1}H NMR (500 MHz, $CDCl_3$) of the major rotamer: δ = 8.01 (s, 1H), 7.79 (d, J = 7.6 Hz, 2H), 7.66 (d, J = 7.8 Hz, 1H), 7.59–7.55 (m, 2H), 7.44–7.40 (m, 2H), 7.35–7.30 (m, 2H), 7.24 (d, J = 8.1 Hz, 1H), 7.15–7.11 (m, 1H), 7.08 (ddd, J = 7.5, 7.1, 1.1 Hz, 1H), 7.02 (d, J = 2.4 Hz, 1H), 6.80 (d, J = 7.6 Hz, 1H), 6.36 (d, J = 8.0 Hz, 1H), 5.29 (d, J = 7.2 Hz, 1H), 4.74 (td, J = 7.5, 5.7 Hz, 1H), 4.47 (td, J = 8.2, 5.5 Hz, 1H), 4.37 (dd, J = 10.3, 6.8 Hz, 1H), 4.27–4.19 (m, 2H), 4.17 (t, J = 6.9 Hz, 1H), 3.65 (s, 3H), 3.32 (dd, J = 14.7, 5.7 Hz, 1H), 3.17 (dd, J = 14.7, 7.4 Hz, 1H), 1.49 (dd, J = 12.4, 6.4 Hz, 1H), 1.45–1.35 (m, 2H), 1.32 (d, J = 6.2 Hz, 3H), 0.80 (d, J = 3.8 Hz, 6H).

$^{13}C\{^{1}H\}$ NMR (126 MHz, $CDCl_3$): δ = 173.0, 172.1, 170.9, 156.1, 143.9, 143.8, 141.4, 141.4, 136.2, 128.0, 127.5, 127.3, 127.2, 125.3, 125.2, 123.5, 122.4, 120.2, 119.9, 118.9, 111.3, 110.4, 67.2, 53.7, 52.4, 51.1, 50.8, 47.1, 41.3, 28.0, 24.8, 22.7, 22.0, 18.6.

IR (neat, cm^{-1}): 3368, 3332, 3281, 2954, 1750, 1705, 1673, 1643, 1533, 1506, 1448, 1290, 1251, 1200, 1154, 1107, 1081, 1031, 758, 738.

HRMS (ESI) m/z: $[M + H]^+$ Calcd for $C_{36}H_{41}N_4O_6$ 625.3021; Found: 625.3009.

Fmoc-L-Trp-L-Trp-OEt (7f)

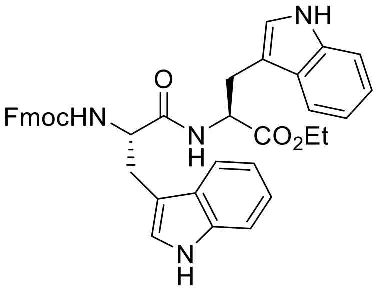

Prepared according to the general procedure from Fmoc-L-tryptophan **2b** (1.07 g, 2.5 mmol, 1.0 equiv), L-tryptophan ethyl ester hydrochloride (672 mg, 1.0 mmol, 1.0 equiv), NMM (2 x 287 µL, 5.25 mmol, 2.1 equiv), IBCF (357 µL, 2.75 mmol, 1.1 equiv), THF (10 mL), DMF (10 mL), 0 °C → rt, overnight. Following the general work-up procedure, **7f** was purified by silica gel CC (mobile phase: EtOAc/cy-hex 1:1).

Yield: 1.49 g (2.33 mmol, 93%; mixture of rotamers: 87:13) of white foamy solid.

mp 95–98 °C

^{1}H NMR (400 MHz, $CDCl_3$) of the major rotamer: δ = 7.90 (s, 1H), 7.87 (s, 1H), 7.77 (d, J = 7.6 Hz, 2H), 7.69 (d, J = 7.9 Hz, 1H), 7.54 (d, J = 7.4 Hz, 1H), 7.48 (d, J = 7.4 Hz, 1H), 7.44–7.37 (m, 2H), 7.35–7.19 (m, 6H), 7.17–7.07 (m, 2H), 6.94–6.84 (m, 2H), 6.59 (s, 1H), 6.29 (d, J = 7.7 Hz, 1H), 5.47 (d, J = 8.0 Hz, 1H), 4.88–4.72 (m, 1H), 4.61–4.50 (m, 1H), 4.41–4.32 (m, 1H), 4.30–4.22 (m, 1H), 4.15 (t, J = 7.1 Hz, 1H), 4.05 (q, J = 8.0 Hz, 2H), 3.34 (dd, J = 14.8, 5.0 Hz, 1H), 3.15 (m, 2H), 3.15–3.04 (m, 1H), 1.18 (t, J = 7.2 Hz, 3H).

^{13}C{^{1}H} NMR (101 MHz, $CDCl_3$): δ = 171.4, 170.9, 156.0, 143.9, 141.4, 136.3, 136.0, 127.9, 127.5, 127.2, 125.3, 123.8, 123.0, 122.4, 122.3, 120.1, 119.7, 119.0, 118.6, 111.3, 110.4, 109.7, 67.2, 61.6, 55.5, 52.9, 47.2, 28.5, 27.6, 14.2 (3 signals missing or overlaping).

IR (neat, cm^{-1}): 3409, 3320, 3057, 2980, 2931, 1710, 1659, 1507, 1458, 1235, 1207, 1032, 738, 538.

HRMS (ESI) m/z: $[M + H]^+$ Calcd for $C_{39}H_{37}N_4O_5$ 641.2758; Found: 641.2757.

Fmoc-L-Tyr-L-Trp-OEt (7g)

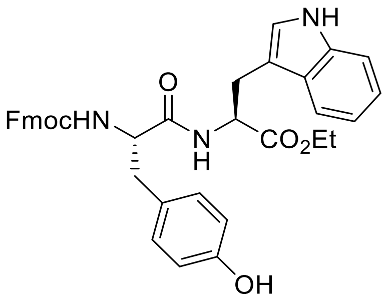

Prepared according to the general procedure from Fmoc-L-tyrosine **5a** (1.01 g, 2.5 mmol, 1.0 equiv), L-tryptophan ethyl ester hydrochloride (672 mg, 1.0 mmol, 1.0 equiv), NMM (2 x 288 µL, 5.25 mmol, 2.1 equiv), IBCF (357 µL, 2.75 mmol, 1.1 equiv), THF (10 mL), DMF (10 mL), 0 °C → rt, overnight. Following the general work-up procedure, **7g** was purified by silica gel CC (mobile phase: EtOAc/*n*-hex 1:1).

Yield: 1.07 g (1.74 mmol, 70%; mixture of rotamers: 88:12) of yellowish solid.

mp 181–185 °C

^{1}H NMR (500 MHz, DMSO-*d*$_6$) of the major rotamer: δ = 10.89 (d, *J* = 2.4 Hz, 1H), 9.16 (s, 1H), 8.44 (d, *J* = 7.3 Hz, 1H), 7.88 (dd, *J* = 7.6, 1.1 Hz, 2H), 7.65 (d, *J* = 7.8 Hz, 1H), 7.62 (d, *J* = 7.5 Hz, 1H), 7.53 (d, *J* = 8.8 Hz, 1H), 7.50 (d, *J* = 7.9 Hz, 1H), 7.40 (ddd, *J* = 7.5, 3.4, 1.1 Hz, 2H), 7.35–7.26 (m, 3H), 7.18 (d, *J* = 2.4 Hz, 1H), 7.09 (d, *J* = 8.5 Hz, 2H), 7.08–7.04 (m, 1H), 6.99 (ddd, *J* = 7.9, 7.0, 1.1 Hz, 1H), 6.63 (d, *J* = 8.5 Hz, 2H), 4.52 (td, *J* = 7.5, 6.2 Hz, 1H), 4.23 (ddd, *J* = 10.8, 8.9, 3.8 Hz, 1H), 4.20–4.09 (m, 3H), 4.00 (q, *J* = 7.1 Hz, 2H), 3.17 (dd, *J* = 14.8, 6.2 Hz, 1H), 3.10 (dd, *J* = 14.7, 7.8 Hz, 1H), 2.87 (dd, *J* = 13.8, 3.8 Hz, 1H), 2.64 (dd, *J* = 13.8, 10.7 Hz, 1H), 1.06 (t, *J* = 7.1 Hz, 3H).

^{13}C{^{1}H} NMR (126 MHz, DMSO-*d*$_6$): δ = 172.0, 171.7, 155.8, 143.8, 143.7, 140.7, 136.1, 130.2, 128.2, 127.6, 127.1, 127.1, 127.1, 125.4, 125.3, 123.7, 121.0, 120.1, 118.4, 118.0, 114.8, 111.4, 109.3, 65.6, 60.5, 56.2, 53.2, 46.6, 36.7, 27.0, 13.9.

IR (neat, cm^{-1}): 3467, 3419, 3338, 1739, 1708, 1654, 1532, 1518, 1490, 1452, 1305, 1267, 1228, 1191, 1085, 1033, 830, 802, 741, 679, 650.

HRMS (ESI) m/z: $[M + H]^+$ Calcd for $C_{37}H_{36}N_3O_6$ 618.2599; Found: 618.2585.

Fmoc-L-Ala-L-Tyr-OMe (7h)

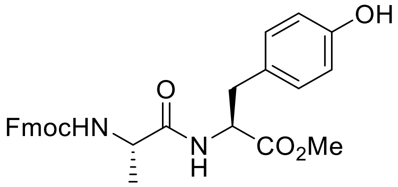

Prepared according to the general procedure from Fmoc-L-alanine (934 mg, 3.0 mmol, 1.0 equiv), L-tyrosine methyl ester hydrochloride (695 mg, 1.0 mmol, 1.0 equiv), NMM (2 x 346 µL, 6.30 mmol, 2.1 equiv), IBCF (428 µL, 3.30 mmol, 1.1 equiv), THF (10 mL), DMF (10 mL), 0 °C → rt, overnight. Following the general work-up procedure, **7h** was purified by silica gel CC (mobile phase: EtOAc/*n*-hex 1:1). The spectroscopic data is in agreement with the literature.[8]

Yield: 1.28 g (2.62 mmol, 87%; mixture of rotamers: 87:13) of white solid.

^{1}H NMR (400 MHz, $CDCl_3$) of the major rotamer: δ = 7.76 (d, J = 7.5 Hz, 2H), 7.58 (d, J = 7.5 Hz, 2H), 7.40 (dd, J = 7.4, 1.2 Hz, 2H), 7.30 (dd, J = 7.5, 1.2 Hz, 2H), 6.90 (d, J = 8.0 Hz, 2H), 6.64 (d, J = 7.9 Hz, 2H), 6.54 (d, J = 8.0 Hz, 1H), 5.89 (s, 1H), 5.39 (d, J = 7.7 Hz, 1H), 4.82 (dt, J = 8.1, 5.8 Hz, 1H), 4.40 (dd, J = 10.4, 7.3 Hz, 1H), 4.35–4.29 (m, 1H), 4.27–4.16 (m, 2H), 3.71 (s, 3H), 3.08 (dd, J = 14.1, 5.5 Hz, 1H), 2.98 (dd, J = 14.0, 5.9 Hz, 1H), 1.34 (d, J = 7.0 Hz, 3H).

^{13}C{^{1}H} NMR (101 MHz, $CDCl_3$): δ = 172.1, 171.9, 156.2, 155.3, 143.9, 141.4, 141.4, 130.5, 127.9, 127.3 (d, J = 2.1), 125.3, 125.2, 120.2, 115.7, 67.4, 53.5, 52.6, 50.5, 47.2, 37.1, 18.6.

3. Trifluoromethylthiolation reactions

3.1. General trifluoromethylthiolation procedure

A 5 mL reaction tube equipped with a magnetic stir bar, was charged with the substrate (0.10 mmol, 1.0 equiv), SCF_3 reagent **1** (0.11–0.25 mmol, 1.1–2.5 equiv), and the specified solvent (DCM or DCE, 1 mL, 0.1 M). After stirring the reaction mixture at room temperature for 5 min, the selected Brønsted or Lewis acid was added (0.25–0.50 mmol, 2.5–5.0 equiv; in two portions, if indicated). The resulting mixture was stirred at the specified temperature (rt or 50 °C) for the indicated time. The reaction mixture was then diluted with DCM and an aqueous workup was performed. The organic phase was dried over anhydrous Na_2SO_4, filtered, concentrated *in vacuo*, and purified by silica gel CC, unless otherwise indicated.

3.2. Synthesis and structural elucidation of (CF_3S)-hexahydropyrrolo[2,3-*b*]indole 3a

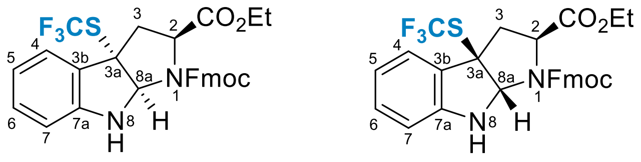

(2*S*,3a*S*,8a*R*)
***endo-cis*-3a**

(2*S*,3a*R*,8a*S*)
***exo-cis*-3a**

Figure S1: (2*S*,3a*R*,8a*S*)-**3a** and (2*S*,3a*S*,8a*R*)-**3a** structure and the (CF_3S)-hexahydropyrrolo[2,3-*b*]indole atom numbering system.

A 50 mL round-bottom flask equipped with a magnetic stirr bar, was charged with Fmoc-Trp-OEt **2a** (272.7 mg, 0.60 mmol, 1.2 equiv), SCF_3 reagent **1** (136.6 mg, 0.60 mmol, 1.2 equiv), and DCM (5 mL). After stirring the reaction mixture at room temperature for 5 min, TfOH (44 µL, 0.50 mmol, 1.0 equiv) was slowly added. The reaction mixture was stirred at room temperature and monitored by ^1H and ^{19}F NMR spectroscopy (20 µL aliquots). After 8 h, the reaction was dilluted with 50 mL of DCM and washed with 1 M HCl (aq.) solution (50 mL), brine (50 mL) and sat. $NaHCO_3$ (aq.) (50 mL). The organic phase was dried over anhydrous Na_2SO_4, filtered, concentrated *in vacuo*, and purified by silica gel CC (mobile phase: DCM) to obtain separated *endo-cis* and *exo-cis* diastereoisomers **3a** as semi-solids in a combined yield of 50%.

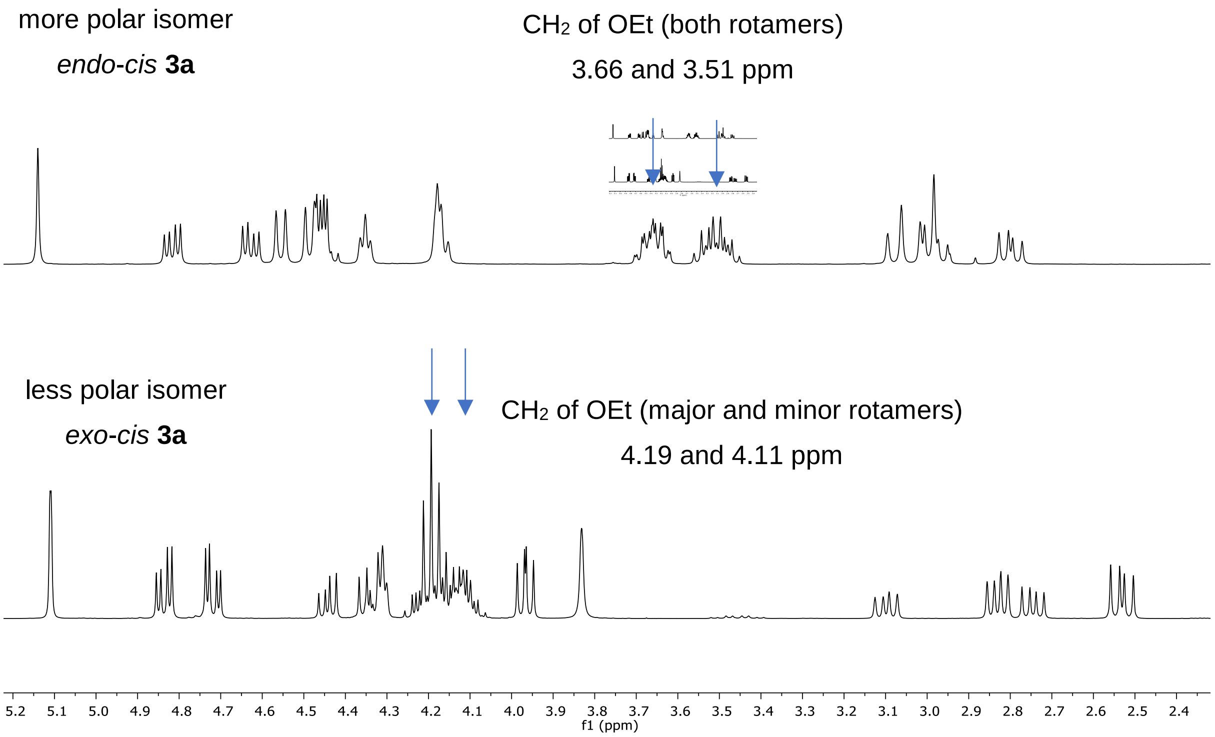

Figure S2: ^1H NMR spectra (400 MHz, $CDCl_3$, 25 °C) of ***endo-cis*-3a** and ***exo-cis*-3a**.

The diastereoisomeric assignment was done based on the characteristic shielding of the ester function by the aromatic ring for the *endo-cis* isomer **3a**. According to the literature, a characteristic upfield shift of the methyl ester groups to ~3.1 ppm is typical for the *endo-cis* isomers, whereas the *exo-cis* isomer geometry does not exhibit the shielding effect.[15] The separated diastereoisomers **3a** were analyzed by ^1H and ^{19}F NMR spectroscopy (Figure S2). The CH_2 signals of the ethyl ester group of the more polar diastereoisomer 3**a** were significantly shifted upfield (3.5–3.6 ppm), compared with the less polar diastereoisomer **3a** with characteristic signals at 4.1–4.2 ppm. Therefore, the *endo*- and *exo*- configurations could be assigned, respectively.

Additional NOESY and ^1H–^{19}F HOESY experiments (2.5 mM and 20 mM in $CDCl_3$, 400 MHz) were performed, however no correlations that would give a conclusive absolute structure were observed (e.g. H_{8a}–H_2). Both diastereoisomers exhibited an additional set of signals in the ^1H and ^{19}F NMR spectra, because of the presence of rotamers (***endo-cis*-3a** ca. 50:50; ***exo-cis*-3a** ca. 63:37), which is consistent with the literature *N*-protected analogues.[16]

1-((9*H*-fluoren-9-yl)methyl) 2-ethyl (2*S*,3a*S*,8a*R*)-3a-((trifluoromethyl)thio)-3,3a,8,8a-tetrahydropyrrolo[2,3-*b*]indole-1,2(2*H*)-dicarboxylate (*endo*-*cis*-3a)

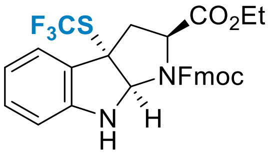

Yield: 59 mg (0.11 mmol, 21%; mixture of rotamers A/B: 50:50; R_f = 0.14 in DCM) of transparent semi-solid.

^{1}H NMR (400 MHz, $CDCl_3$): δ = 7.89–7.82 (m, 2H, Ar–H of Fmoc), 7.74 (dd, J = 7.6, 2.8 Hz, 2H, Ar–H of Fmoc), 7.65 (d, J = 7.4 Hz, 1H, Ar–H of Fmoc), 7.63–7.58 (m, 1H, Ar–H of Fmoc), 7.52–7.35 (m, 8H, Ar–H of Fmoc), 7.31–7.24 (m, 2H, Ar–H of Fmoc), 7.17–7.07 (m, 4H, CH-4 and CH-6), 6.77–6.72 (m, 1H, CH-5), 6.69 (dd, J = 7.5, 0.9 Hz, 1H, CH-5), 6.65 (d, J = 7.7 Hz, 1H, CH-7), 6.43 (d, J = 7.9 Hz, 1H, CH-7), 5.66 (s, 1H, CH-8a), 5.44 (s, 1H, NH-8), 5.14 (s, 1H, CH-8a), 4.82 (dd, J = 10.7, 4.9 Hz, 1H, CH_2 of Fmoc), 4.63 (dd, J = 10.7, 5.0 Hz, 1H, CH_2 of Fmoc), 4.56 (d, J = 8.9 Hz, 1H, CH-2), 4.49 (d, J = 9.0 Hz, 1H, CH-2), 4.48–4.40 (m, 2H, CH_2 of Fmoc), 4.35 (t, J = 5.0 Hz, 1H, CH of Fmoc), 4.19–4.15 (m, 2H, NH-8 and CH of Fmoc), 3.72–3.61 (m, 2H, CH_2 of CO_2Et), 3.57–3.45 (m, 2H, CH_2 of CO_2Et), 3.11–2.94 (m, 3H, CH_2-3), 2.80 (dd, J = 13.2, 9.1 Hz, 1H, CH_2-3), 0.89 (t, J = 7.1 Hz, 6H, CH_3 of CO_2Et).

^{13}C{^{1}H} NMR (101 MHz, $CDCl_3$): δ = 169.9 (C=O of CO_2Et), 169.7 (C=O of CO_2Et), 154.5 (C=O of Fmoc), 153.5 (C=O of Fmoc), 149.8 (C-7a), 149.3 (C-7a), 143.9 (Fmoc), 143.8 (Fmoc), 143.7 (Fmoc), 143.6 (Fmoc), 141.7 (Fmoc), 141.4 (Fmoc), 141.4 (Fmoc), 141.3 (Fmoc), 131.2 (CH-6), 131.1(CH-6), 129.6 (q, J = 310.0 Hz, CF_3S), 128.1 (Fmoc), 128.0 (Fmoc), 127.9 (Fmoc), 127.8 (Fmoc), 127.5 (Fmoc), 127.4 (Fmoc), 127.2 (Fmoc), 127.1 (Fmoc), 124.94 (CH-4), 124.86 (CH-4), 124.8 (Ar–H of Fmoc), 124.7 (C-3b), 124.6 (Ar–H of Fmoc), 124.3 (C-3b), 120.4 (Fmoc), 120.3 (Fmoc), 120.1 (Fmoc), 119.6 (CH-5), 119.4 (CH-5), 110.3 (CH-7), 109.8 (CH-7), 81.8 (q, J = 1.9 Hz, CH-8a), 81.2 (q, J = 1.8 Hz, CH-8a), 67.9 (CH_2 of Fmoc), 67.0 (CH_2 of Fmoc), 62.2 (C-3a), 61.7 (CH_2 of CO_2Et), 61.51 (CH_2 of CO_2Et), 61.48 (C-3a), 58.9 (CH-2), 58.8 (CH-2), 47.3 (CH of Fmoc), 47.2 (CH of Fmoc), 40.5 (q, J = 2.0 Hz, CH_2-3), 39.8 (q, J = 2.2 Hz, CH_2-3), 13.8 (CH_3 of CO_2Et), 13.8 (CH_3 of CO_2Et).

One ^{13}C quartet resonance for CF_3S was not reported due to an overlap of signals.

^{19}F NMR (376 MHz, $CDCl_3$): δ = –37.09, –37.28.

IR (neat, cm^{-1}): 3413, 3340, 3057, 2976, 2927, 1705, 1416, 1313, 1226, 1206, 1103, 1080, 1032, 738.

HRMS (ESI) m/z: $[M + H]^+$ Calcd for $C_{29}H_{26}F_3N_2O_4S$ 555.1560; Found: 555.1567.

1-((9*H*-fluoren-9-yl)methyl) 2-ethyl (2*S*,3a*R*,8a*S*)-3a-((trifluoromethyl)thio)-3,3a,8,8a-tetrahydropyrrolo[2,3-*b*]indole-1,2(2*H*)-dicarboxylate (*exo-cis*-3a)

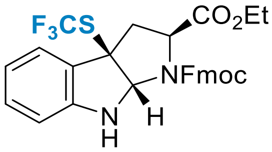

Yield: 80 mg (0.14 mmol, 29%; mixture of rotamers: 63:37; R_f = 0.26 in DCM) of yellowish semi-solid.

^{1}H NMR (400 MHz, $CDCl_3$) of the major rotamer: δ = 7.87 (d, J = 7.5 Hz, 1H, Ar–H of Fmoc), 7.84 (d, J = 6.9 Hz, 1H, Ar–H of Fmoc), 7.63 (d, J = 3.8 Hz, 1H, Ar–H of Fmoc), 7.61 (d, J = 4.0 Hz, 1H, Ar–H of Fmoc), 7.55–7.35 (m, 4H, Ar–H of Fmoc), 7.28 (d, J = 7.1 Hz, 1H, CH-4), 7.13 (d, J = 7.5 Hz, 1H, CH-4), 7.11 (dd, J = 7.7, 1.2 Hz, 1H, CH-6), 6.75 (dd, J = 7.5, 1.0 Hz, 1H, CH-5), 6.28 (dd, J = 7.8, 1.0 Hz, 1H, CH-7), 5.11 (d, J = 1.5 Hz, 1H, CH-8a), 4.84 (dd, J = 10.7, 4.4 Hz, 1H, CH_2 of Fmoc), 4.72 (dd, J = 10.7, 3.8 Hz, 1H, CH_2 of Fmoc), 4.31 (t, J = 4.2 Hz, 1H, CH of Fmoc), 4.25–4.08 (m, 2H, CH_2 of CO_2Et), 3.97 (dd, J = 8.7, 6.9 Hz, 1H, CH-2), 3.83 (d, J = 1.6 Hz, 1H, NH-8), 2.83 (dd, J = 13.1, 7.0 Hz, 1H, CH-3), 2.53 (dd, J = 13.1, 8.7 Hz, 1H, CH-3), 1.25 (t, J = 7.2 Hz, 3H, CH_3 of CO_2Et).

^{1}H NMR (400 MHz, $CDCl_3$) of the minor rotamer: δ = 7.75 (d, J = 4.0 Hz, 1H, Ar–H of Fmoc), 7.73 (d, J = 4.0 Hz, 1H, Ar–H of Fmoc), 7.55–7.35 (m, 4H, Ar–H of Fmoc), 7.31–7.24 (m, 2H, Ar–H of Fmoc), 7.21 (dd, J = 7.7, 1.2 Hz, 1H, CH-6), 6.87 (dd, J = 7.5, 1.0 Hz, 1H, CH-5), 6.70 (d, J = 7.9 Hz, 1H, CH-7), 5.77 (d, J = 1.0 Hz, 1H, CH-8a), 5.62 (s, 1H, NH-8), 4.44 (dd, J = 10.6, 6.3 Hz, 1H, CH_2 of Fmoc), 4.39–4.31 (m, 1H, CH_2 of Fmoc), 4.25–4.08 (m, 4H, CH_2 of CO_2Et, CH-2, and CH of Fmoc), 3.10 (dd, J = 13.6, 7.9 Hz, 1H, CH-3), 2.75 (dd, J = 13.6, 7.6 Hz, 1H, CH-3), 1.22 (t, J = 7.1 Hz, 3H, CH_3 of CO_2Et).

^{13}C{^{1}H} NMR (101 MHz, $CDCl_3$) of the major rotamer: δ = 170.7 (C=O of CO_2Et), 153.7 (C=O of Fmoc), 147.8 (C-7a), 143.8 (Fmoc), 143.7 (Fmoc), 141.8 (Fmoc), 141.2 (Fmoc), 130.7 (C-6), 129.4 (q, J = 310.0 Hz, CF_3S), 128.2 (Fmoc), 128.1 (Fmoc), 127.7 (Fmoc), 127.5 (Fmoc), 125.5 (C-3b), 124.6 (Fmoc), 124.50 (Fmoc), 124.3 (C-4), 120.5 (Fmoc), 120.3 (Fmoc), 119.7 (C-5), 109.9 (C-7), 81.5 (q, J = 1.7 Hz, C-8a), 66.5 (CH_2 of Fmoc), 61.87 (C-3a), 61.8 (CH_2 of CO_2Et), 58.9 (C-2), 47.2 (CH of Fmoc), 40.6 (q, J = 1.9 Hz, C-3), 14.1 (CH_3 of CO_2Et).

^{13}C{^{1}H} NMR (101 MHz, $CDCl_3$) of the minor rotamer: δ = 171.2 (C=O of CO_2Et), 154.4 (C=O of Fmoc), 148.2 (C-7a), 143.9 (Fmoc), 143.2 (Fmoc), 141.4 (Fmoc), 141.3 (Fmoc), 130.9 (C-6), 128.0 (Fmoc), 127.9 (Fmoc), 127.2 (Fmoc), 127.2 (Fmoc), 126.2 (C-3b), 125.1 (Fmoc), 124.9 (Fmoc), 124.46 (C-4), 120.1 (Fmoc), 120.1 (Fmoc), 120.1 (C-5), 110.8 (C-7), 82.5 (q, J = 1.8 Hz, C-8a), 68.0 (CH_2 of Fmoc), 61.91 (CH_2 of CO_2Et), 61.3 (C-3a), 58.6 (C-2), 47.1 (CH of Fmoc), 41.1 (q, J = 1.9 Hz, C-3), 14.2 (CH_3 of CO^2Et). ^{13}C quartet resonance for CF_3S was not reported due to an overlap of signals.

^{19}F NMR (376 MHz, $CDCl_3$) or the major rotamer: δ = –37.30.

^{19}F NMR (376 MHz, $CDCl_3$) or the minor rotamer: δ = –37.05.

IR (neat, cm^{-1}): 3413, 3053, 2984, 1745, 1706, 1419, 1315, 1299, 1257, 1192, 1104, 1032, 737.

HRMS (ESI) m/z: $[M + H]^+$ Calcd for $C_{29}H_{26}F_3N_2O_4S$ 555.1560: Found: 555.1577.

3.3. Trifluoromethylthiolation of tryptophan and derivatives 2a-f

Trifluoromethylthiolation of Fmoc-protected Trp substrates optimization procedure

A 5 mL reaction tube equipped with a magnetic stir bar, was charged with Fmoc-Trp-OR **2a,b** (0.10 mmol, 1.0 equiv), SCF_3 reagent **1** (0.12 mmol, 1.2 equiv), and the specified solvent (DCM or DCE, 1 mL, 0.1 M). After stirring the reaction mixture at room temperature for 5 min, the selected Brønsted or Lewis acid was added (0.10–0.50 mmol, 1.0–5.0 equiv; in two portions if indicated). The resulting mixture was stirred at the specified temperature (rt or 50 °C) for 6–48 h. Afterwards, an aliquot of the reaction mixture (20 µL) was used to determine the **3** *vs.* **4** ratio by ^{19}F NMR (DMSO-d_6) analysis. The reaction mixture was then diluted with 10 mL of DCM and washed with 1 M HCl (aq.), brine, and sat. $NaHCO_3$ (aq.) in the case of ethyl ester analogue **4a**. The organic phase was dried over anhydrous Na_2SO_4, filtered, concentrated *in vacuo*, and purified by silica gel CC. Isolated yield of pure (CF_3S)-pyrroloindole **3**, Fmoc-(CF_3S)Trp-OR **4** or an unseparable mixture thereof was then noted.

Fmoc-L-(CF_3S)Trp-OEt (4a)

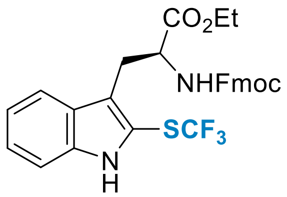

Prepared following the optimization procedure from Fmoc-L-tryptophan ethyl ester **2a** (45.5 mg, 0.10 mmol, 1.0 equiv), SCF_3 reagent **1** (0.12 mmol, 27.3 mg, 1.2 equiv), TfOH (22 µL, 0.25 mmol, 2.5 equiv), DCE (1 mL, 0.1 M), 50 °C, 6 h. Isolation: diluted with 10 mL of DCM and washed with 1 M HCl (aq.) (2 x 5 mL), brine (1 x 5 mL), and sat. $NaHCO_3$ (aq.) (1 x 5 mL). Purification: silica gel CC (dry loading; mobile phase: EtOAc/cy-hexane 1:3) to afford the trifluoromethylthiolated product **4a**.

Yield: 50 mg (0.090 mmol, 90%; mixture of rotamers: 87:13) of white solid.

mp 171–174 °C

$[\alpha]_D^{20}$ = –8.4 (*c* 0.16, MeOH)

^{1}H NMR (500 MHz, $CDCl_3$) of the major rotamer: δ = 8.48 (s, 1H), 7.77 (d, *J* = 7.6 Hz, 2H), 7.68 (d, *J* = 8.1 Hz, 1H), 7.56 (d, *J* = 7.5 Hz, 1H), 7.53 (d, *J* = 7.5 Hz, 1H), 7.43–7.38 (m, 2H), 7.35 (d, *J* = 8.3 Hz, 1H), 7.33–7.28 (m, 3H), 7.16 (ddd, *J* = 8.1, 6.9, 1.1 Hz, 1H), 5.40 (d, *J* = 8.4 Hz, 1H), 4.76 (dt, *J* = 8.4, 6.3 Hz, 1H), 4.37–4.33 (m, 2H), 4.22–4.15 (m, 2H), 4.14–4.05 (m, 1H), 3.49 (dd, *J* = 14.5, 6.3 Hz, 1H), 3.42 (dd, *J* = 14.5, 6.4 Hz, 1H), 1.20 (t, *J* = 7.1 Hz, 3H).

^{13}C{^{1}H} NMR (126 MHz, $CDCl_3$): δ = 171.8, 155.7, 144.0, 143.9, 141.4, 137.6, 128.5 (q, *J* = 311.9 Hz), 127.8, 127.5, 127.2, 125.3, 125.2, 121.5, 120.9, 120.1, 120.0, 115.0, 111.5, 67.2, 61.9, 54.4, 47.2, 28.1, 14.1.

^{19}F NMR (471 MHz, $CDCl_3$): δ = –42.49.

IR (neat, cm^{-1}): 3344, 3324, 3060, 2991, 2944, 1737, 1685, 1538, 1278, 1227, 1193, 1153, 1117, 1087, 1041, 867, 780, 758, 735, 667, 620.

HRMS (ESI) m/z: $[M + H]^+$ Calcd for $C_{29}H_{26}F_3N_2O_4S$ 555.1560; Found: 555.1558.

Fmoc-L-(CF_3S)Trp-OH ((*S*)-4b)

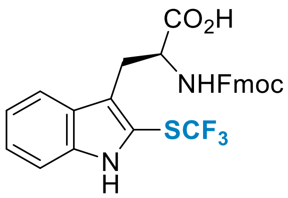

Prepared following the optimization procedure from Fmoc-L-tryptophan **2b** (42.6 mg, 0.10 mmol, 1.0 equiv), SCF_3 reagent **1** (27.3 mg, 0.12 mmol, 1.2 equiv), $BF_3{\cdot}OEt_2$ (31 µL, 0.25 mmol, 2.5 equiv), DCE (1 mL, 0.1 M), 50 °C, 24 h. Isolation: diluted with 15 mL of DCM and washed with 1 M HCl (aq.) (1 x 10 mL), and brine (1 x 10 mL). Purification: silica gel CC (dry loading; mobile phase: 1. DCM (elution of non-polar impurities) → 2. DCM/MeOH 10:1) to afford the trifluoromethylthiolated product **(*S*)-4b**.

Yield: 44 mg (0.084 mmol, 84%; mixture of rotamers: 87:13) of yellowish solid.

mp 123–126 °C

$[\alpha]_D^{20}$ = –8.7 (*c* 0.195, MeOH)

^{1}H NMR (500 MHz, DMSO-*d*$_6$) of the major rotamer: δ = 11.81 (s, 1H), 7.87 (d, *J* = 7.6 Hz, 2H), 7.79 (d, *J* = 8.1 Hz, 1H), 7.66–7.61 (m, 2H), 7.43–7.35 (m, 3H), 7.32–7.20 (m, 3H), 7.07–7.01 (m, 1H), 4.21 (td, *J* = 8.5, 5.5 Hz, 1H), 4.17–4.09 (m, 3H), 3.40 (dd, *J* = 14.2, 5.4 Hz, 1H), 3.20 (dd, *J* = 14.2, 8.7 Hz, 1H).

^{13}C{^{1}H} NMR (126 MHz, DMSO-*d*$_6$): δ = 173.4, 155.8, 143.8, 143.7, 140.7, 137.6, 128.7 (q, *J* = 311.6 Hz), 127.6, 127.1, 126.9, 125.3, 124.0, 122.3, 120.2, 120.1, 119.5, 113.3, 111.6, 65.7, 55.4, 46.6, 27.0.

^{19}F NMR (471 MHz, DMSO-*d*$_6$): δ = –42.18.

IR (neat, cm^{-1}): 3403, 3359, 3333, 3310, 3063, 3043, 2928, 2859, 1707, 1516, 1449, 1415, 1342, 1214, 1132, 1109, 1090, 1057, 740.

HRMS (ESI) m/z: $[M + H]^+$ Calcd for $C_{27}H_{22}F_3N_2O_4S$ 527.1247; Found: 527.1247.

HPLC analysis: t_R = 9.96 min, 98% *ee* (Chiralpak IA-3 column; flow rate of 1.0 mL/min; mobile phase: 15% *i*PrOH + 0.1% TFA in *n*-hexane; detection at 254 nm).

Gram-scale synthesis of Fmoc-L-(CF_3S)Trp-OH 4b

A 100 mL round bottom flask equipped with a magnetic stir bar, was charged with Fmoc-L-tryptophan **2b** (1.71 g, 4.0 mmol, 1.0 equiv), SCF_3 reagent **1** (1.10 g, 4.8 mmol, 1.2 equiv), and 20 mL of DCE (0.2 M). The reaction mixture was cooled in an ice bath and stirred for 5 min before the dropwise addition of $BF_3{\cdot}OEt_2$ (48% solution; 2.5 mL, 10 mmol, 2.5 equiv). The resulting mixture was warmed with a heating block to 50 °C in stirred for 24 h. 50 mL of DCM was added to the reaction mixture, followed by two consecutive washes with 1 M HCl (aq.) and brine. The organic phase was dried over anhydrous Na_2SO_4, filtered, and concentrated *in vacuo*. The crude product was purified by silica gel CC (dry loading, mobile phase: DCM →

DCM : MeOH 20:1) to obtain the Fmoc-(CF_3S)Trp-OH **4b** as a pure yellow solid in 93% yield (1.95 g).

Fmoc-D-(CF_3S)Trp-OH ((*R*)-4b)

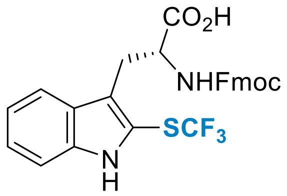

Prepared same as **4b** from Fmoc-D-tryptophan **2b** (42.5 mg, 0.10 mmol, 1.0 equiv), SCF_3 reagent **1** (26.9 mg, 0.12 mmol, 1.2 equiv), BF_3·OEt_2 (31 µL, 0.25 mmol, 2.5 equiv), DCE (1 mL, 0.1 M), 50 °C, 24 h. Purification: silica gel CC (DCM/MeOH 10:1) to afford the trifluoromethylthiolated product **(*R*)-4b**. The spectroscopic data of **(*R*)-4b** is in agreement with **(*S*)-4b**.

Yield: 41 mg (0.079 mmol, 79%; mixture of rotamers: 86:14) of white-yellowish solid.

^{1}H NMR (400 MHz, DMSO-*d*$_6$) of the major rotamer: δ = 11.74 (s, 1H), 7.88 (d, *J* = 7.5 Hz, 2H), 7.82 (d, *J* = 8.2 Hz, 1H), 7.64–7.60 (m, 2H), 7.44–7.37 (m, 2H), 7.36 (d, *J* = 8.3 Hz, 1H), 7.33–7.25 (m, 2H), 7.20 (ddd, *J* = 8.3, 6.9, 1.1 Hz, 1H), 7.05–6.99 (m, 1H), 4.24–3.97 (m, 4H), 3.40 (dd, *J* = 14.1, 5.0 Hz, 1H), 3.17 (dd, *J* = 14.0, 8.4 Hz, 1H).

^{19}F NMR (471 MHz, DMSO-*d*$_6$): δ = –42.23.

HRMS (ESI) m/z: $[M + H]^+$ Calcd for $C_{27}H_{22}F_3N_2O_4S$ 527.1247; Found: 527.1247.

HPLC analysis: t_R = 8.29 min, >99% *ee* (Chiralpak IA-3 column; flow rate of 1.0 mL/min; mobile phase: 15% *i*PrOH + 0.1% TFA in *n*-hexane; detection at 254 nm).

H_2N-(CF_3S)Trp-OEt (4c)

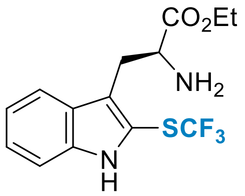

Prepared following the general trifluoromethylthiolation procedure from L-tryptophan ethyl ester hydrochloride **2c** (26.7 mg, 0.10 mmol, 1.0 equiv), SCF_3 reagent **1** (24.8 mg, 0.11 mmol, 1.1 equiv), TfOH (22 µL, 0.25 mmol, 2.5 equiv), DCM (1 mL, 0.1 M), rt, 30 min. Isolation: diluted with 10 mL of DCM and sat. $NaHCO_3$ (aq), then extracted with DCM two more times (2 x 10 mL). Purification: silica gel CC (mobile phase: 1. DCM (elution of non-polar impurities) → 2. DCM/MeOH 10:1) to afford the trifluoromethylthiolated product **4c**.

Yield: 31.9 mg (0.096 mmol, 96%) of yellow solid.

mp 109–111 °C

$[\alpha]_D^{20}$ = +27.0 (*c* 0.35, MeOH)

^{1}H NMR (500 MHz, $CDCl_3$): δ = 8.41 (s, 1H), 7.68 (dd, *J* = 8.1, 1.0 Hz, 1H), 7.36 (d, *J* = 8.3 Hz, 1H), 7.30 (ddd, *J* = 8.3, 6.9, 1.1 Hz, 1H), 7.17 (ddd, *J* = 8.1, 6.9, 1.1 Hz, 1H), 4.19–4.10

(m, 2H), 3.84 (dd, *J* = 8.5, 5.4 Hz, 1H), 3.40 (dd, *J* = 14.2, 5.3 Hz, 1H), 3.17 (dd, *J* = 14.2, 8.5 Hz, 1H), 1.63 (bs, 2H), 1.20 (t, *J* = 7.1 Hz, 3H).

^{13}C{^{1}H} NMR (126 MHz, $CDCl_3$): δ = 175.2, 137.7, 128.6 (q, *J* = 311.9 Hz), 127.3, 124.9, 122.5, 120.5, 120.0, 114.9 (q, *J* = 2.3 Hz), 111.5, 61.3, 55.3, 30.6, 14.2.

^{19}F NMR (471 MHz, $CDCl_3$): δ = –42.55.

IR (neat, cm^{-1}): 3364, 3304, 2985, 2852, 1715, 1443, 1382, 1344, 1298, 1233, 1206, 1130, 1107, 1031, 948, 892, 855, 809, 745.

HRMS (ESI) m/z: $[M + H]^+$ Calcd for $C_{14}H_{16}F_3N_2O_2S$ 333.0879; Found: 333.0877.

N-Fmoc-(2-(trifluoromethyl)thio)-tryptamine (4d)

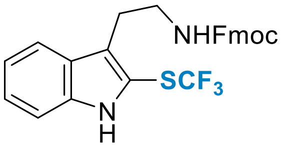

Prepared following the general trifluoromethylthiolation procedure from *N*-Fmoc-tryptamine **2d** (38.2 mg, 0.10 mmol, 1.0 equiv), SCF_3 reagent **1** (27.2 mg, 0.12 mmol, 1.2 equiv), TfOH (22 µL, 0.25 mmol, 2.5 equiv), DCM (1 mL, 0.1 M), rt, 16 h. Isolation: diluted with 15 mL of DCM and washed with 10% HCl (aq.) (1 x 10 mL), and brine (1 x 10 mL). Purification: silica gel CC (mobile phase: EtOAc/*n*-hex 1:3) to afford the trifluoromethylthiolated product **4d**.

Yield: 43.6 mg (0.090 mmol, 90%; mixture of rotamers: 84:16) of yellowish white solid.

mp 149–152 °C

^{1}H NMR (500 MHz, $CDCl_3$) of the major rotamer: δ = 8.32 (s, 1H), 7.77 (d, *J* = 7.5 Hz, 2H), 7.71 (d, *J* = 8.1 Hz, 1H), 7.57 (d, *J* = 7.5 Hz, 2H), 7.42–7.38 (m, 3H), 7.34–7.28 (m, 3H), 7.19–7.15 (m, 1H), 4.87 (t, *J* = 6.3 Hz, 1H), 4.41 (d, *J* = 7.0 Hz, 2H), 4.21 (t, *J* = 7.0 Hz, 1H), 3.57–3.49 (m, 2H), 3.16 (t, *J* = 7.0 Hz, 2H).

^{13}C{^{1}H} NMR (126 MHz, $CDCl_3$): δ = 156.5, 144.1, 141.4, 137.7, 128.5 (q, *J* = 311.7 Hz), 127.8, 127.4, 127.2, 125.2, 125.2, 124.3, 120.7, 120.2, 120.1, 114.0, 111.5, 66.8, 47.4, 41.5, 25.6.

^{19}F NMR (471 MHz, $CDCl_3$): δ = –42.66.

IR (neat, cm^{-1}): 3401, 3317, 3066, 2930, 1688, 1670, 1539, 1448, 1340, 1255, 1131, 1107, 1016, 756, 735, 672, 621.

HRMS (ESI) m/z: $[M + H]^+$ Calcd for $C_{26}H_{22}F_3N_2O_2S$ 483.1349; Found: 483.1344.

N-methyl-(2-(trifluoromethyl)thio)-tryptamine (4e)

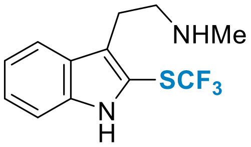

Prepared following the general trifluoromethylthiolation procedure from *N*-methyltryptamine **2e** (17.4 mg, 0.10 mmol, 1.0 equiv), SCF_3 reagent **1** (27.3 mg, 0.12 mmol, 1.2 equiv), TfOH (22 µL, 0.25 mmol, 2.5 equiv), DCM (1 mL, 0.1 M), rt, 16 h. Isolation: diluted with 10 mL of DCM and 3 ml of 0.2 M $NaHCO_3$ (aq.), then extracted with DCM two more times (3 x 10 mL).

Purification: silica gel CC (mobile phase: 1. DCM (elution of non-polar impurities) → 2. DCM/MeOH 10:1) to afford the trifluoromethylthiolated product **4e**.

Yield: 21.2 mg (0.077 mmol, 77%) of white solid.

mp 137–140 °C

^{1}H NMR (500 MHz, CDCl$_3$): δ = 8.70 (s, 1H), 7.68 (d, J = 8.1 Hz, 1H), 7.35 (d, J = 8.3 Hz, 1H), 7.29 (ddd, J = 8.2, 6.9, 1.1 Hz, 1H), 7.15 (ddd, J = 8.1, 7.0, 1.1 Hz, 1H), 3.15 (t, J = 7.3 Hz, 2H), 2.94 (t, J = 7.3 Hz, 2H), 2.49 (s, 3H), 1.61 (bs, 1H).

^{13}C{^{1}H} NMR (126 MHz, CDCl$_3$): δ = 137.7, 128.6 (q, J = 311.8 Hz), 127.4, 125.3, 124.9, 120.3, 120.1, 113.8 (q, J = 2.3 Hz), 111.5, 52.3, 36.4, 25.3.

^{19}F NMR (471 MHz, CDCl$_3$): δ = –42.75.

IR (neat, cm^{-1}): 1463, 1417, 1360, 1341, 1223, 1164, 1124, 1101, 1082, 1040, 1007, 99, 874, 823, 807, 776, 742, 664.

HRMS (ESI) m/z: [M + H]$^+$ Calcd for $C_{12}H_{14}F_3N_2S$ 275.0824; Found: 275.0826.

(2-(trifluoromethyl)thio)-tryptamine (4f)

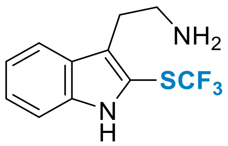

Prepared following the general trifluoromethylthiolation procedure from tryptamine **2f** (16.0 mg, 0.10 mmol, 1.0 equiv), SCF_3 reagent **1** (27.5 mg, 0.12 mmol, 1.2 equiv), TfOH (22 µL, 0.25 mmol, 2.5 equiv), DCM (1 mL, 0.1 M), rt, 16 h. Isolation: diluted with 10 mL of DCM and 3 ml of 0.2 M $NaHCO_3$ (aq.), then extracted with DCM two more times (3 x 10 mL). Purification: silica gel CC (mobile phase: 1. DCM (elution of non-polar impurities) → 2. DCM/MeOH 10:1) to afford the trifluoromethylthiolated product **4f**.

Yield: 23.9 mg (0.092 mmol, 92%) of white solid.

mp 112–115 °C

^{1}H NMR (500 MHz, CDCl$_3$): δ = 8.58 (s, 1H), 7.67 (d, J = 8.1 Hz, 1H), 7.37 (d, J = 8.3 Hz, 1H), 7.31 (ddd, J = 8.2, 6.9, 1.1 Hz, 1H), 7.16 (ddd, J = 8.0, 6.8, 1.0 Hz, 1H), 3.11–3.03 (m, 4H), 1.36 (bs, 2H).

^{13}C{^{1}H} NMR (126 MHz, CDCl$_3$): δ = 137.7, 128.6 (q, J = 311.8 Hz), 127.4, 125.1, 124.9, 120.4, 120.2, 114.0, 111.5, 42.8, 29.4.

^{19}F NMR (471 MHz, CDCl$_3$): δ = –42.73.

IR (neat, cm^{-1}): 2837, 1573, 1441, 1341, 1228, 1105, 1016, 941, 746, 668.

HRMS (ESI) m/z: [M + H]$^+$ Calcd for $C_{11}H_{12}F_3N_2S$ 261.0668; Found: 261.0667.

3.4. Trifluoromethylthiolation of C5-substituted tryptamines 2g-i

Table S1. Trifluoromethylthiolation of C5-substituted tryptamines **2g-i**.

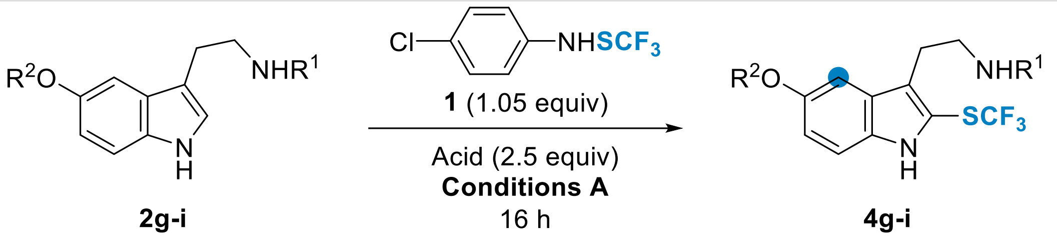

Entry	R^1	R^2	Substrate	Acid	*mono vs. bis*[a]
1	H·HCl	H	**5-HT**	TfOH	1 : 2.4
2	H·HCl	H	**5-HT**	BF_3·OEt_2	3.8 : 1
3	Fmoc	H	**2g**	TfOH	3.2 : 1
4[b,c]	Fmoc	H	**2g**	BF_3·OEt_2	2.1 : 1
5	Fmoc	Bn	**2h**	BF_3·OEt_2	*mono*[d]
6[c,e]	Fmoc	Bn	**2h**	BF_3·OEt_2	*mono* (82%)
7	Ac	Me	**2i**	TfOH	11 : 1
8	Ac	Me	**2i**	BF_3·OEt_2	*mono*[f]
9[c]	Ac	Me	**2i**	BF_3·OEt_2	*mono* (66%)

C5-substituted tryptamine (0.10 mmol, 1.0 eq.), $ArNHSCF_3$ **1** (1.05 equiv), acid (2.5 equiv), **Conditions A** (DCM; 1 mL, 0.1 M; rt). [a]Ratios determined by ^{19}F NMR analysis (DMSO-d_6) of the crude reaction mixture, isolated yield in parantheses. [b]48 h. [c]BF_3·OEt_2 (5.0 equiv), 48 h. [d]*mono*-SCF_3-pyrroloindole **3h** as the major product. [e]**Conditions B** (DCE; 1 mL, 0.1 M; 50 °C), 24 h. [f]Reaction not complete.

Optimization procedure

A 5 mL reaction tube equipped with a magnetic stir bar, was charged with the corresponding C5-substituted tryptamine **2g-i** or serotonin hydrochloride **5-HT** (0.10 mmol, 1.0 equiv), SCF_3 reagent **1** (0.105 mmol, 1.05 equiv), and the solvent (DCM or DCE, 1 mL, 0.1 M). After stirring the reaction mixture for 5 min at room temperature, the selected Brønsted or Lewis acid was added (0.25–0.50 mmol, 2.5–5.0 equiv). The resulting mixture was stirred at the specified temperature (rt or 50 °C) for 24 h. Afterwards, an aliquot of the reaction mixture (20 µL) was used to determine the *mono vs. bis* ratio by ^{19}F NMR (DMSO-d_6) analysis and to estimate the conversion.

***O*-Benzyl-*N*-Fmoc-(2-(trifluoromethyl)thio)-serotonin (4h)**

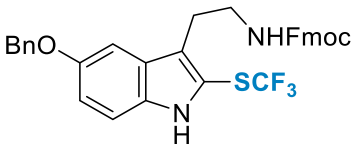

Prepared following the general trifluoromethylthiolation procedure from *O*-benzyl-*N*-Fmoc-serotonin **2h** (48.7 mg, 0.10 mmol, 1.0 equiv), SCF_3 reagent **1** (23.8 mg, 0.105 mmol, 1.05 equiv), $BF_3{\cdot}OEt_2$ (2 x 31 µL, 0.50 mmol, 5.0 equiv; in two portions), DCE (1 mL, 0.1 M), 50 °C, 24 h. Isolation: diluted with 20 mL of DCM and washed with 1 M HCl (aq.) (10 mL), brine (10 mL), and sat. $NaHCO_3$ (aq.) (10 mL). Purification: silica gel CC (mobile phase: DCM) to afford the *mono*-trifluoromethylthiolated serotonin derivative **4h**.

Yield: 48 mg (0.082 mmol, 82%; mixture of rotamers: 83:17) of white solid.

mp 136–138 °C

^{1}H NMR (400 MHz, $CDCl_3$) of the major rotamer: δ = 8.34 (s, 1H), 7.74 (d, *J* = 7.5 Hz, 2H), 7.54 (d, *J* = 7.5 Hz, 2H), 7.44–7.25 (m, 10H), 7.19 (d, *J* = 2.4 Hz, 1H), 7.04 (dd, *J* = 8.9, 2.4 Hz, 1H), 5.06 (s, 2H), 4.88 (t, *J* = 6.1 Hz, 1H), 4.40 (d, *J* = 7.0 Hz, 2H), 4.19 (t, *J* = 7.0 Hz, 1H), 3.53–3.45 (m, 2H), 3.10 (t, *J* = 7.1 Hz, 2H).

^{13}C{^{1}H} NMR (101 MHz, $CDCl_3$): δ = 156.5, 153.7, 144.0, 141.4, 137.2, 133.1, 128.6, 128.5 (q, *J* = 311.8 Hz), 128.0, 127.8, 127.8, 127.6, 127.1, 125.2, 123.6, 120.1, 116.6, 114.2, 112.5, 102.3, 70.8, 66.8, 47.3, 41.2, 25.5.

^{19}F NMR (376 MHz, $CDCl_3$): δ = –42.89.

IR (neat, cm^{-1}): 3437, 3308, 3065, 3033, 2915, 2850, 1690, 1532, 1446, 1225, 1133, 1112, 1014, 755, 733, 727, 691.

HRMS (ESI) m/z: $[M + H]^+$ Calcd for $C_{33}H_{28}F_3N_2O_3S$ 589.1767; Found: 589.1767.

2-(trifluoromethyl)thio)-melatonin (4i)

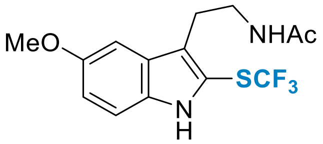

Prepared following the general trifluoromethylthiolation procedure from melatonin **2i** (23.1 mg, 0.10 mmol, 1.0 equiv), SCF_3 reagent **1** (23.2 mg, 0.102 mmol, 1.02 equiv), $BF_3{\cdot}OEt_2$ (2 x 31 µL, 0.50 mmol, 5.0 equiv; in one portion), DCM (1 mL, 0.1 M), rt, 24 h. Isolation: diluted with 20 mL of DCM and washed with 1 M HCl (aq.) (10 mL), brine (10 mL), and sat. $NaHCO_3$ (aq.) (10 mL). Purification: silica gel CC (mobile phase: EtOAc/cy-hex 1:1) to afford the *mono*-trifluoromethylthiolated melatonin derivative **4i**.

Yield: 22 mg (0.066 mmol, 66%) of brown solid.

mp 142–144 °C

^{1}H NMR (400 MHz, $CDCl_3$): δ = 8.65 (s, 1H), 7.29 (d, *J* = 8.9 Hz, 1H), 7.09 (d, *J* = 2.4 Hz, 1H), 6.97 (dd, *J* = 8.9, 2.4 Hz, 1H), 5.64 (t, *J* = 6.2 Hz, 1H), 3.85 (s, 3H), 3.61–3.54 (m, 2H), 3.11 (t, *J* = 6.9 Hz, 2H), 1.94 (s, 3H).

$^{13}C\{^{1}H\}$ NMR (101 MHz, $CDCl_3$): δ = 170.5, 154.6, 133.0, 128.5 (q, J = 311.6 Hz), 127.6, 123.7, 116.3, 114.0 (q, J = 2.4 Hz), 112.6, 100.5, 55.9, 39.9, 25.1, 23.5.

^{19}F NMR (376 MHz, $CDCl_3$): δ = –42.92.

IR (neat, cm^{-1}): 3340, 3300, 3077, 2850, 2838, 1645, 1553, 1438, 1304, 1143, 1103, 1027, 792, 614, 595.

HRMS (ESI) m/z: $[M + H]^+$ Calcd for $C_{14}H_{16}F_3N_2O_2S$ 333.0879; Found: 333.0884.

3.5. Trifluoromethylthiolation of tyrosine derivatives 5a-i

Trifluoromethylthiolation of Fmoc-protected Tyr substrates optimization procedure

A 10 mL round bottom flask equipped with a magnetic stir bar, was charged with Fmoc-Tyr-OR **5a,b** (0.10 mmol, 1.0 eq.), SCF_3 reagent **1** (0.12–0.25 mmol, 1.2–2.5 eq.), and the specified solvent (DCM or DCE, 1 mL, 0.1 M). After stirring the reaction mixture at room temperature for 5 min, the selected Brønsted or Lewis acid was added (0.25–0.50 mmol, 2.5–5.0 equiv; in two portions if indicated). The resulting mixture was stirred at the specified temperature (rt or 50 °C) for 24 h. Afterwards, an aliquot of the reaction mixture (20 µL) was used to determine the conversion to **6a,b** by ^{1}H NMR (DMSO-d_6) analysis.

Fmoc-L-(CF_3S)Tyr-OH ((*S*)-6a)

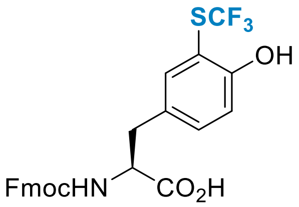

Prepared following the general trifluoromethylthiolation procedure from Fmoc-L-tyrosine **5a** (40.2 mg, 0.10 mmol, 1.0 equiv), SCF_3 reagent **1** (34.0 mg, 0.15 mmol, 1.5 equiv), TfOH (22 µL, 0.25 mmol, 2.5 equiv), DCM (1 mL, 0.1 M), rt, 24 h. Isolation: diluted with 15 mL of DCM and washed with 1 M HCl (aq.) (2 x 10 mL), and brine (1 x 10 mL). Purification: silica gel CC (mobile phase: DCM/MeOH 10:1 → 5:1) to afford the trifluoromethylthiolated product **(*S*)-6a**.

Yield: 40 mg (0.079 mmol, 79%; mixture of rotamers: 87:13) of white solid.

mp 128–131 °C

$[\alpha]_D^{20}$ = +18.0 (*c* 0.19, MeOH)

^{1}H NMR (500 MHz, DMSO-d_6) of the major rotamer: δ = 10.72 (bs, 1H), 7.88 (d, J = 7.6 Hz, 2H), 7.67–7.58 (m, 2H), 7.42–7.38 (m, 2H), 7.35 (d, J = 2.2 Hz, 1H), 7.33–7.26 (m, 2H), 7.20 (dd, J = 8.5, 2.2 Hz, 1H), 6.92 (d, J = 8.4 Hz, 1H), 6.90–6.83 (m, 1H), 4.30–4.21 (m, 1H), 4.19–4.09 (m, 2H), 4.02–3.88 (m, 1H), 3.04 (dd, J = 14.0, 4.4 Hz, 1H), 2.81 (dd, J = 13.7, 8.3 Hz, 1H).

^{13}C{^{1}H} NMR (126 MHz, DMSO-d_6): δ = 158.1, 155.5, 143.9, 143.9, 140.7, 140.7, 138.6, 134.4, 130.4, 129.8 (q, J = 308.6 Hz), 127.6, 127.1, 125.3, 125.1, 121.4, 120.1, 116.3, 107.4, 65.4, 56.7, 46.7, 36.1.

^{19}F NMR (471 MHz, DMSO-d_6): δ = –41.47.

IR (neat, cm^{-1}): 3305, 1689, 1581, 1539, 1488, 1447, 1415, 1333, 1297, 1261, 1103, 1052, 757, 738.

HRMS (ESI) m/z: $[M + H]^+$ Calcd for $C_{25}H_{21}F_3NO_5S$ 504.1087; Found: 504.1087.

HPLC analysis: *t*R = 16.01 min, 99% *ee* (Chiralpak IA-3 column; flow rate of 1.0 mL/min; mobile phase: 10 → 20% *i*PrOH + 0.1% TFA in *n*-hexane in 20 min; detection at 254 nm).

Gram-scale synthesis of Fmoc-L-(CF_3S)Tyr-OH 6a:

A 250 mL round bottom flask equipped with a magnetic stir bar, was charged with Fmoc-L-tyrosine **5a** (1.61 g, 4.0 mmol, 1.0 eq.), SCF_3 reagent **1** (1.37 g, 6.0 mmol, 1.2 eq.), and 40 mL of DCM (0.1 M). The reaction mixture was cooled in an ice bath and stirred for 5 min before the dropwise addition of TfOH (880 µL, 10 mmol, 2.5 eq.). The resulting mixture was warmed to room temperature and stirred for 24 h. Then, 75 mL of DCM was added to the reaction mixture, followed by two consecutive washes with 1 M HCl and brine. The organic phase was dried over anhydrous Na_2SO_4, filtered, and concentrated *in vacuo*. The crude product was purified by silica gel CC (mobile phase: DCM/MeOH = 20:1 → DCM/MeOH 5:1) to obtain the trifluoromethylthiolated product **6a** as an yellowish solid in 77% yield (1.55 g).

Fmoc-D-(CF_3S)Tyr-OH ((*R*)-6a)

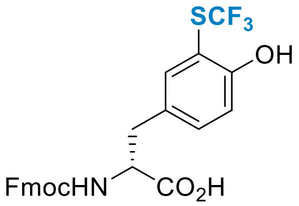

Prepared same as **6a** from Fmoc-D-tyrosine **5a** (40.2 mg, 0.10 mmol, 1.0 equiv), SCF_3 reagent **1** (34.5 mg, 0.15 mmol, 1.5 equiv), TfOH (1 x 22 µL, 0.25 mmol, 2.5 equiv), DCM (1 mL, 0.1 M), rt, 24 h. Isolation: diluted with 15 mL of DCM and washed with 1 M HCl (aq.) (1 x 10 mL), and brine (1 x 10 mL). Purification: silica gel CC (mobile phase: DCM/MeOH 10:1 → 5:1) to afford the trifluoromethylthiolated product **(*R*)-6a**. The spectroscopic data of **(*R*)-6a** in MeOD-d_4 is in agreement with **(*S*)-6a (**DMSO-d_6).

Yield: 39 mg (0.077 mmol, 77%; mixture of rotamers: 88:12) of white solid.

^{1}H NMR (400 MHz, MeOD-d_4) of the major rotamer: δ = 7.76 (d, J = 7.6 Hz, 2H), 7.56 (d, J = 7.5 Hz, 2H), 7.43 (s, 1H), 7.39–7.33 (m, 2H), 7.30–7.21 (m, 3H), 6.86 (d, J = 8.2 Hz, 1H), 4.38–4.25 (m, 2H), 4.21–4.07 (m, 2H), 3.18–3.11 (m, 1H), 2.91–2.81 (m, 1H).

^{19}F NMR (376 MHz, MeOD-d_4): δ = –44.47.

HRMS (ESI) m/z: $[M + H]^+$ Calcd for $C_{25}H_{21}F_3NO_5S$ 504.1087; Found: 504.1086.

HPLC analysis: *t*R = 14.27 min, >99% *ee* (Chiralpak IA-3 column; flow rate of 1.0 mL/min; mobile phase: 10 → 20% *i*PrOH + 0.1% TFA in *n*-hexane in 20 min; detection at 254 nm).

Fmoc-L-(CF_3S)Tyr-OMe (6b)

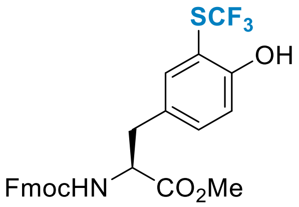

Prepared following the general trifluoromethylthiolation procedure from Fmoc-L-tyrosine methyl ester **5b** (41.7 mg, 0.10 mmol, 1.0 equiv), SCF_3 reagent **1** (56.9 mg, 0.25 mmol, 2.5 equiv), TfOH (2 x 22 µL, 0.50 mmol, 5.0 equiv; 2nd addition after 18 h), DCM (1 mL, 0.1 M), rt,

24 h. Isolation: diluted with 15 mL of DCM and washed with 10% HCl (aq.) (1 x 10 mL), brine (1 x 10 mL), and sat. $NaHCO_3$ (aq.) (1 x 10 mL). Purification: silica gel CC (dry loading; mobile phase: EtOAc/*n*-hex 1:3) to afford the trifluoromethylthiolated product **6b**.

Yield: 48 mg (0.093 mmol, 93%; mixture of rotamers: 90:10) of white solid.

mp 146–149 °C

$[\alpha]_D^{20}$ = +3.0 (*c* 0.22, MeOH)

^{1}H NMR (500 MHz, $CDCl_3$) of the major rotamer: δ = 7.78 (d, *J* = 7.6 Hz, 2H), 7.58 (dd, *J* = 7.5, 4.5 Hz, 2H), 7.43–7.39 (m, 2H), 7.34 (d, *J* = 1.2 Hz, 1H), 7.33–7.29 (m, 2H), 7.13 (dd, *J* = 8.5, 2.2 Hz, 1H), 6.98 (d, *J* = 8.4 Hz, 1H), 6.35 (s, 1H), 5.31 (d, *J* = 8.0 Hz, 1H), 4.64 (dt, *J* = 8.1, 5.6 Hz, 1H), 4.47 (dd, *J* = 10.7, 7.1 Hz, 1H), 4.39 (dd, *J* = 10.7, 6.7 Hz, 1H), 4.21 (t, *J* = 6.8 Hz, 1H), 3.73 (s, 3H), 3.09 (dd, *J* = 14.3, 6.0 Hz, 1H), 3.08–3.01 (m, 1H).

^{13}C{^{1}H} NMR (126 MHz, $CDCl_3$): δ = 171.7, 157.4, 155.7, 143.9, 143.8, 141.5, 141.4, 138.8, 135.5, 129.0, 128.8 (q, *J* = 310.6 Hz), 127.9, 127.2, 125.2, 125.1, 120.2, 120.1, 116.6, 108.5 (q, *J* = 1.7 Hz), 67.1, 54.9, 52.6, 47.3, 37.2.

^{19}F NMR (471 MHz, $CDCl_3$): δ = –42.61.

IR (neat, cm^{-1}): 3397, 3307, 3067, 2953, 1733, 1689, 1607, 1538, 1488, 1296, 1265, 1218, 1104, 1052, 757, 734.

HRMS (ESI) m/z: $[M + H]^+$ Calcd for $C_{26}H_{23}F_3NO_5S$ 518.1244; Found: 518.1243.

N-Fmoc-(3-(trifluoromethyl)thio)-tyramine (6d)

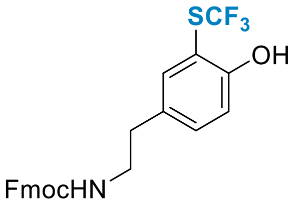

Prepared following the general trifluoromethylthiolation procedure from *N*-Fmoc-tyramine **5d** (35.9 mg, 0.10 mmol, 1.0 equiv), SCF_3 reagent **1** (56.7 mg, 0.25 mmol, 2.5 equiv), TfOH (2 x 22 µL, 0.50 mmol, 5.0 equiv; 2nd addition after 18 h), DCM (1 mL, 0.1 M), rt, 24 h. Isolation: diluted with 15 mL of DCM and washed with 10% HCl (aq.) (2 x 10 mL), and brine (1 x 10 mL). Purification: silica gel CC (dry loading; mobile phase: EtOAc/*n*-hex 1:1) to afford the trifluoromethylthiolated product **6d**.

Yield: 37 mg (0.081 mmol, 81%; mixture of rotamers: 84:16) of yellowish solid.

mp 33–36 °C

^{1}H NMR (500 MHz, $CDCl_3$) of the major rotamer: δ = 7.77 (d, *J* = 7.6 Hz, 2H), 7.57 (d, *J* = 7.5 Hz, 2H), 7.44–7.36 (m, 3H), 7.35–7.28 (m, 2H), 7.23 (dd, *J* = 8.4, 2.3 Hz, 1H), 7.01 (d, *J* = 8.4 Hz, 1H), 6.34 (s, 1H), 4.79 (t, *J* = 6.3 Hz, 1H), 4.42 (d, *J* = 6.8 Hz, 2H), 4.21 (t, *J* = 6.9 Hz, 1H), 3.46–3.36 (m, 2H), 2.76 (t, *J* = 6.9 Hz, 2H).

^{13}C{^{1}H} NMR (126 MHz, $CDCl_3$): δ = 157.0, 156.5, 144.0, 141.5, 138.2, 134.9, 132.0, 128.9 (q, *J* = 310.7 Hz), 127.8, 127.2, 125.1, 120.1, 116.6, 108.4, 66.7, 47.4, 42.3, 35.1.

^{19}F NMR (376 MHz, $CDCl_3$): δ = –42.73.

IR (neat, cm^{-1}): 3410, 3336, 2945, 1689, 1489, 1449, 1416, 1247, 1098, 1056, 825, 756, 739.

HRMS (ESI) m/z: $[M + H]^+$ Calcd for $C_{24}H_{21}F_3NO_3S$ 460.1189; Found: 460.1188.

(3-(trifluoromethyl)thio)-tyramine trifluoromethanesulfonate salt (6e)

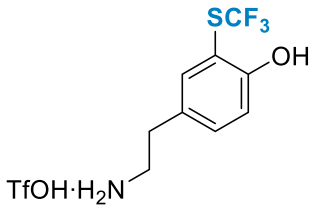

Prepared following the general trifluoromethylthiolation procedure from tyramine **5e** (27.4 mg, 0.20 mmol, 1.0 equiv), SCF_3 reagent **1** (114 mg, 0.50 mmol, 2.5 equiv), TfOH (2 x 44 µL, 0.50 mmol, 5.0 equiv; 2nd addition after 18 h), DCM (2 mL, 0.1 M), rt, 24 h. Isolation: diluted with 10 mL of DCM and 10 mL of sat. $NaHCO_3$ (aq.). Another addition of 15 mL of DCM to remove organic impurities. Then extracted from the aqueous layer with EtOAc (2 x 15 mL). The organic layers were combined, dried over anhydrous Na_2SO_4, filtered, and concentrated *in vacuo* to afford the trifluoromethylthiolated triflate salt **6e**.

Yield: 55 mg (0.142 mmol, 71%) of white solid.

mp 156–160 °C

^{1}H NMR (400 MHz, MeOD-*d₄*): δ = 7.42 (d, J = 2.3 Hz, 1H), 7.27 (dd, J = 8.4, 2.3 Hz, 1H), 6.92 (d, J = 8.4 Hz, 1H), 3.04 (dd, J = 8.6, 6.8 Hz, 2H), 2.82 (dd, J = 8.6, 6.7 Hz, 2H).

^{13}C{^{1}H} NMR (101 MHz, MeOD-d_4): δ = 160.6, 139.6, 134.7, 131.3 (q, J = 307.8 Hz), 129.9, 118.1, 110.7 (q, J = 1.9), 42.5, 34.6.

^{19}F NMR (376 MHz, MeOD-d_4): δ = –44.58 (3F, CF_3S), –80.20 (3F, triflate).

IR (neat, cm^{-1}): 3128, 1603, 1491, 1255, 1231, 1169, 1126, 1101, 1033, 942, 819, 765, 632, 585, 515, 484, 417.

HRMS (ESI) m/z: $[M + H]^+$ Calcd for $C_9H_{11}F_3NOS$ 238.0508; Found: 238.0501.

***N*-Fmoc-(2-(trifluoromethyl)thio)-dopamine (6f)**

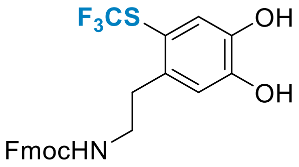

Prepared following the general trifluoromethylthiolation procedure from *N*-Fmoc-dopamine **5f** (37.6 mg, 0.10 mmol, 1.0 equiv), SCF_3 reagent **1** (28 mg, 0.12 mmol, 1.2 equiv), TfOH (22 µL, 0.25 mmol, 2.5 equiv), DCM (1 mL, 0.1 M), rt, 24 h. Isolation: diluted with 15 mL of DCM and washed with 10% HCl (aq.) (2 x 10 mL), and brine (1 x 10 mL). Purification: silica gel CC (dry loading; mobile phase: EtOAc/*n*-hex 1:1) to afford the trifluoromethylthiolated product **6f**.

Yield: 33.1 mg (0.070 mmol, 70%; mixture of rotamers: 84:16) of white solid.

mp 75–78 °C

^{1}H NMR (400 MHz, MeOD-d_4) of the major rotamer: δ = 7.78 (d, J = 7.5 Hz, 2H), 7.62 (d, J = 7.4 Hz, 2H), 7.37 (dd, J = 7.5, 1.1 Hz, 2H), 7.30 (dd, J = 7.4, 1.2 Hz, 2H), 7.08 (s, 1H), 6.82 (s, 1H), 4.30 (d, J = 7.0 Hz, 2H), 4.18 (t, J = 6.9 Hz, 1H), 3.30–3.25 (m, 2H), 2.95 (dd, J = 8.3, 6.4 Hz, 2H).

^{13}C{^{1}H} NMR (101 MHz, MeOD-d_4): δ = 158.8, 150.2, 145.6, 145.3, 142.6, 138.2, 131.3 (q, J = 307.7 Hz), 128.7, 128.1, 126.2, 125.7, 120.9, 118.4, 112.9, 67.8, 48.4, 43.1, 34.8.

^{19}F NMR (376 MHz, MeOD-d_4): δ = –45.56.

IR (neat, cm^{-1}): 3330, 1686, 1656, 1597, 1538, 1507, 1477, 1447, 1266, 1252, 1124, 1102, 1046, 1005, 780, 755, 730.

HRMS (ESI) m/z: $[M + H]^+$ Calcd for $C_{24}H_{21}F_3NO_4S$ 476.1138; Found: 476.1135.

(2-(trifluoromethyl)thio)-dopamine trifluoromethanesulfonate salt (6g)

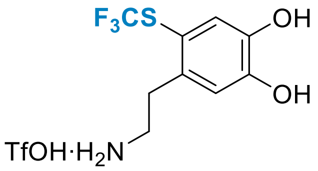

Prepared following the general trifluoromethylthiolation procedure from dopamine hydrochloride **5g** (38.0 mg, 0.20 mmol, 1.0 equiv), SCF_3 reagent **1** (115 mg, 0.50 mmol, 2.5 equiv), TfOH (2 x 44 µL, 0.50 mmol, 5.0 equiv; 2nd addition after 18 h), DCM (2 mL, 0.1 M), rt, 24 h. Isolation: diluted with 10 mL of DCM and 10 mL of sat. $NaHCO_3$ (aq.). Another addition of DCM (2 x 10 mL) to remove impurities in the organic layer. Then extracted from the aqueous layer with EtOAc (4 x 10 mL). The organic layers were combined, dried over anhydrous Na_2SO_4, filtered, and concentrated *in vacuo* to afford the trifluoromethylthiolated triflate salt **6g**.

Yield: 55 mg (0.142 mmol, 68%) of off-white solid.

mp 124–128 °C

^{1}H NMR (400 MHz, MeOD-d_4): δ = 7.08 (s, 1H), 6.80 (s, 1H), 3.10–3.03 (m, 2H), 3.03–2.97 (m, 2H).

^{13}C{^{1}H} NMR (101 MHz, MeOD-d_4): δ = 151.9, 146.9, 136.1, 131.2 (q, J = 307.7 Hz), 125.7, 118.4, 111.7, 42.3, 33.9.

^{19}F NMR (376 MHz, MeOD-d_4): δ = –44.75 (3F, CF_3S), –79.21 (3F, triflate).

IR (neat, cm^{-1}): 3182, 1600, 1503, 1449, 1358, 1258, 1232, 1169, 1104, 1028, 884, 809, 755, 633, 516, 475, 419.

HRMS (ESI) m/z: $[M + H]^+$ Calcd for $C_9H_{11}F_3NO_2S$ 254.0457; Found: 254.0457.

***N*-Fmoc-2-((trifluoromethyl)thio)-L-DOPA (6h)**

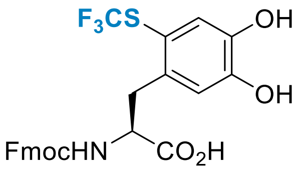

Prepared following the general trifluoromethylthiolation procedure from Fmoc-L-DOPA **5h** (42.2 mg, 0.10 mmol, 1.0 equiv), SCF_3 reagent **1** (24.7 mg, 0.11 mmol, 1.1 equiv), TfOH (22 µL, 0.25 mmol, 2.5 equiv), DCM (1 mL, 0.1 M), rt, overnight. Isolation: diluted with 15 mL of DCM and washed with 1 M HCl (aq.) (2 x 10 mL), and brine (1 x 10 mL). The organic layer was dried over anhydrous Na_2SO_4, filtered, and concentrated *in vacuo* (several coevaporations with pentane) to afford the trifluoromethylthiolated product **6h**.

Yield: 51 mg (0.097 mmol, 97%; mixture of rotamers: 86:14) of yellow foamy solid.

mp 181–185 °C

$[\alpha]_D^{20}$ = –8.9 (*c* 0.575, MeOH)

^{1}H NMR (400 MHz, DMSO-*d_6*) of the major rotamer: δ = 12.70 (bs, 1H), 9.69 (bs, 1H), 9.52 (bs, 1H), 7.87 (d, *J* = 7.5 Hz, 2H), 7.79 (d, *J* = 8.5 Hz, 1H), 7.66 (dd, *J* = 11.0, 7.5 Hz, 2H), 7.40 (ddd, *J* = 7.5, 3.1, 1.2 Hz, 2H), 7.31 (dd, *J* = 7.4, 1.2 Hz, 2H), 7.06 (s, 1H), 6.93 (s, 1H), 4.31–4.09 (m, 4H), 3.30 (dd, *J* = 14.0, 5.3 Hz, 1H), 2.97 (dd, *J* = 14.0, 9.9 Hz, 1H).

^{13}C{^{1}H} NMR (101 MHz, DMSO-*d_6*): δ = 173.2, 156.0, 148.9, 144.8, 143.8, 140.7, 134.9, 129.8 (q, *J* = 308.6 Hz), 127.7, 127.1, 125.4, 125.3, 124.5, 120.1, 118.5, 110.5, 65.8, 55.0, 46.6, 34.4.

^{19}F NMR (376 MHz, DMSO-*d_6*): δ = –42.81.

IR (neat, cm^{-1}): 3312, 3069, 2927, 1695, 1511, 1275, 1231, 1102, 757, 739, 621, 540, 478.

HRMS (ESI) m/z: $[M + Na]^+$ Calcd for $C_{25}H_{20}F_3NNaO_6S$ 542.0856; Found: 542.0867.

N-Fmoc-2-((trifluoromethyl)thio)-L-DOPA benzyl ester (6i)

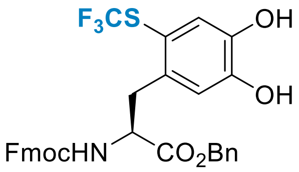

Prepared following the general trifluoromethylthiolation procedure from Fmoc-L-DOPA benzyl ester **5i** (51 mg, 0.10 mmol, 1.0 equiv), SCF_3 reagent **1** (27.3 mg, 0.12 mmol, 1.2 equiv), $BF_3 \cdot OEt_2$ (2 x 31 µL, 0.50 mmol, 5.0 equiv; 2nd addition after 7 h), DCE (1 mL, 0.1 M), 50 °C, 24 h. Isolation: diluted with 15 mL of DCM and washed with 1 M HCl (aq.) (1 x 10 mL), brine (1 x 10 mL), and sat. $NaHCO_3$ (aq.) (1 x 10 mL). Purification: silica gel CC (dry loading; mobile phase: EtOAc/*n*-hex 1:3) to afford the trifluoromethylthiolated product **6i**.

Yield: 47 mg (0.077 mmol, 77%; mixture of rotamers: 85:15) of off-white solid.

mp 132–134 °C

$[\alpha]_D^{20}$ = –8.1 (*c* 1.55, MeOH)

^{1}H NMR (400 MHz, MeOD-*d_4*) of the major rotamer: δ = 7.76 (d, *J* = 7.5 Hz, 2H), 7.58 (d, *J* = 7.5 Hz, 2H), 7.40–7.33 (m, 2H), 7.31–7.22 (m, 7H), 7.07 (s, 1H), 6.84 (s, 1H), 5.10 (d, *J* = 2.2 Hz, 2H), 4.48 (dd, *J* = 9.0, 6.5 Hz, 1H), 4.29–4.21 (m, 2H), 4.13 (t, *J* = 7.2 Hz, 1H), 3.39 (dd, *J* = 13.9, 6.5 Hz, 1H), 3.09 (dd, *J* = 13.9, 9.0 Hz, 1H).

$^{13}C\{^1H\}$ NMR (101 MHz, MeOD-d_4): δ = 173.2, 158.4, 150.1, 146.1, 145.2, 145.1, 142.5, 137.0, 135.9, 131.2 (q, J = 307.8 Hz), 129.5, 129.2, 129.1, 128.7, 128.2, 126.3, 126.3, 125.9, 120.9, 119.0, 113.4, 68.2, 67.9, 56.8, 48.3, 36.4.

^{19}F NMR (376 MHz, MeOD-d_4): δ = –45.51.

IR (neat, cm^{-1}): 3401, 3316, 3065, 2927, 2855, 1690, 1511, 1255, 1148, 1126, 1103, 1031, 755, 733, 694.

HRMS (ESI) m/z: $[M + H]^+$ Calcd for $C_{32}H_{27}F_3NO_6S$ 610.1506; Found: 610.1505.

3.6. Late-stage trifluoromethylthiolation of Trp and Tyr containing di- and tripeptides 7a-g

Fmoc-L-(CF_3S)Trp-L-Leu-OBn (8a)

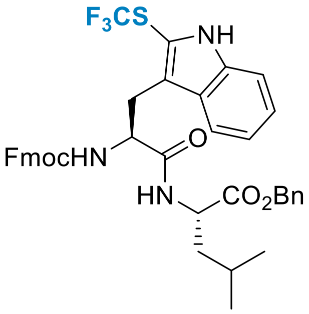

Prepared following the general trifluoromethylthiolation procedure from Fmoc-L-Trp-L-Leu-OBn **7a** (63.0 mg, 0.10 mmol, 1.0 equiv), SCF_3 reagent **1** (27.5 mg, 0.12 mmol, 1.2 equiv), $BF_3{\cdot}OEt_2$ (2 x 32 µL, 0.50 mmol, 5.0 equiv; 2nd addition after 24 h), DCE (1 mL, 0.1 M), 50 °C, 48 h. Isolation: diluted with 15 mL of DCM and washed with 10% HCl (aq.) (2 x 10 mL), brine (1 x 10 mL), and sat. $NaHCO_3$ (aq.) (1 x 10 mL). Purification: silica gel CC (dry loading; mobile phase: EtOAc/*n*-hex 1:4 → 1:2) to afford the trifluoromethylthiolated product **8a**.

Yield: 54 mg (0.074 mmol, 74%; mixture of rotamers: 88:12) of white-yellowish solid.

mp 140–143 °C

^{1}H NMR (500 MHz, $CDCl_3$) of the major rotamer: δ = 8.19 (s, 1H), 7.76 (d, *J* = 7.7 Hz, 3H), 7.60–7.49 (m, 2H), 7.43–7.27 (m, 11H), 7.16 (ddd, *J* = 8.0, 7.0, 1.1 Hz, 1H), 5.91 (d, *J* = 8.2 Hz, 1H), 5.60 (d, *J* = 8.0 Hz, 1H), 5.05 (s, 2H), 4.65–4.45 (m, 2H), 4.39–4.28 (m, 2H), 4.17 (t, *J* = 7.2 Hz, 1H), 3.41 (dd, *J* = 14.4, 6.4 Hz, 1H), 3.35 (dd, *J* = 14.1, 8.1 Hz, 1H), 1.56–1.43 (m, 2H), 1.43–1.35 (m, 1H), 0.84 (d, *J* = 6.2 Hz, 3H), 0.81 (d, *J* = 6.3 Hz, 3H).

^{13}C{^{1}H} NMR (126 MHz, $CDCl_3$): δ = 172.1, 170.4, 155.9, 143.9, 143.8, 141.4, 137.6, 135.4, 128.8, 128.6, 128.5 (q, *J* = 311.7 Hz), 128.4, 127.9, 127.2, 127.2, 127.1, 125.3, 125.2, 121.6, 121.0, 120.1, 120.1, 115.2, 111.6, 67.3, 67.2, 55.3, 51.1, 47.2, 42.0, 28.6, 24.8, 22.7, 22.2.

^{19}F NMR (471 MHz, $CDCl_3$): δ = –42.54.

IR (neat, cm^{-1}): 3400, 3295, 3064, 2955, 1720, 1685, 1650, 1533, 1448, 1318, 1271, 1248, 1131, 1107, 1040, 734, 696, 668.

HRMS (ESI) m/z: $[M + H]^+$ Calcd for $C_{40}H_{39}F_3N_3O_5S$ 730.2557; Found: 730.2552.

H_2N-L-(CF_3S)Trp-L-Leu-OMe (8b)

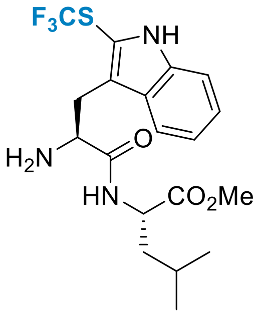

Prepared following the general trifluoromethylthiolation procedure from H_2N-L-Trp-L-Leu-OMe **7b** (33.7 mg, 0.10 mmol, 1.0 equiv), SCF_3 reagent **1** (26.0 mg, 0.11 mmol, 1.1 equiv), $BF_3{\cdot}OEt_2$ (31 µL, 0.25 mmol, 2.5 equiv), DCM (1 mL, 0.1 M), rt, 16 h. Isolation: diluted with 15 mL of DCM and washed with sat. $NaHCO_3$ (aq.) (1 x 10 mL). The remaining aqueous phase was then extracted with DCM (2 x 10 mL). Purification: silica gel CC (dry loading; mobile phase: DCM → DCM/MeOH 10:1) to afford the trifluoromethylthiolated product **8b**.

Yield: 35.2 mg (0.082 mmol, 80%) of yellowish solid.

mp 81–85 °C

^{1}H NMR (500 MHz, $CDCl_3$): δ = 8.70 (s, 1H), 7.72 (dd, J = 8.1, 1.0 Hz, 1H), 7.66 (d, J = 8.5 Hz, 1H), 7.43–7.36 (m, 1H), 7.31 (ddd, J = 8.2, 7.0, 1.1 Hz, 1H), 7.15 (ddd, J = 8.1, 7.0, 1.0 Hz, 1H), 4.61 (td, J = 8.6, 5.3 Hz, 1H), 3.81 (dd, J = 9.7, 4.1 Hz, 1H), 3.71 (s, 3H), 3.58 (dd, J = 14.5, 4.0 Hz, 1H), 3.06 (dd, J = 14.5, 9.7 Hz, 1H), 1.67–1.53 (m, 3H), 0.93 (d, J = 5.2 Hz, 3H), 0.92 (d, J = 5.1 Hz, 3H).

^{13}C{^{1}H} NMR (126 MHz, $CDCl_3$): δ = 174.4, 173.6, 137.8, 128.5 (q, J = 311.9 Hz), 127.2, 125.2, 123.1, 120.7, 120.3, 114.9 (q, J = 2.3 Hz), 111.5, 55.6, 52.4, 50.6, 41.6, 30.4, 25.0, 22.9, 22.2.

^{19}F NMR (471 MHz, $CDCl_3$): δ = –42.46.

IR (neat, cm^{-1}): 3270, 2957, 1738, 1656, 1515, 1438, 1342, 1275, 1206, 1129, 1105, 908, 843, 731, 647.

HRMS (ESI) m/z: $[M + H]^+$ Calcd for $C_{19}H_{25}F_3N_3O_3S$ 432.1563; Found: 432.1564.

Fmoc-L-Ala-L-(CF_3S)Trp-OBn (8c)

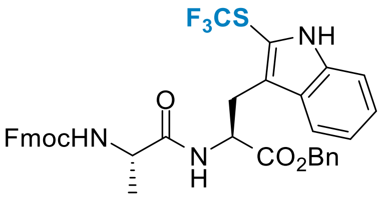

Prepared following the general trifluoromethylthiolation procedure from Fmoc-L-Ala-L-Trp-OBn **7c** (58.8 mg, 0.10 mmol, 1.0 equiv), SCF_3 reagent **1** (24 mg, 0.11 mmol, 1.1 equiv), $BF_3{\cdot}OEt_2$ (48% solution, 2 x 62 µL, 0.50 mmol, 5.0 equiv; 2nd addition after 6 h), DCE (1 mL, 0.1 M), 50 °C, 16 h. Isolation: diluted with 15 mL of DCM and washed with 1 M HCl (aq.) (1 x 10 mL), brine (1 x 10 mL), and sat. $NaHCO_3$ (aq.) (1 x 10 mL). Purification: silica gel CC (dry loading; mobile phase: EtOAc/*n*-hex 7:3) to afford the trifluoromethylthiolated product **8c**.

Yield: 51 mg (0.074 mmol, 74%; mixture of rotamers: 88:12) of white-yellowish solid.

mp 76–80 °C

^{1}H NMR (400 MHz, $CDCl_3$) of the major rotamer: δ = 8.25 (s, 1H), 7.78 (d, J = 7.6 Hz, 2H), 7.62–7.56 (m, 3H), 7.44–7.38 (m, 2H), 7.35–7.22 (m, 7H), 7.18 (dd, J = 6.6, 2.9 Hz, 2H), 7.11 (ddd, J = 8.1, 6.4, 1.5 Hz, 1H), 6.51 (d, J = 7.9 Hz, 1H), 5.19 (d, J = 7.8 Hz, 1H), 5.12–4.95 (m, 3H), 4.39 (dd, J = 10.5, 7.2 Hz, 1H), 4.30 (dd, J = 10.4, 6.9 Hz, 1H), 4.23–4.15 (m, 2H), 3.48 (dd, J = 14.5, 6.8 Hz, 1H), 3.39 (dd, J = 14.5, 6.3 Hz, 1H), 1.26 (s, 3H).

$^{13}C\{^1H\}$ NMR (101 MHz, $CDCl_3$): δ = 171.9, 171.3, 155.9, 144.0, 141.5, 137.5, 135.0, 128.7, 128.6, 128.4 (q, J = 311.9 Hz), 128.4, 127.9, 127.4, 127.2, 125.2, 125.2, 121.2, 120.8, 120.2, 119.9, 114.9, 111.6, 67.6, 67.1, 52.8, 50.5, 47.2, 31.1, 29.8, 27.7, 18.8.

^{19}F NMR (376 MHz, $CDCl_3$): δ = –42.69.

IR (neat, cm^{-1}): 3298, 3065, 2957, 1724, 1658, 1508, 1449, 1393, 1342, 1213, 1105, 739, 696, 621, 542, 460.

HRMS (ESI) m/z: $[M + H]^+$ Calcd for $C_{37}H_{33}F_3N_3O_5S$ 688.2088; Found: 688.2090.

Fmoc-L-Ala-L-(CF_3S)Trp-OEt (8d)

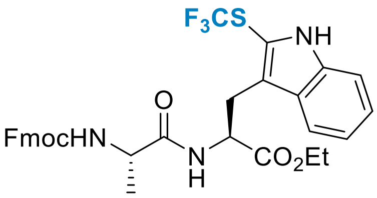

Prepared following the general trifluoromethylthiolation procedure from Fmoc-L-Ala-L-Trp-OEt (**7d**) (52.9 mg, 0.10 mmol, 1.0 equiv), SCF_3 reagent **1** (26.1 mg, 0.11 mmol, 1.1 equiv), TfOH (22 µL, 0.25 mmol, 2.5 equiv), DCM (1 mL, 0.1 M), rt, 16 h. Isolation: diluted with 15 mL of DCM and washed with 10% HCl (aq.) (2 x 10 mL), brine (1 x 10 mL), and sat. $NaHCO_3$ (aq.) (1 x 10 mL). Purification: silica gel CC (dry loading; mobile phase: EtOAc/*n*-hex 1:1) to afford the trifluoromethylthiolated product **8d**.

Yield: 49.6 mg (0.079 mmol, 79%; mixture of rotamers: 88:12) of yellow solid.

mp 210–212 °C

^1H NMR (500 MHz, DMSO-d_6) of the major rotamer: δ = 11.91 (s, 1H), 8.40 (d, J = 7.5 Hz, 1H), 7.89 (d, J = 7.5 Hz, 2H), 7.77–7.68 (m, 2H), 7.63 (d, J = 8.1 Hz, 1H), 7.47 (d, J = 7.9 Hz, 1H), 7.44–7.36 (m, 3H), 7.35–7.29 (m, 2H), 7.28–7.18 (m, 1H), 7.13–7.03 (m, 1H), 4.55–4.47 (m, 1H), 4.31–4.06 (m, 4H), 3.97–3.75 (m, 2H), 3.35–3.28 (m, 1H), 3.25 (dd, J = 14.2 Hz, 6.9, 1H), 1.19 (d, J = 7.1 Hz, 3H), 0.91 (t, J = 7.1 Hz, 3H).

$^{13}C\{^1H\}$ NMR (126 MHz, DMSO-d_6): δ = 172.7, 171.2, 155.6, 143.9, 143.8, 140.7, 137.6, 128.6 (q, J = 311.3 Hz), 127.6, 127.1, 126.6, 125.3, 125.3, 124.2, 120.8, 120.1, 119.7, 113.5, 111.8, 65.6, 60.4, 52.9, 49.7, 46.7, 26.6, 18.2, 13.6.

^{19}F NMR (471 MHz, DMSO-d_6): δ = –42.23.

IR (neat, cm^{-1}): 3383, 3294, 1726, 1694, 1655, 1534, 1447, 1369, 1324, 1286, 1257, 1237, 1149, 1116, 1079, 1047, 1031, 736, 702, 679, 651.

HRMS (ESI) m/z: $[M + H]^+$ Calcd for $C_{32}H_{31}F_3N_3O_5S$ 626.1931; Found: 626.1924.

Fmoc-L-Ala-L-(CF_3S)Trp-L-Leu-OMe (8e)

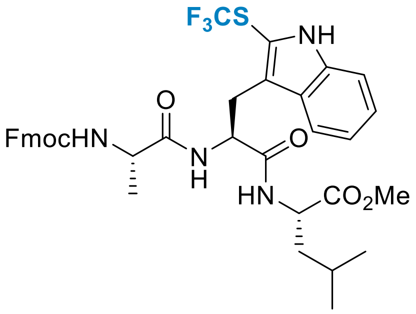

Prepared following the general trifluoromethylthiolation procedure from Fmoc-L-Ala-L-Trp-L-Leu-OMe (**7e**) (62.5 mg, 0.10 mmol, 1.0 equiv), SCF_3 reagent **1** (26.4 mg, 0.12 mmol, 1.2 equiv), TfOH (22 µL, 0.25 mmol, 2.5 equiv), DCM (1 mL, 0.1 M), rt, 16 h. Isolation: diluted with 15 mL of DCM and washed with 10% HCl (aq.) (2 x 10 mL), brine (1 x 10 mL), and sat. $NaHCO_3$ (aq.) (1 x 10 mL). Purification: silica gel CC (dry loading; mobile phase: EtOAc/*n*-hex 1:1) to afford the trifluoromethylthiolated product **8e**.

Yield: 48 mg (0.066 mmol, 66%; mixture of rotamers: 88:12) of white-yellowish solid.

mp 183–186 °C

^{1}H NMR (500 MHz, DMSO-*d₆*) of the major rotamer: δ = 11.79 (s, 1H), 8.12 (d, *J* = 7.9 Hz, 1H), 7.95 (d, *J* = 8.2v, 1H), 7.89 (d, *J* = 7.6 Hz, 2H), 7.74 (d, *J* = 8.7 Hz, 1H), 7.75–7.68 (m, 2H), 7.52 (d, *J* = 7.4 Hz, 1H), 7.43–7.39 (m, 2H), 7.37 (d, *J* = 8.3 Hz, 1H), 7.35–7.30 (m, 2H), 7.26–7.16 (m, 1H), 7.11–7.00 (m, 1H), 4.63–4.55 (m, 1H), 4.32–4.11 (m, 4H), 4.04–3.97 (m, 1H), 3.52 (s, 3H), 3.30 (dd, *J* = 14.1, 6.9 Hz, 1H), 3.11 (dd, *J* = 14.1, 7.3 Hz, 1H), 1.55–1.36 (m, 3H), 1.08 (d, *J* = 7.1 Hz, 3H), 0.80 (d, *J* = 6.4 Hz, 3H), 0.76 (d, *J* = 6.4 Hz, 3H).

^{13}C{^{1}H} NMR (126 MHz, DMSO-d_6): δ = 172.4, 172.2, 170.6, 155.7, 143.9, 143.8, 140.7, 137.6, 128.9, 128.8 (q, *J* = 311.3 Hz), 127.7, 127.3, 127.1, 126.8, 125.3, 125.3, 123.9, 121.4, 121.1, 120.1, 119.4, 113.6, 111.6, 65.7, 53.1, 51.8, 50.1, 46.6, 27.3, 24.0, 22.6, 21.4, 18.0.

^{19}F NMR (471 MHz, DMSO-d_6): δ = –42.42.

IR (neat, cm^{-1}): 3397, 3285, 2956, 2165, 2114, 1740, 1688, 1643, 1530, 1447, 1238, 1133, 1106, 1046, 754, 740, 680, 661.

HRMS (ESI) m/z: $[M + H]^+$ Calcd for $C_{37}H_{40}F_3N_4O_6S$ 725.2615; Found: 725.2607.

Fmoc-L-(CF_3S)Trp-L-(CF_3S)Trp-OEt (8f)

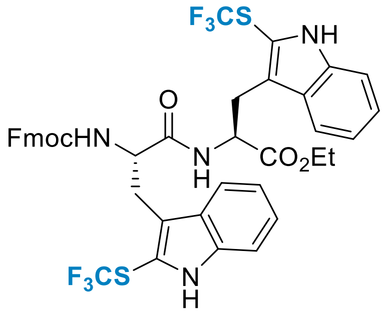

Prepared following the general trifluoromethylthiolation procedure from Fmoc-L-Trp-L-Trp-OEt (**7f**) (63.9 mg, 0.10 mmol, 1.0 equiv), SCF_3 reagent **1** (50.6 mg, 0.22 mmol, 2.2 equiv),

$BF_3 \cdot OEt_2$ (2 x 61.7 µL, 1.0 mmol, 10 equiv; 2nd addition after 1 h), DCE (1 mL, 0.1 M), 50 °C, 48 h. Isolation: diluted with 15 mL of DCM and washed with 1 M HCl (aq.) (2 x 10 mL), brine (1 x 10 mL), and sat. $NaHCO_3$ (aq.) (1 x 10 mL). Purification: silica gel CC (dry loading; mobile phase: EtOAc/*n*-hex 1:3) to afford the trifluoromethylthiolated product **8f**.

Yield: 60 mg (0.071 mmol, 71%; mixture of rotamers: 85:15) of white solid.

mp 244–246 °C

^{1}H NMR (400 MHz, DMSO-*d*$_6$) of the major rotamer: δ = 11.91 (s, 1H), 11.82 (s, 1H), 8.47 (d, *J* = 7.5 Hz, 1H), 7.87 (d, *J* = 7.6 Hz, 2H), 7.76 (d, *J* = 8.1 Hz, 1H), 7.65–7.60 (m, 3H), 7.47 (d, *J* = 9.0 Hz, 1H), 7.44–7.34 (m, 4H), 7.33–7.18 (m, 4H), 7.11–7.05 (m, 1H), 7.05–7.01 (m, 1H), 4.58–4.50 (m, 1H), 4.45 (td, *J* = 8.7, 5.7 Hz, 1H), 4.15–4.07 (m, 3H), 3.90–3.78 (m, 2H), 3.34–3.17 (m, 3H), 3.11 (dd, *J* = 14.2, 8.7 Hz, 1H), 0.89 (t, *J* = 7.1 Hz, 3H).

^{13}C{^{1}H} NMR (101 MHz, DMSO-*d*$_6$): δ = 171.1, 170.9, 155.5, 143.8, 143.7, 140.7, 140.7, 137.6, 137.6, 128.72 (q, *J* = 311.6 Hz), 128.56 (q, *J* = 311.5 Hz), 127.7, 127.1, 126.8, 126.6, 125.4, 124.2, 121.3, 120.7, 120.1, 120.0, 119.7, 119.4, 113.7, 113.5 (q, *J* = 2.4), 111.8, 111.7, 65.8, 60.5, 55.2, 52.9, 46.5, 27.7, 26.8, 13.6.

^{19}F NMR (376 MHz, DMSO-*d*$_6$): δ = –42.42, –42.50.

IR (neat, cm^{-1}): 3385, 3316, 3061, 2935, 1697, 1652, 1532, 1165, 1107, 1048, 1026, 754, 736, 671.

HRMS (ESI) m/z: $[M + H]^+$ Calcd for $C_{41}H_{35}F_6N_4O_5S_2$ 841.1948; Found: 841.1947.

Fmoc-L-Tyr-L-(CF_3S)Trp-OEt (8g)

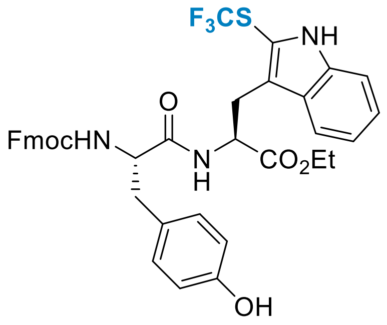

Prepared following the general trifluoromethylthiolation procedure from Fmoc-L-Tyr-L-Trp-OEt (**7g**) (61.8 mg, 0.10 mmol, 1.0 equiv), SCF_3 reagent **1** (23.7 mg, 0.105 mmol, 1.05 equiv), TfOH (22 µL, 0.25 mmol, 2.5 equiv), DCM (1 mL, 0.1 M), rt, 16 h. Isolation: diluted with 15 mL of DCM and washed with 10% HCl (aq.) (2 x 10 mL), brine (1 x 10 mL), and sat. $NaHCO_3$ (aq.) (1 x 10 mL). Purification: silica gel CC (dry loading; mobile phase: EtOAc/*n*-hex 1:1) to afford the trifluoromethylthiolated product **8g**.

Yield: 56 mg (0.078 mmol, 78%; mixture of rotamers: 88:12) of white solid.

mp 214–217 °C

^{1}H NMR (500 MHz, DMSO-*d*$_6$) of the major rotamer: δ = 11.92 (s, 1H), 9.16 (s, 1H), 8.56 (d, *J* = 7.4 Hz, 1H), 7.88 (d, *J* = 7.6 Hz, 2H), 7.65 (d, *J* = 6.8 Hz, 1H), 7.62 (d, *J* = 7.6 Hz, 1H), 7.50 (d, *J* = 8.9 Hz, 1H), 7.43–7.38 (m, 3H), 7.34–7.27 (m, 2H), 7.26–7.22 (m, 1H), 7.10 (d, *J* = 7.9 Hz, 1H), 7.08 (d, *J* = 8.3 Hz, 2H), 6.63 (d, *J* = 8.5 Hz, 2H), 4.58–4.51 (m, 1H), 4.23 (ddd, *J* =

10.7, 8.9, 3.9 Hz, 1H), 4.20–4.05 (m, 3H), 3.93–3.84 (m, 2H), 3.39–3.31 (m, 1H), 3.27 (dd, *J* = 14.2, 6.8 Hz, 1H), 2.83 (dd, *J* = 13.8, 3.9 Hz, 1H), 2.62 (dd, *J* = 13.8, 10.6 Hz, 1H), 0.92 (t, *J* = 7.1 Hz, 3H).

$^{13}C\{^{1}H\}$ NMR (126 MHz, DMSO-d_6): δ = 171.9, 171.2, 155.8, 155.7, 143.8, 143.7, 140.7, 137.6, 130.1, 128.9, 128.6 (q, *J* = 311.5 Hz), 128.1, 127.6, 127.3, 127.1, 127.1, 126.6, 125.4, 125.3, 124.2, 121.4, 120.7, 120.1, 119.7, 119.7, 114.8, 113.5, 111.8, 65.6, 60.5, 56.1, 53.0, 46.6, 36.7, 26.7, 13.6.

^{19}F NMR (471 MHz, DMSO-d_6): δ = –42.21.

IR (neat, cm^{-1}): 3373, , 3292, 1721, 1692, 1651, 1530, 1512, 1447, 1286, 1233, 1146, 1113, 1030, 827, 740, 700, 674, 652.

HRMS (ESI) m/z: $[M + H]^+$ Calcd for $C_{38}H_{35}F_3N_3O_6S$ 718.2193; Found: 718.2185.

4. Synthesis of trifluoromethylthiolated endomorphin-1 analogues

4.1. Late-stage trifluoromethylthiolation of endomorphin-1

Table S2. Late-stage trifluoromethylthiolation of endomorphin-1 (**EM-1·TFA**).

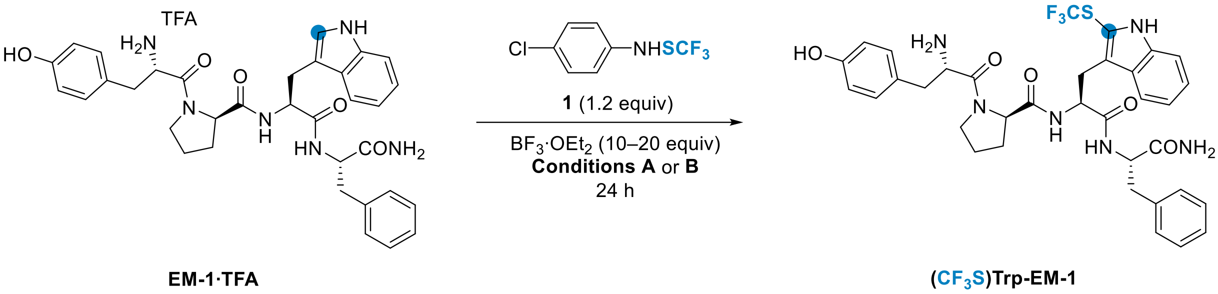

Entry	BF_3·OEt_2 equiv	Conditions	UPLC-MS Conversion (%)[a]
1	10	**A**	40
2	10	**B**	52
3	20	**A**	69

EM-1·TFA (10 mg, 0.014 mmol, 1.0 equiv), SCF_3 reagent **1** (1.2 equiv), BF_3·OEt_2 (10–20 equiv), **Conditions A** (DCM; 0.05 M; rt) or **Conditions B** (DCE; 0.05 M; 50 °C). [a]Conversion to **(CF_3S)Trp-EM-1** determined by UPLC-MS analysis (λ = 215 nm, 10 → 90% MeCN + 0.1% TFA in H_2O + 0.1% TFA, 3 min run).

Optimization procedure: A vial was filled with **EM-1·TFA** (10 mg, 0.014 mmol, 1.0 equiv), SCF_3 reagent (3.8 mg, 0.017 mmol, 1.2 equiv), solvent (DCM or DCE, 274 µL, 0.05 M), and equipped with a magnetic stirr bar when performing the experiment at 50 °C. After shaking the mixture on a platform shaker (or heating on the magnetic stirrer for 50 °C) for 5 min, BF_3·OEt_2 was added (18–35 µL, 0.14–0.28 mmol, 10–20 equiv). After 24 h, the reaction mixture was quenched with 2 mL of 0.2 M $NaHCO_3$ (aq.) and extracted with 2 mL of EtOAc two times. The organic layer was concentrated *in vacuo* and analyzed by UPLC-MS (10 → 90% MeCN + 0.1% TFA in H_2O + 0.1% TFA, λ = 215 nm, 3 min run). The signals at 1.66 min (**EM-1**; *m/z* = 611.59) and 1.88 min (**(CF_3S)Trp-EM-1**; *m/z* = 711.65) were integrated to determine the conversion to **(CF_3S)Trp-EM-1**. The ^{1}H and ^{19}F NMR of the crude neutralized peptide was performed in MeOD-d_4 (400 MHz).

Entry 1 (Table S2):

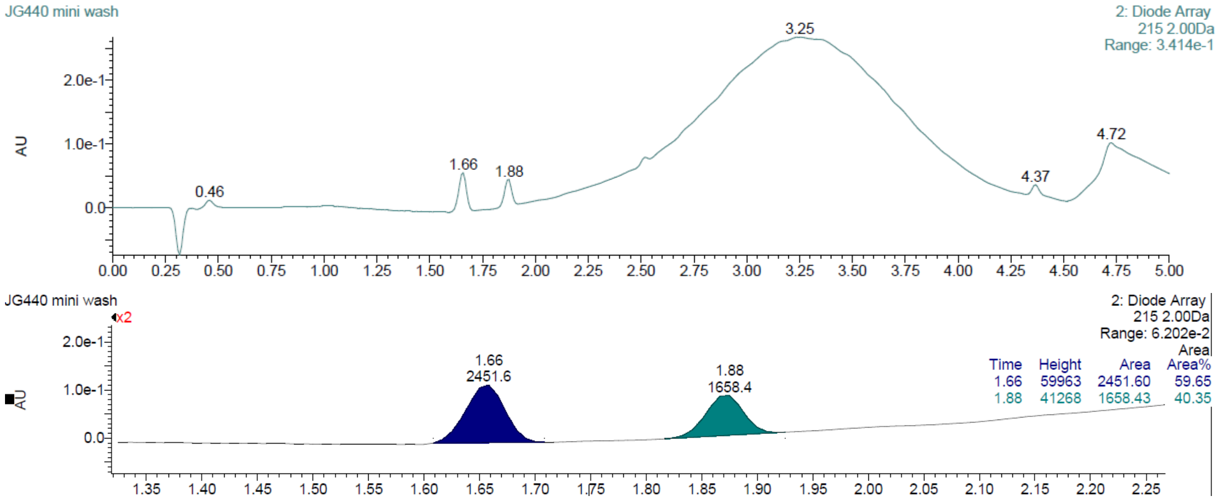

Entry 2 (Table S2):

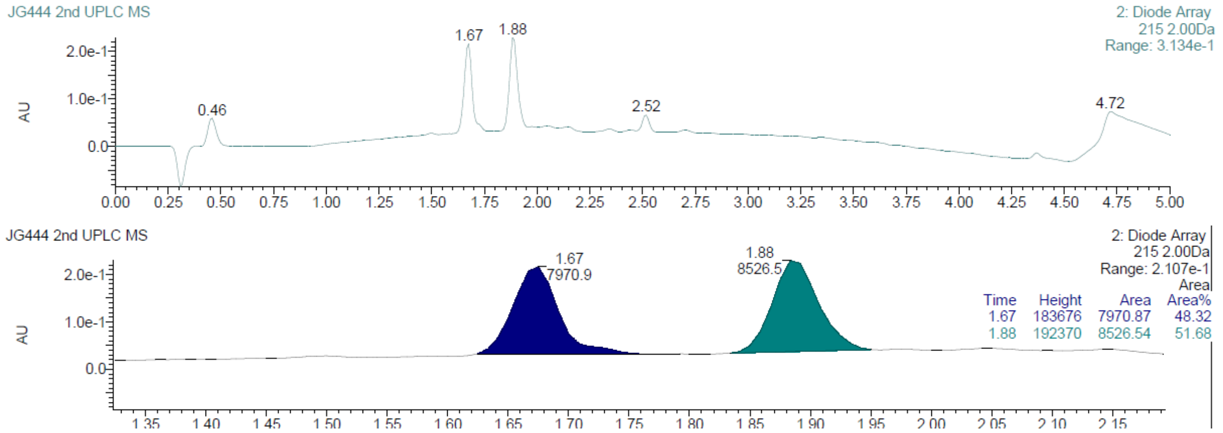

Entry 3 (Table S2):

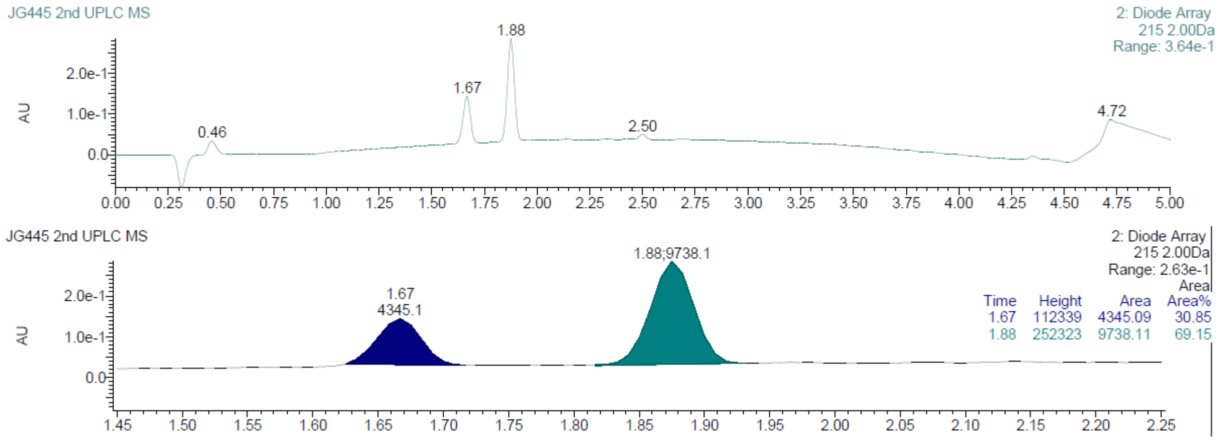

MS spectrum (1.66 min):

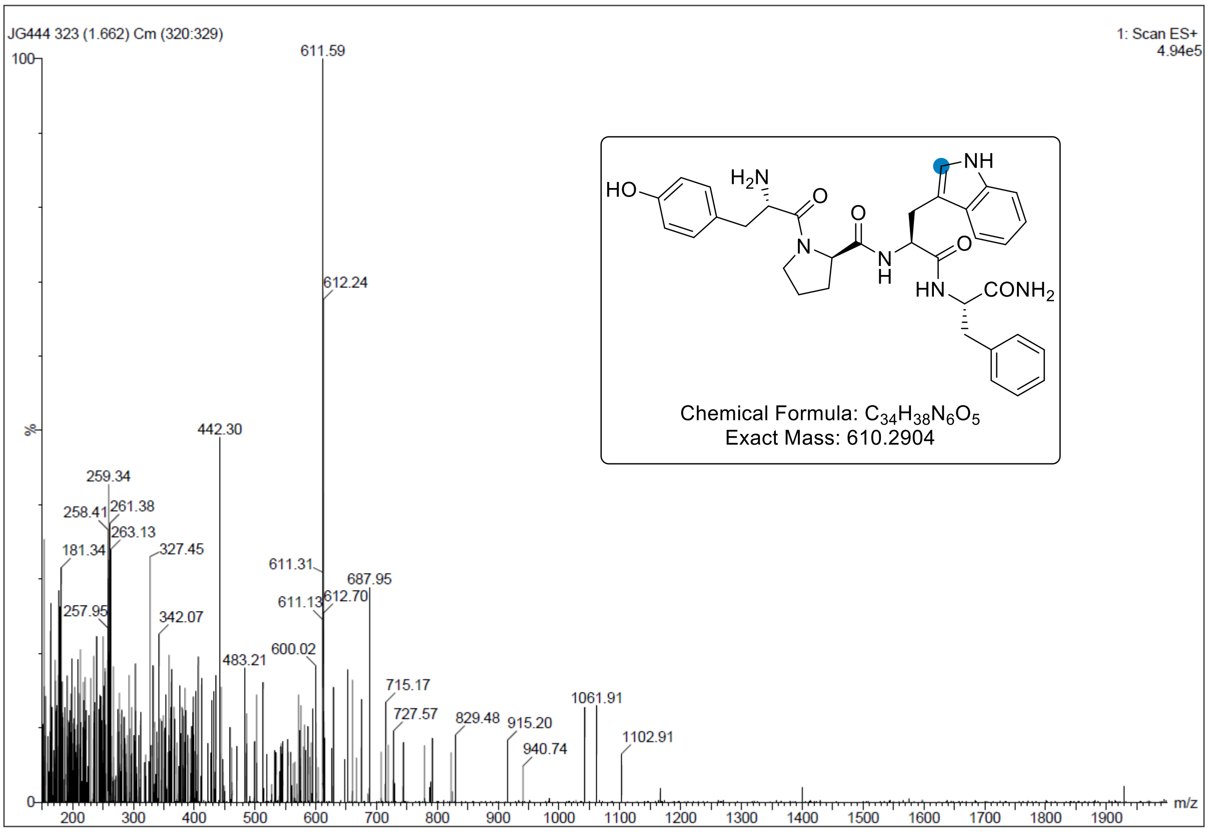

MS spectrum (1.92 min):

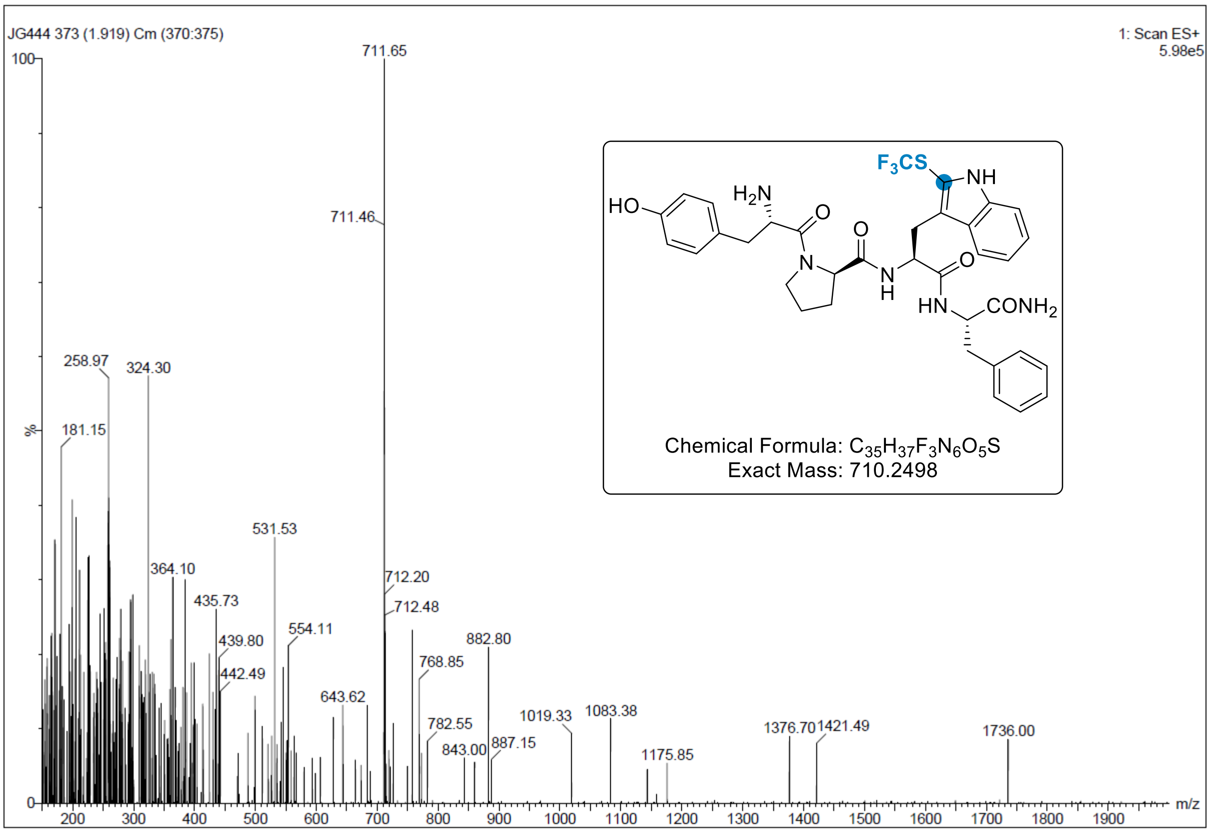

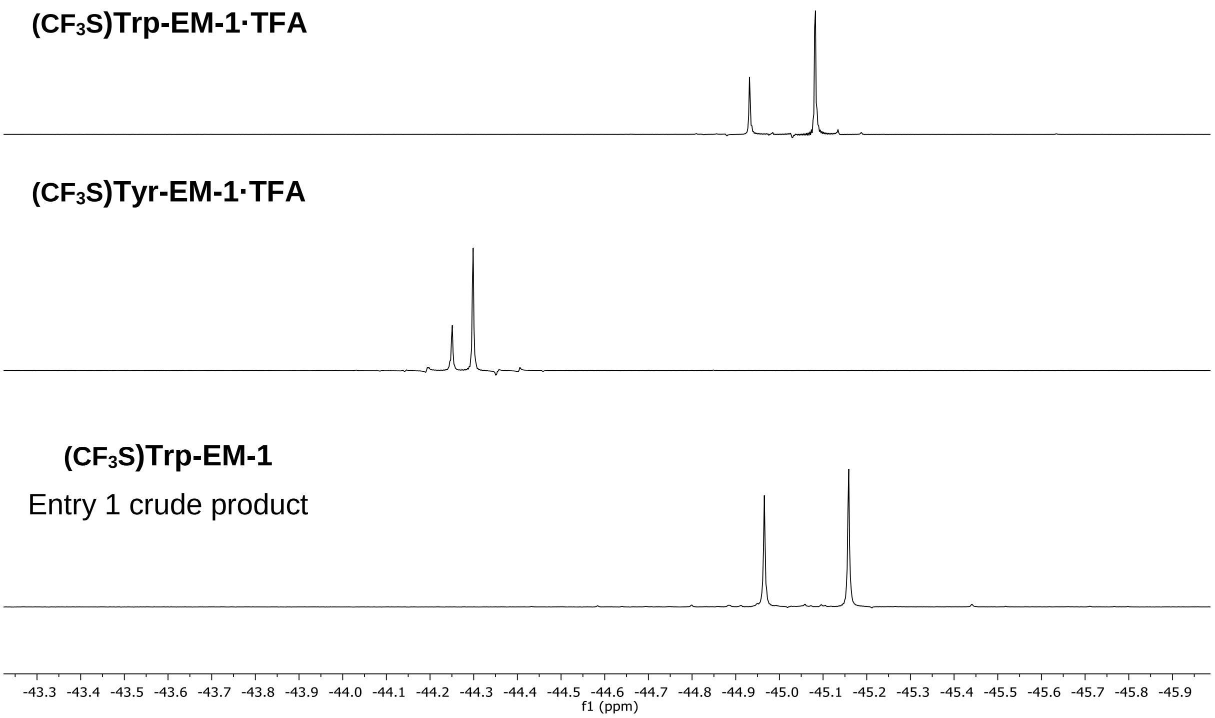

Figure S3: ^{19}F NMR spectra of **(CF_3S)Trp-EM-1·TFA**, **(CF_3S)Trp-EM-1·TFA**, and the crude product after late-stage trifluoromethylthiolation (Entry 1) in MeOD-d_4 (400 MHz, referenced to C_6F_6 at –165.37 ppm).

4.2. SPPS of endomorphin-1 analogues

General Procedure for solid-phase peptide synthesis. SPPS of CF_3S-EM-1s and tripeptides was performed manually, using standard Fmoc-strategy procotols in plastic syringes. Fmoc removal was performed by using 20% piperidine in DMF (1 mL per 100 mg of resin) for 5 and then 15 min. Peptide coupling of commercial amino acids was performed using 3 equiv of the corresponding Fmoc-AA-OH, 3 equiv of HATU, and 5 equiv of DIPEA for 45–60 min. (CF_3S)-residues were coupled using 1.5 equiv of Fmoc-(CF_3S)AA-OH, 3 equiv of HATU and 3 equiv of DIPEA for 2 h to overnight. EM-1 analogues were synthesized on a Fmoc-Rink amide AM polystyrene resin (0.64 mmol/g), while peptides **9-12** were synthesized on a preloaded Fmoc-Leu-Wang resin (0.80 mmol/g). The individual coupling steps were analyzed by Kaiser and Chloranil assays and/or small scale cleavages, analyzed by UPLC-MS. The starting **EM-1·TFA** for late-stage CF_3S-introduction was prepared using the Liberty Blue 2.0 Microwave peptide synthesizer. Peptide cleavages were performed with a cleavage solution of TFA/H_2O/TIPS 95:2.5:2.5 (v/v/v) (1 mL per 100 mg of resin) at rt for 2.5 h. Afterwards, the vessel was washed with DCM (2 x 3 mL) and the volatile components were evaporated using air flow. The crude peptides were then precipitated in cold Et_2O and freeze-dried. The crude products were purified by semi-preparative HPLC. All peptides were obtained with purity greater than 95% (RP-HPLC analysis, detection at λ = 210 nm).

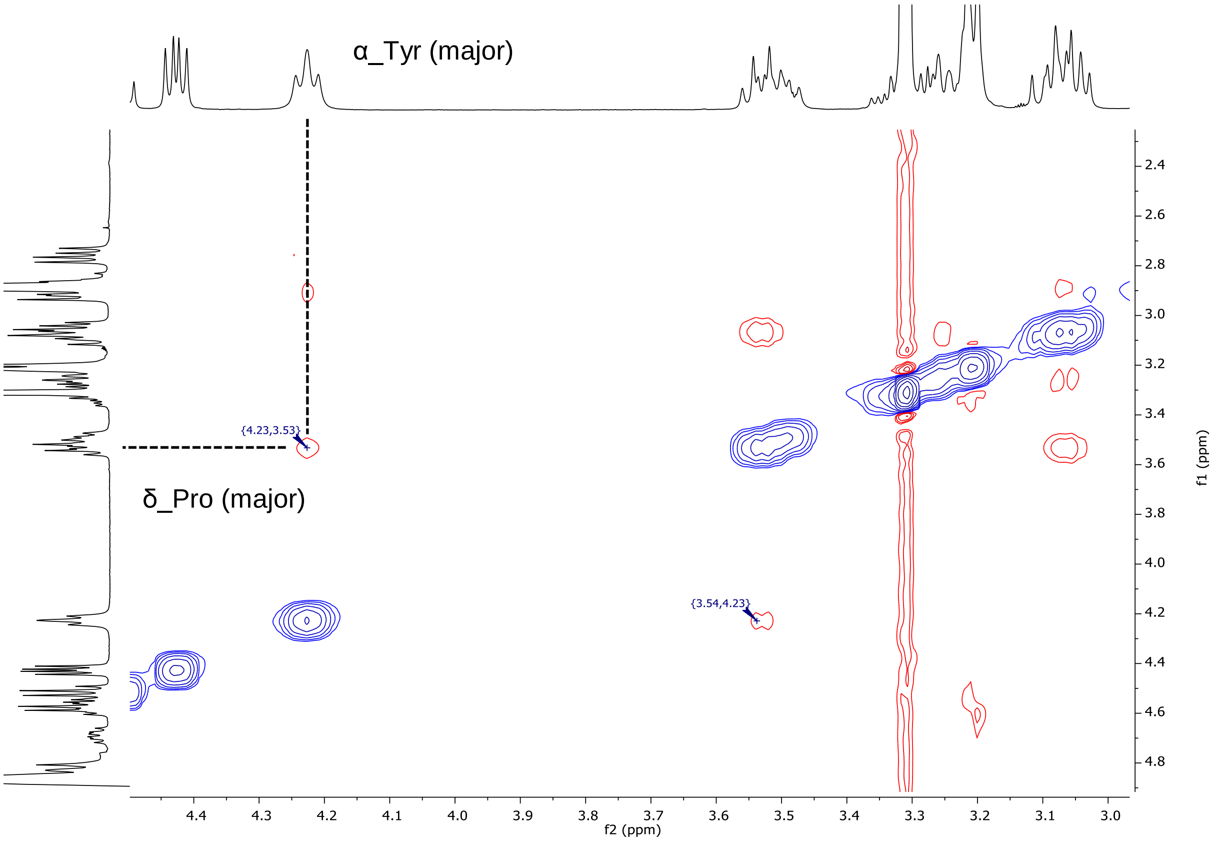

Figure S4: Determination of *trans*/*cis* conformation by NMR spectroscopy (major conformer gives a α_Tyr correlation with *δ*_Pro in ^{1}H–^{1}H NOESY in MeOD-d_3).

EM-1·TFA (TFA·H_2N-Tyr-Pro-Trp-Phe-NH_2)

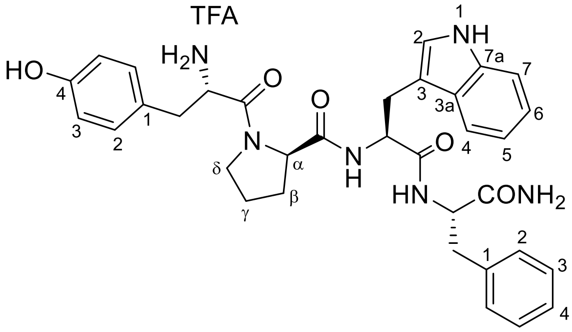

Prepared according to the general SPPS procedure from Rink amide AM resin (400 mg, 0.64 mmol/g, 0.256 mmol). The crude peptide was purified using semi-preparative reverse-phase HPLC (80% MQ H_2O + 0.1% TFA/20% MeCN + 0.1% TFA → 60% MeCN + 0.1% TFA, 20 min) and freeze dried.

Yield: 44.6 mg (0.062 mmol, 24%) of white solid; *trans*/*cis* = 66:34 (determination by ^{1}H–^{1}H NOESY in MeOD-*d₃*).

^{1}H NMR (400 MHz, MeOD-*d$_3$*) of the major conformer (*trans*): *δ* = 10.40–10.33 (m, 1H, NH-1_Trp), 7.88 (d, *J* = 6.7 Hz, 1H, NH_Trp), 7.66 (d, *J* = 7.9 Hz, 1H, NH_Phe), 7.60 (dd, *J* = 7.7, 1.3 Hz, 1H, CH-4_Trp), 7.30–7.27 (m, 1H, CH-7_Trp), 7.27–6.96 (m, 11H, CH-2_Tyr, OH_Tyr, CH-2_Trp, CH-5_Trp, CH-6_Trp, CH-2_Phe, CH-3_Phe, CH-4_Phe), 6.75 (d, *J* = 8.5 Hz, 2H, CH-3_Tyr), 4.61–4.55 (m, 1H, CH-α_Trp), 4.52 (dt, *J* = 7.9, 6.9 Hz, 1H, CH-α_Phe), 4.43 (dd, *J* = 8.3, 4.9 Hz, 1H, CH-α_Pro), 4.23 (t, *J* = 7.0 Hz, 1H, CH-α_Tyr), 3.57–3.46 (m, 1H, CH-δ_Pro), 3.21 (d, *J* = 6.4 Hz, 2H, CH_2-β_Trp), 3.12–3.02 (m, 1H, CH-δ_Pro), 2.95–2.85 (m, 3H, CH_2-β_Phe, CH-β_Tyr), 2.76 (dd, *J* = 14.4, 7.8 Hz, 1H, CH-β_Tyr), 2.04–1.93 (m, 1H, CH-β_Pro), 1.87–1.78 (m, 2H, CH_2-γ_Pro), 1.78–1.69 (m, 1H, CH-β_Pro).

^{13}C{^{1}H} NMR (101 MHz, MeOD-*d$_3$*) of the major conformer (*trans*): *δ* = 175.5 ($CONH_2$), 173.4 (Trp–CO–Phe), 173.3 (Pro–CO–Trp), 169.0 (Tyr–CO–Pro), 158.2 (C-4_Tyr), 138.1–138.0 (C-1_Phe, C-7a_Trp), 131.7 (C-2_Tyr), 130.2 (C-3_Phe), 129.3 (C-2_Phe), 128.7 (C-3a_Trp), 127.6 (C-4_Phe), 125.6 (C-1_Tyr), 125.0 (C-2_Trp), 122.5 (C-6_Trp), 119.9 (C-5_Trp), 119.2 (C-4_Trp), 116.7 (C-3_Tyr), 112.4 (C-7_Trp), 110.2 (C-3_Trp), 61.5 (α_Pro), 56.0 (α_Trp), 55.5 (α_Phe), 54.6 (α_Tyr), 48.2 (δ_Pro), 38.3 (β_Phe), 36.8 (β_Tyr), 29.8 (β_Pro), 28.3 (β_Trp), 25.8 (γ_Pro).

^{1}H NMR (400 MHz, MeOD-*d$_3$*) of the minor conformer (*cis*): *δ* = 10.47–10.43 (m, 1H, NH-1_Trp), 8.10 (d, *J* = 7.8 Hz, 1H, NH_Phe), 7.78 (d, *J* = 7.7 Hz, 1H, NH_Trp), 7.60 (dd, *J* = 7.7, 1.3 Hz, 1H, CH-4_Trp), 7.42–7.36 (m, 1H, CH-7_Trp), 7.27–6.96 (m, 9H, OH_Trp, CH-2_Trp, CH-5_Trp, CH-6_Trp, CH-2_Phe, CH-3_Phe, CH-4_Phe), 6.89 (d, *J* = 8.5 Hz, 2H, CH-2_Tyr), 6.70 (d, *J* = 8.5 Hz, 2H, CH-3_Tyr), 4.69 (ddd, *J* = 9.5, 7.8, 5.7 Hz, 1H, CH-α_Trp), 4.61–4.55 (m, 1H, CH-α_Phe), 3.57–3.46 (m, 1H, CH-α_Tyr), 3.38–3.33 (m, 1H, CH-δ_Pro), 3.30–3.22 (m, 3H, CH-α_Pro, CH-δ_Pro, CH-β_Trp), 3.12–3.02 (m, 2H, CH-β_Trp, CH-β_Phe), 2.95–2.85 (m, 3H, CH-β_Phe, CH_2-β_Tyr), 1.54 (ddd, *J* = 10.6, 7.5, 5.4 Hz, 2H, CH_2-β_Pro), 1.49–1.40 (m, 1H, CH-γ_Pro), 1.26–1.11 (m, 1H, CH-γ_Pro).

^{13}C{^{1}H} NMR (101 MHz, MeOD-*d$_3$*) of the minor conformer (*cis*): *δ* = 175.7 ($CONH_2$), 173.6 (Trp–CO–Phe), 173.1 (Pro–CO–Trp), 168.9 (Tyr–CO–Pro), 158.5 (C-4_Tyr), 138.1–138.0 (C-

1_Phe, C-7a_Trp), 131.3 (C-2_Tyr), 130.2 (C-3_Phe), 129.3 (C-2_Phe), 128.5 (C-3a_Trp), 127.6 (C-4_Phe), 125.2 (C-1_Tyr), 124.7 (C-2_Trp), 122.6 (C-6_Trp), 119.9 (C-5_Trp), 119.2 (C-4_Trp), 116.7 (C-3_Tyr), 112.4 (C-7_Trp), 110.8 (C-3_Trp), 61.1 (α_Pro), 55.7 (α_Trp), 55.6 (α_Phe), 54.6 (α_Tyr), 47.9 (δ_Pro), 38.7 (β_Phe), 38.0 (β_Tyr), 32.3 (β_Pro), 28.4 (β_Trp), 22.7 (γ_Pro).

HRMS (ESI) m/z: $[M + H]^+$ Calcd for $C_{34}H_{40}N_6O_5$ 611.2976; Found: 611.2978.

RP-HPLC analysis: >99% purity; *t*R = 8.06 min (20% → 60% MeCN + 0.1% TFA in MQ + 0.1% TFA, λ = 210 nm, 20 min).

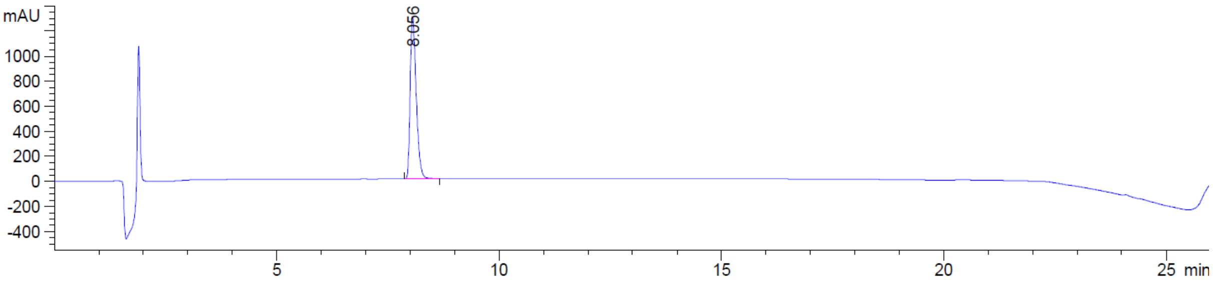

(CF₃S)Trp-EM-1·TFA (TFA·H₂N-Tyr-Pro-(CF₃S)Trp-Phe-NH₂)

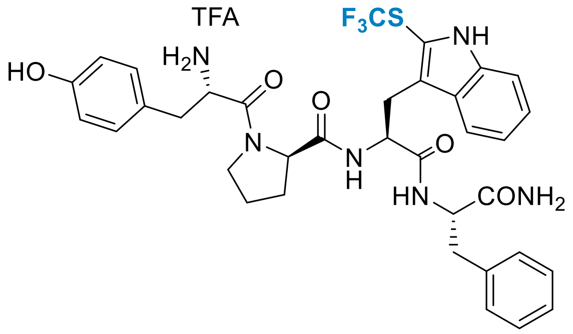

Prepared according to the general SPPS procedure from Rink amide AM resin (200 mg, 0.64 mmol/g, 0.128 mmol). The crude peptide was purified using semi-preparative reverse-phase HPLC (70% MQ H_2O + 0.1% TFA/30% MeCN + 0.1% TFA → 70% MeCN + 0.1% TFA, 20 min) and freeze dried.

Yield: 27.1 mg (0.033 mmol, 26%) of white solid; *trans*/*cis* = 71:29 (determination by ^1H–^1H ROESY in MeOD-*d₃*).

^1H NMR (400 MHz, MeOD-*d₃*) of the major conformer (*trans*): *δ* = 11.35 (s, 1H, NH-1_Trp), 8.02 (d, *J* = 7.0 Hz, 1H, NH_Trp), 7.86 (d, *J* = 8.1 Hz, 1H, NH_Phe), 7.73 (dd, *J* = 8.0, 0.9 Hz, 1H, CH-4_Trp), 7.35 (d, *J* = 8.2 Hz, 1H, CH-7_Trp), 7.31–6.86 (m, 10H, OH_Tyr, CH-2_Tyr, CH-5_Trp, CH-6_Trp, CH-2_Phe, CH-3_Phe, CH-4_Phe), 6.73 (d, *J* = 8.5 Hz, 2H, CH-3_Tyr), 4.65–4.57 (m, 1H, CH-α_Trp), 4.57–4.47 (m, 1H, CH-α_Phe), 4.41 (dd, *J* = 8.2, 4.7 Hz, 1H, CH-α_Pro), 4.23 (t, *J* = 7.1 Hz, 1H, CH-α_Tyr), 3.61–3.47 (m, 1H, CH-δ_Pro), 3.44–3.30 (m, 2H, CH_2-β_Trp), 3.13–2.96 (m, 3H, CH-δ_Pro, CH-β_Tyr, CH-β_Phe), 2.94–2.80 (m, 2H, CH-β_Tyr, CH-β_Phe), 1.97 (ddt, *J* = 12.0, 8.4, 6.5 Hz, 1H, CH-β_Pro), 1.87–1.79 (m, 2H, CH_2-γ_Pro), 1.76 (dt, *J* = 13.2, 5.1 Hz, 1H, CH-β_Pro).

$^{13}C\{^1H\}$ NMR (101 MHz, MeOD-*d₃*) of the major conformer (*trans*): *δ* = 175.2 ($CONH_2$), 173.1 (Pro–CO–Trp), 172.6 (Trp–CO–Phe), 168.9 (Tyr–CO–Pro), 158.2 (C-4_Tyr), 139.3 (C-

7a_Trp), 138.2 (C-1_Phe), 131.7 (C-2_Tyr), 130.2 (C-2_Phe), 130.1 (q, J = 310.4 Hz, CF_3S), 129.2 (C-3_Phe), 128.2 (C-3a_Trp), 127.5 (C-4_Phe), 125.6 (C-1_Tyr), 125.3 (C-6_Trp), 121.6 (C-3_Trp), 120.9 (C-5_Trp), 120.6 (C-4_Trp), 116.7 (C-3_Tyr), 115.9 (C-2_Trp), 112.7 (C-7_Trp), 61.4 (α_Pro), 55.8 (α_Trp), 54.7 (α_Tyr), 48.3 (δ_Pro), 38.7 (β_Phe), 37.0 (β_Tyr), 29.7 (β_Pro), 28.1 (β_Trp), 25.87 (γ_Pro).

^{1}H NMR (400 MHz, MeOD-*d_3*) of the minor conformer (*cis*): δ = 11.48 (s, 1H, NH-1_Trp), 8.07 (d, J = 7.9 Hz, 1H, NH_Phe), 7.85 (d, J = 8.1 Hz, 1H, NH_Trp), 7.69 (dd, J = 8.1, 1.0 Hz, 1H, CH-4_Trp), 7.41 (d, J = 8.3 Hz, 1H, CH-7_Trp), 7.33 (s, 1H, OH_Tyr), 7.31–6.86 (m, 9H, CH-2_Tyr, CH-5_Trp, CH-6_Trp, CH-2_Phe, CH-3_Phe, CH-4_Phe), 6.71 (d, J = 8.4 Hz, 2H, CH-3_Tyr), 4.77–4.68 (m, 1H, CH-α_Trp), 4.57–4.47 (m, 1H, CH-α_Phe), 3.61–3.47 (m, 1H, CH-α_Tyr), 3.44–3.30 (m, 4H, CH-δ_Pro, CH_2-β_Trp, CH-α_Pro), 3.28–3.20 (m, 1H, CH-δ_Pro), 3.13–2.96 (m, 2H, CH-β_Tyr, CH-β_Phe), 2.94–2.80 (m, 2H, CH-β_Tyr, CH-β_Phe), 1.57–1.42 (m, 3H, CH-γ_Pro, CH_2-β_Pro), 1.23–1.12 (m, 1H, CH-γ_Pro).

^{13}C{^{1}H} NMR (101 MHz, MeOD-*d_3*) of the minor conformer (*cis*): δ = 175.3 ($CONH_2$), 172.9 (Pro–CO–Trp), 172.8 (Trp–CO–Phe), 168.9 (Tyr–CO–Pro), 158.5 (C-4_Tyr), 139.3 (C-7a_Trp), 138.1 (C-1_Phe), 131.3 (C-1_Tyr), 130.2 (C-2_Phe), 129.3 (C-3_Phe), 128.2 (C-3a_Trp), 127.6 (C-4_Phe), 125.4 (C-1_Tyr), 125.2 (C-6_Trp), 121.5 (C-3_Trp), 121.0 (C-5_Trp), 120.5 (C-4_Trp), 116.7 (C-3_Tyr), 115.7 (C-2_Trp), 112.8 (C-7_Trp), 61.1 (α_Pro), 55.7 (α_Phe), 55.6 (α_Phe), 54.6 (α_Tyr), 47.9 (δ_Pro), 38.8 (β_Phe), 38.1 (β_Tyr), 32.3 (β_Pro), 27.8 (β_Trp), 22.7 (γ_Pro).

CF_3S quartet resonance of the minor conformer could not be detected.

^{19}F NMR (376 MHz, MeOD-*d_3*): δ = –44.93 (CF_3S, 3F, minor), –45.08 (CF_3S, 3F, major), –77.07 (TFA, 3F).

HRMS (ESI) m/z: $[M + H]^+$ Calcd for $C_{35}H_{38}F_3N_6O_5S$ 711.2571; Found: 711.2585.

RP-HPLC analysis: >99% purity; *t*R = 10.94 min (20% → 60% MeCN + 0.1% TFA in MQ + 0.1% TFA, λ = 210 nm, 20 min).

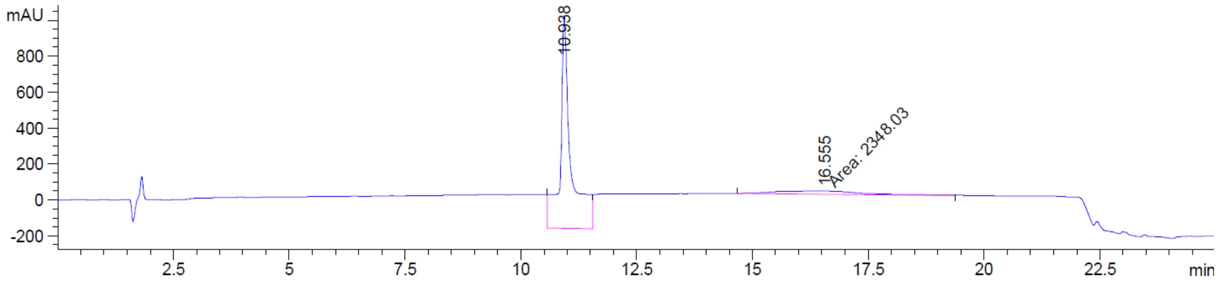

(CF$_3$S)Tyr-EM-1·TFA (TFA·H$_2$N-(CF$_3$S)Tyr-Pro-Trp-Phe-NH$_2$)

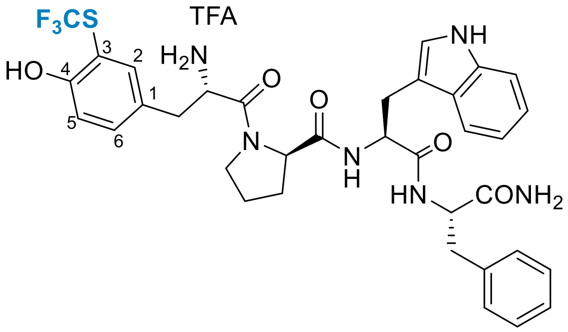

Prepared according to the general SPPS procedure from Rink amide AM resin (300 mg, 0.64 mmol/g, 0.192 mmol). The crude peptide was purified using semi-preparative reverse-phase HPLC (70% MQ H_2O + 0.1% TFA/30% MeCN + 0.1% TFA → 70% MeCN + 0.1% TFA, 20 min) and freeze dried.

Yield: 29.8 mg (0.036 mmol, 19%) of white solid; *trans*/*cis* = 71:29 (determination by ^{1}H–^{1}H NOESY in MeOD-*d$_3$*).

^{1}H NMR (400 MHz, MeOD-*d$_3$*) of the major conformer (*trans*): δ = 10.38 (d, J = 2.3 Hz, 1H, NH-1_Trp), 7.93 (d, J = 6.7 Hz, 1H, NH_Trp), 7.66 (d, J = 7.9 Hz, 1H, NH_Phe), 7.61 (d, J = 7.5 Hz, 1H, CH-4_Trp), 7.48 (d, J = 2.3 Hz, 1H, CH-2_Tyr), 7.27 (dd, J = 8.1, 0.9 Hz, 1H, CH-7_Trp), 7.25–6.95 (m, 10H, OH_Tyr, CH-6_Tyr, CH-2_Trp, CH-5_Trp, CH-6_Trp, CH-2_Phe, CH-3_Phe, CH-4_Phe), 6.90 (d, J = 8.4 Hz, 1H, CH-5_Tyr), 4.64–4.55 (m, 1H, CH-α_Trp), 4.55–4.49 (m, 1H, CH-α_Phe), 4.43 (dd, J = 8.2, 4.9 Hz, 1H, CH-α_Pro), 4.27 (t, J = 6.9 Hz, 1H, CH-α_Tyr), 3.63–3.50 (m, 1H, CH-δ_Pro), 3.29–3.14 (m, 2H, CH$_2$-β_Trp), 3.14–3.00 (m, 1H, CH-δ_Pro), 2.96–2.85 (m, 3H, CH$_2$-β_Phe, CH-β_Tyr), 2.79 (dd, J = 14.5, 7.6 Hz, 1H, CH-β_Tyr), 2.04–1.94 (m, 1H, CH-β_Pro), 1.88–1.79 (m, 2H, CH$_2$-γ_Pro), 1.79–1.70 (m, 1H, CH-β_Pro).

^{13}C{^{1}H} NMR (101 MHz, MeOD-*d$_3$*) of the major conformer (*trans*): δ = 175.5 (CONH$_2$), 173.4 (Trp–CO–Phe), 173.4 (Pro–CO–Trp), 168.7 (Tyr–CO–Pro), 160.7 (C-4_Tyr), 140.68 (C-2_Tyr), 138.0 (C-7a_Trp), 138.0 (C-1_Phe), 135.8 (C-6_Tyr), 131.1 (q, J = 308.0 Hz, CF$_3$S), 130.2 (C-2_Phe), 129.3 (C-3_Phe), 128.7 (C-3a_Trp), 127.6 (C-4_Phe), 126.7 (C-1_Tyr), 125.0 (C-2_Trp), 122.4 (C-6_Trp), 119.9 (C-5_Trp), 119.2 (C-4_Trp), 118.0 (C-5_Tyr), 112.4 (C-7_Trp), 110.8 (C-3_Tyr), 110.2 (C-3_Trp), 61.5 (α_Pro), 56.0 (α_Trp), 55.4 (α_Phe), 54.2 (α_Tyr), 48.3 (δ_Pro), 38.3 (β_Phe), 36.2 (β_Tyr), 29.8 (β_Pro), 28.4 (β_Trp), 25.8 (γ_Pro).

^{1}H NMR (400 MHz, MeOD-*d$_3$*) of the minor conformer (*cis*): δ = 10.46 (s, 1H, NH-1_Trp), 8.15 (d, J = 7.9 Hz, 1H, NH_Trp), 7.80 (d, J = 7.7 Hz, 1H, NH_Phe), 7.61 (d, J = 7.5 Hz, 1H, CH-4_Trp), 7.40–7.35 (m, 2H, OH_Tyr, CH-7_Trp), 7.30 (d, J = 2.3 Hz, 1H, CH-2_Tyr), 7.25–6.95 (m, 9H, CH-6_Tyr, CH-2_Trp, CH-5_Trp, CH-6_Trp, CH-2_Phe, CH-3_Phe, CH-4_Phe), 6.90 (d, J = 8.4 Hz, 1H, CH-5_Tyr), 4.74–4.66 (m, 1H, CH-α_Trp), 4.64–4.55 (m, 1H, CH-α_Phe), 3.63–3.50 (m, 1H, CH-α_Tyr), 3.45 (dd, J = 7.2, 4.2 Hz, 1H, CH-α_Pro), 3.38–3.32 (m, 1H, CH-δ_Pro), 3.29–3.14 (m, 2H, CH-δ_Pro, CH-β_Trp), 3.14–3.00 (m, 2H, CH-β_Trp, CH-β_Phe), 2.96–2.85 (m, 3H, CH$_2$-β_Tyr, CH-β_Phe), 1.59–1.49 (m, 2H, CH$_2$-β_Pro), 1.48–1.37 (m, 1H, CH-γ_Pro), 1.26–1.12 (m, 1H, CH-γ_Pro).

$^{13}C\{^1H\}$ NMR (101 MHz, MeOD-d_3) of the minor conformer (*cis*): δ = 175.7 ($CONH_2$), 173.6 (Pro–CO–Trp), 172.9 (Trp–CO–Phe), 168.6 (Tyr–CO–Pro), 160.8 (C-4_Tyr), 140.1 (C-2_Tyr), 138.1 (C-7a_Trp), 138.0 (C-1_Phe), 135.2 (C-6_Tyr), 130.2 (C-2_Phe), 129.3 (C-3_Phe), 128.5 (C-3a_Trp), 127.6 (C-4_Phe), 126.4 (C-1_Tyr), 124.7 (C-2_Trp), 122.6 (C-6_Trp), 119.9 (C-5_Trp), 119.2 (C-4_Trp), 118.0 (C-5_Tyr), 112.5 (C-7_Trp), 111.0 (C-3_Tyr), 110.7 (C-3_Trp), 61.3 (α_Pro), 55.8 (α_Trp), 55.8 (α_Phe), 54.1 (α_Tyr), 47.8 (δ_Pro), 38.7 (β_Phe), 37.3 (β_Tyr), 32.3 (β_Pro), 28.5 (β_Trp), 22.6 (γ_Pro).

CF_3S quartet resonance of the minor conformer could not be detected.

^{19}F NMR (376 MHz, MeOD-d_3): δ = –44.25 (CF_3S, 3F, minor), –44.30 (CF_3S, 3F, major), –77.13 (TFA, 3F).

HRMS (ESI) m/z: $[M + H]^+$ Calcd for $C_{35}H_{38}F_3N_6O_5S$ 711.2571; Found: 711.2572.

RP-HPLC analysis: >99% purity; *t*R = 11.59 min (20% → 60% MeCN + 0.1% TFA in MQ + 0.1% TFA, λ = 210 nm, 20 min).

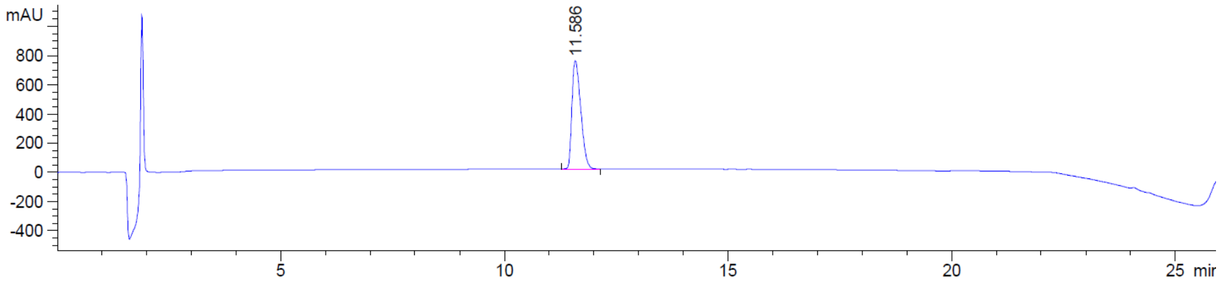

5. Enantiopurity analysis

***rac*-4b**: Chiralpak IA-3 column; flow rate of 1.0 mL/min; mobile phase: 15% *i*PrOH + 0.1% TFA in *n*-hexane; detection at 254 nm. Prepared from **(*S*)-4b** and **(*R*)-4b**.

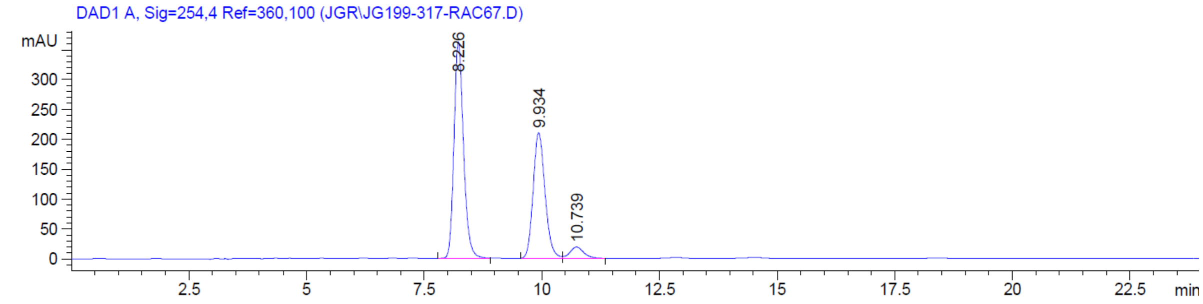

Signal 1: DAD1 A, Sig=254,4 Ref=360,100

Peak #	RetTime [min]	Type	Width [min]	Area [mAU*s]	Height [mAU]	Area %
1	8.226	BB	0.2166	5113.82227	362.46671	55.5935
2	9.934	BV	0.2688	3688.25635	210.11105	40.0958
3	10.739	VB	0.3144	396.52255	19.08476	4.3107

(*S*)-4b: Chiralpak IA-3 column; flow rate of 1.0 mL/min; mobile phase: 15% *i*PrOH + 0.1% TFA in *n*-hexane; detection at 254 nm. *t*R = 9.96 min, 98% *ee*.

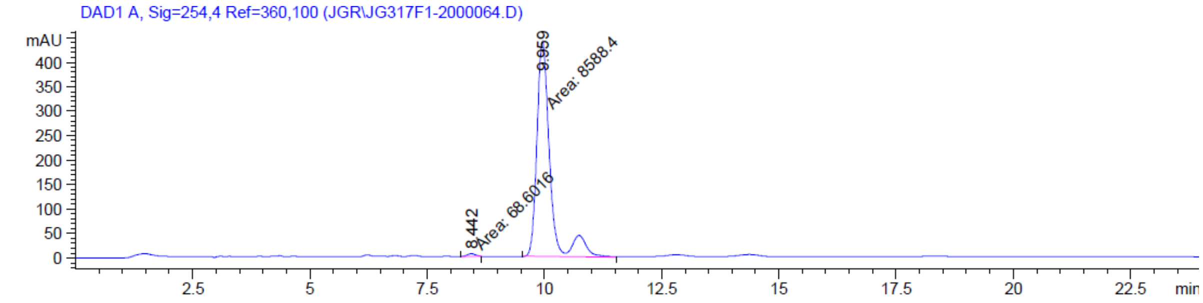

Signal 1: DAD1 A, Sig=254,4 Ref=360,100

Peak #	RetTime [min]	Type	Width [min]	Area [mAU*s]	Height [mAU]	Area %
1	8.442	MM	0.1956	68.60157	5.84628	0.7924
2	9.959	MM	0.3264	8588.40332	438.57419	99.2076

(*R*)-4b: Chiralpak IA-3 column; flow rate of 1.0 mL/min; mobile phase: 15% *i*PrOH + 0.1% TFA in *n*-hexane; detection at 254 nm. *t*R = 8.29 min, >99% *ee*.

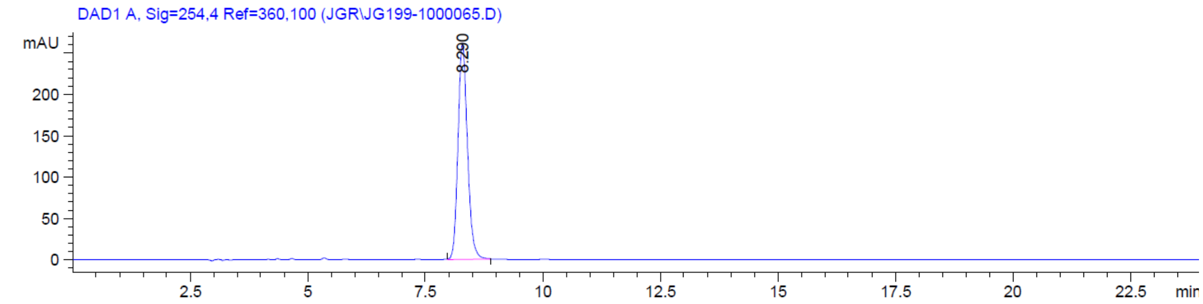

Signal 1: DAD1 A, Sig=254,4 Ref=360,100

Peak #	RetTime [min]	Type	Width [min]	Area [mAU*s]	Height [mAU]	Area %
1	8.290	BB	0.2154	3654.18091	260.91183	100.0000

***rac*-6a**: Chiralpak IA-3 column; flow rate of 1.0 mL/min; mobile phase: 10 → 20% *i*PrOH + 0.1% TFA in *n*-hexane in 20 min; detection at 254 nm. Prepared from **(*S*)-6a** and **(*R*)-6a**.

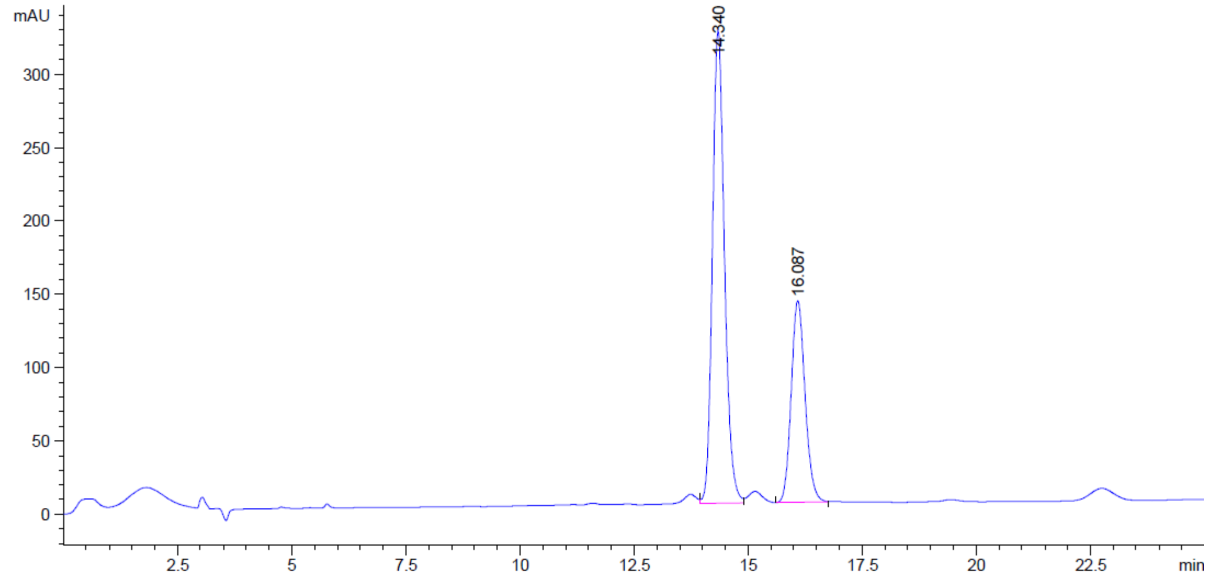

Peak #	RetTime [min]	Type	Width [min]	Area [mAU*s]	Height [mAU]	Area %
1	14.340	VV	0.2804	5926.41797	322.54355	67.1919
2	16.087	VB	0.3241	2893.71411	137.20050	32.8081

(*S*)-6a: Chiralpak IA-3 column; flow rate of 1.0 mL/min; mobile phase: 10 → 20% *i*PrOH + 0.1% TFA in *n*-hexane in 20 min; detection at 254 nm. *t*R = 16.01 min, 99% *ee*.

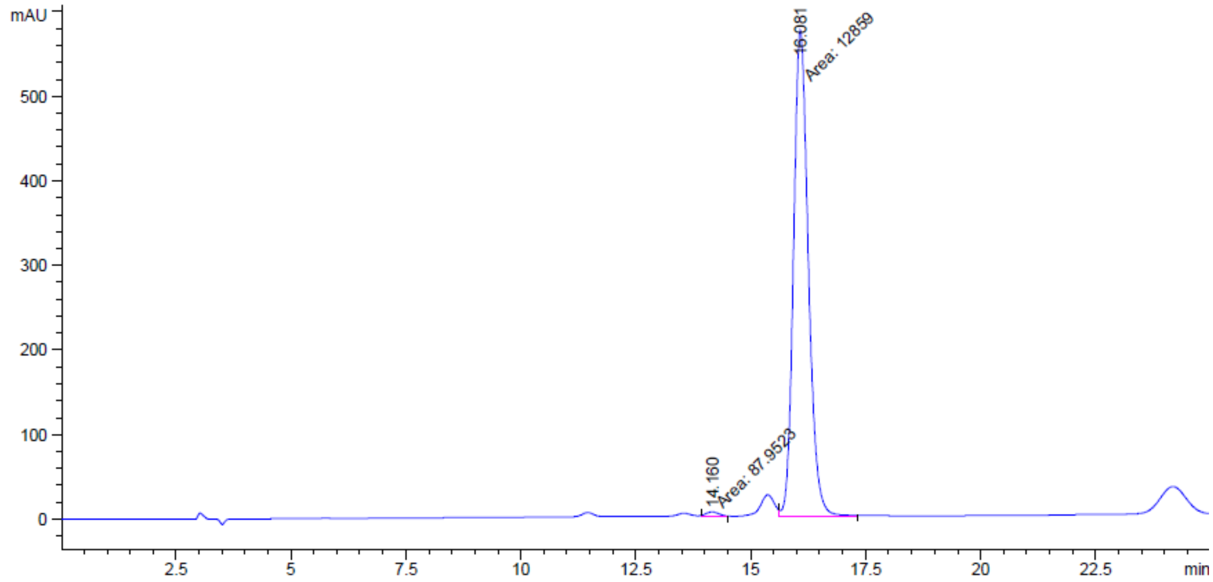

Signal 1: DAD1 A, Sig=254,4 Ref=360,100

Peak #	RetTime [min]	Type	Width [min]	Area [mAU*s]	Height [mAU]	Area %
1	14.160	MM	0.3006	87.95234	4.87695	0.6793
2	16.081	MM	0.3732	1.28590e4	574.23077	99.3207

(*R*)-6a: Chiralpak IA-3 column; flow rate of 1.0 mL/min; mobile phase: 10 → 20% *i*PrOH + 0.1% TFA in *n*-hexane in 20 min; detection at 254 nm. *t*R = 14.27 min, >99% *ee*.

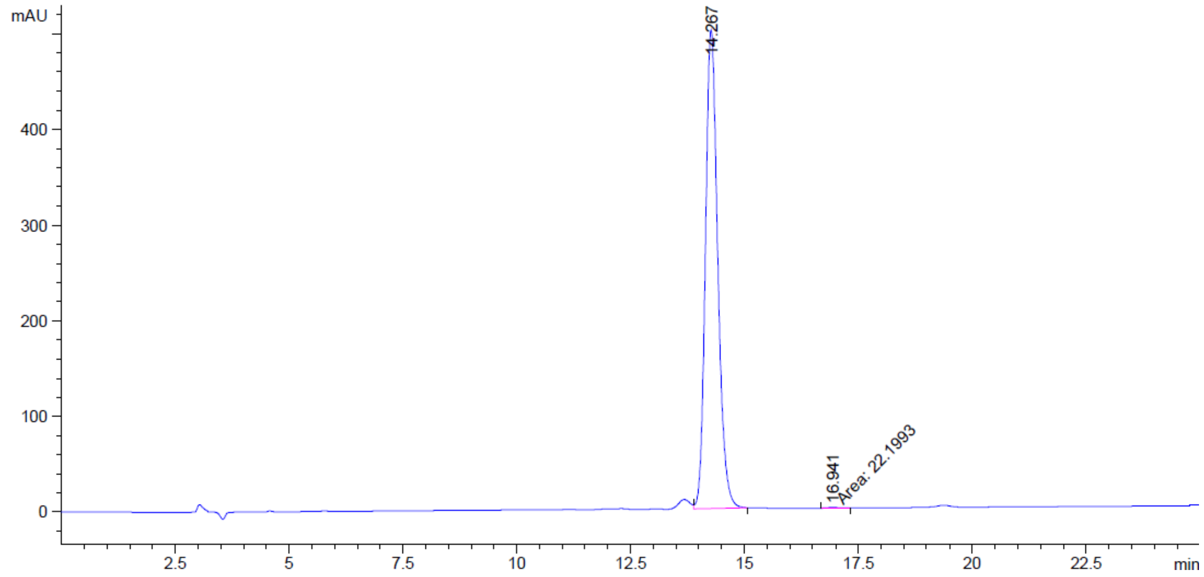

Signal 1: DAD1 A, Sig=254,4 Ref=360,100

Peak #	RetTime [min]	Type	Width [min]	Area [mAU*s]	Height [mAU]	Area %
1	14.267	VB	0.2895	9416.30273	500.42062	99.7648
2	16.941	MM	0.4328	22.19928	8.54919e-1	0.2352

6. Hydrophobicity index determination

The peptides were prepared according to the SPPS general procedure.

The chromatographic hydrophobicity index (CHI) was determined according to the protocols described previously.[17] Experiments were performed at pH 7 using 50 mM ammonium acetate in mQ H_2O and MeCN as mobile phase. Peptides **9-12** were dissolved in 50 mM ammonium acetate before injection (25 µL injection volume). Retention factors (k') were determined at λ = 254 nm at various MeCN concentrations ranging from 6–22% (k' = (t*R*–t*0*) / t*R*). Then, log k' was expressed as a function of MeCN content and linear regression was applied to four values per peptide. The intercept log k'_w at MeCN% = 0 and the slopes S were extracted from the linear fit for each peptide (correlation coefficient: $R^2 > 0.99$). The isocratic hydrophobicity index (CHI) was calcd for each peptide using the following equation:

$$\phi_0 = -\log k'_w / S$$

TFA·H$_2$N-L-Ala-L-Tyr-L-Leu-OH (9)

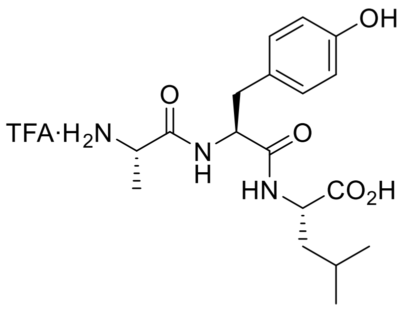

Prepared according to the general SPPS procedure from Fmoc-L-leucine preloaded Wang resin (400 mg, 0.80 mmol/g, 0.32 mmol). The crude peptide was purified using semi-preparative reverse-phase HPLC (80% MQ H_2O + 0.1% TFA/20% MeCN + 0.1% TFA → 50% MeCN + 0.1% TFA, 15 min) and freeze dried.

Yield: 61.5 mg (0.128 mmol, 40%) of white solid.

^{1}H NMR (400 MHz, MeOD-*d$_3$*): *δ* = 8.41 (d, *J* = 8.0 Hz, 1H), 8.30 (d, *J* = 8.1 Hz, 1H), 7.10 (d, *J* = 8.5 Hz, 2H), 6.68 (d, *J* = 8.5 Hz, 2H), 4.63 (ddd, *J* = 9.8, 7.9, 4.8 Hz, 1H), 4.45–4.36 (m, 1H), 3.83 (q, *J* = 7.0 Hz, 1H), 3.11 (dd, *J* = 14.3, 4.8 Hz, 1H), 2.79 (dd, *J* = 14.2, 9.8 Hz, 1H), 1.76–1.57 (m, 3H), 1.46 (d, *J* = 7.0 Hz, 3H), 0.95 (d, *J* = 6.3 Hz, 3H), 0.91 (d, *J* = 6.2 Hz, 3H).

^{13}C{^{1}H} NMR (101 MHz, MeOD-*d$_3$*): *δ* = 176.2, 173.3, 170.9, 157.3, 131.1, 128.8, 116.2, 56.4, 52.5, 50.1, 41.8, 37.8, 25.8, 23.3, 21.8, 17.7.

HRMS (ESI) m/z: $[M + H]^+$ Calcd for $C_{18}H_{28}N_3O_5$ 366.2023; Found: 366.2032.

RP-HPLC analysis: >95% purity; *t*R = 10.42 min (7% MeCN in ammonium acetate buffer (pH = 7), λ = 210 nm, 20 min).

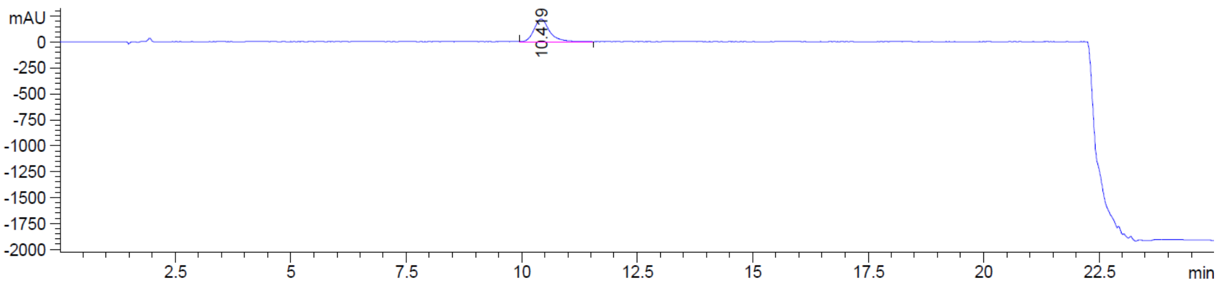

TFA·H₂N-L-Ala-L-Trp-L-Leu-OH (10)

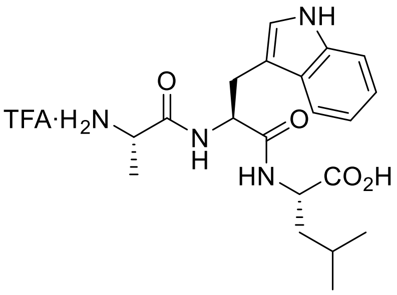

Prepared according to the general SPPS procedure from Fmoc-L-leucine preloaded Wang resin (400 mg, 0.80 mmol/g, 0.32 mmol). The crude peptide was purified using semi-preparative reverse-phase HPLC (80% MQ H_2O + 0.1% TFA/20% MeCN + 0.1% TFA → 50% MeCN + 0.1% TFA, 20 min) and freeze dried.

Yield: 8.8 mg (0.017 mmol, 5%) of white solid.

^{1}H NMR (400 MHz, MeOD-*d₃*): δ = 10.37–10.29 (m, 1H), 8.42–8.33 (m, 2H), 7.66 (dd, *J* = 7.8, 1.0 Hz, 1H), 7.31 (dd, *J* = 8.0, 0.9 Hz, 1H), 7.15 (d, *J* = 2.4 Hz, 1H), 7.08 (ddd, *J* = 8.1, 7.0, 1.2 Hz, 1H), 7.01 (ddd, *J* = 8.0, 7.0, 1.1 Hz, 1H), 4.80 (ddd, *J* = 9.1, 7.8, 5.2 Hz, 1H), 4.44 (td, *J* = 8.0, 6.7 Hz, 1H), 3.81 (q, *J* = 7.0 Hz, 1H), 3.36–3.31 (m, 1H), 3.10 (dd, *J* = 14.9, 9.0 Hz, 1H), 1.73–1.59 (m, 3H), 1.45 (d, *J* = 7.0 Hz, 3H), 0.94 (d, *J* = 6.3 Hz, 3H), 0.90 (d, *J* = 6.1 Hz, 3H).

^{13}C{^{1}H} NMR (101 MHz, MeOD-*d₃*): δ = 175.8, 173.7, 170.8, 138.1, 128.6, 124.7, 122.3, 119.7, 119.2, 112.2, 110.7, 55.3, 52.1, 50.2, 41.6, 28.8, 25.8, 23.3, 21.7, 17.7.

HRMS (ESI) m/z: $[M + H]^+$ Calcd for $C_{20}H_{29}N_4O_4$ 389.2183; Found: 389.2189.

RP-HPLC analysis: >95% purity; *t*R = 12.06 min (13% MeCN in ammonium acetate buffer (pH = 7), λ = 210 nm, 20 min).

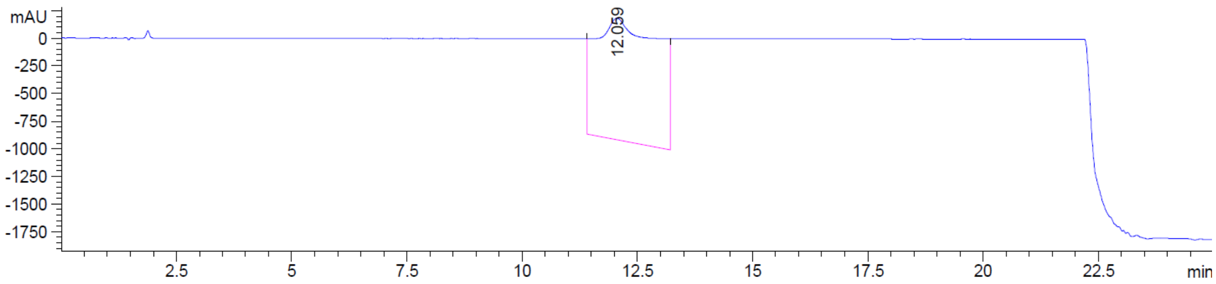

TFA·H$_2$N-L-Ala-L-(CF$_3$S)Tyr-L-Leu-OH (11)

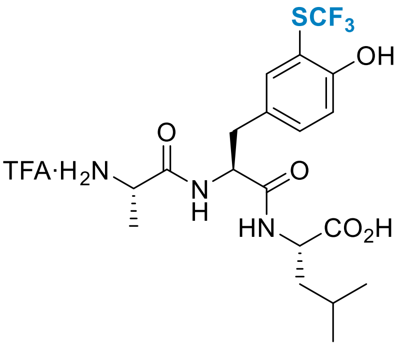

Prepared according to the general SPPS procedure from Fmoc-L-leucine preloaded Wang resin (300 mg, 0.80 mmol/g, 0.24 mmol). The crude peptide was purified using semi-preparative reverse-phase HPLC (80% MQ H_2O + 0.1% TFA/30% MeCN + 0.1% TFA → 50% MeCN + 0.1% TFA, 20 min) and freeze dried.

Yield: 46.3 mg (0.080 mmol, 33%) of white solid.

^{1}H NMR (400 MHz, MeOD-*d$_3$*): δ = 8.48 (d, J = 7.8 Hz, 1H), 8.47 (d, J = 7.8 Hz, 1H), 7.50 (d, J = 2.2 Hz, 1H), 7.31 (dd, J = 8.4, 2.3 Hz, 1H), 6.87 (d, J = 8.4 Hz, 1H), 4.64 (ddd, J = 9.5, 8.0, 4.9 Hz, 1H), 4.41 (td, J = 8.0, 6.0 Hz, 1H), 3.82 (q, J = 7.0 Hz, 1H), 3.13 (dd, J = 14.2, 4.9 Hz, 1H), 2.82 (dd, J = 14.3, 9.6 Hz, 1H), 1.76–1.58 (m, 3H), 1.47 (d, J = 7.0 Hz, 3H), 0.95 (d, J = 6.3 Hz, 3H), 0.91 (d, J = 6.2 Hz, 3H).

^{13}C{^{1}H} NMR (101 MHz, MeOD-*d$_3$*): δ = 175.7, 173.1, 170.9, 159.9, 140.2, 135.2, 131.1 (q, J = 307.8 Hz), 130.0, 117.4, 110.2 (q, J = 1.9 Hz), 56.1, 52.1, 50.1, 41.6, 37.4, 25.8, 23.3, 21.7, 17.7.

^{19}F NMR (376 MHz, MeOD-*d$_3$*): δ = –44.48 (CF_3S, 3F), –76.99 (TFA, 3F).

HRMS (ESI) m/z: $[M + H]^+$ Calcd for $C_{19}H_{27}F_3N_3O_5S$ 466.1618; Found: 466.1628.

RP-HPLC analysis: 96% purity; *t*R = 11.68 min (17% MeCN in ammonium acetate buffer (pH = 7), λ = 210 nm, 20 min).

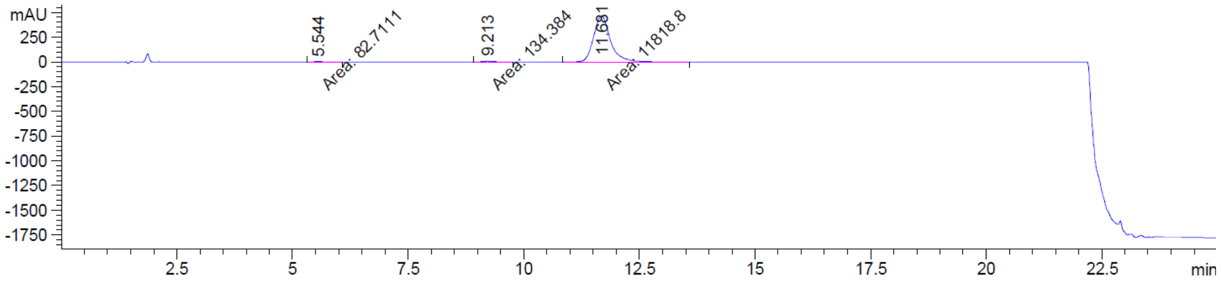

TFA·H$_2$N-L-Ala-L-(CF$_3$S)Trp-L-Leu-OH (12)

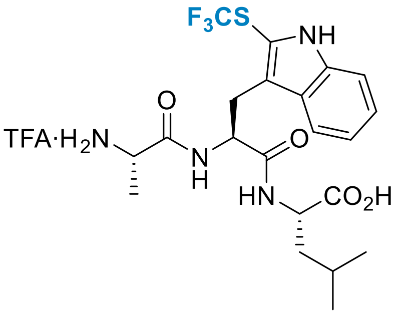

Prepared according to the general SPPS procedure from Fmoc-L-leucine preloaded Wang resin (300 mg, 0.80 mmol/g, 0.24 mmol). The crude peptide was purified using semi-preparative reverse-phase HPLC (80% MQ H_2O + 0.1% TFA/20% MeCN + 0.1% TFA → 50% MeCN + 0.1% TFA, 20 min) and freeze dried.

Yield: 11.4 mg (0.018 mmol, 8%) of white solid.

^{1}H NMR (400 MHz, MeOD-*d*$_3$): δ = 11.35 (s, 1H), 8.40 (d, J = 7.9 Hz, 1H), 8.19 (d, J = 8.2 Hz, 1H), 7.78 (dd, J = 8.1, 1.0 Hz, 1H), 7.37 (d, J = 8.3 Hz, 1H), 7.23 (ddd, J = 8.2, 7.0, 1.1 Hz, 1H), 7.09 (ddd, J = 8.0, 7.0, 1.0 Hz, 1H), 4.84 (d, J = 15.3 Hz, 1H), 4.37 (td, J = 8.2, 5.8 Hz, 1H), 3.81 (q, J = 7.0 Hz, 1H), 3.46 (dd, J = 14.3, 7.2 Hz, 1H), 3.35–3.25 (m, 1H), 1.66–1.50 (m, 3H), 1.44 (d, J = 7.0 Hz, 3H), 0.90 (d, J = 6.2 Hz, 3H), 0.87 (d, J = 6.1 Hz, 3H).

^{13}C{^{1}H} NMR (101 MHz, MeOD-*d*$_3$): δ = 175.4, 172.8, 170.7, 139.4, 130.2 (q, J = 310.5 Hz), 128.2, 125.2, 121.4, 120.8, 120.7, 115.9, 112.6, 55.2, 52.1, 50.2, 41.9, 28.5, 25.7, 23.2, 21.8, 17.6.

^{19}F NMR (376 MHz, MeOD-*d*$_3$): δ = –45.15 (CF_3S, 3F), –76.96 (TFA, 3F).

HRMS (ESI) m/z: $[M + H]^+$ Calcd for $C_{21}H_{28}F_3N_4O_4S$ 489.1778; Found: 489.1788.

RP-HPLC analysis: >95% purity; t*R* = 18.36 min (19% MeCN in ammonium acetate buffer (pH = 7), λ = 210 nm, 20 min).

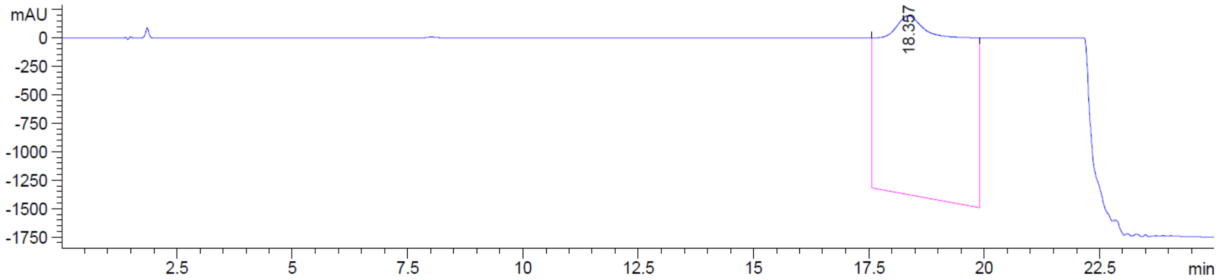

Table of retention times (t*R*), dead times (t*0*), and calculated log k' and CHI for peptides **9-12** at pH 7:

	TFA·H_2N-Ala-Tyr-Leu-OH (**9**)				TFA·H_2N-Ala-Trp-Leu-OH (**10**)				TFA·H_2N-Ala-(CF_3S)Tyr-Leu-OH (**11**)				TFA·H_2N-Ala-(CF_3S)Trp-Leu-OH (**12**)			
**MeCN (%)**	***t*R**	**t_0**	**k'**	**log(k')**	***t*R**	**t_0**	**k'**	**log(k')**	***t*R**	**t_0**	**k'**	**log(k')**	***t*R**	**t_0**	**k'**	**log(k')**
6	14.389	1.936	6.432	0.808												
7	10.420	1.918	4.433	0.647												
8	7.933	1.904	3.166	0.501												
9	6.204	1.889	2.284	0.359												
12					15.635	1.862	7.397	0.869								
13					12.058	1.853	5.507	0.741								
14					9.609	1.852	4.188	0.622								
15					7.736	1.846	3.191	0.504	18.845	1.848	9.198	0.964				
17									11.681	1.839	5.352	0.729				
18									9.342	1.835	4.091	0.612				
19									7.589	1.829	3.149	0.498	18.356	1.830	9.031	0.956
20													14.315	1.825	6.844	0.835
21													11.304	1.820	5.211	0.717
22													9.120	1.815	4.025	0.605
**CHI**	**11.37**				**19.13**				**23.27**				**27.15**			

Peptide **9** (6% MeCN in 50 mM ammonium acetate buffer, pH 7, 1 mL/min):

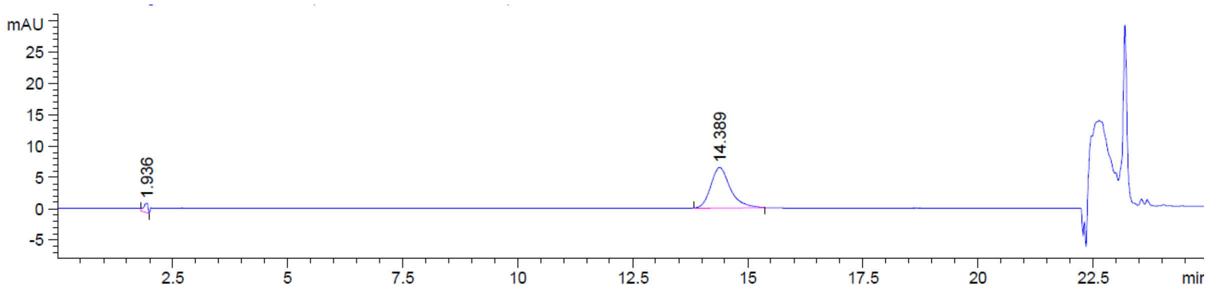

Peptide **9** (7% MeCN in 50 mM ammonium acetate buffer, pH 7, 1 mL/min):

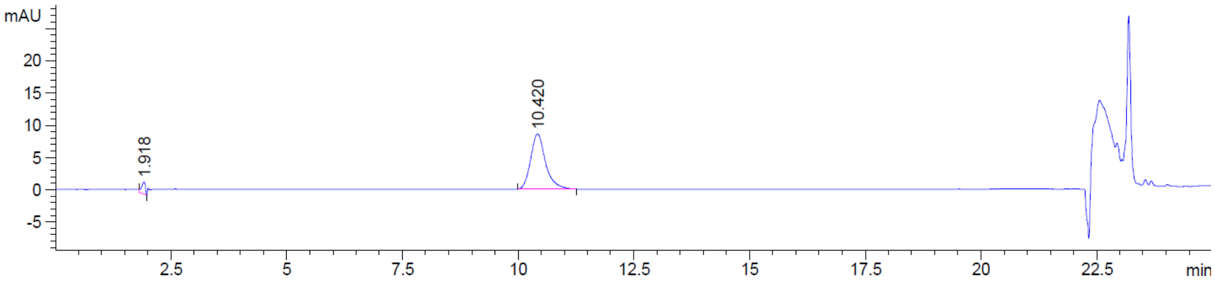

Peptide **9** (8% MeCN in 50 mM ammonium acetate buffer, pH 7, 1 mL/min):

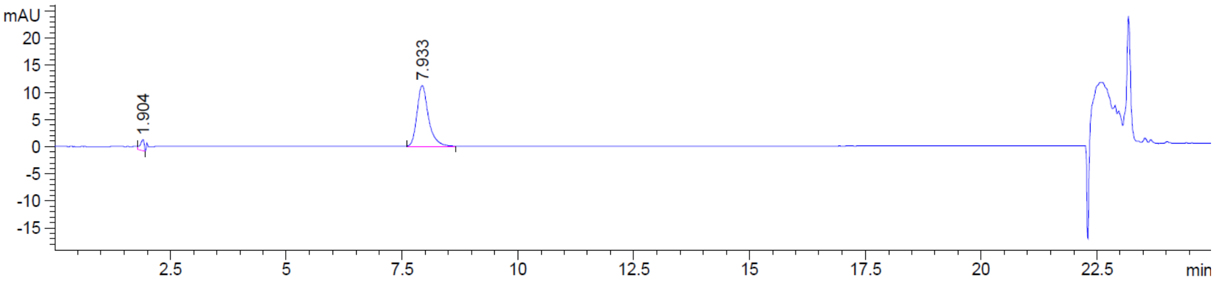

Peptide **9** (9% MeCN in 50 mM ammonium acetate buffer, pH 7, 1 mL/min):

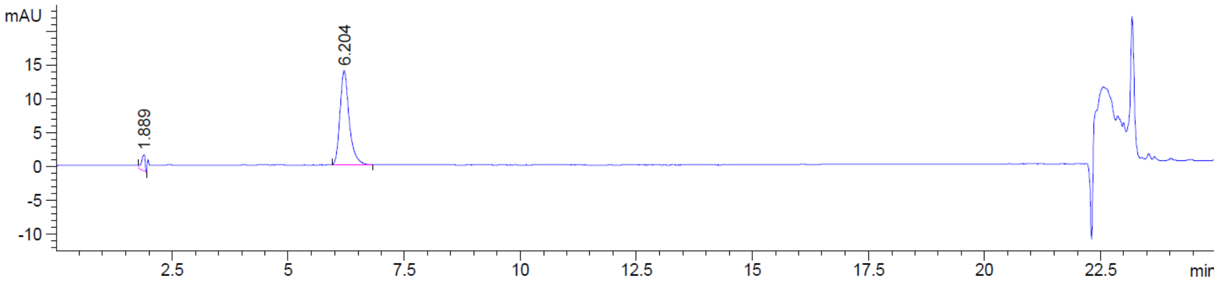

Peptide **10** (12% MeCN in 50 mM ammonium acetate buffer, pH 7, 1 mL/min):

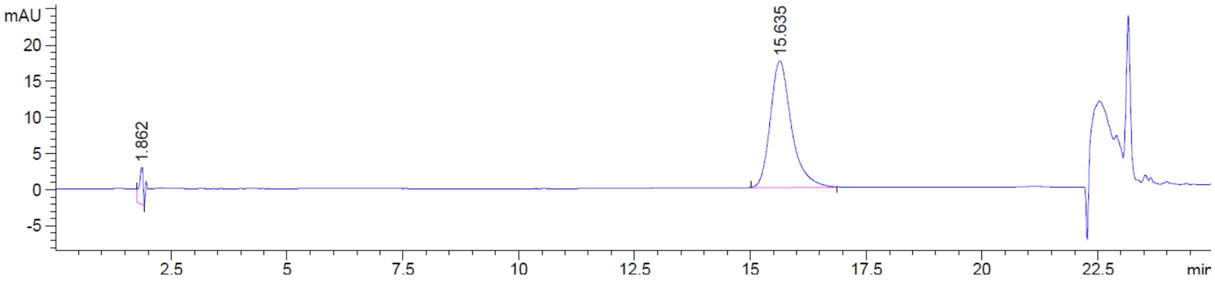

Peptide **10** (13% MeCN in 50 mM ammonium acetate buffer, pH 7, 1 mL/min):

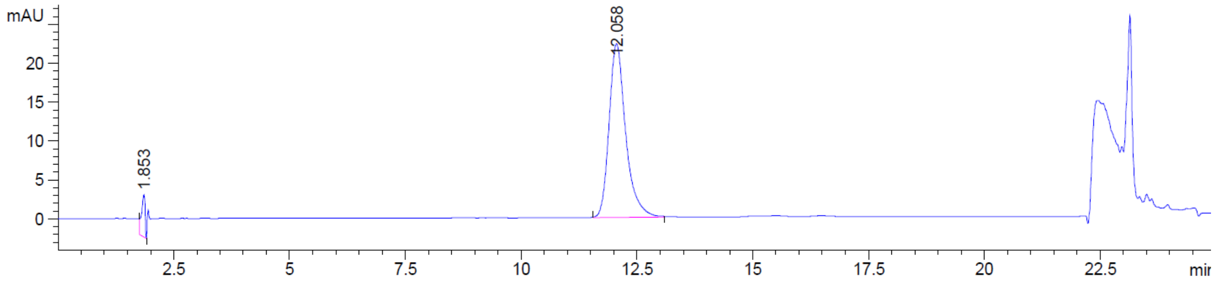

Peptide **10** (14% MeCN in 50 mM ammonium acetate buffer, pH 7, 1 mL/min):

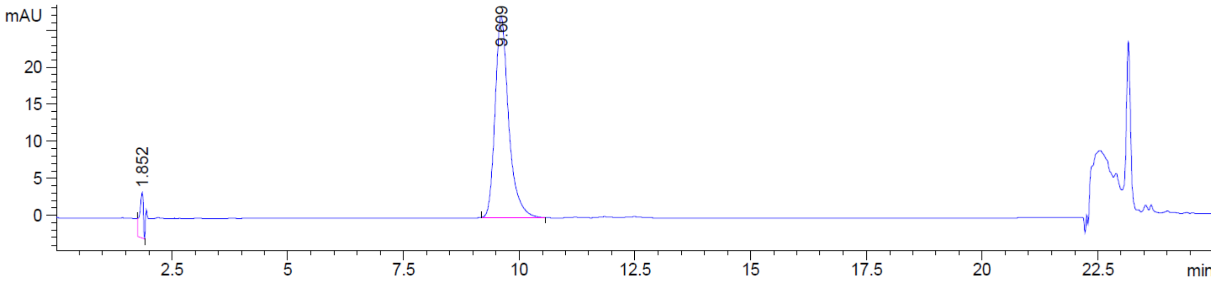

Peptide **10** (15% MeCN in 50 mM ammonium acetate buffer, pH 7, 1 mL/min):

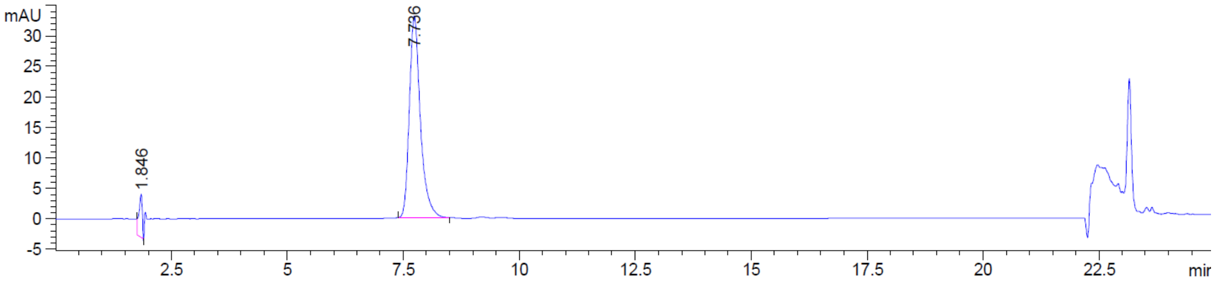

Peptide **11** (15% MeCN in 50 mM ammonium acetate buffer, pH 7, 1 mL/min):

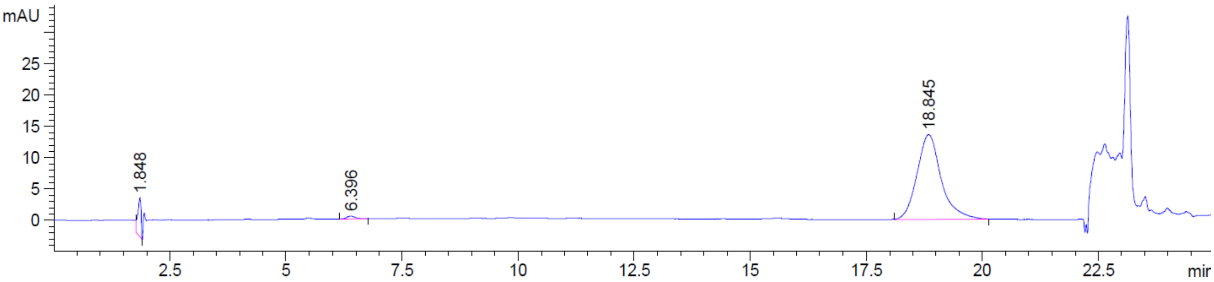

Peptide **11** (17% MeCN in 50 mM ammonium acetate buffer, pH 7, 1 mL/min):

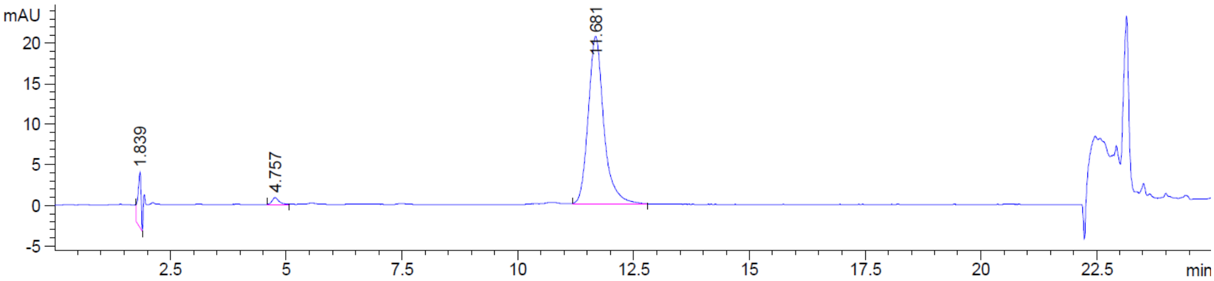

Peptide **11** (18% MeCN in 50 mM ammonium acetate buffer, pH 7, 1 mL/min):

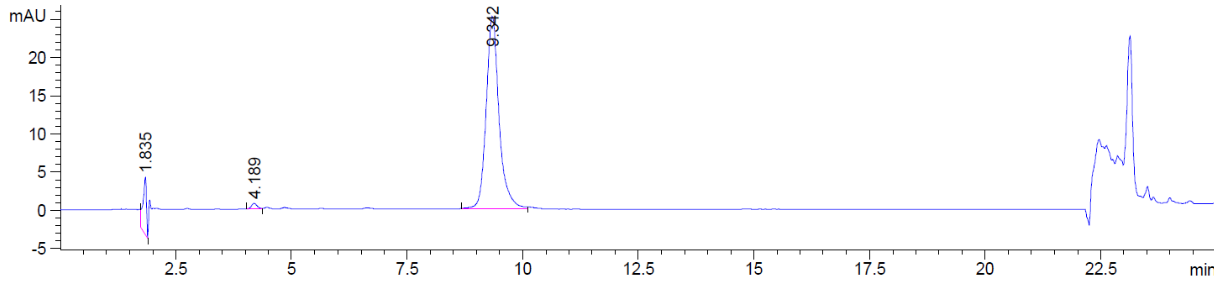

Peptide **11** (19% MeCN in 50 mM ammonium acetate buffer, pH 7, 1 mL/min):

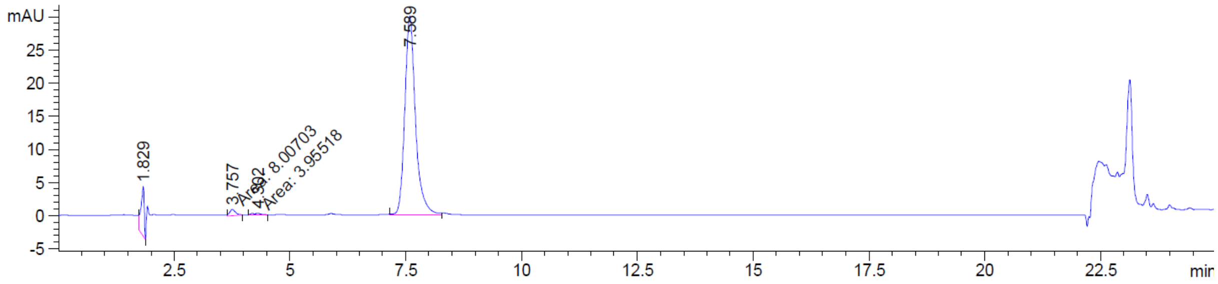

Peptide **12** (19% MeCN in 50 mM ammonium acetate buffer, pH 7, 1 mL/min):

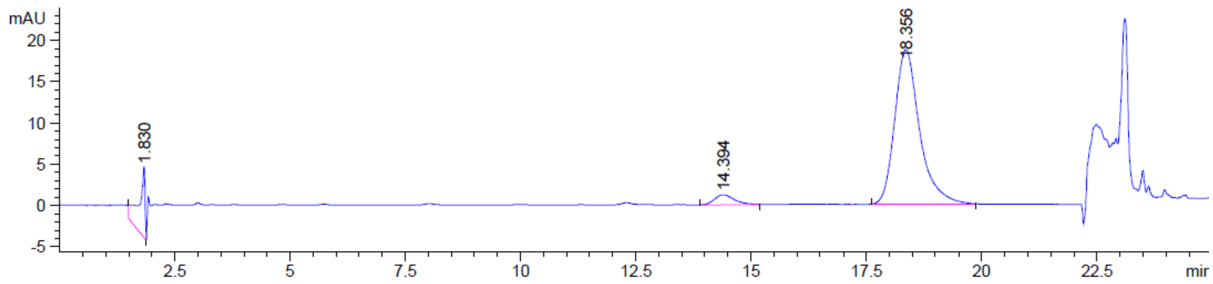

Peptide **12** (20% MeCN in 50 mM ammonium acetate buffer, pH 7, 1 mL/min):

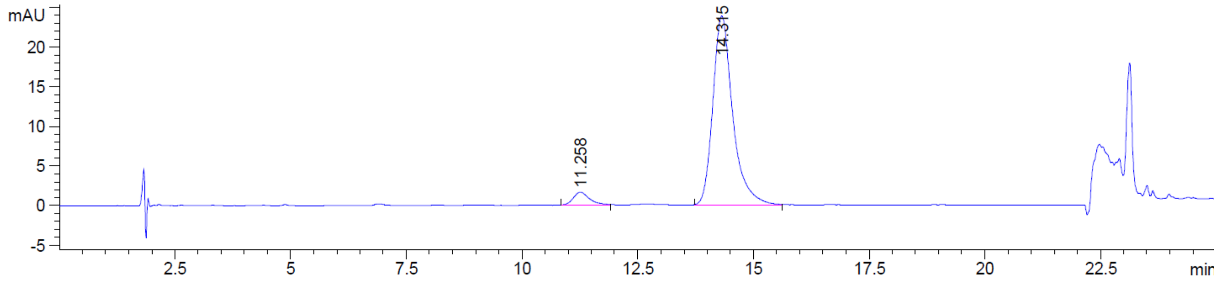

Peptide **12** (21% MeCN in 50 mM ammonium acetate buffer, pH 7, 1 mL/min):

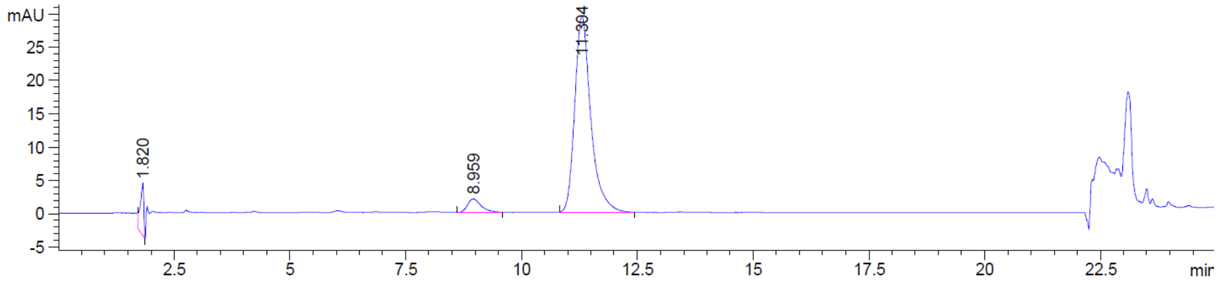

Peptide **12** (22% MeCN in 50 mM ammonium acetate buffer, pH 7, 1 mL/min):

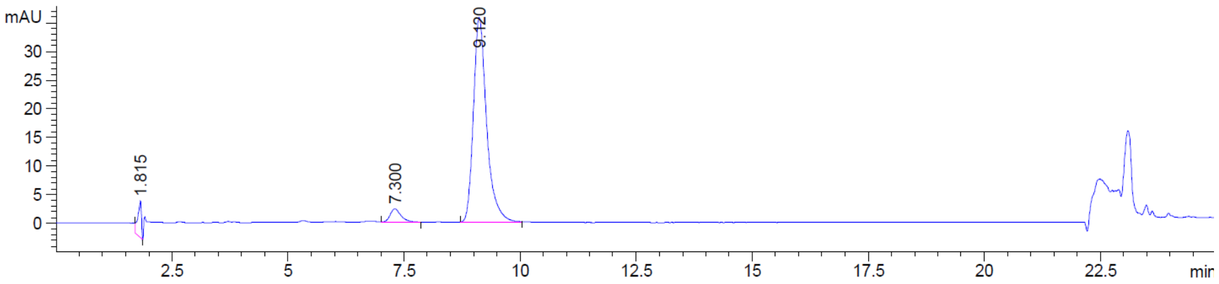

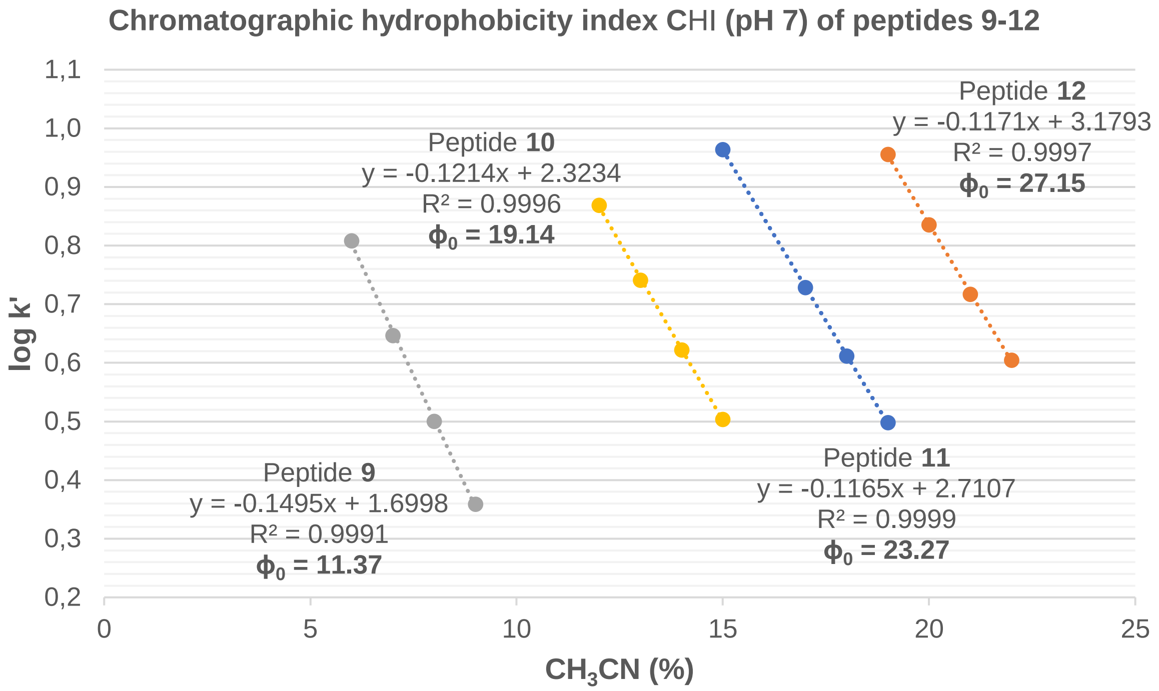

Figure S5: RP-HPLC determination of the chromatographic hydrophobicity index CHI for peptides **9-12** at pH 7 by linear regression of log k' values as a function of MeCN content.

7. pK_a determination

The effect of the CF_3S group on the pK_a value was determined on the tyramine model by ^{1}H and ^{19}F NMR spectroscopy according to a slightly modified literature procedure.[18] The determination is based on the dependence of the chemical shift of the *ortho* vicinal NMR active nucleus on the protonation state of the phenolic function of tyramine. By recording ^{1}H (or ^{19}F) NMR spectra at different pH values, the chemical shift of the indicative resonance adjacent to the acidic phenolic function can be plotted against the pH of the solution. The inflection point of the resulting sigmoidal curve is indicative of the pK_a value of the phenol.

Approximately 25 mL of the stock solution of the analyte in mQ H_2O was prepared (adjusted to obtain the final 1 mM concentration in the NMR tubes). 450 µL of the stock solution was aliquoted into the Eppendorf vial, and the pH of the solution was measured (the pH meter was calibrated at three points; calibration curve > 0.95. The stock solution was then titrated with 0.5 M NaOH (aq.) and 0.5 M HCl (aq.) solutions while the pH was measured. 450 µL aliquots of the solution were taken at representative pH values. A DSS (sodium trimethylsilylpropanesulfonate) solution was then prepared separately in D_2O at a concentration adjusted to the final concentration of 27.5 µM in the NMR tubes. NMR samples were prepared by adding 50 µL of the D_2O reference solution to the vials to obtain 90:10 H_2O/D_2O tyramine triflate salt solutions with different pH values, which were transferred to the NMR tubes. The ^{1}H NMR spectrum of the first sample was recorded manually to determine the frequency for Watergate solvent suppression. The other spectra were recorded in automation and referenced to DSS at 0.00 ppm. Chemical shifts of the reporter proton (*ortho* to the hydroxyl group) were plotted against solution pH. Non-linear curve fitting of the resulting sigmoidal curve was performed using GraphPad Prism software. The inflection point gave the pK_a value, which was corrected by subtracting 0.04 pK_a units due to the deuterium isotope effect and the sample consisting of 10% D_2O.[19]

First, we evaluated our protocol by determining the pK_a value of a non-fluorinated compound **5e**. Tyramine triflate salt was used as a reference and prepared by adding TfOH (2.5 equiv) in DCM (0.1 M), stirring for 1 h, and performing the same workup as for **6e**. The determined pK_a value of the **5e** triflate salt is comparable to the ^{1}H NMR spectroscopically determined pK_a value of paracetamol reported in the literature (paracetamol: 9.80; triflate salt of **5e**: 9.87)[18] and to the pK_a values determined for tyramine by a spectroscopic pH-titration method (9.62 in H_2O).[20] The pK_a value of the compound **6e** is reported as the average of two measurements. Moreover, in the case of (CF_3S)-tyramine **6e**, hexafluorobenzene was added as a reference after the ^{1}H NMR pK_a determination to check whether the pK_a value could be determined by ^{19}F NMR spectroscopy. ^{19}F NMR spectra were obtained in automation and referenced to C_6F_6 at –163.01 ppm and were subjected to the same processing as the proton spectra. The CF_3S group proved to be a sensitive reporter and gave comparable results (both giving an averaged pK_a value of about 8.1) to the ^{1}H NMR determination using the *ortho*-proton as the reporter nucleus.

Table of chemical shifts of ^{1}H and ^{19}F nuclei depending on the solution pH, R^2 values, and extracted pK_a values:

Entry	pH	Tyramine triflate salt (**5e**) *ortho*-resonance (ppm)	pH	(CF_3S)-Tyramine triflate salt (**6e**) 1st *ortho*-resonance (ppm)	(CF_3S)-Tyramine triflate salt (**6e**) 1st CF_3S-resonance (ppm)	pH	(CF_3S)-Tyramine triflate salt (**6e**) 2nd *ortho*-resonance (ppm)	(CF_3S)-Tyramine triflate salt (**6e**) 2nd CF_3S-resonance (ppm)
1	3.55	6.89	3.43	7.06	-43.22	4.32	7.06	-43.21
2	7.32	6.89	4.11	7.06	-43.22	5.65	7.04	-43.22
3	8.56	6.89	6.50	7.03	-43.24	6.11	7.04	-43.23
4	9.00	6.87	7.31	6.97	-43.30	6.69	7.01	-43.24
5	9.48	6.80	7.77	6.95	-43.28	7.18	6.99	-43.26
6	9.81	6.76	8.02	6.90	-43.33	7.58	6.96	-43.28
7	10.12	6.72	8.43	6.86	-43.35	7.74	6.90	-43.33
8	10.52	6.68	9.05	6.78	-43.40	8.47	6.83	-43.38
9	10.94	6.63	9.51	6.76	-43.42	8.69	6.80	-43.40
10	11.15	6.62	10.04	6.71	-43.46	9.01	6.78	-43.41
11	11.47	6.61	11.05	6.69	-43.47	9.60	6.74	-43.44
12	11.99	6.60	12.48	6.68	-43.47	10.09	6.71	-43.46
13	12.21	6.60				10.62	6.70	-43.46
14	12.43	6.59				11.64	6.68	-43.46
pK_a		9.94		8.26	8.31		8.12	8.00
R^2		0.9947		0.9744	0.9771		0.9773	0.9836
std. error		0.04		0.11	0.11		0.10	0.09
adjusted pK_a		9.90		8.22	8.27		8.08	7.96

Zoomed in ^{1}H NMR spectra of **5e** triflate salt aromatic resonances at different pH, 400 MHz:

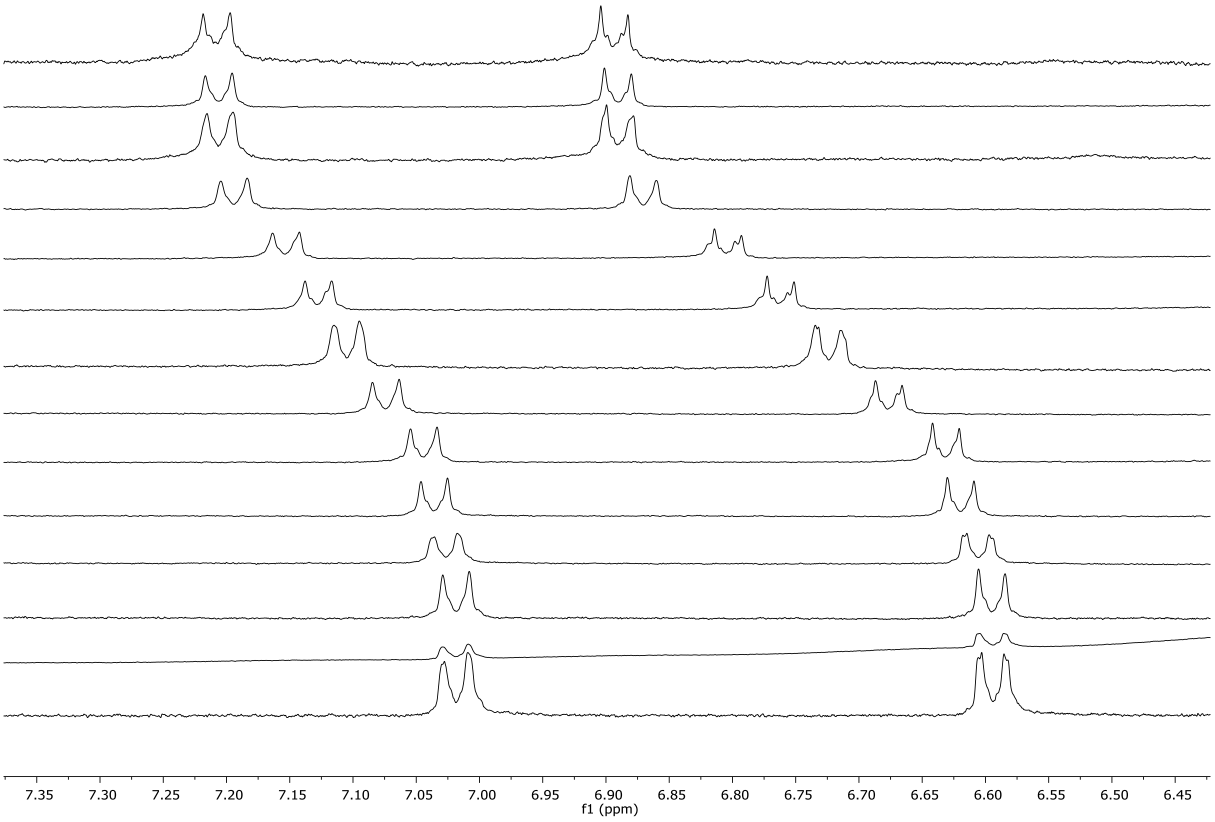

^{1}H NMR *ortho*-resonance (ppm) of **5e** as a function of the solution pH:

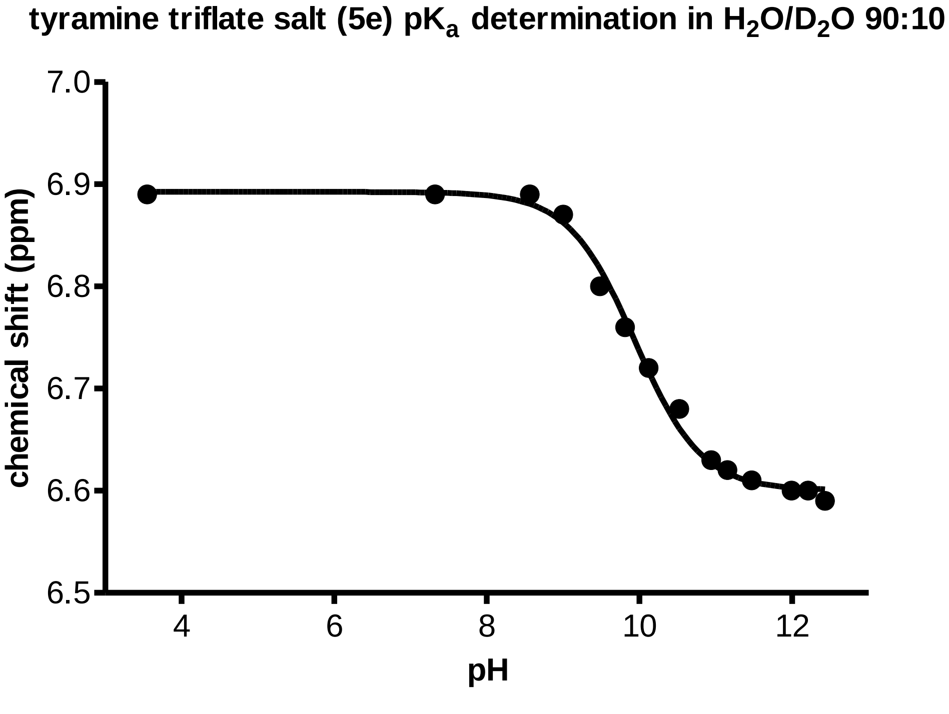

Zoomed in ^{1}H NMR spectra of **6e** aromatic resonances at different pH, 400 MHz (first analysis):

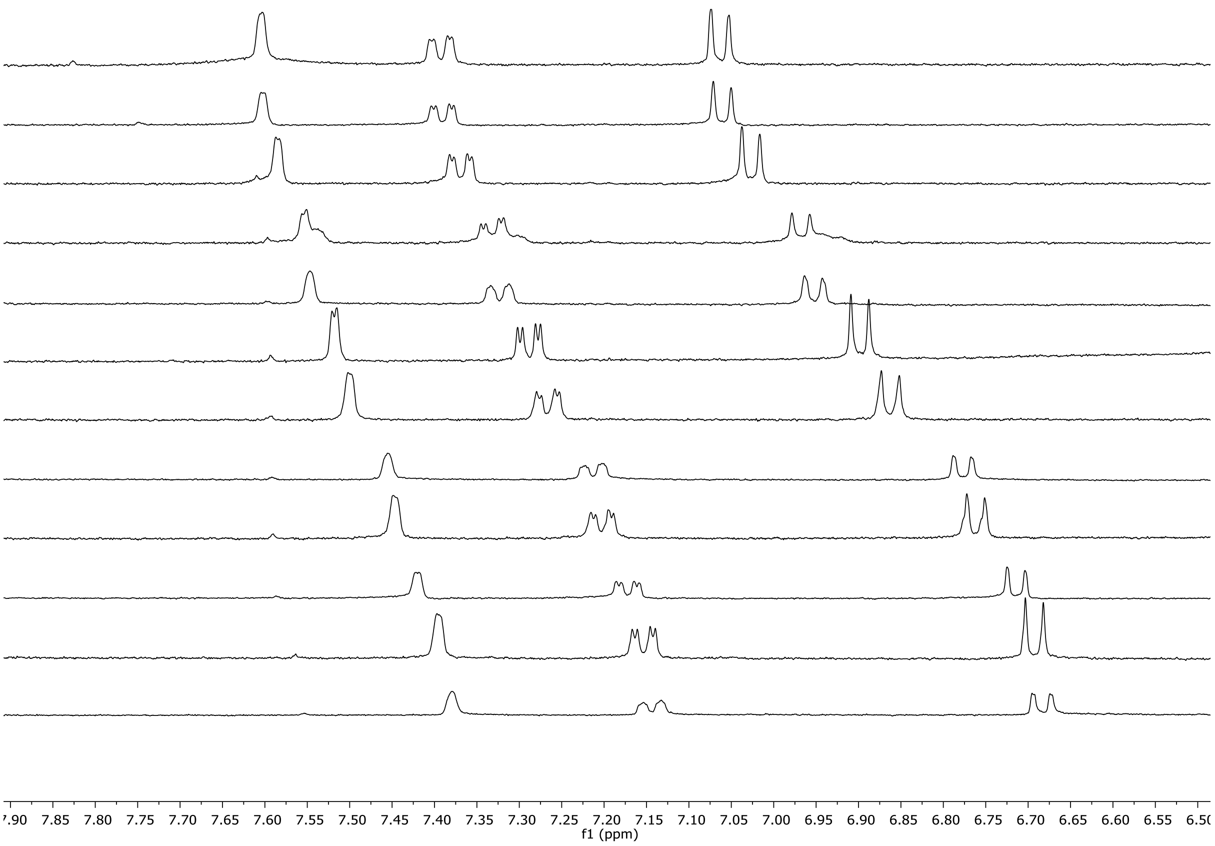

^{1}H NMR *ortho*-resonance (ppm) of **6e** as a function of the solution pH (first analysis):

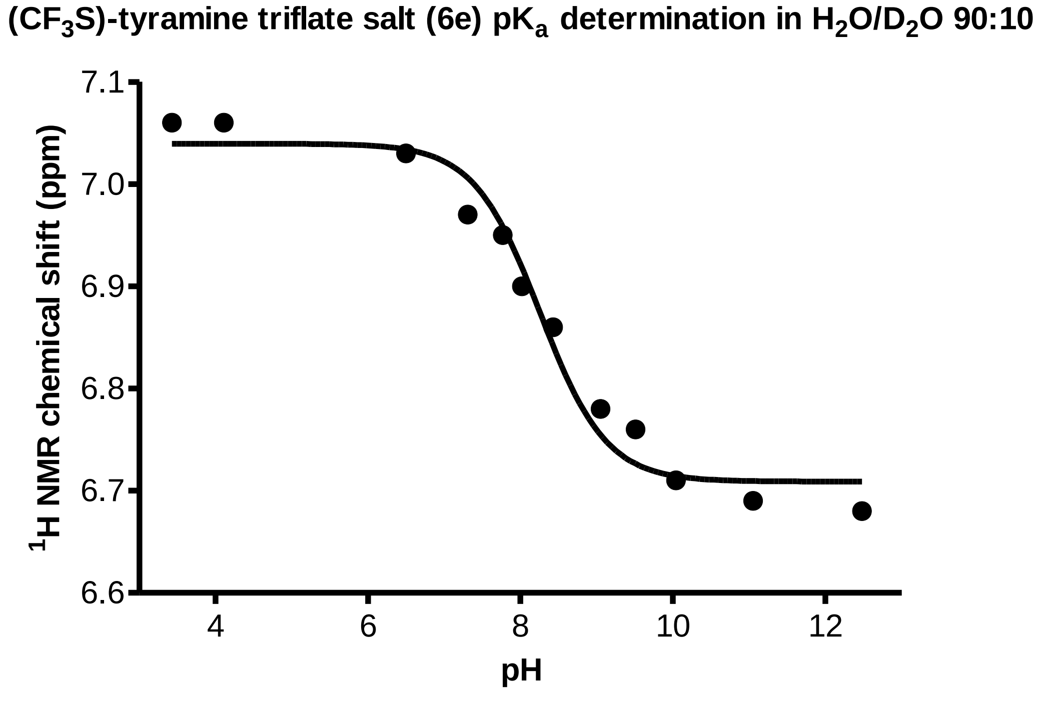

Zoomed in ^{19}F NMR spectra of **6e** CF_3S resonance at different pH, 376 MHz (first analysis):

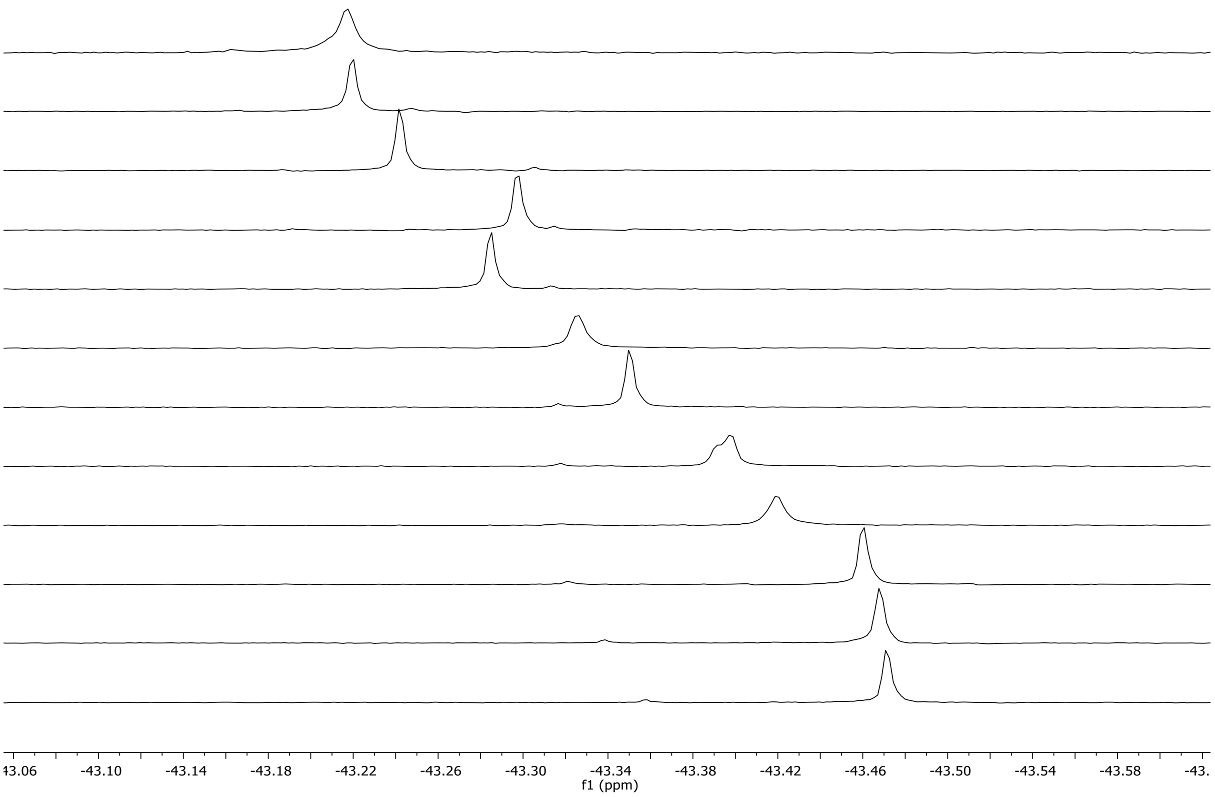

^{19}F NMR CF_3S resonance (ppm) of **6e** as a function of the solution pH (first analysis; the chemical shift at pH = 7.31 not taken into account for pK_a value calculation):

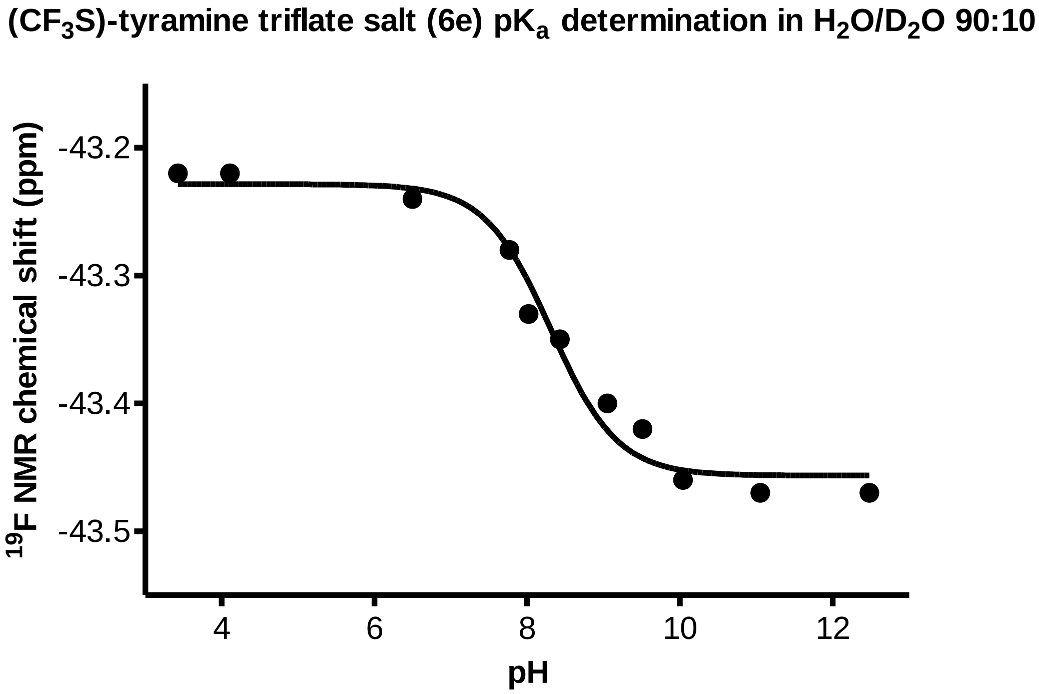

Zoomed in ^{1}H NMR spectra of **6e** aromatic resonances at different pH, 400 MHz (second analysis):

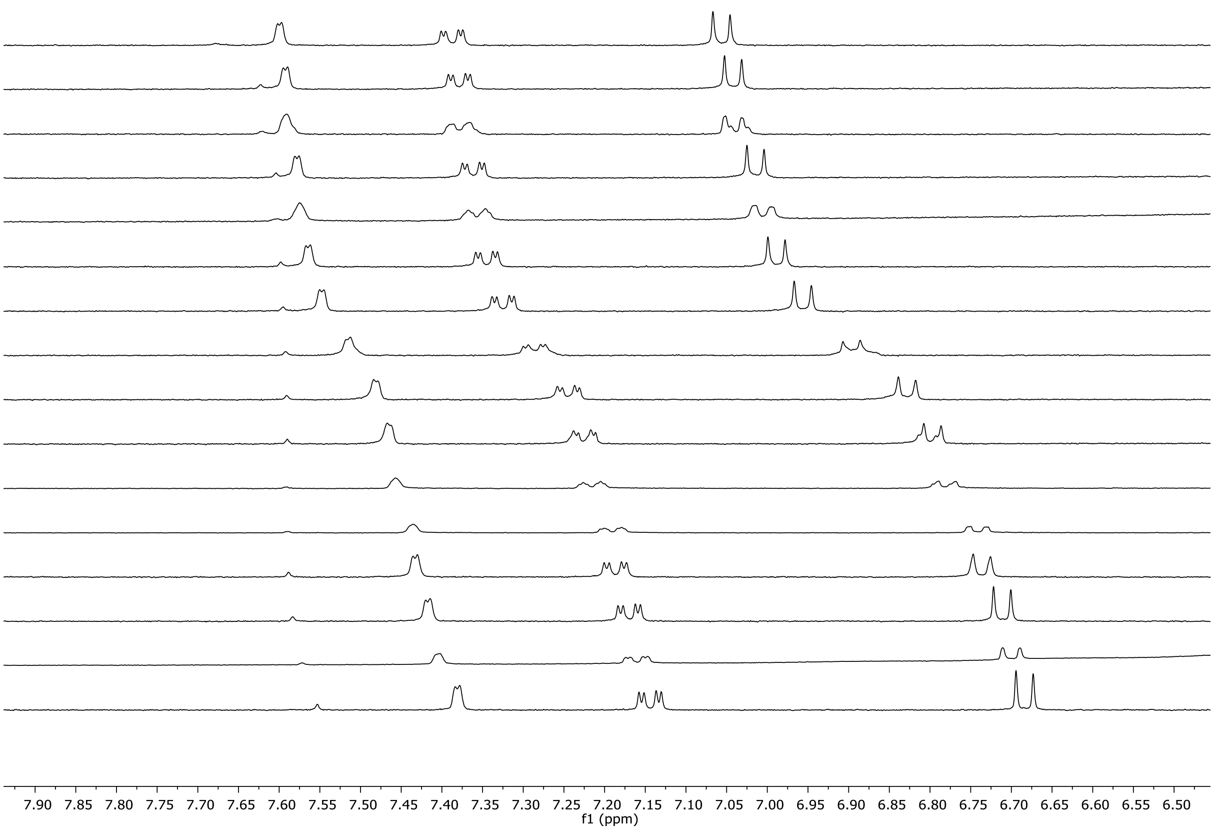

^{1}H NMR *ortho*-resonance (ppm) of **6e** as a function of the solution pH (second analysis):

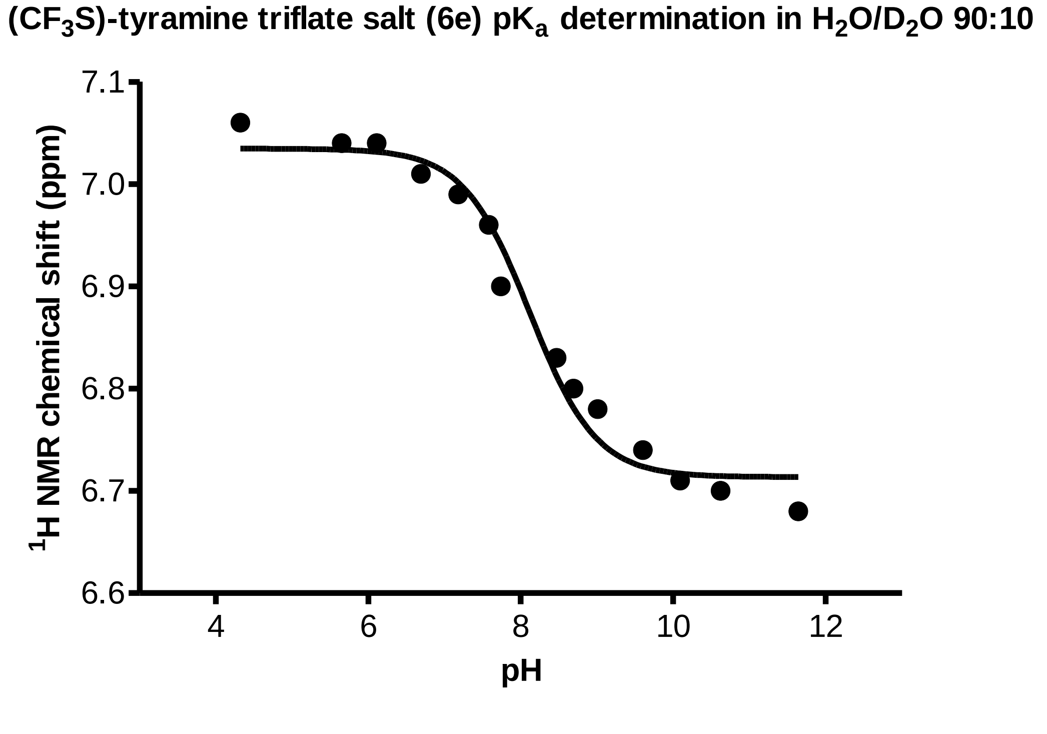

Zoomed in ^{19}F NMR spectra of **6e** CF_3S resonance at different pH, 376 MHz (second analysis):

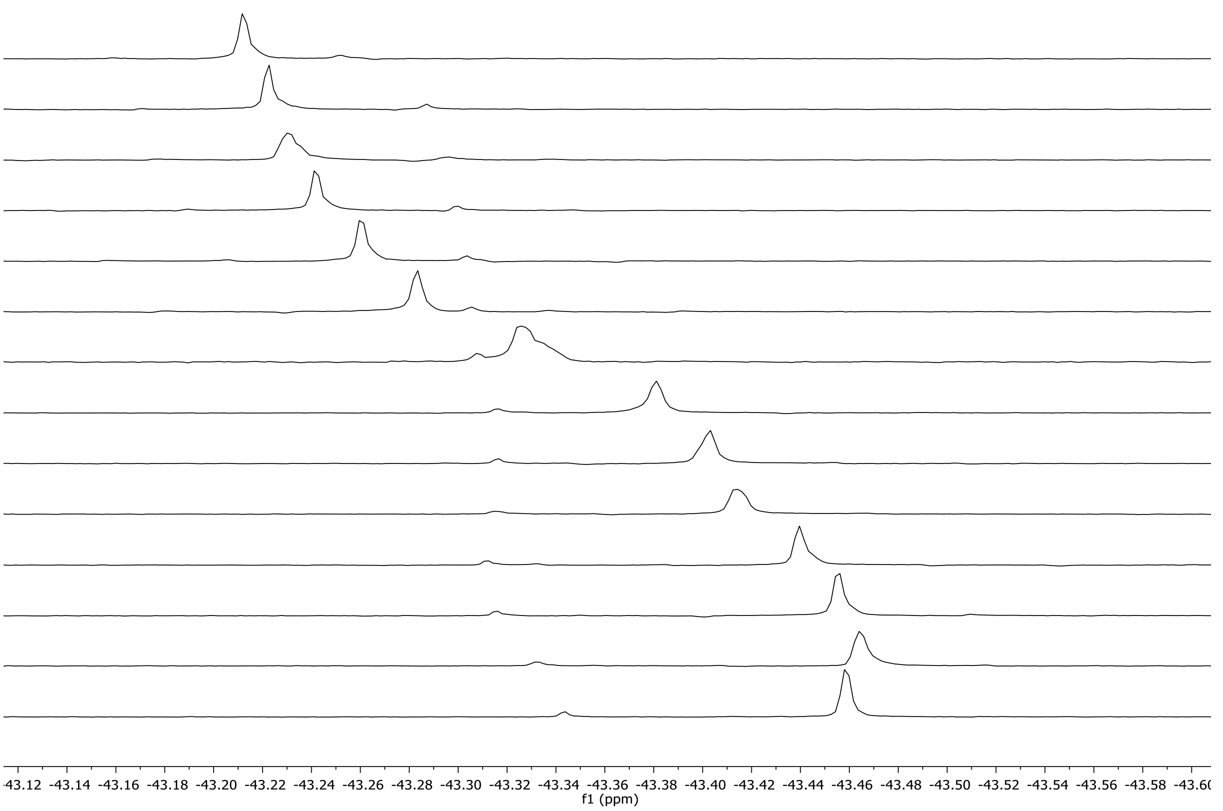

^{19}F NMR CF_3S resonance (ppm) of **6e** as a function of the solution pH (second analysis):

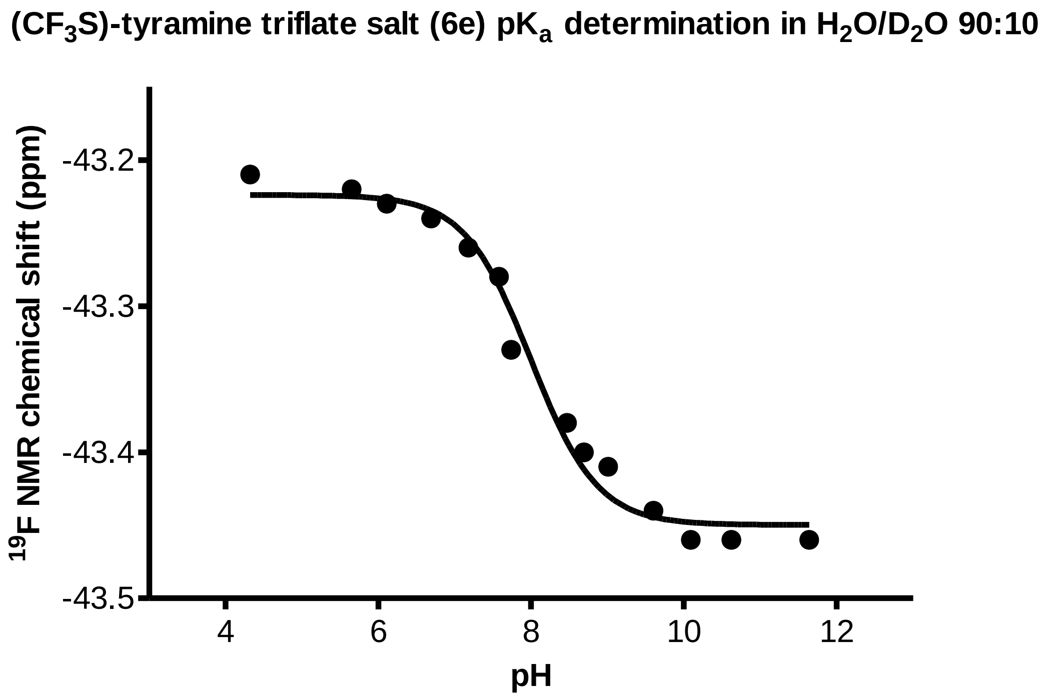

8. Copies of NMR spectra

8.1. NMR spectra of starting materials

^{1}H NMR spectrum of **1** in $CDCl_3$, 400 MHz

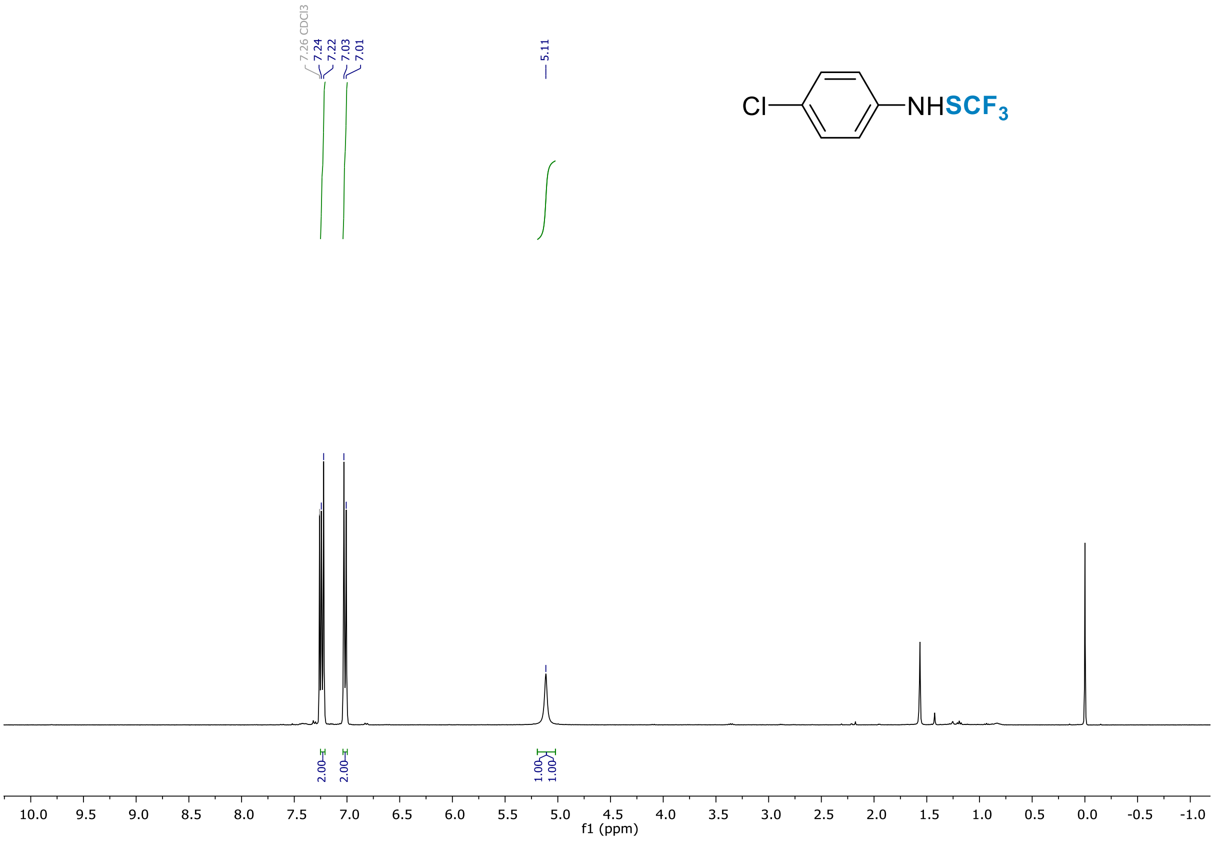

$^{13}C\{^{1}H\}$ NMR spectrum of **1** in $CDCl_3$, 126 MHz

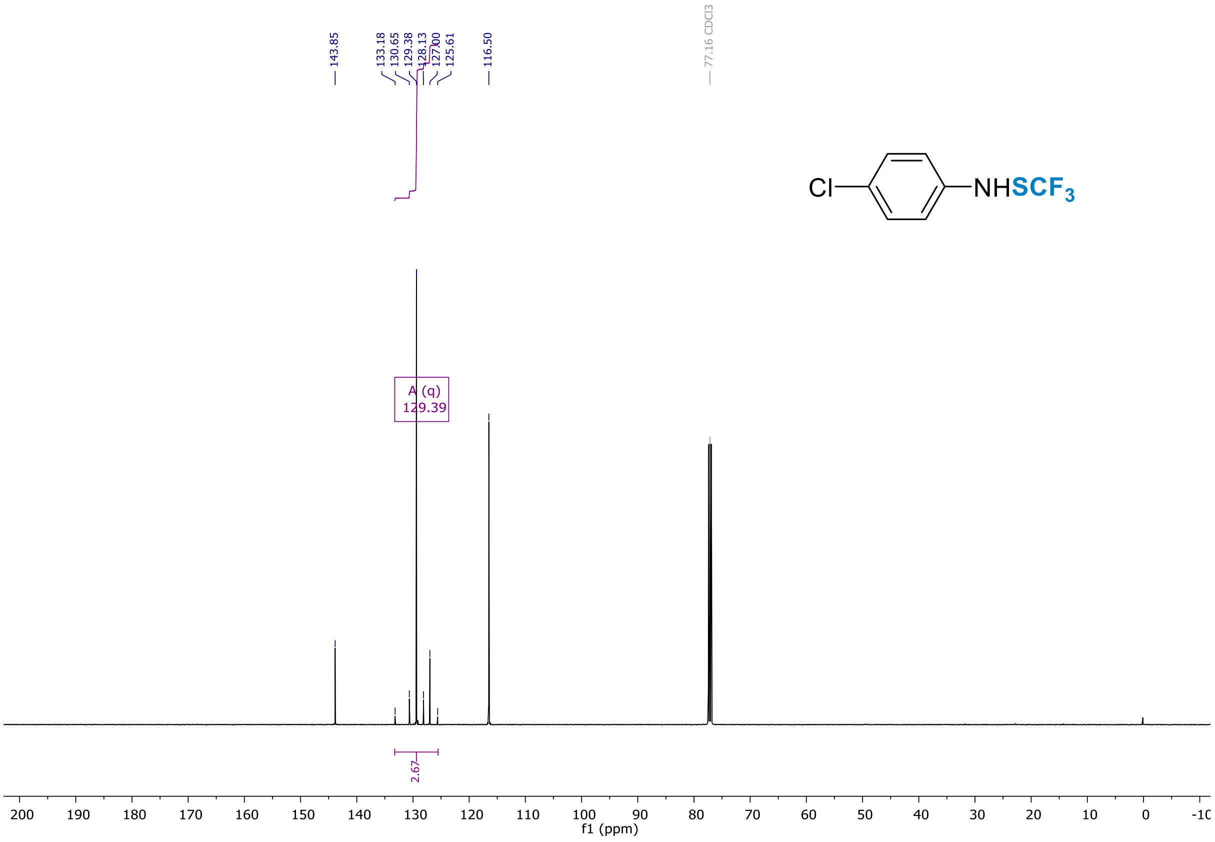

^{19}F NMR spectrum of **1** in $CDCl_3$, 367 MHz

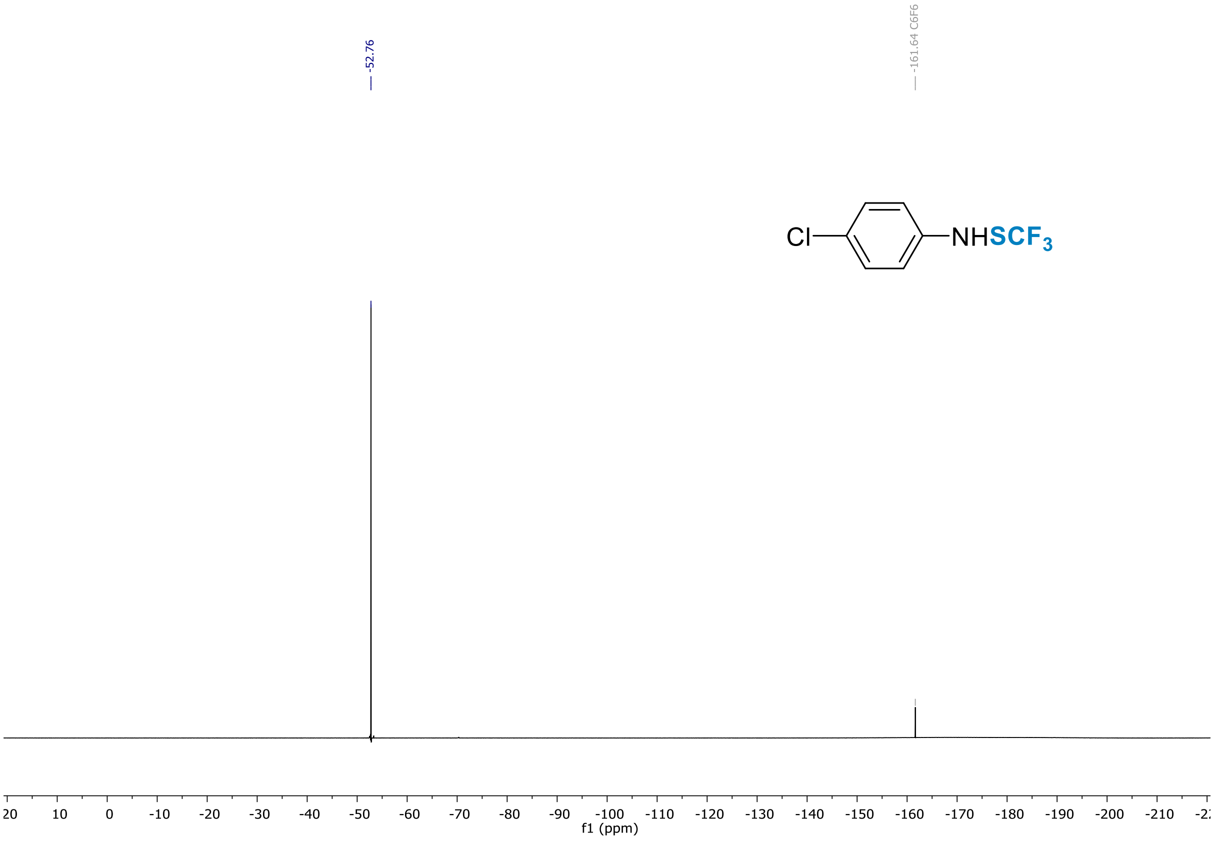

^{1}H NMR spectrum of **2a** in $CDCl_3$, 500 MHz

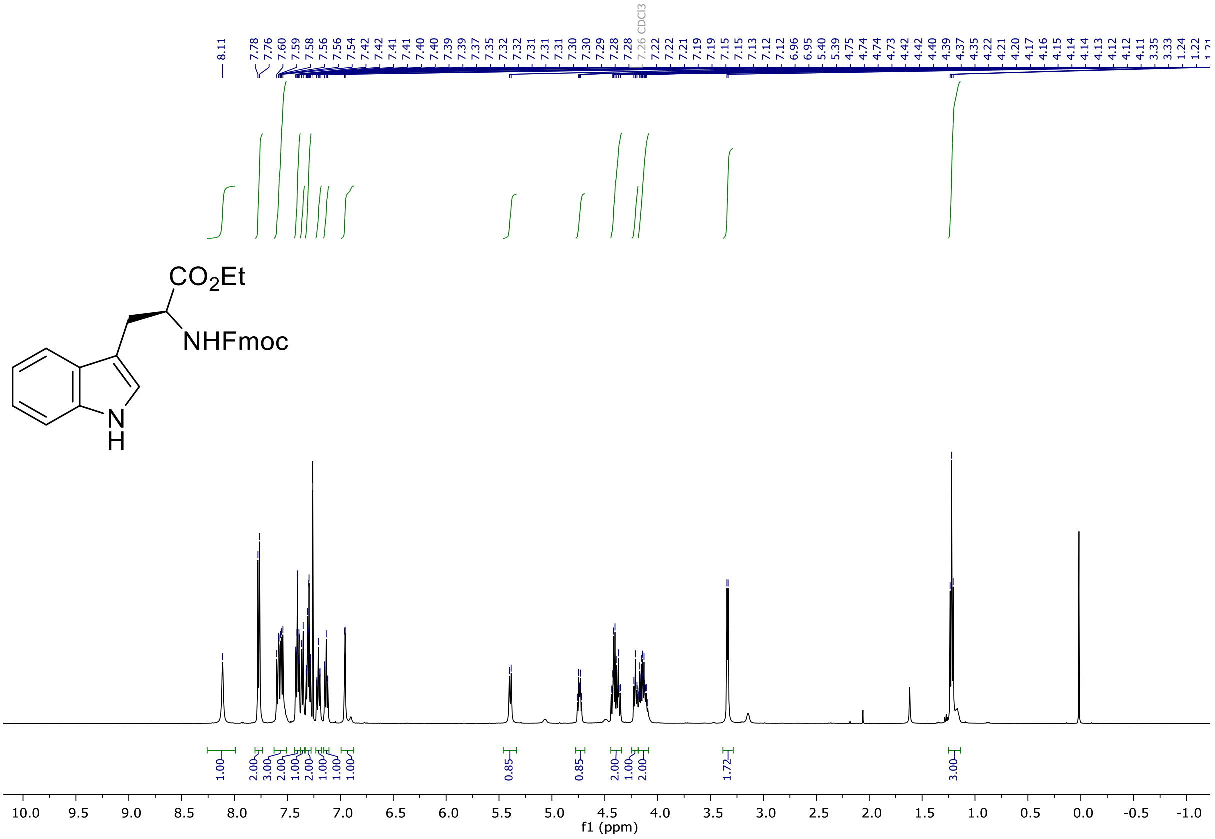

^{13}C{^{1}H} NMR spectrum of **2a** in $CDCl_3$, 126 MHz

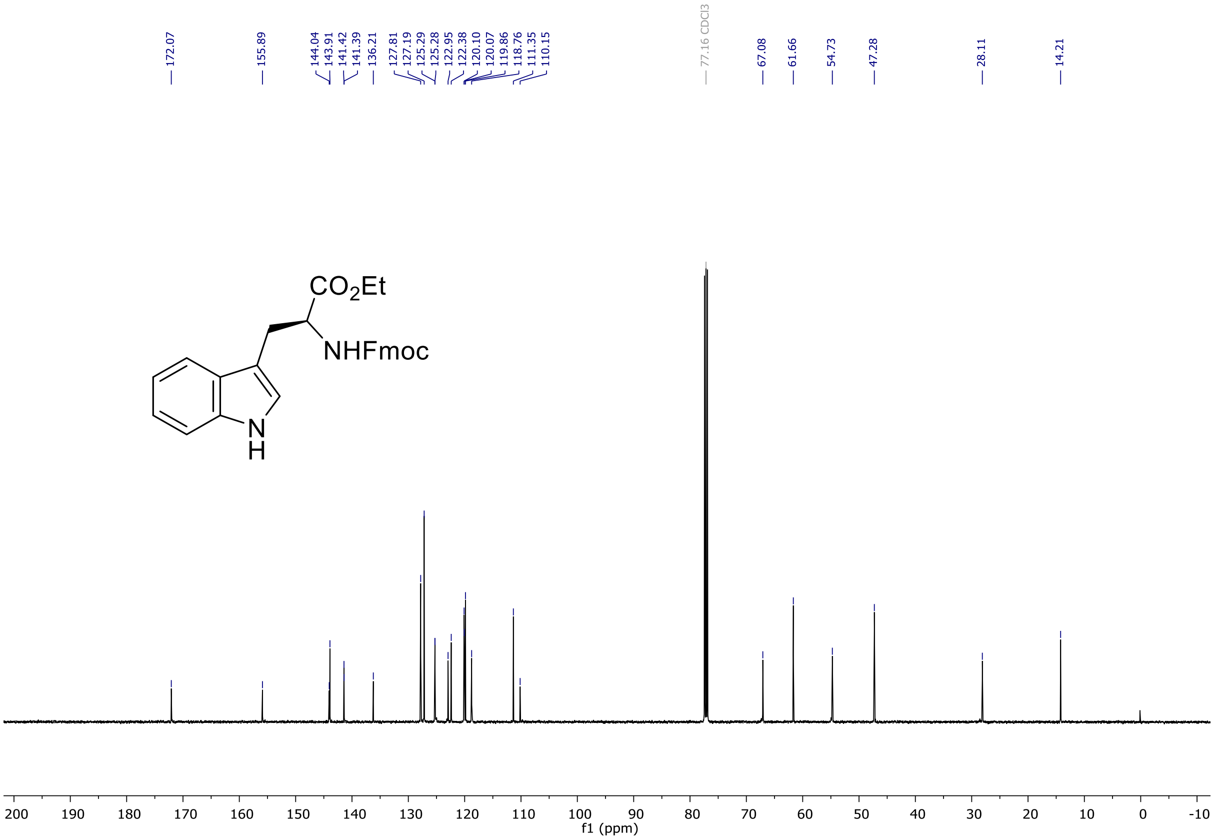

^{1}H NMR spectrum of **2d** in $CDCl_3$, 500 MHz

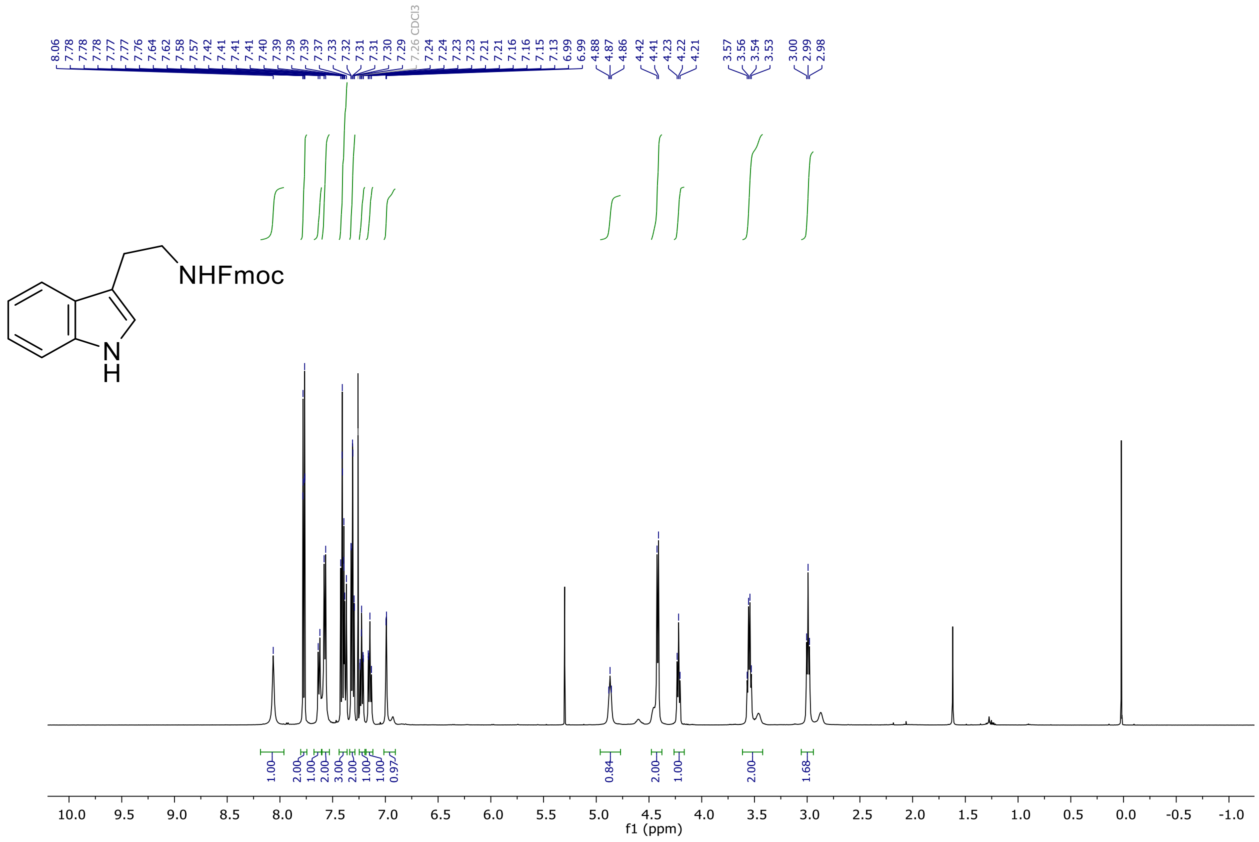

$^{13}C\{^{1}H\}$ NMR spectrum of **2d** in $CDCl_3$, 126 MHz

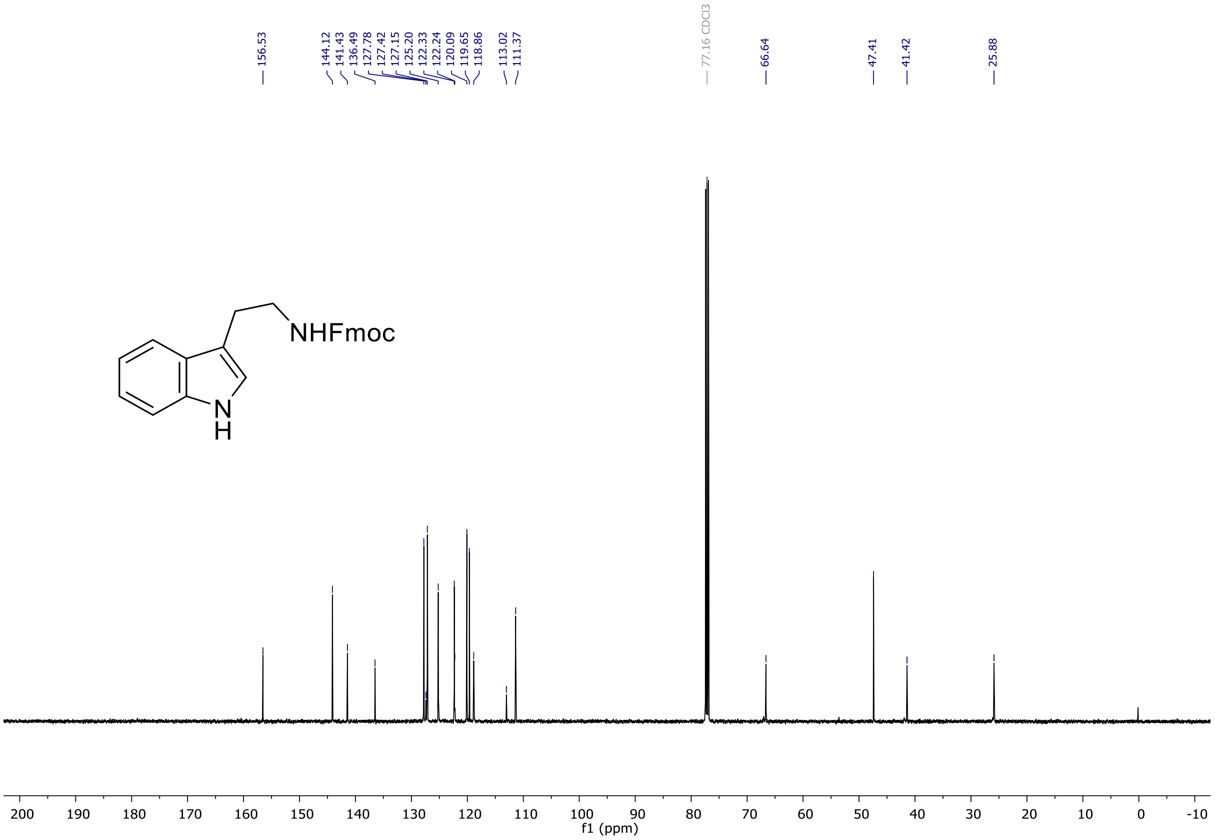

^{1}H NMR spectrum of **2g** in MeOD-d_4, 400 MHz

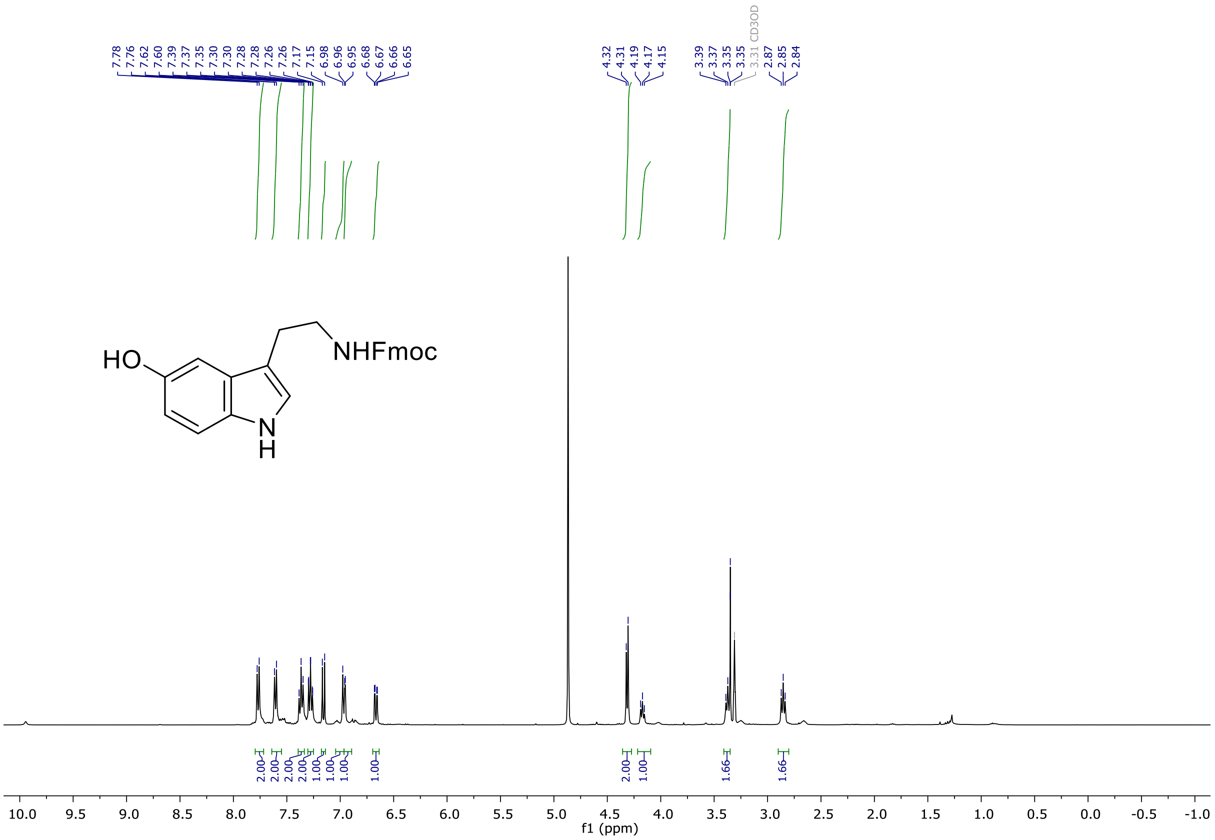

^{13}C{^{1}H} NMR spectrum of **2g** in MeOD-d_4, 101 MHz

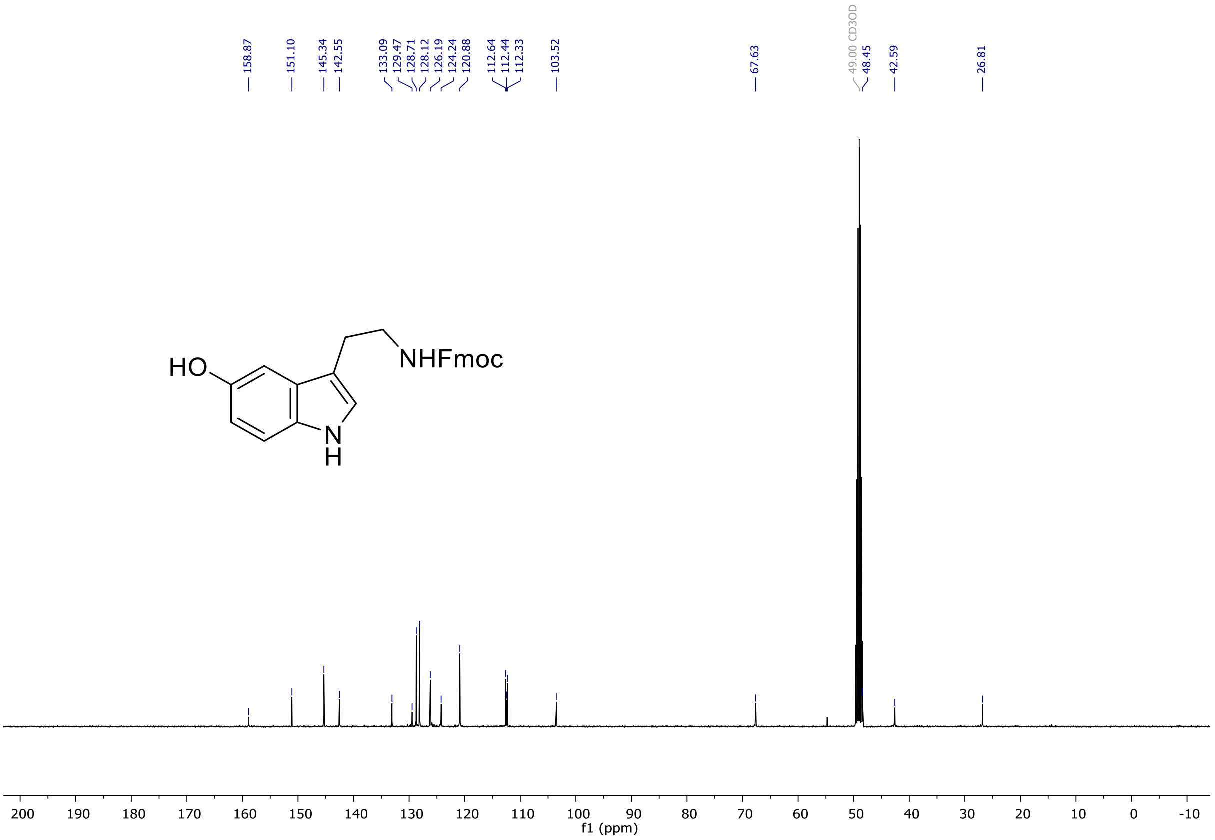

^{1}H NMR spectrum of **2h** in $CDCl_3$, 400 MHz

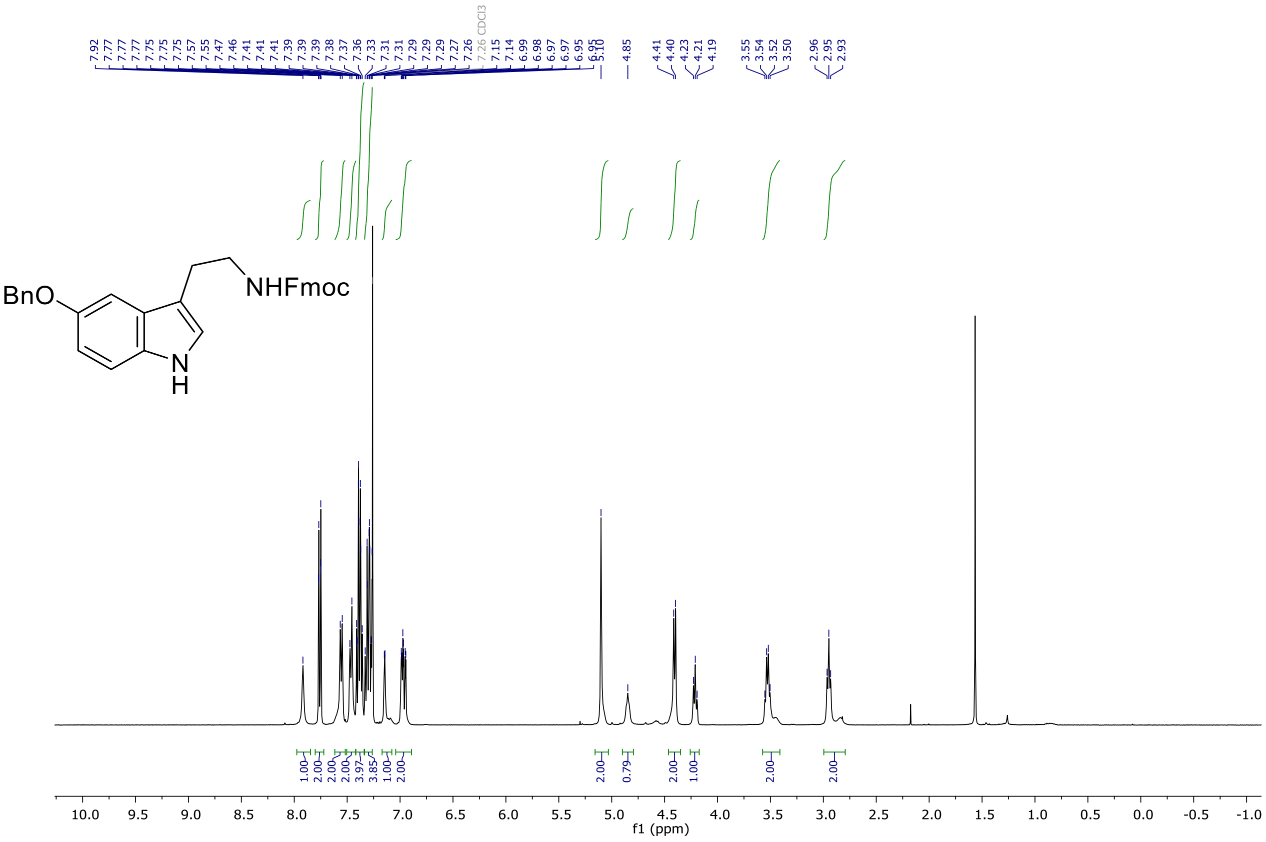

^{13}C{^{1}H} NMR spectrum of **2h** in $CDCl_3$, 101 MHz

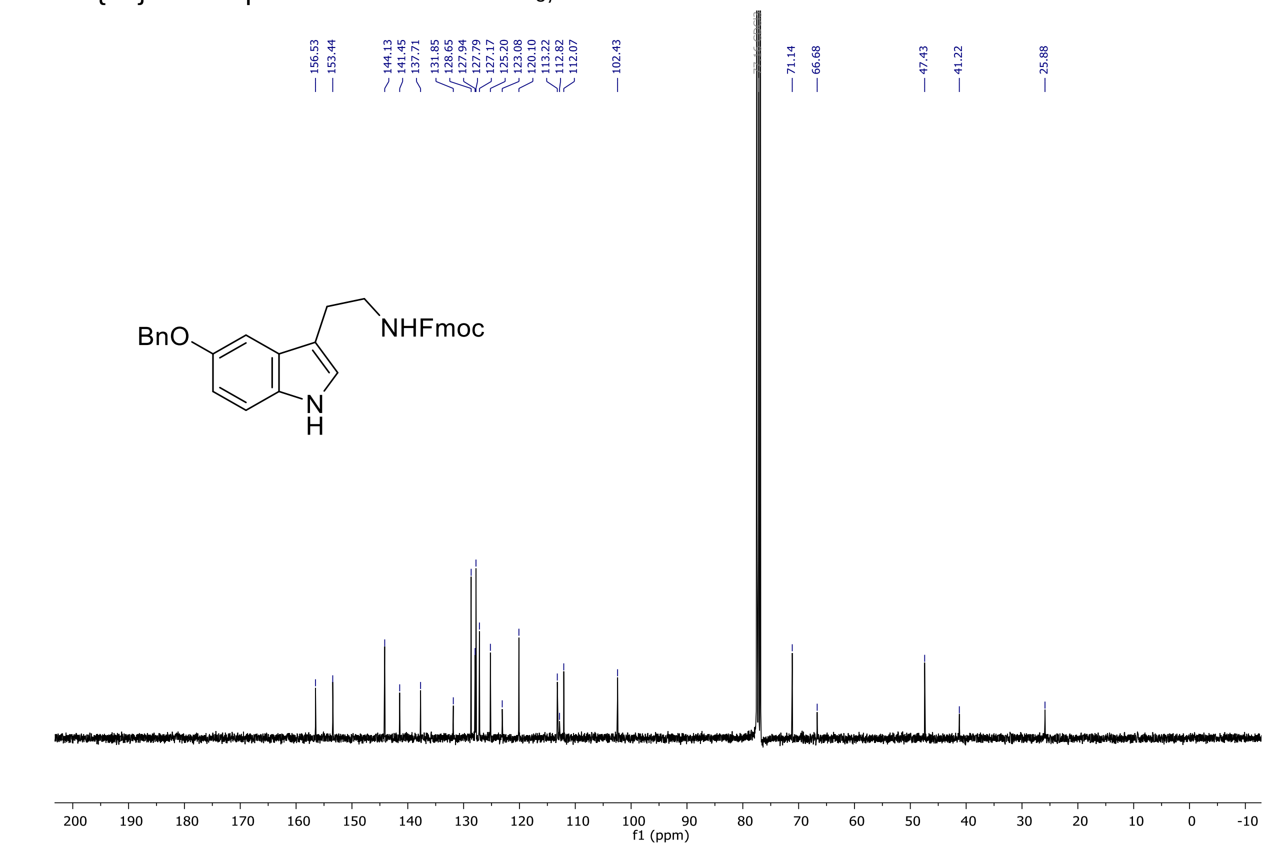

^{1}H NMR spectrum of **(*R*)-5a** in DMSO-d_6, 400 MHz

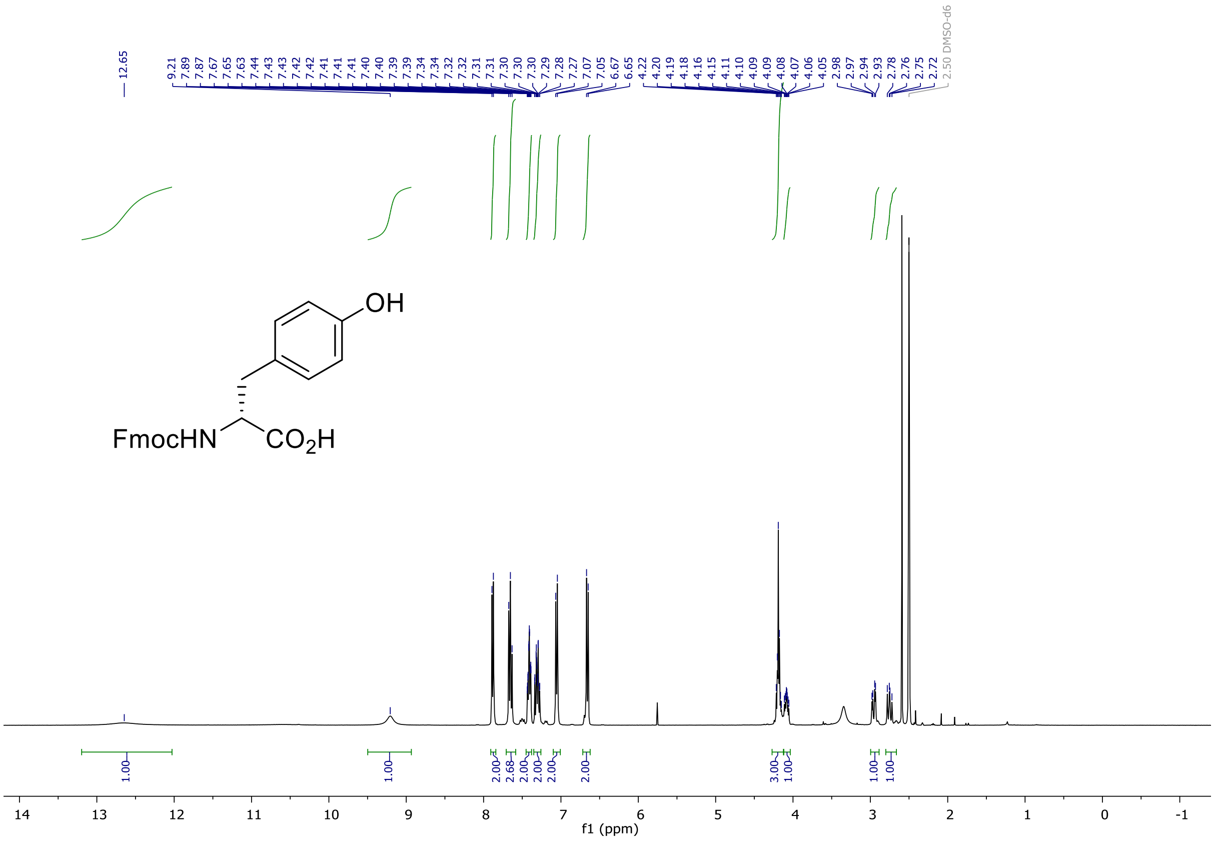

^{13}C{^{1}H} NMR spectrum of ***R*)-5a** in DMSO-d_6, 101 MHz

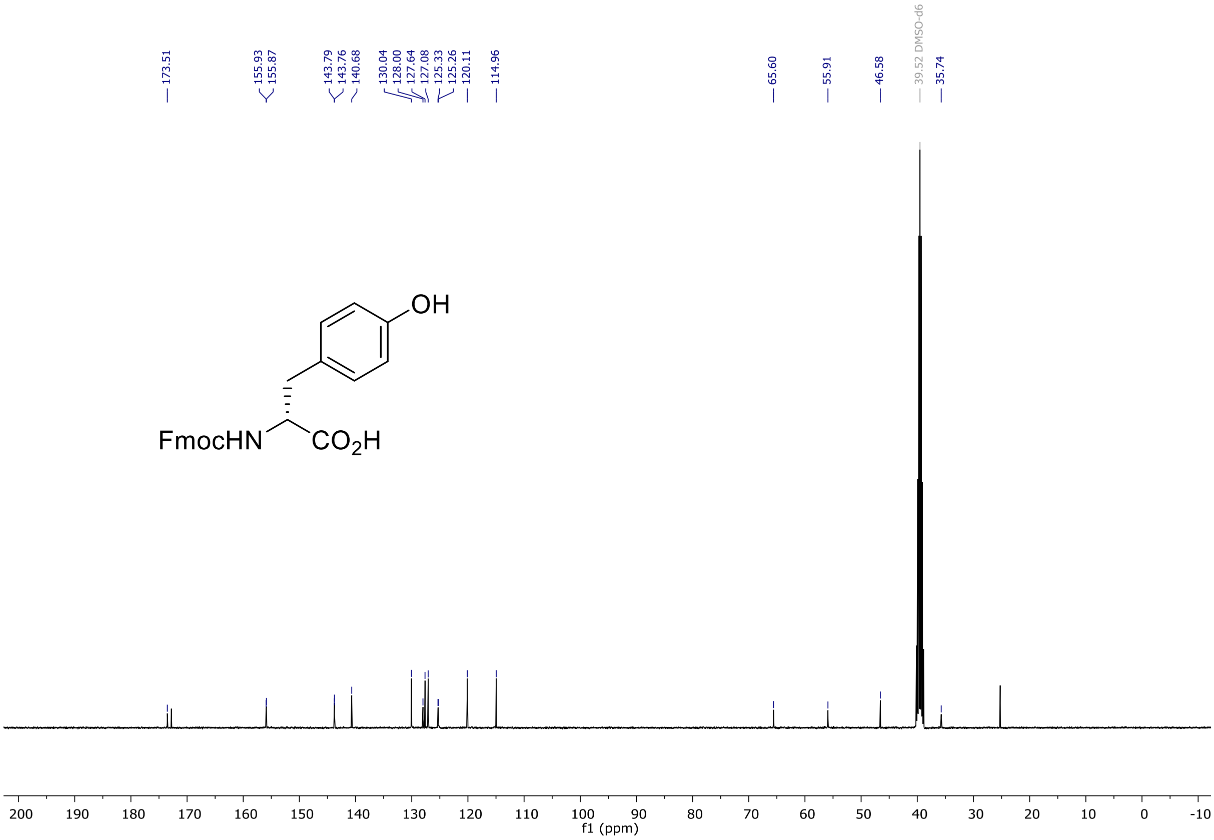

^{1}H NMR spectrum of **5b** in $CDCl_3$, 500 MHz

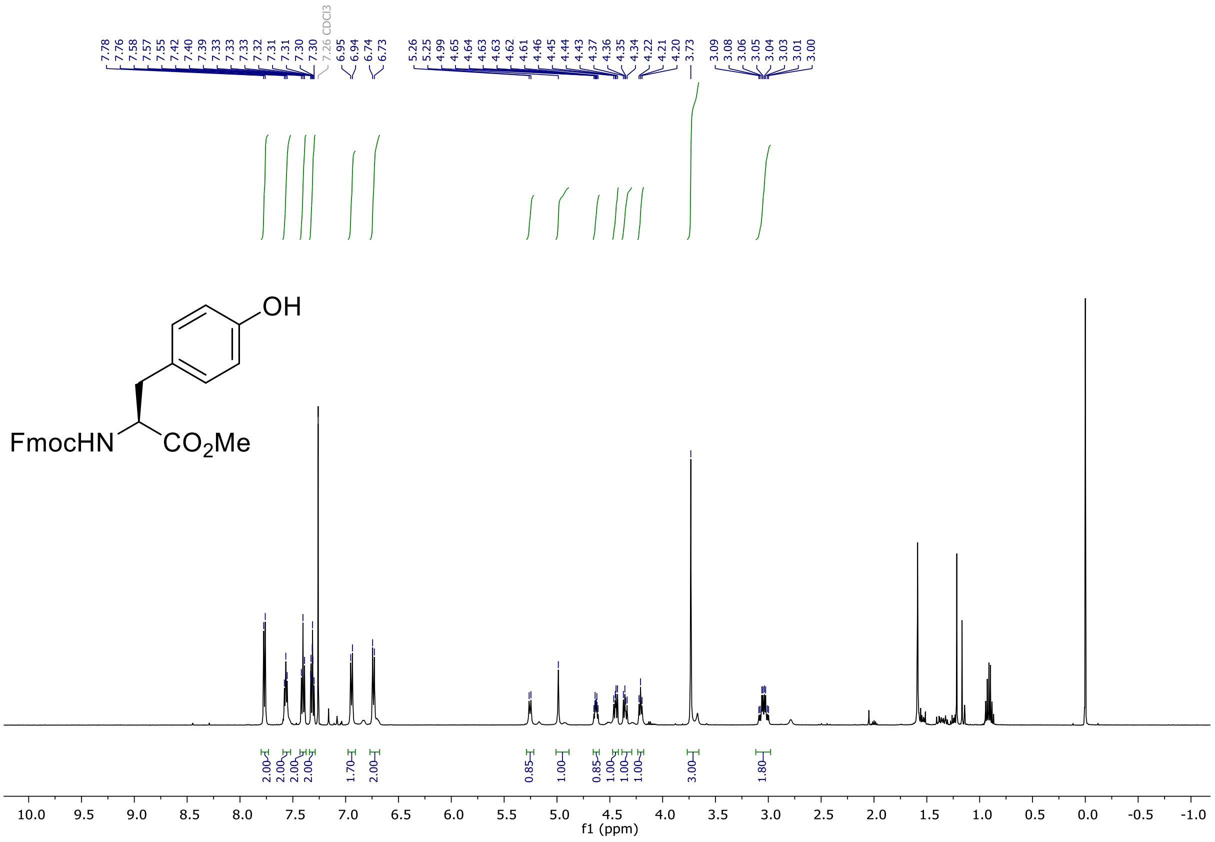

^{13}C{^{1}H} NMR spectrum of **5b** in $CDCl_3$, 126 MHz

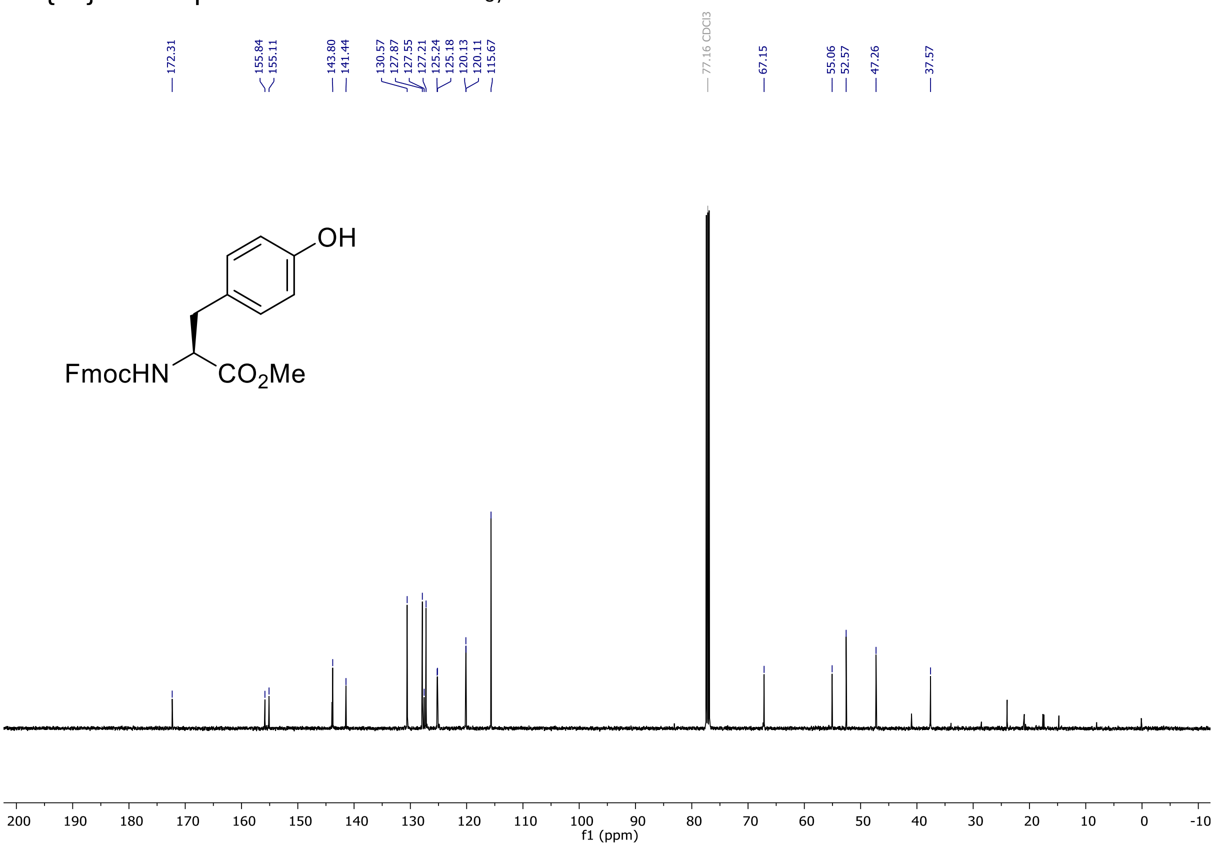

^{1}H NMR spectrum of **5d** in DMSO-d_6, 500 MHz

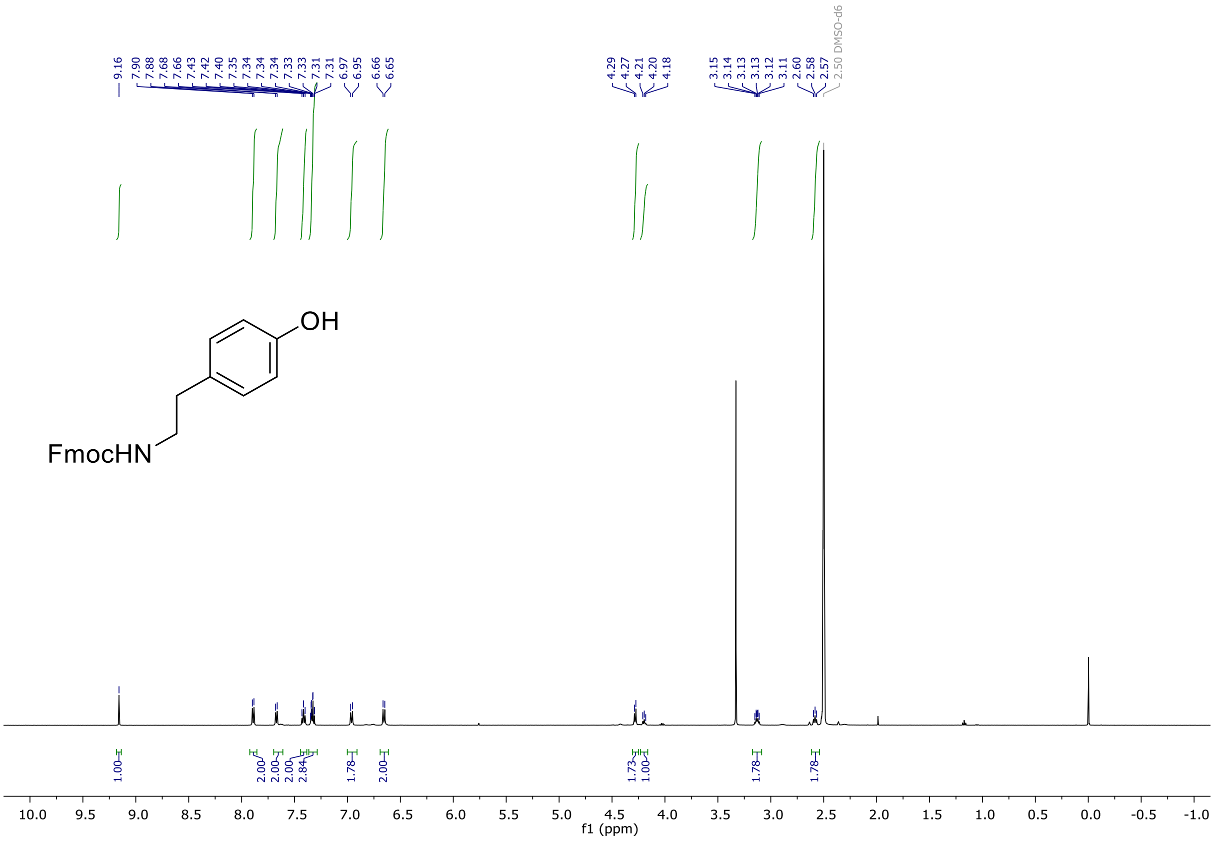

^{13}C{^{1}H} NMR spectrum of **5d** in DMSO-d_6, 126 MHz

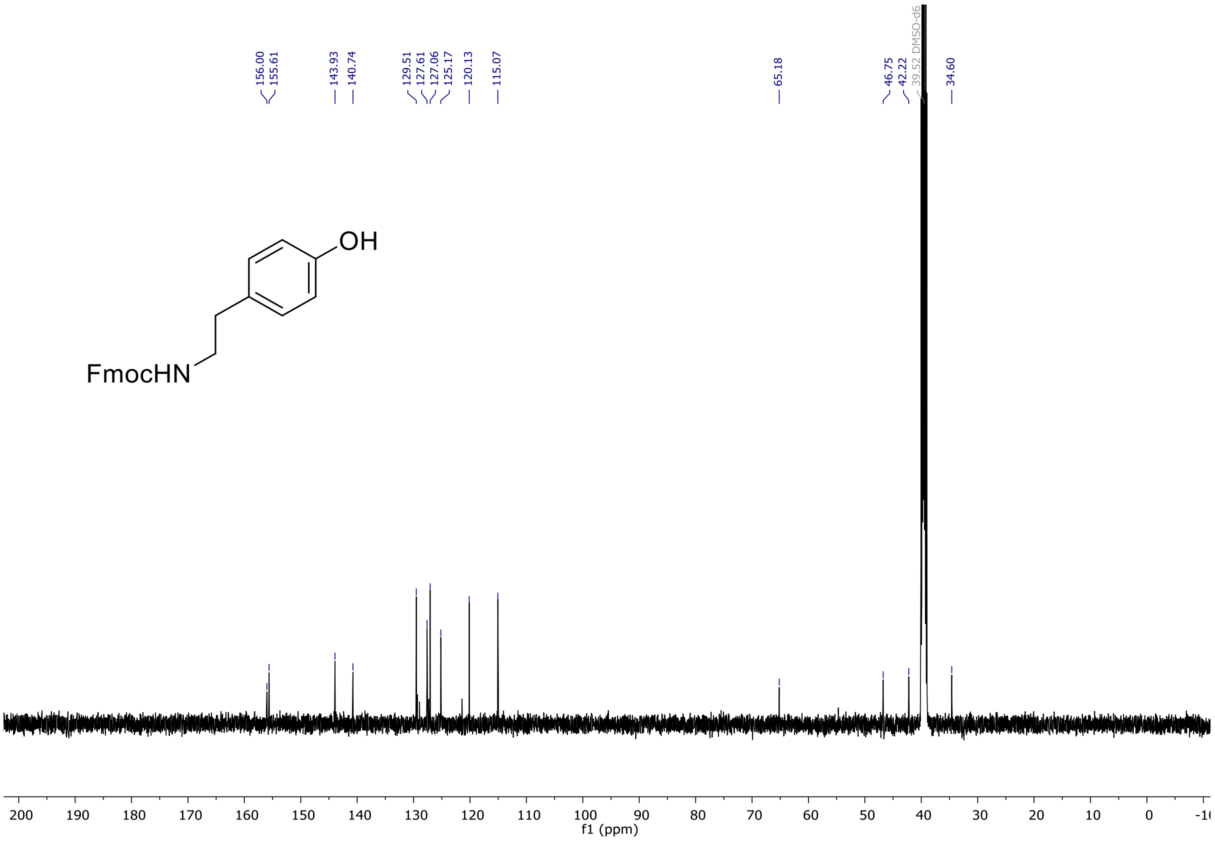

^{1}H NMR spectrum of **5f** in DMSO-d_6, 500 MHz

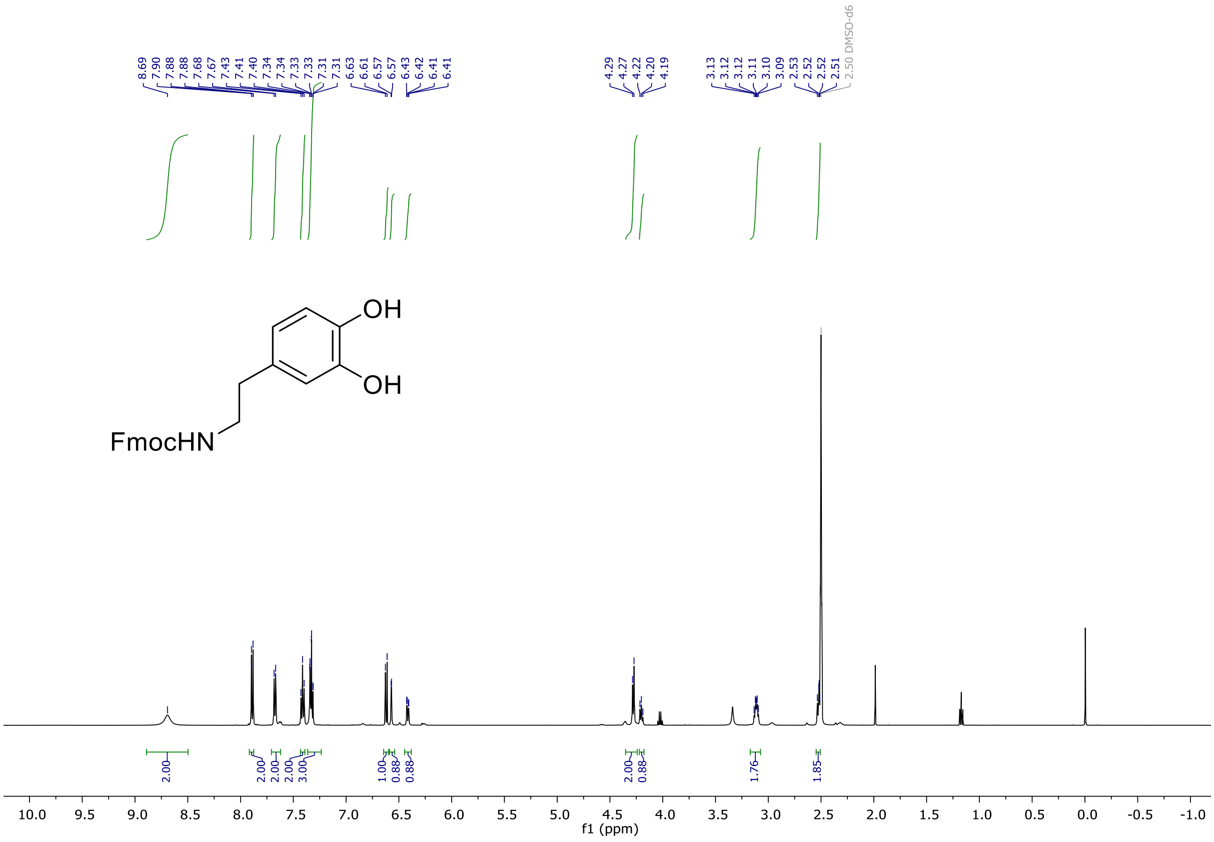

^{13}C{^{1}H} NMR spectrum of **5f** in DMSO-d_6, 126 MHz

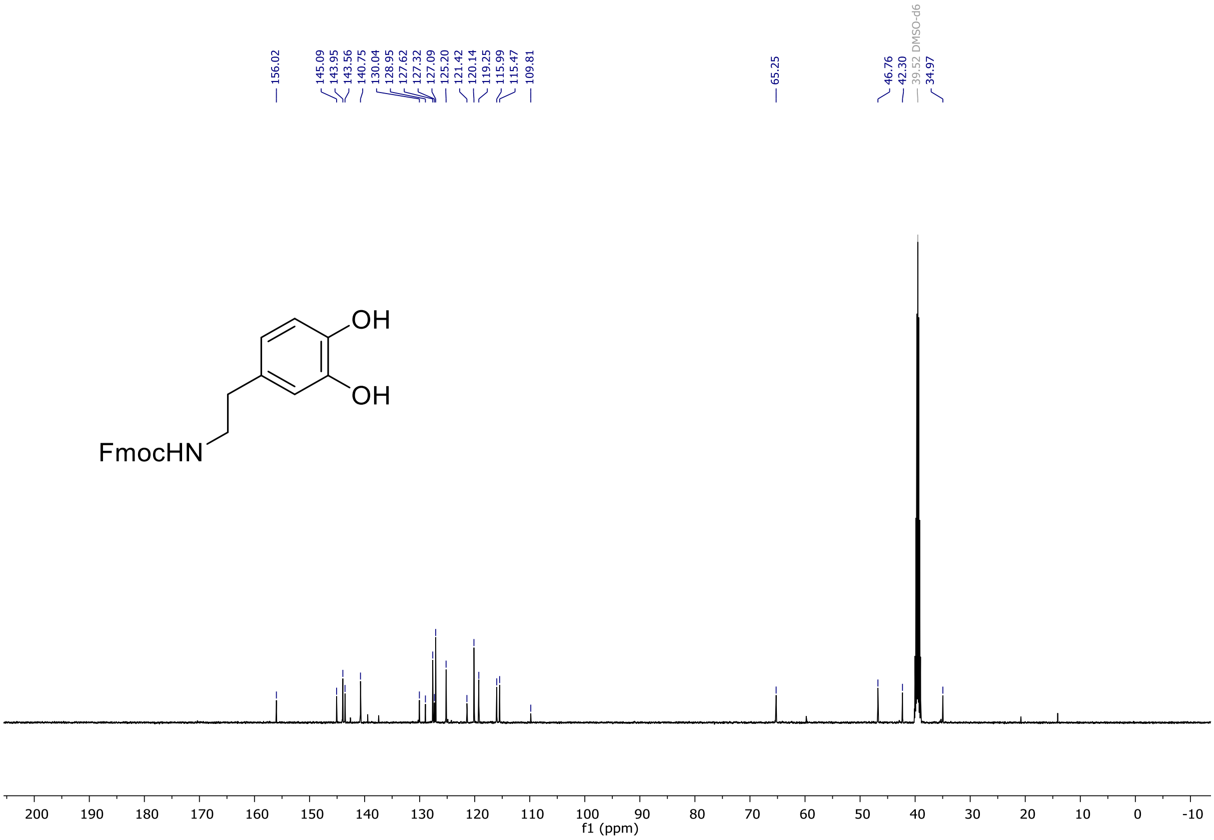

^{1}H NMR spectrum of **5h** in DMSO-d_6, 400 MHz

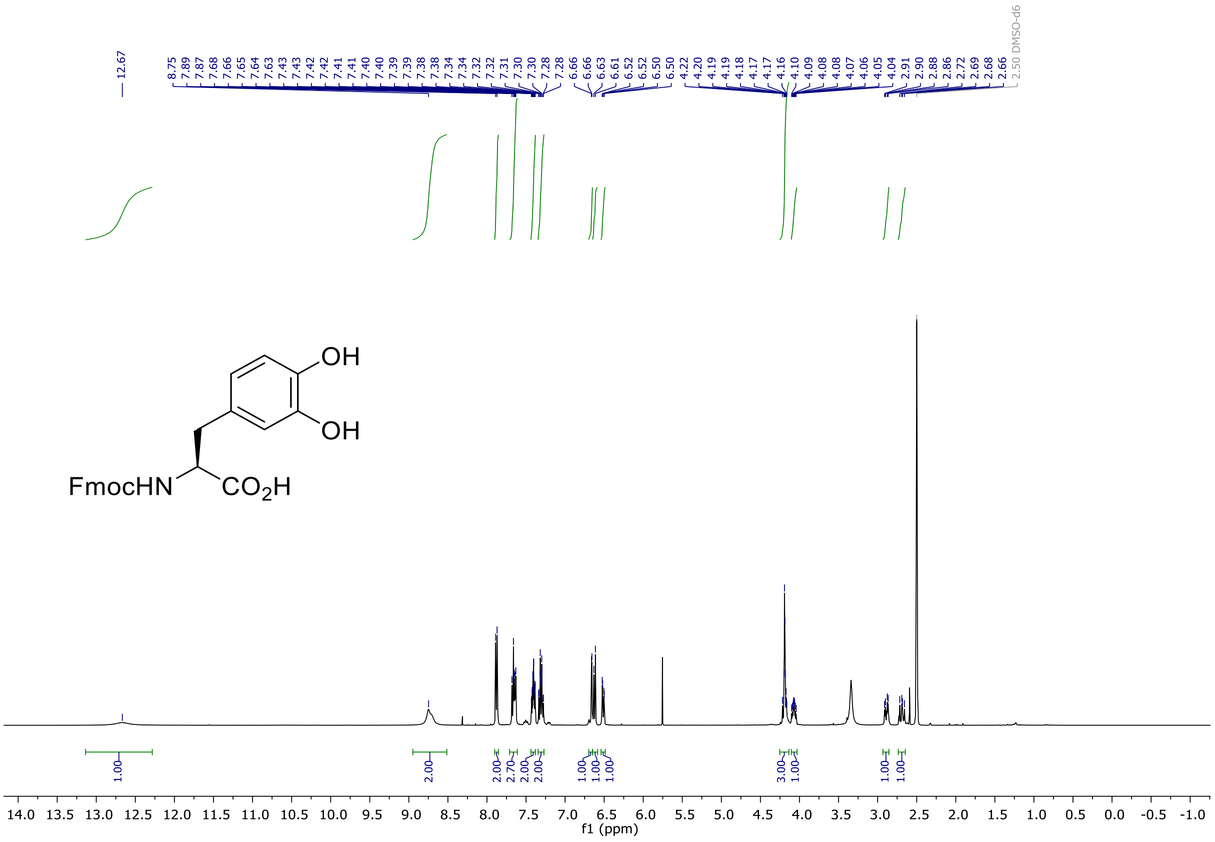

$^{13}C\{^{1}H\}$ NMR spectrum of **5h** in DMSO-d_6, 101 MHz

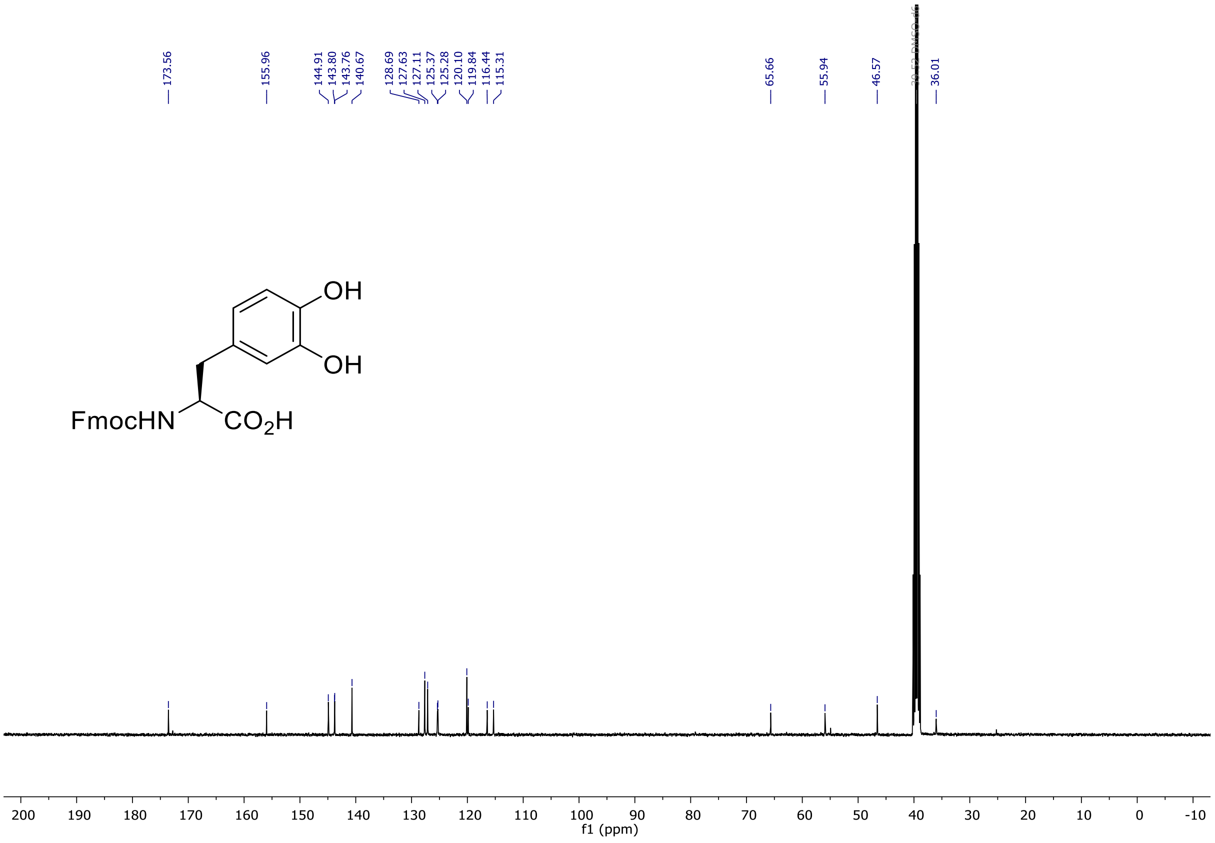

^{1}H NMR spectrum of **5i** in MeOD-d_4, 400 MHz

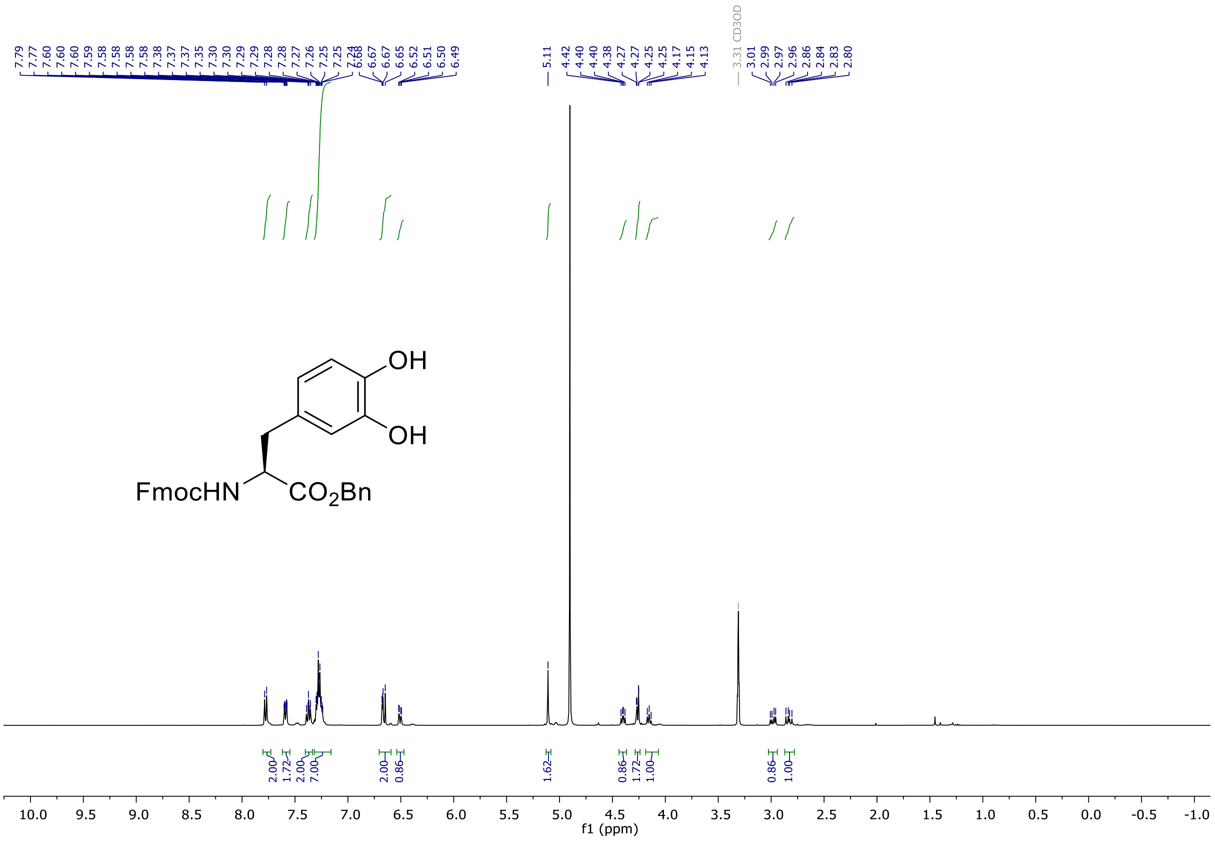

^{13}C{^{1}H} NMR spectrum of **5i** in MeOD-d_4, 101 MHz

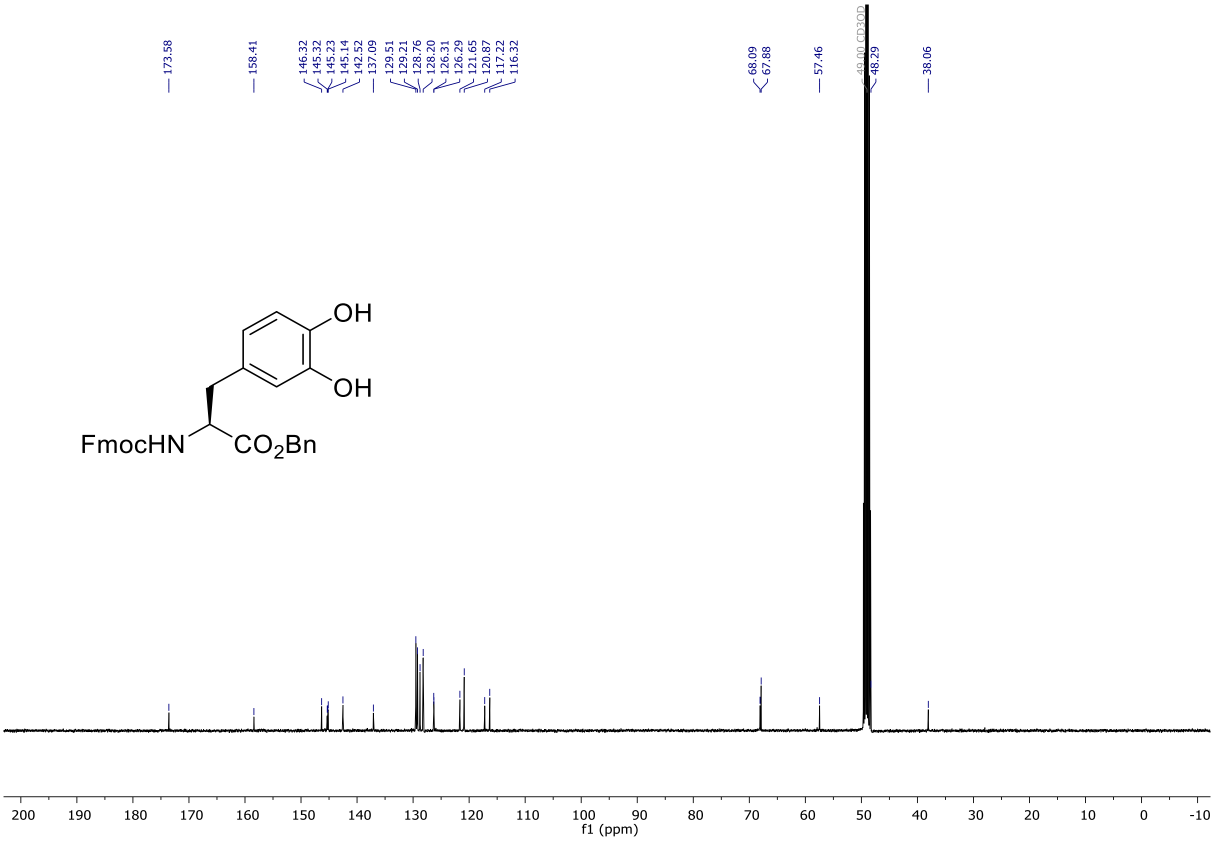

^{1}H NMR spectrum of **7a** in $CDCl_3$, 400 MHz

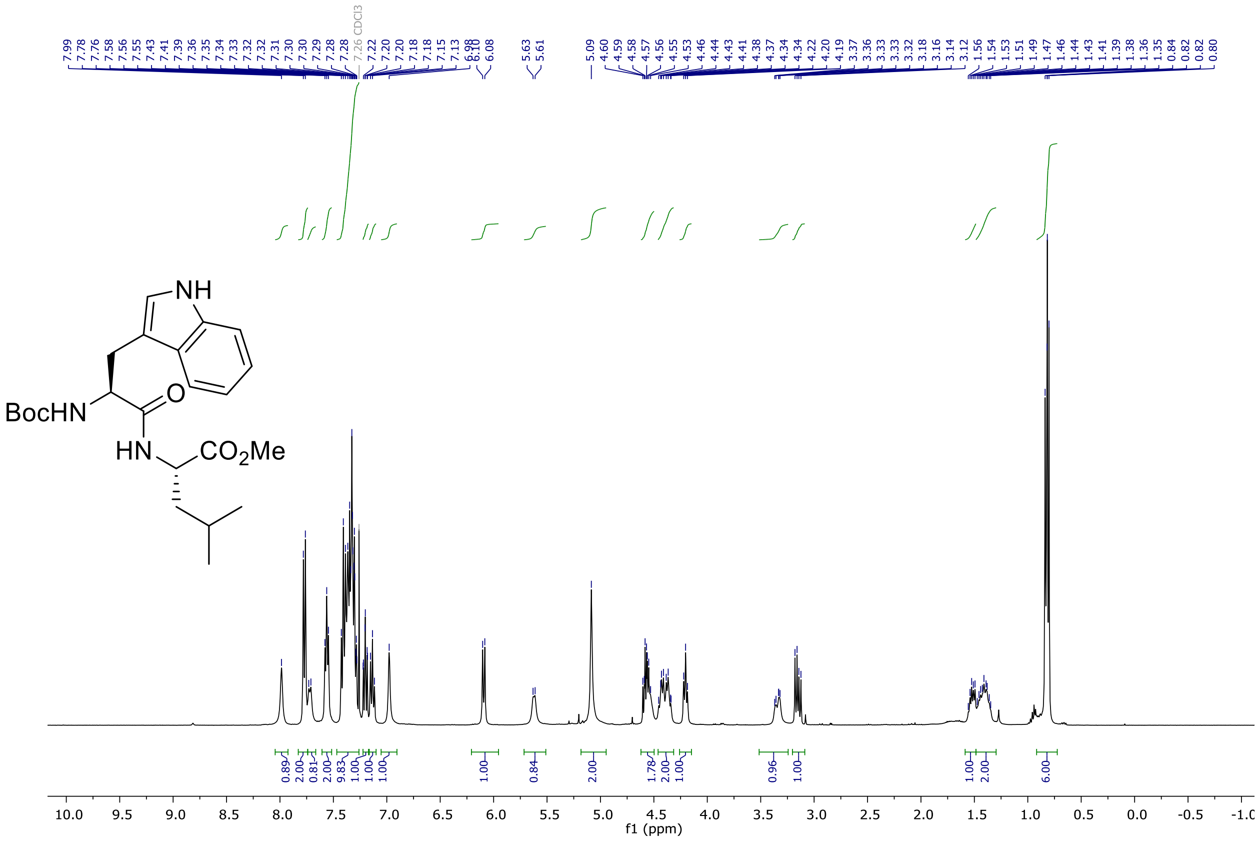

^{13}C{^{1}H} NMR spectrum of **7a** in $CDCl_3$, 101 MHz

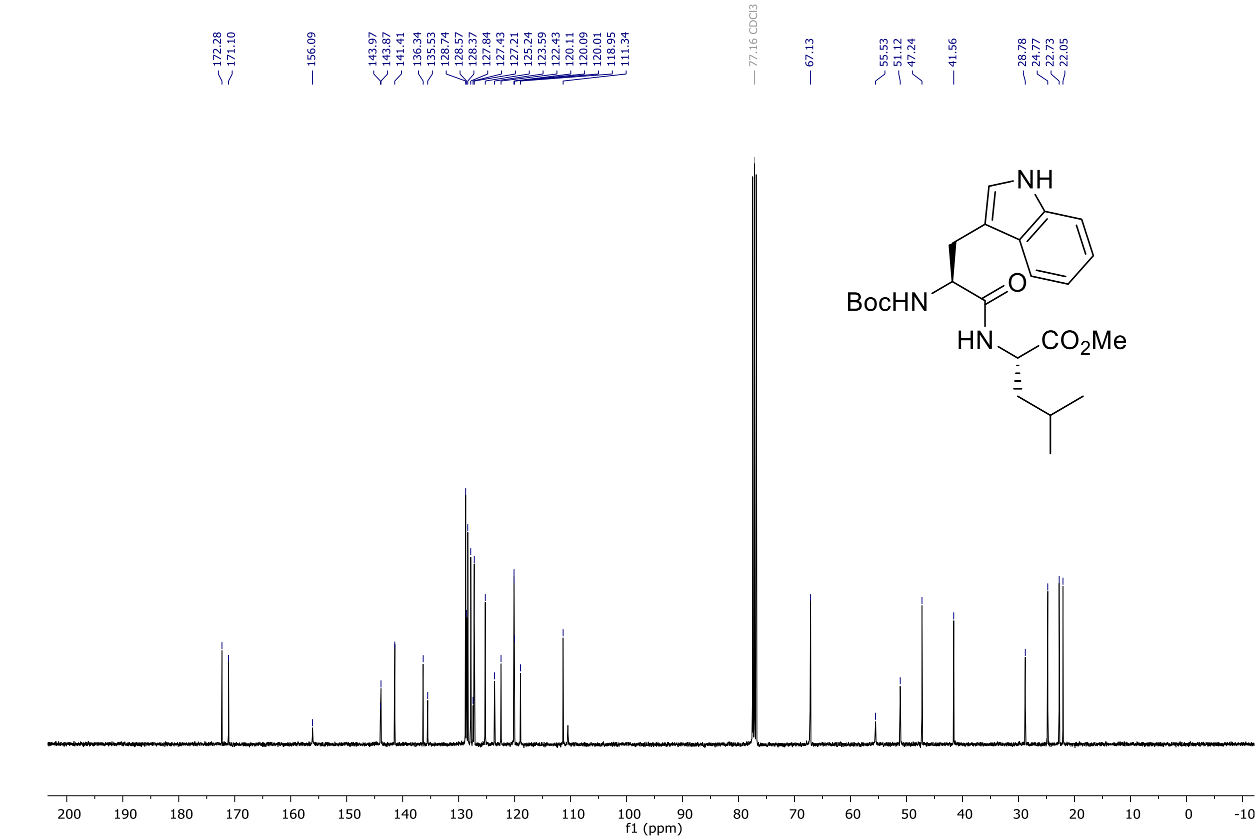

^{1}H NMR spectrum of **7b'** in $CDCl_3$, 500 MHz

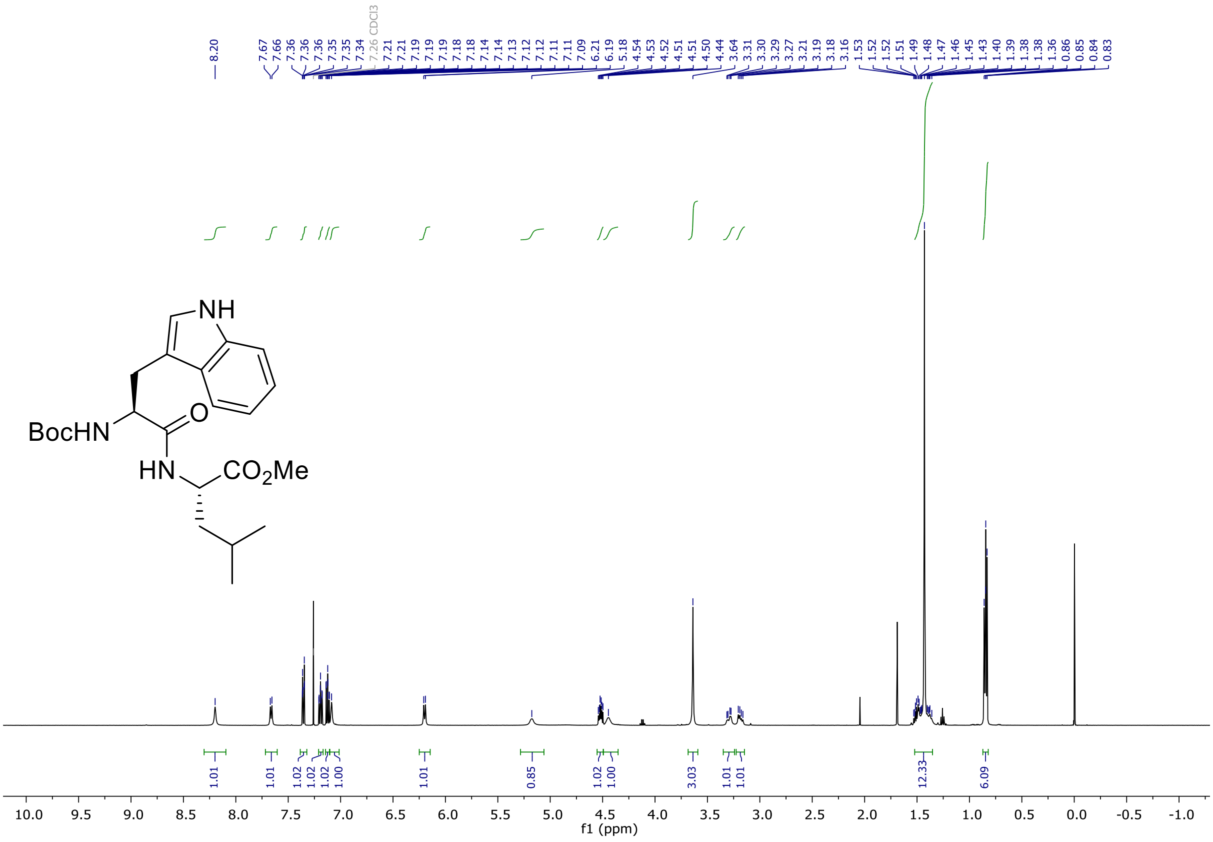

^{13}C{^{1}H} NMR spectrum of **7b'** in $CDCl_3$, 126 MHz

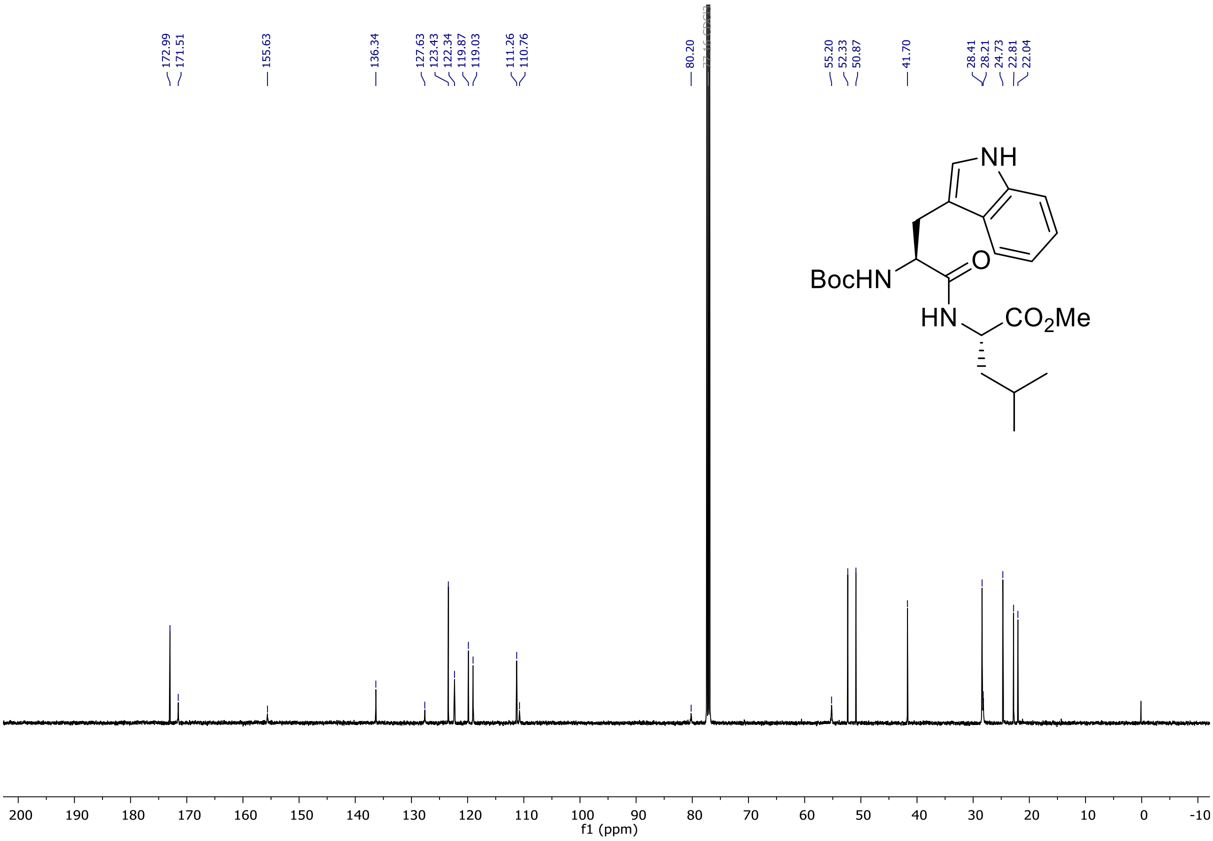

^{1}H NMR spectrum of **7b** in $CDCl_3$, 500 MHz

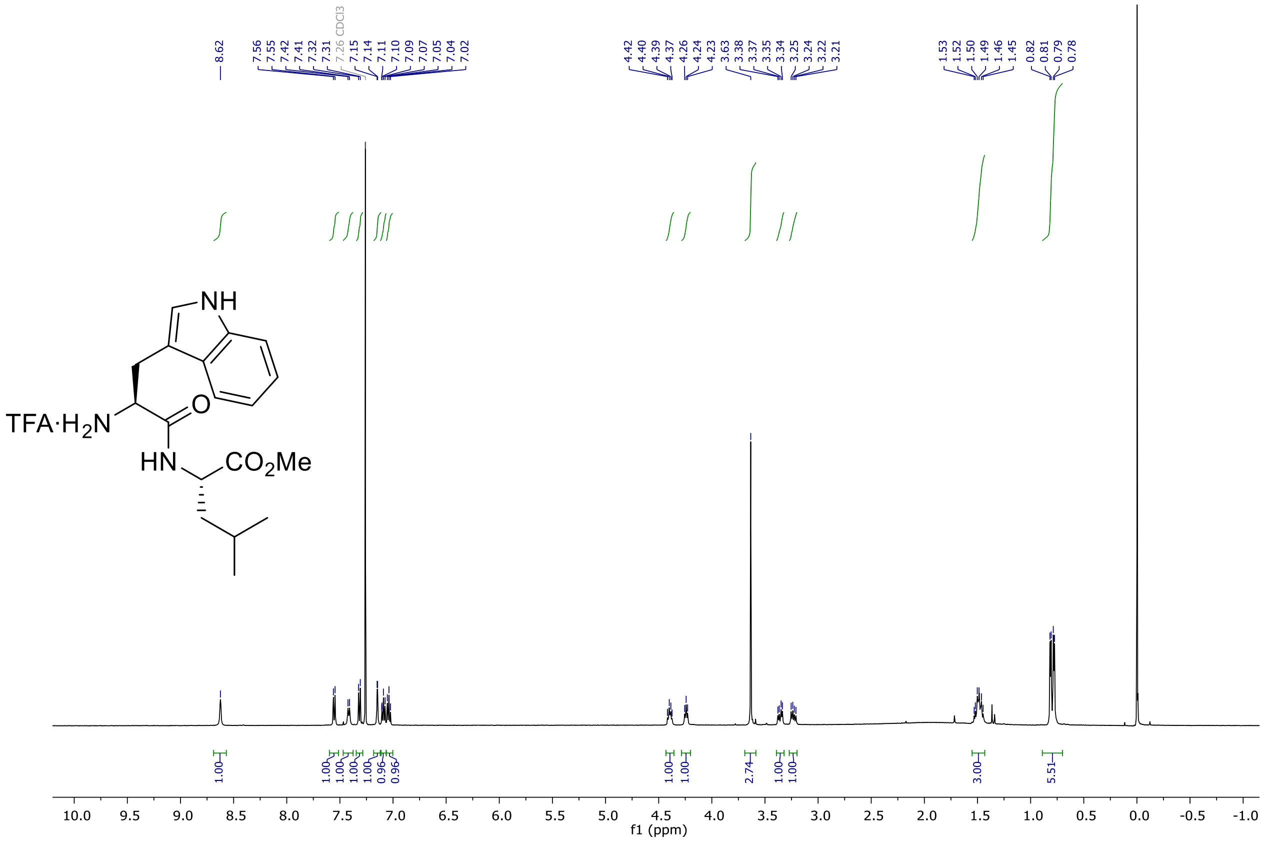

$^{13}C\{^{1}H\}$ NMR spectrum of **7b** in $CDCl_3$, 126 MHz

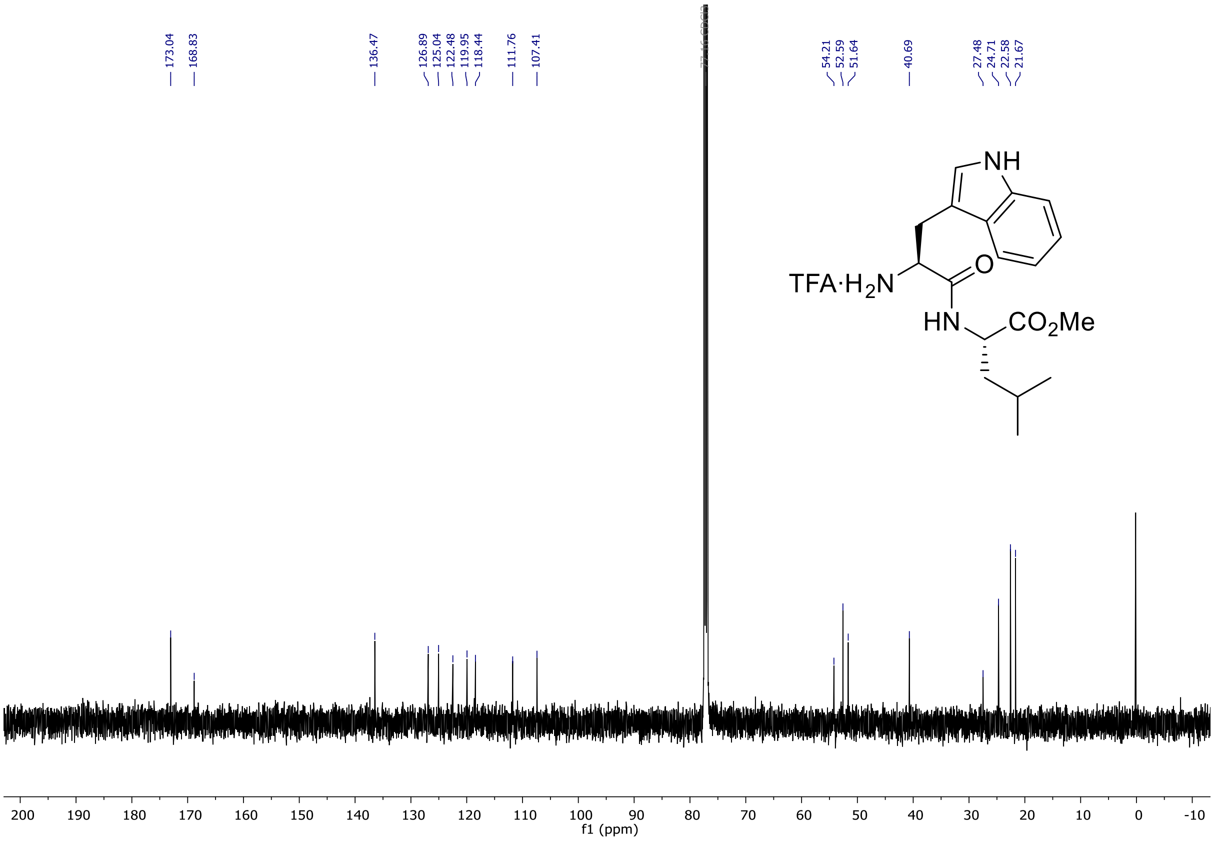

^{1}H NMR spectrum of **7c** in $CDCl_3$, 400 MHz

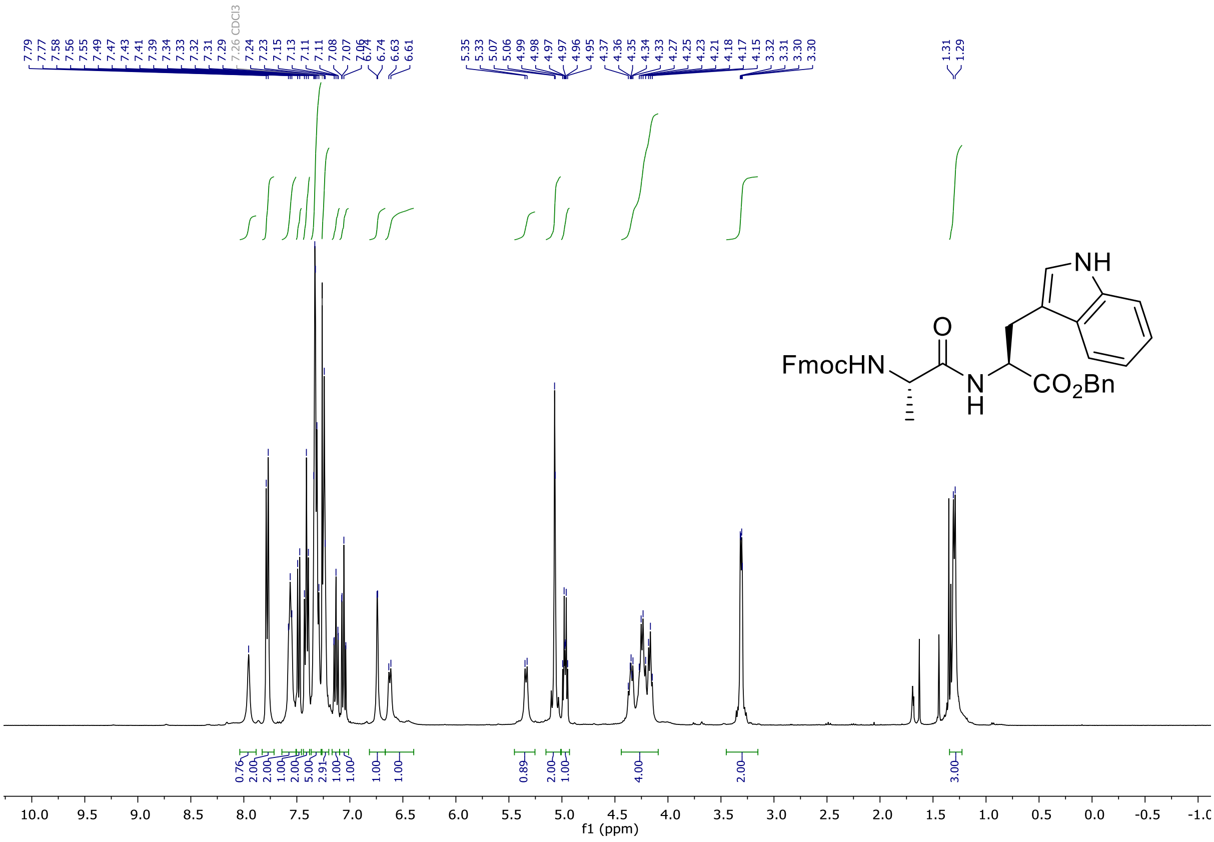

$^{13}C\{^{1}H\}$ NMR spectrum of **7c** in $CDCl_3$, 101 MHz

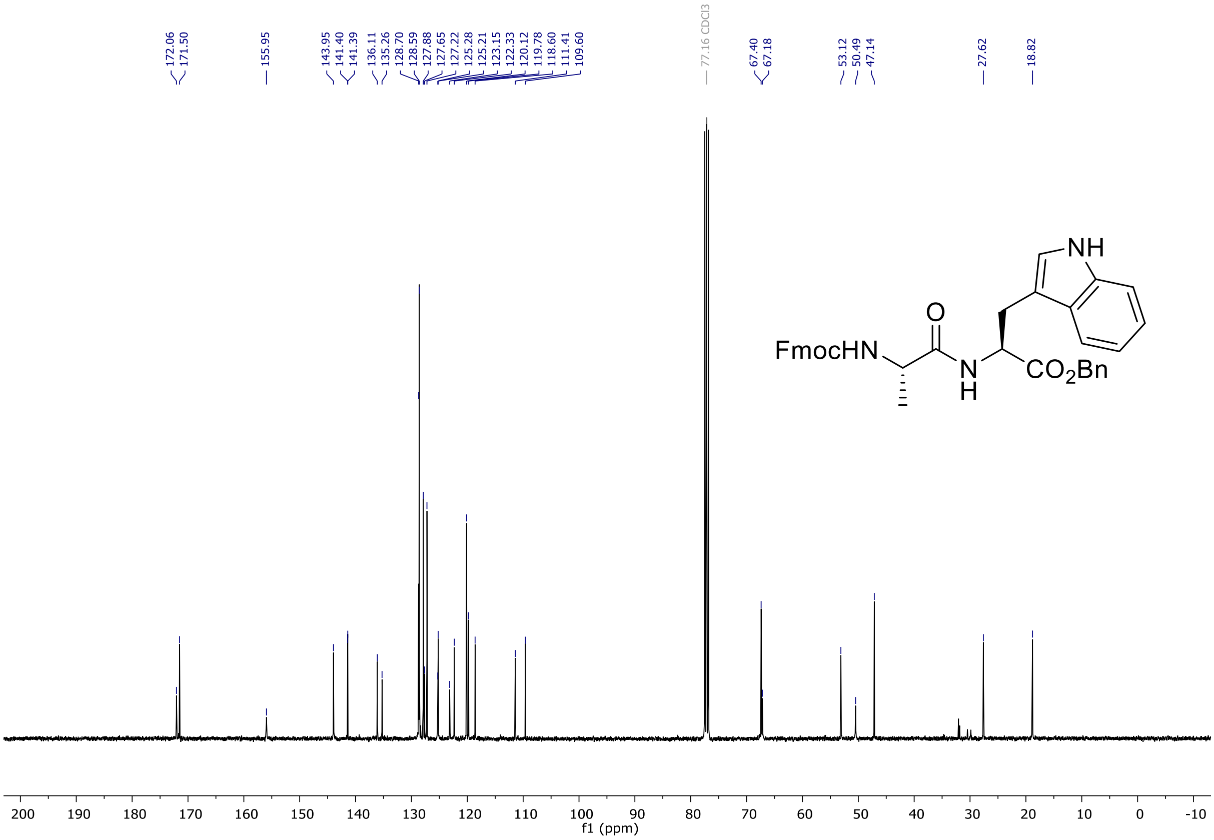

^{1}H NMR spectrum of **7d** in $CDCl_3$, 500 MHz

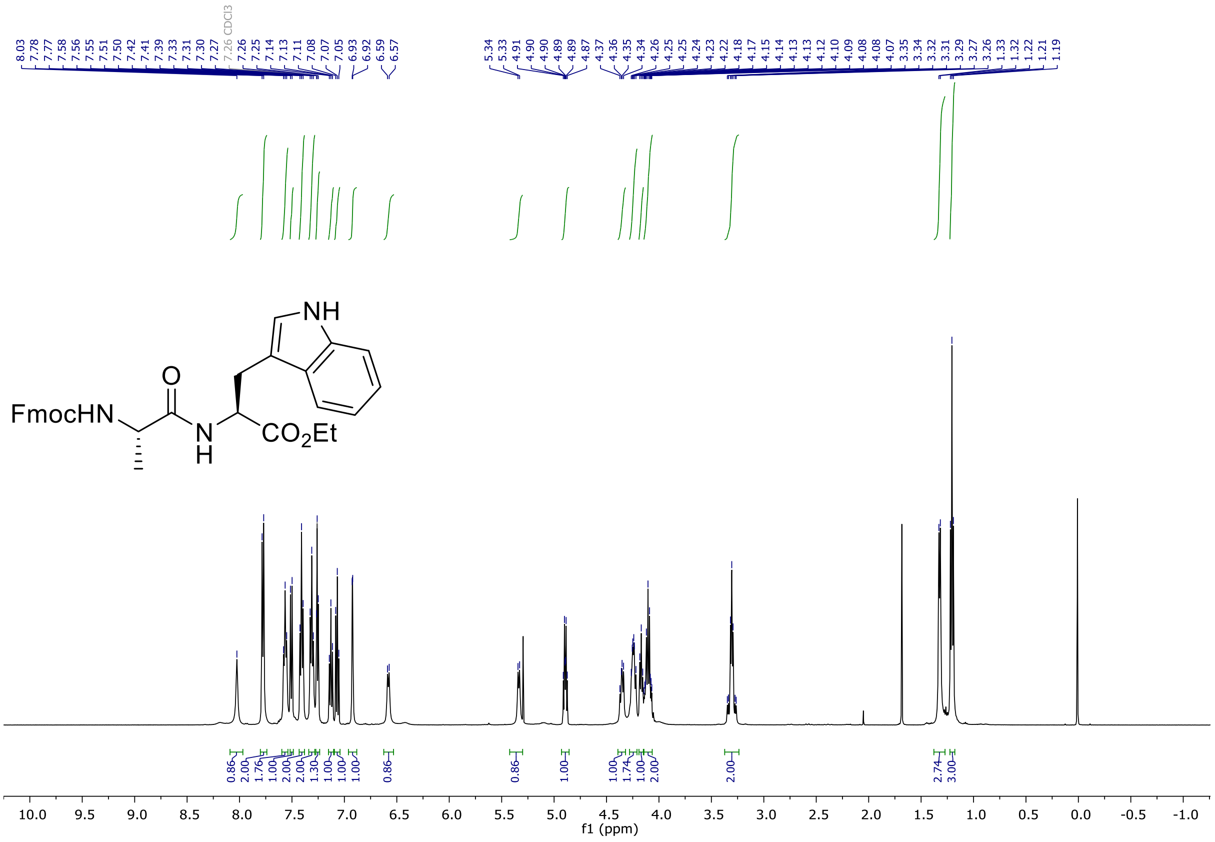

^{13}C{^{1}H} NMR spectrum of **7d** in $CDCl_3$, 126 MHz

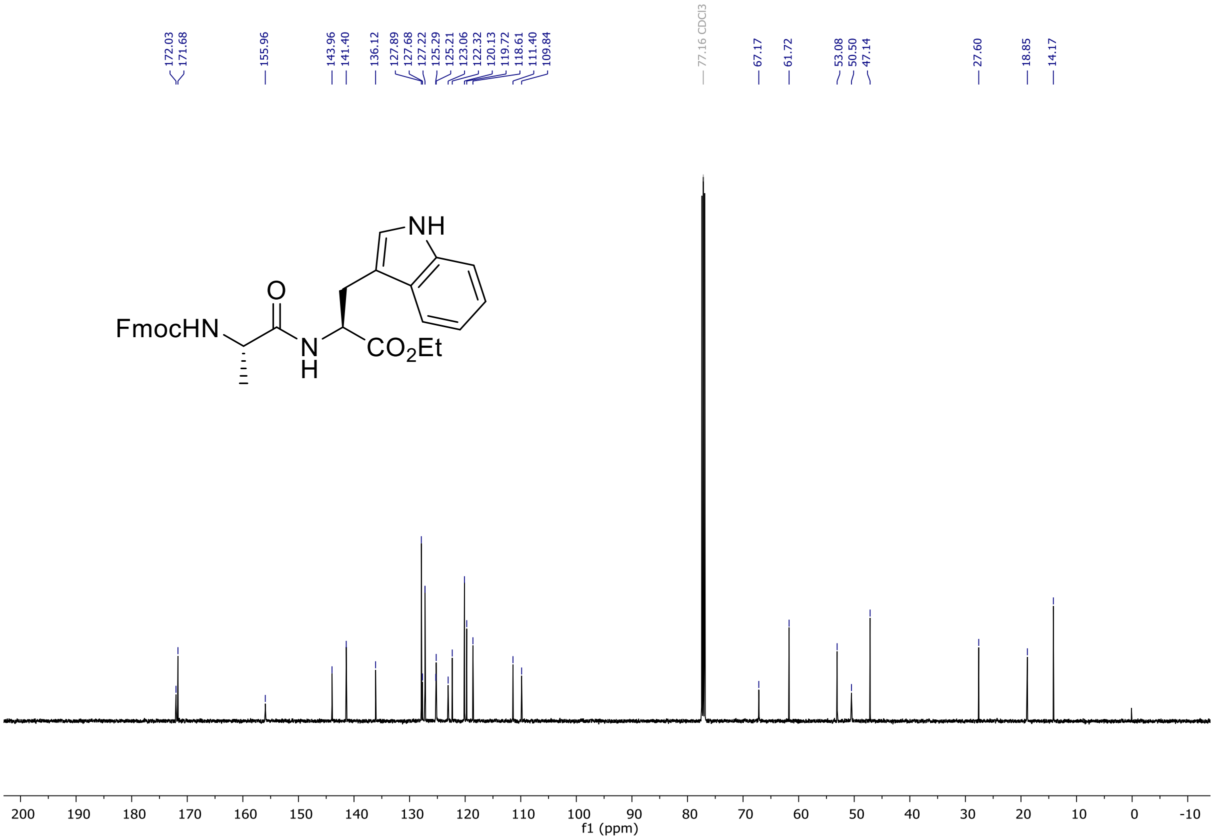

^{1}H NMR spectrum of **7e** in $CDCl_3$, 500 MHz

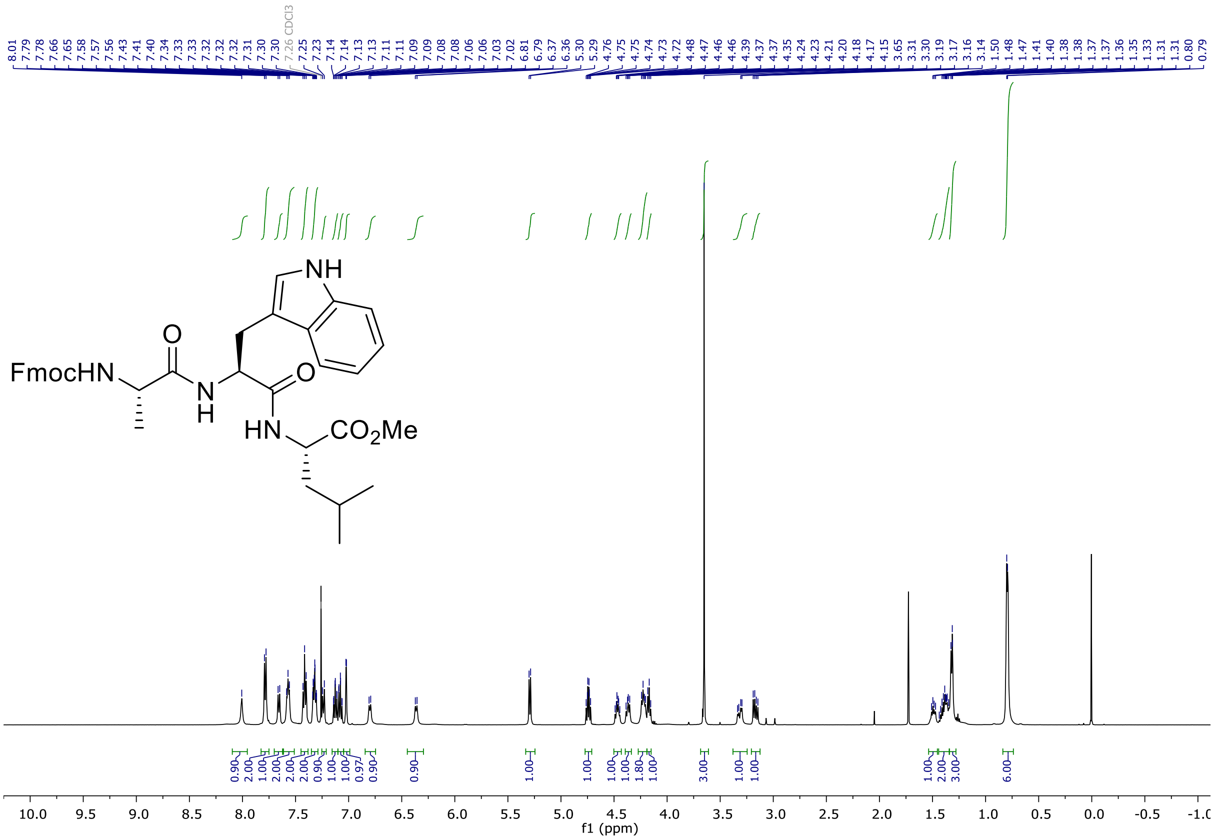

^{13}C{^{1}H} NMR spectrum of **7e** in $CDCl_3$, 126 MHz

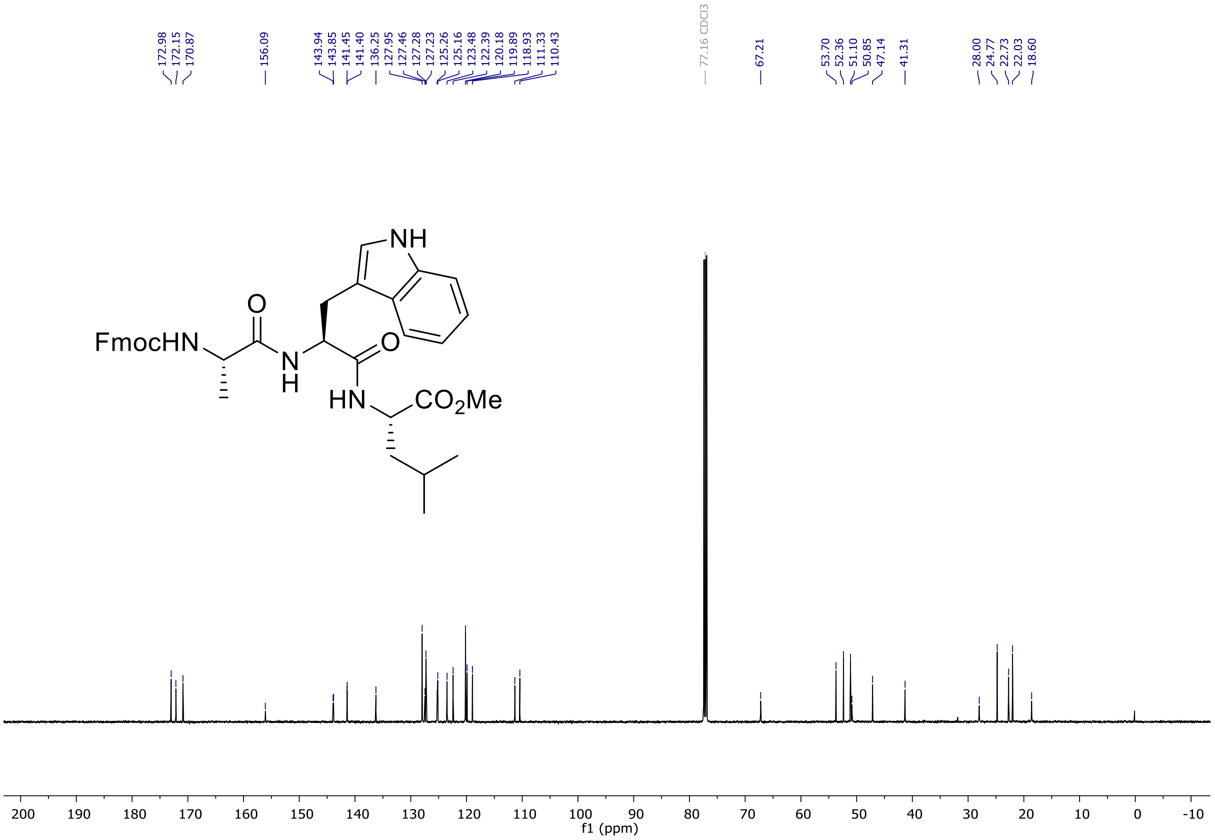

^{1}H NMR spectrum of **7f** in $CDCl_3$, 400 MHz

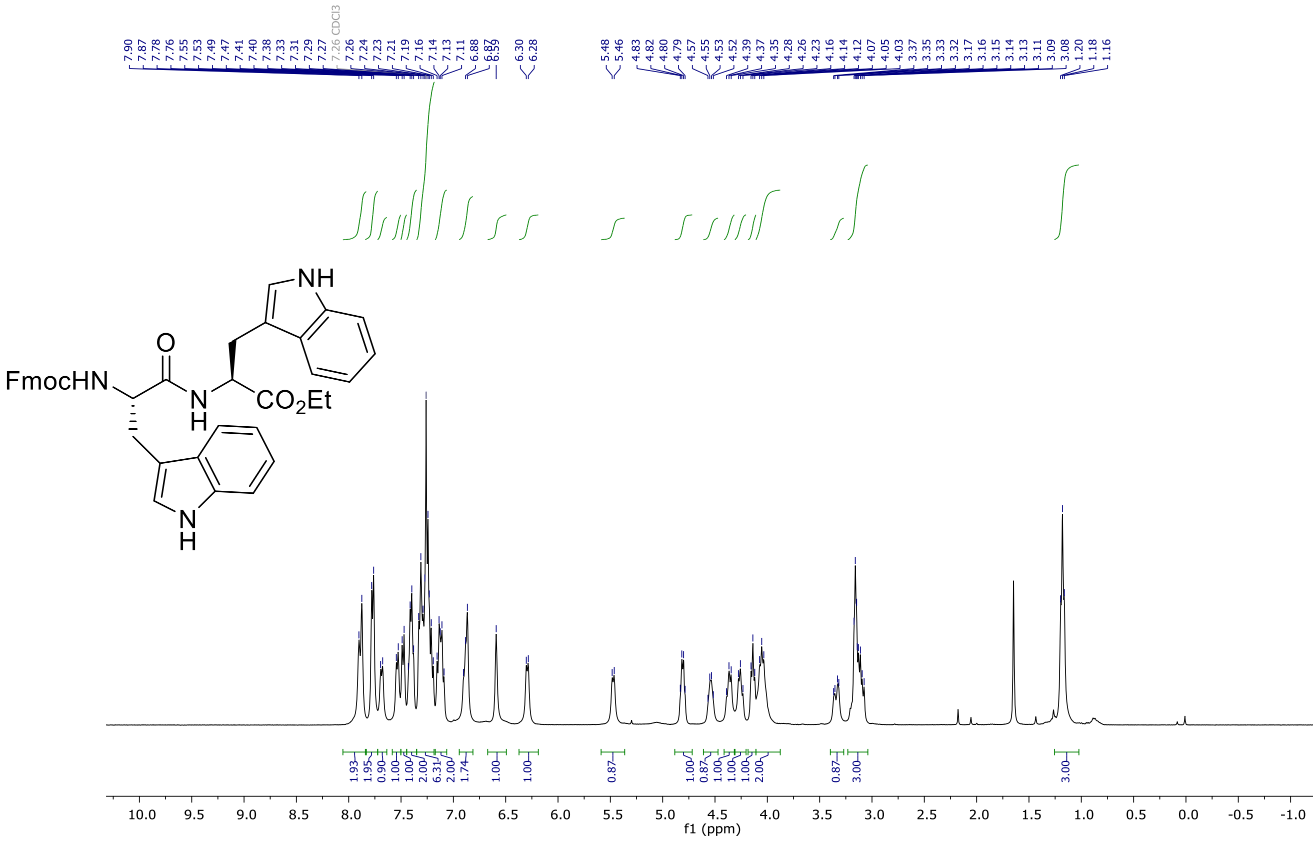

$^{13}C\{^{1}H\}$ NMR spectrum of **7f** in $CDCl_3$, 101 MHz

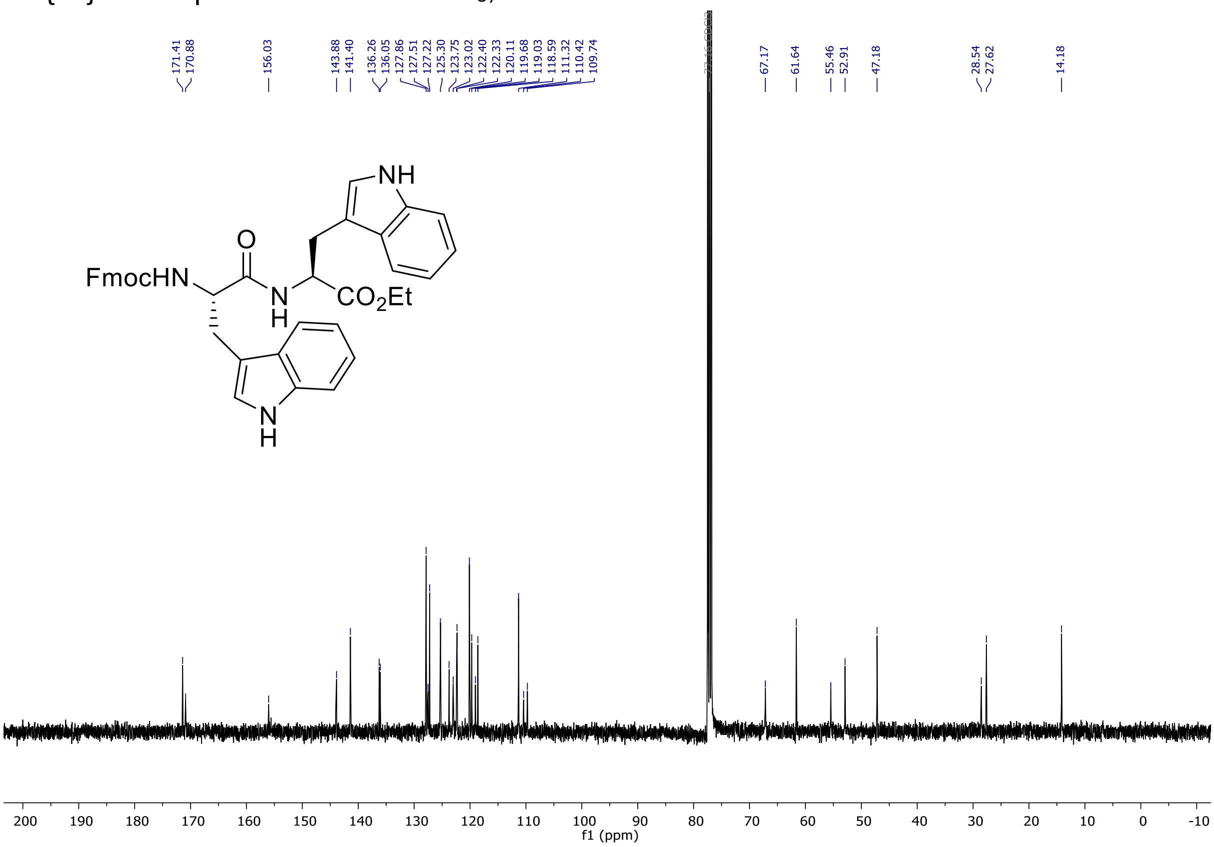

^{1}H NMR spectrum of **7g** in DMSO-d_6, 500 MHz

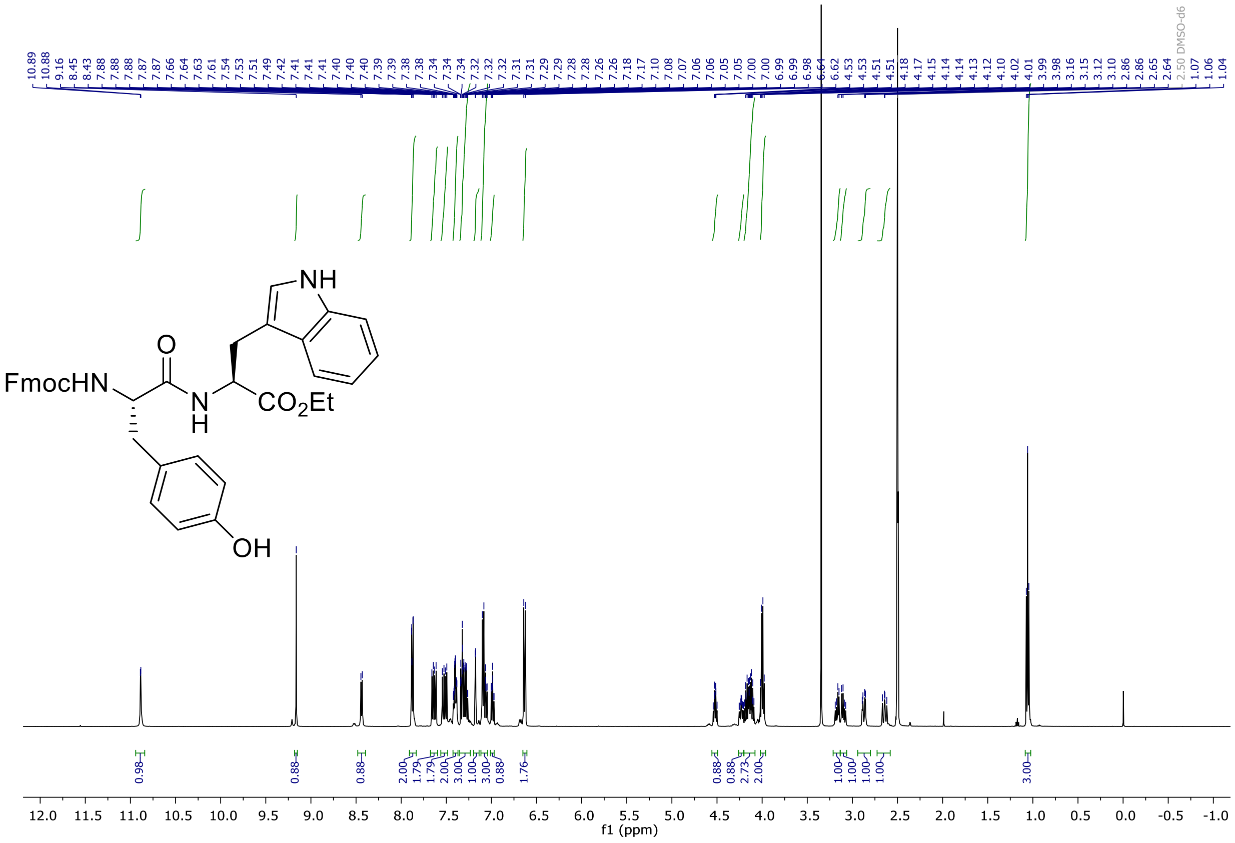

^{13}C{^{1}H} NMR spectrum of **7g** in DMSO-d_6, 126 MHz

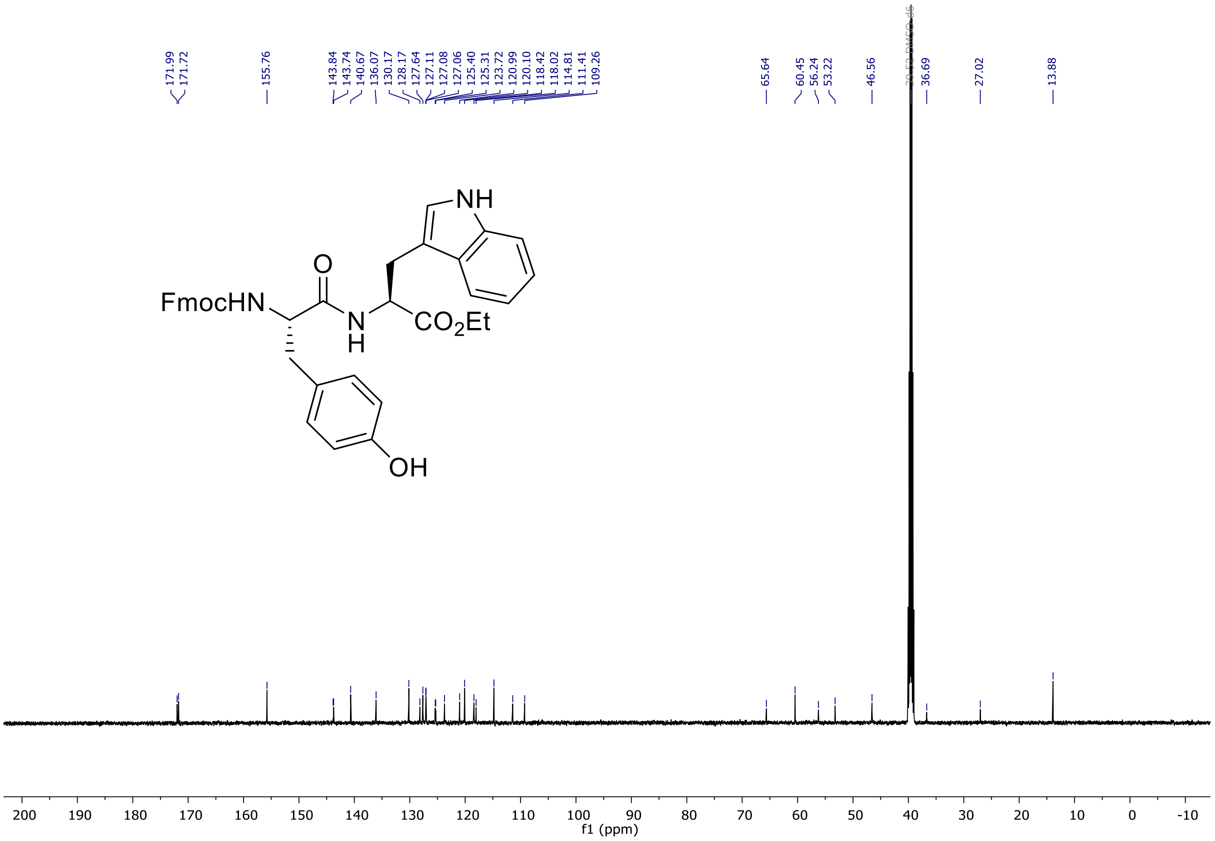

^{1}H NMR spectrum of **7h** in $CDCl_3$, 400 MHz

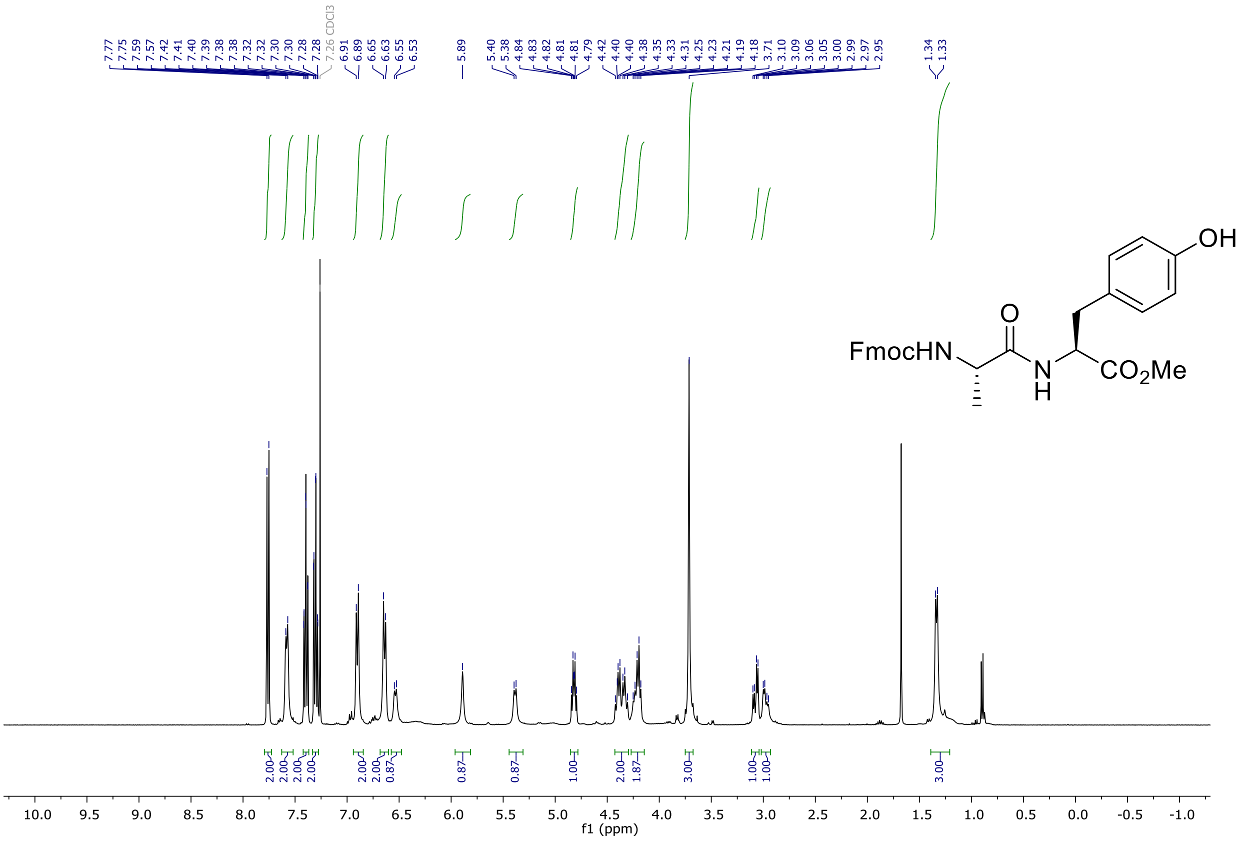

^{13}C{^{1}H} NMR spectrum of **7h** in $CDCl_3$, 101 MHz

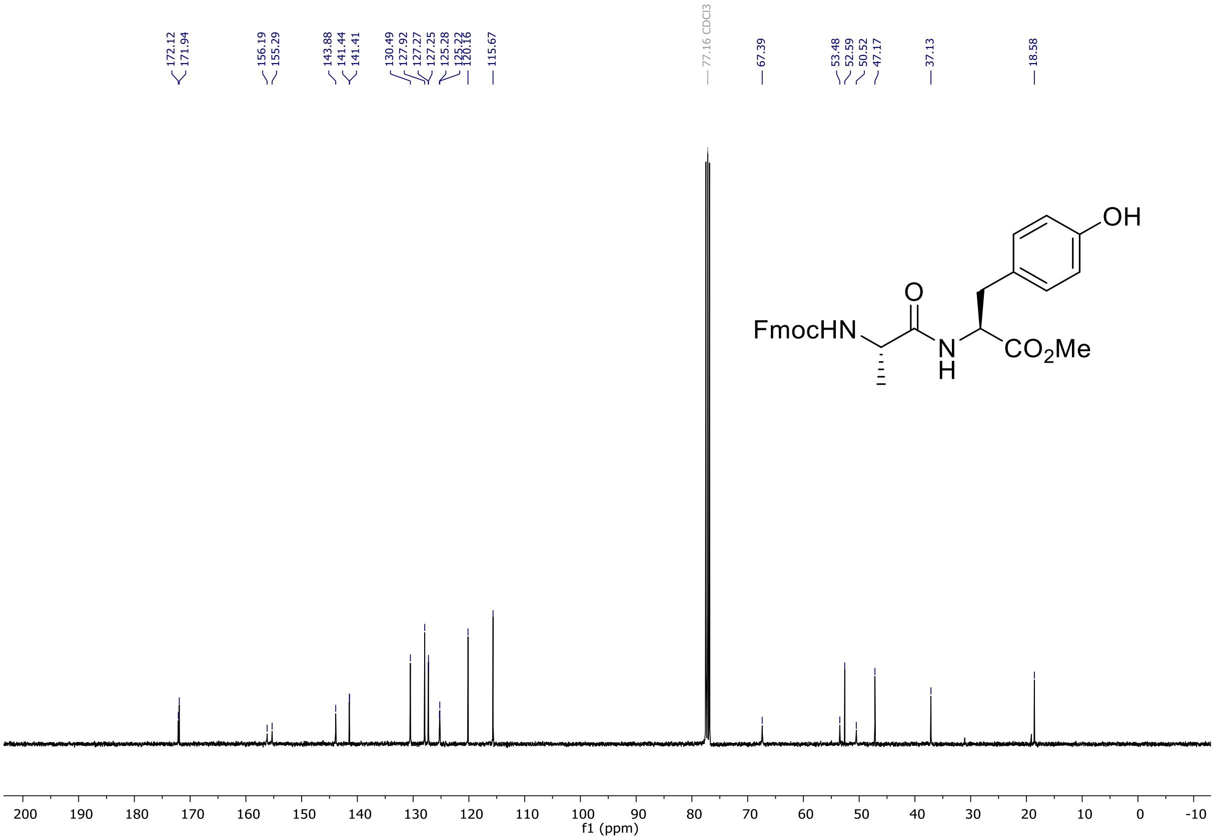

8.2. NMR spectra of new compounds

^{1}H NMR spectrum of ***endo-cis*-3a** in $CDCl_3$, 400 MHz

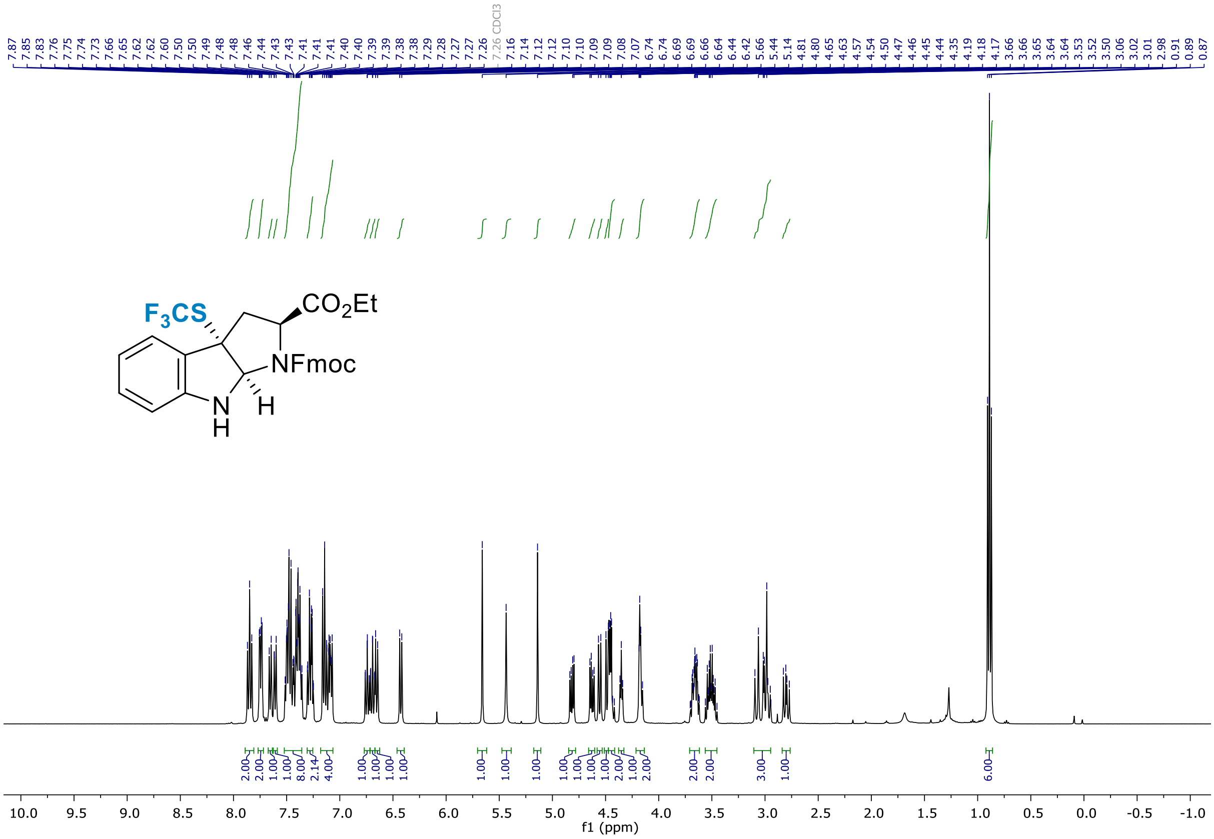

$^{13}C\{^{1}H\}$ NMR spectrum of ***endo-cis*-3a** in $CDCl_3$, 101 MHz

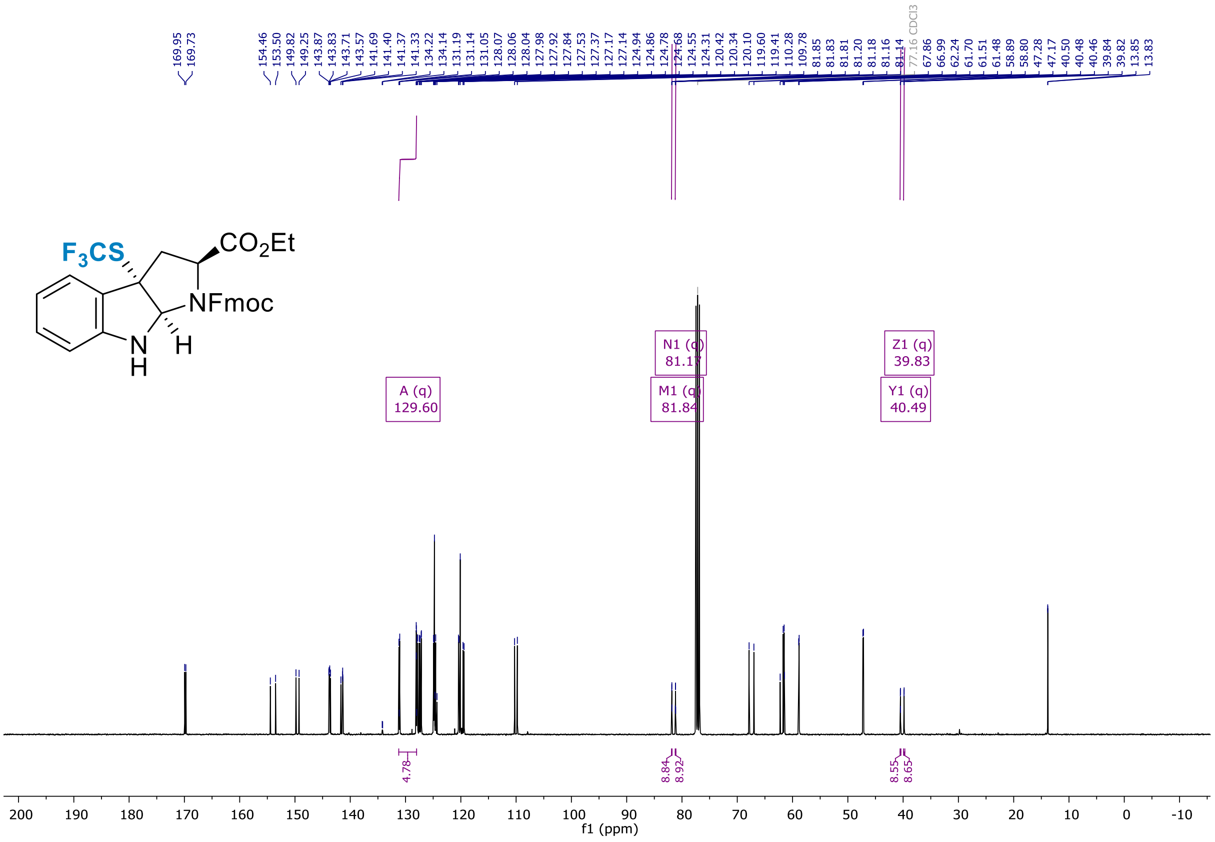

^{19}F NMR spectrum of ***endo-cis*-4a** in $CDCl_3$, 376 MHz (referenced to C_6F_6 at –161.64 ppm)

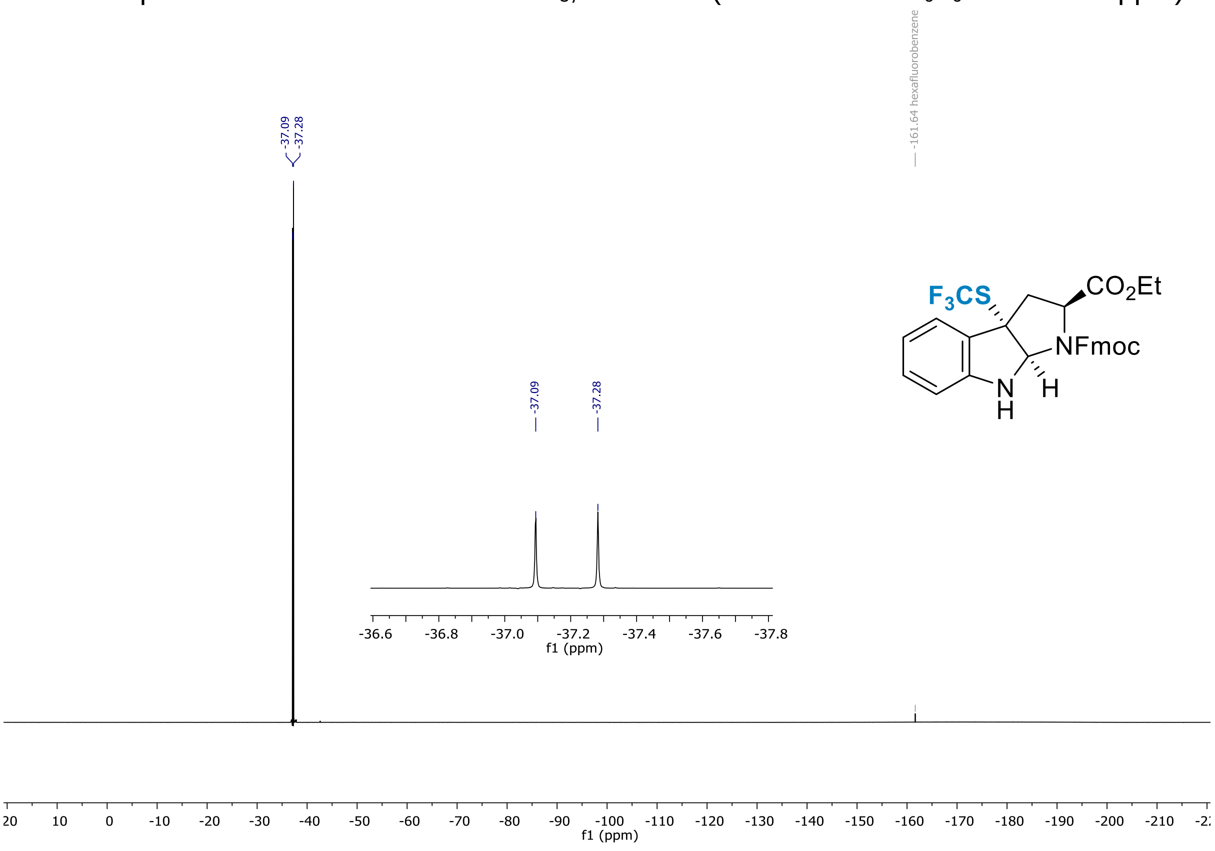

^{1}H NMR spectrum of ***exo-cis*-3a** in $CDCl_3$, 400 MHz

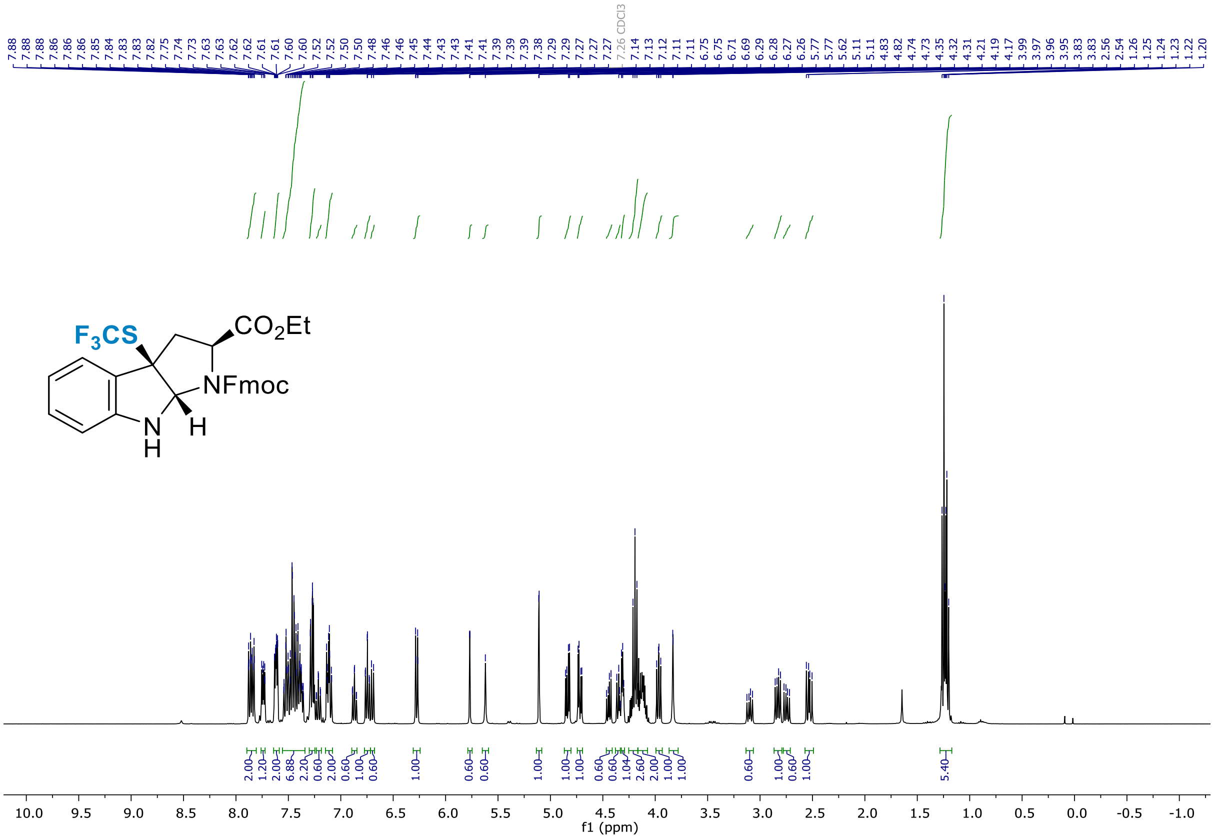

$^{13}C\{^{1}H\}$ NMR spectrum of ***exo-cis*-3a** in $CDCl_3$, 101 MHz

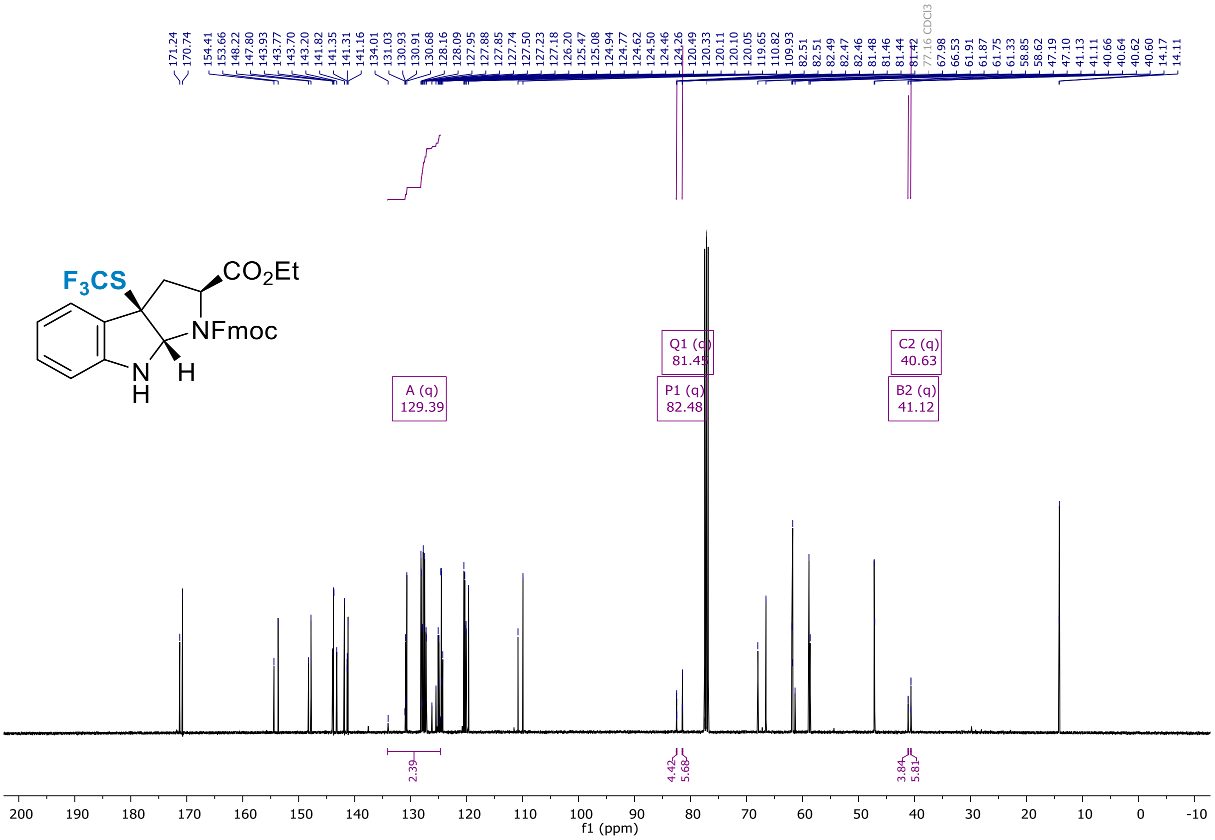

^{19}F NMR spectrum of ***exo-cis*-4a** in $CDCl_3$, 376 MHz (referenced to C_6F_6 at –161.64 ppm)

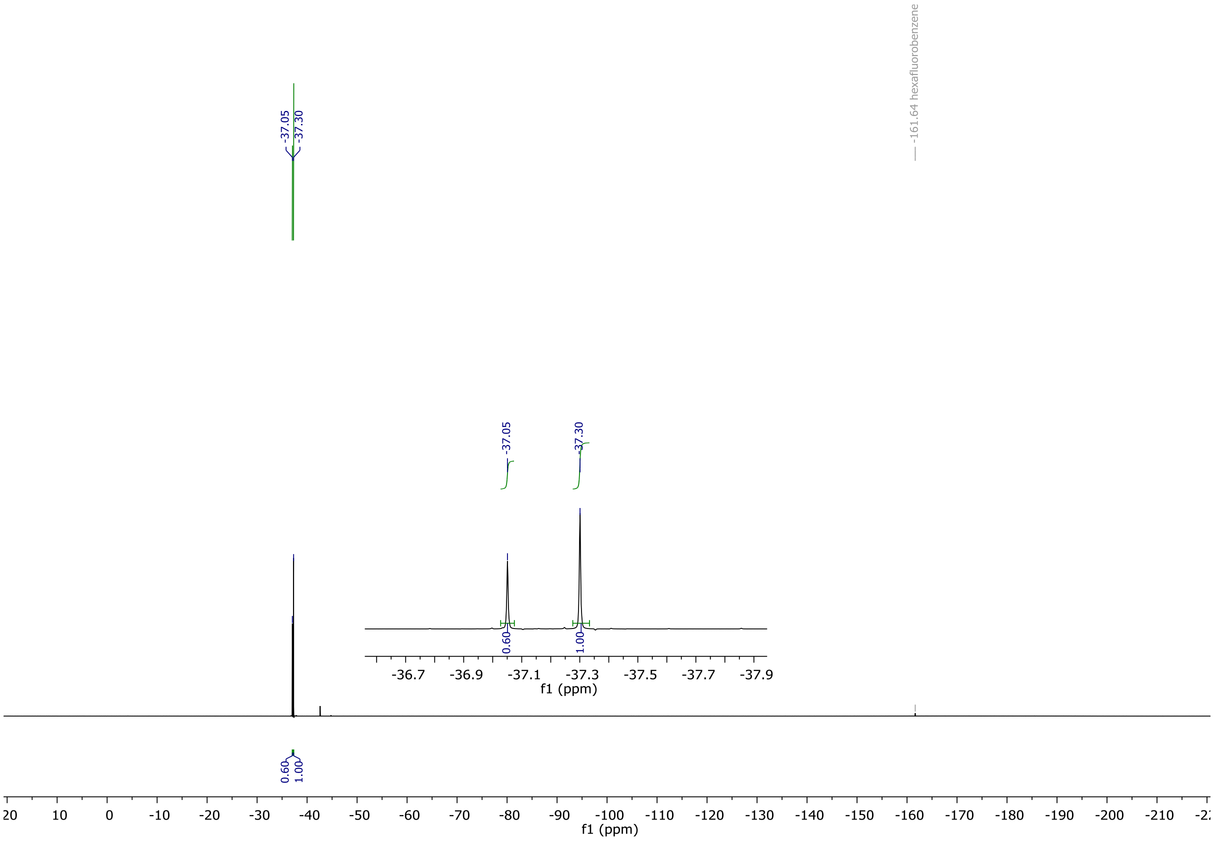

^{1}H NMR spectrum of **4a** in $CDCl_3$, 500 MHz

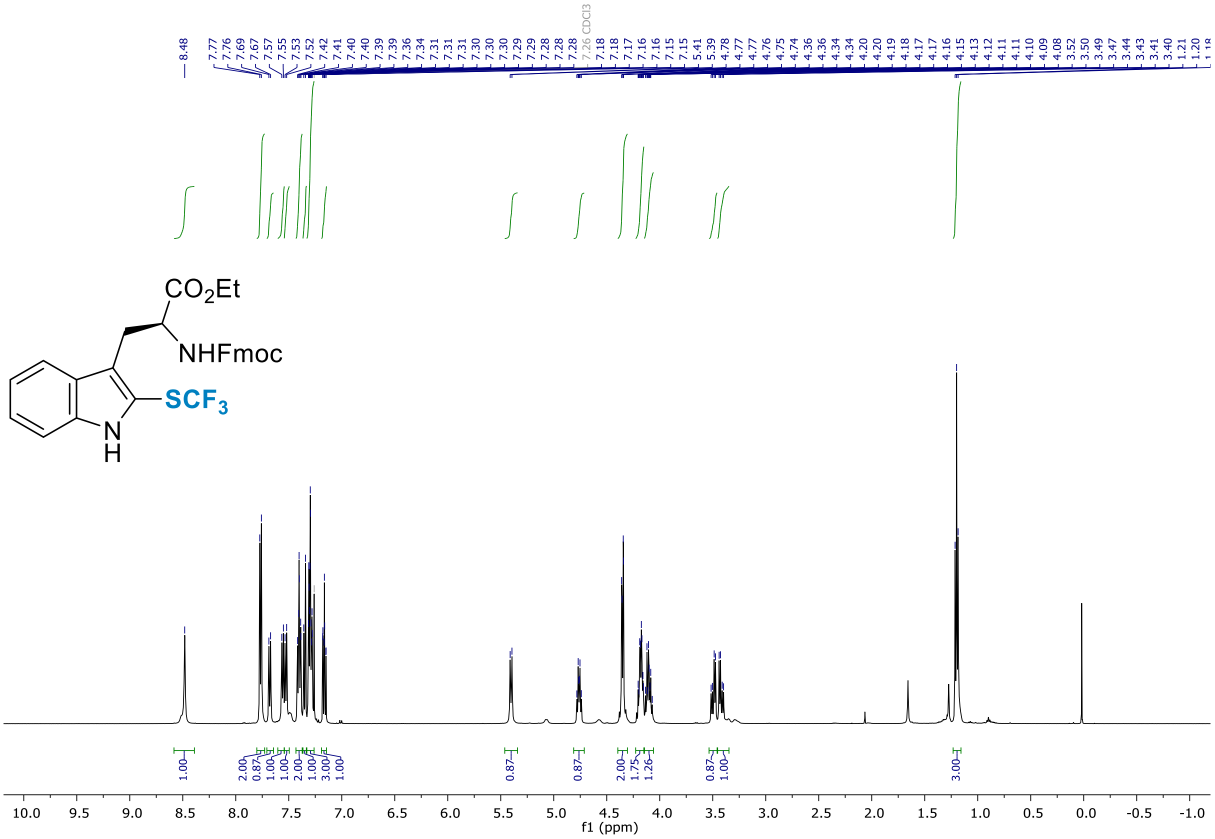

$^{13}C\{^{1}H\}$ NMR spectrum of **4a** in $CDCl_3$, 126 MHz

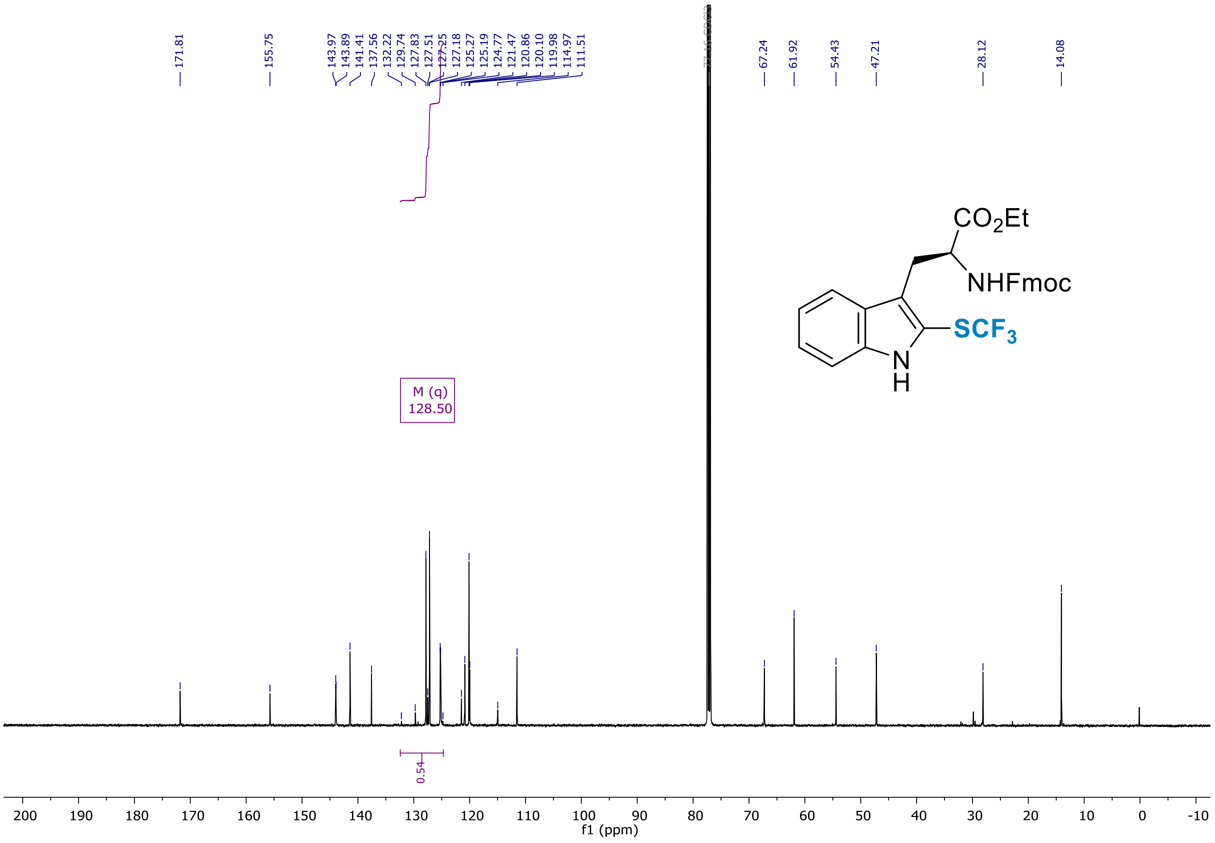

^{19}F NMR spectrum of **4a** in $CDCl_3$, 471 MHz (referenced to $CFCl_3$ at 0.65 ppm)

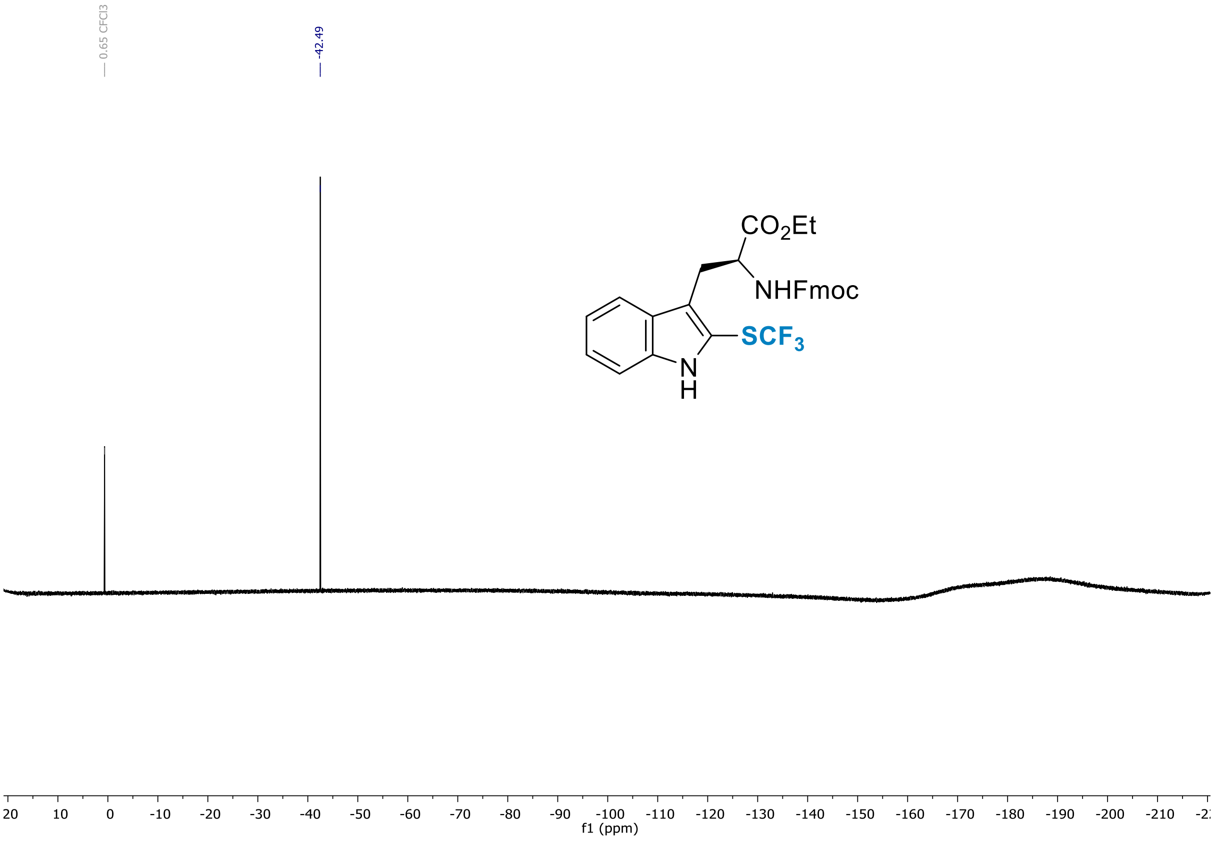

^{1}H NMR spectrum of **4b** in DMSO-d_6, 500 MHz

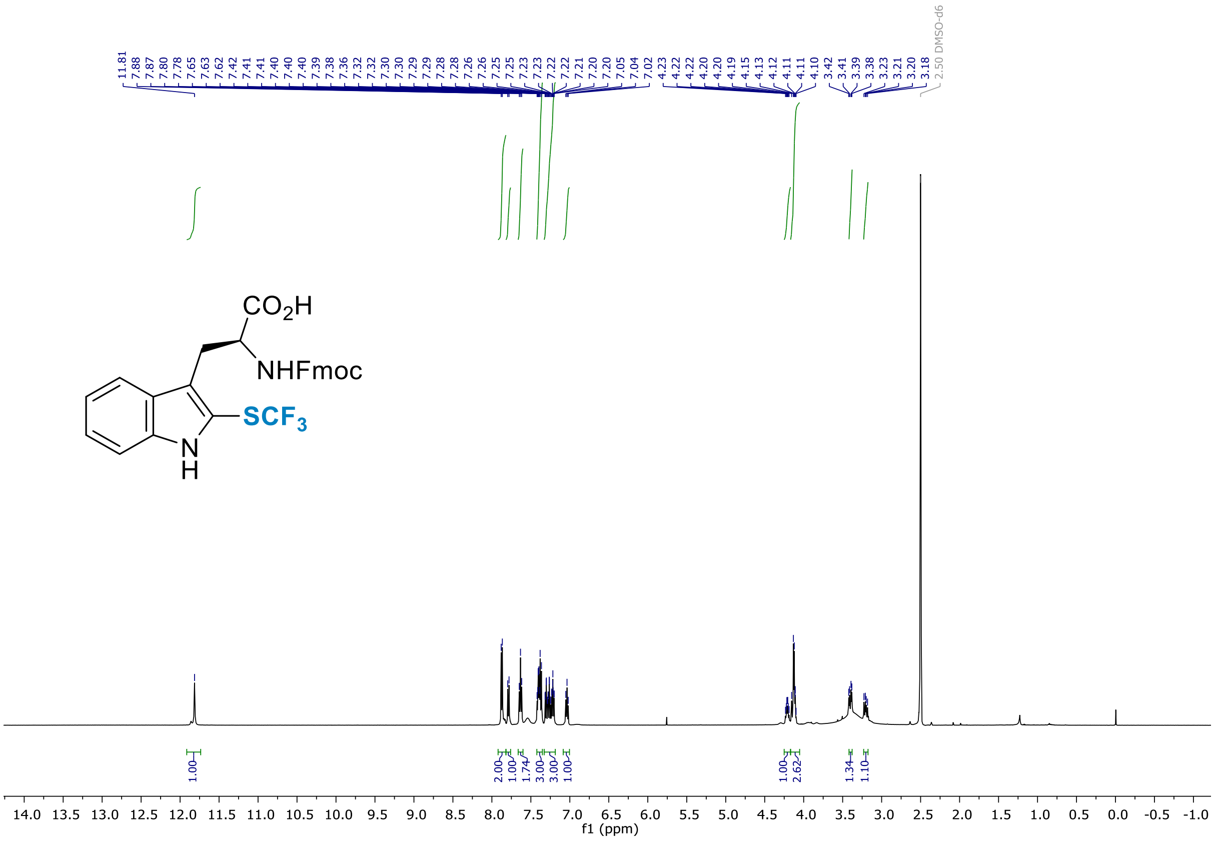

$^{13}C\{^{1}H\}$ NMR spectrum of **4b** in DMSO-d_6, 126 MHz

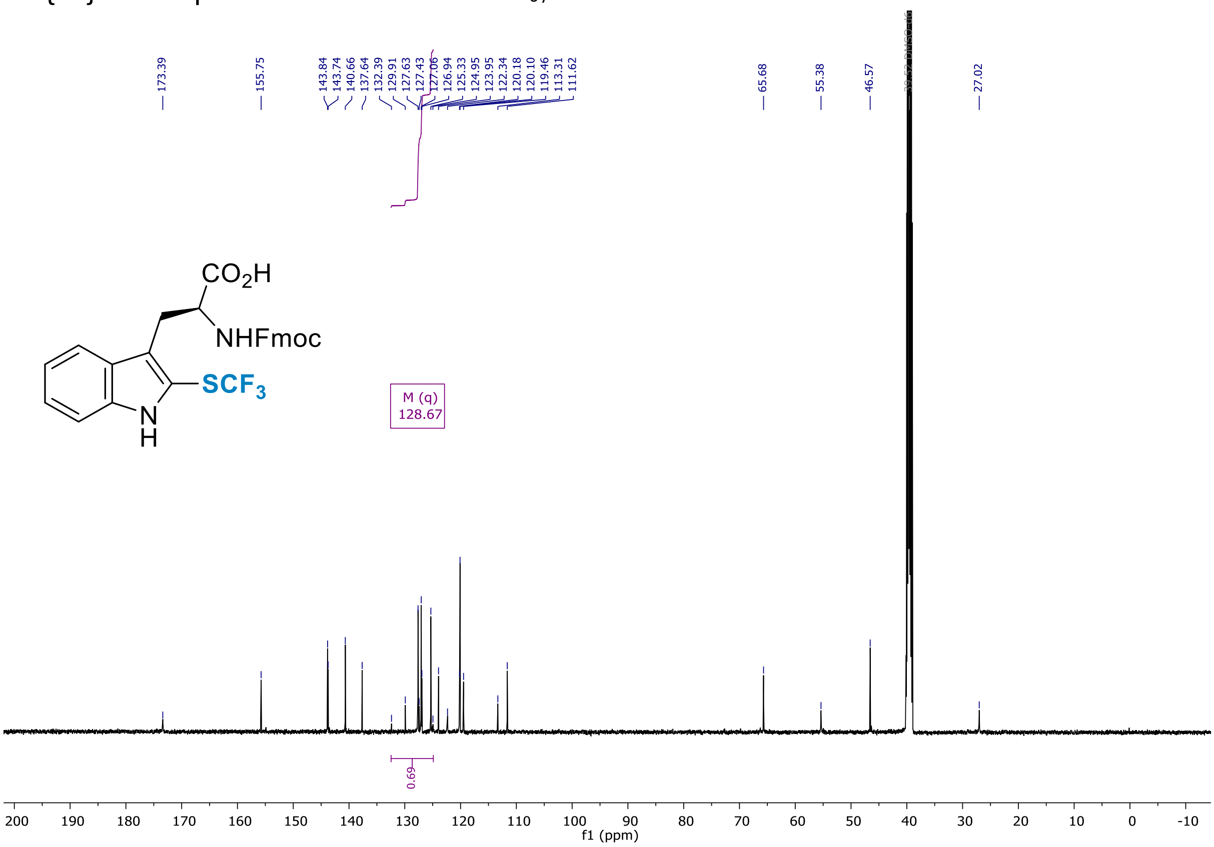

^{19}F NMR spectrum of **4b** in DMSO-d_6, 471 MHz (referenced to $CFCl_3$ at –0.24 ppm)

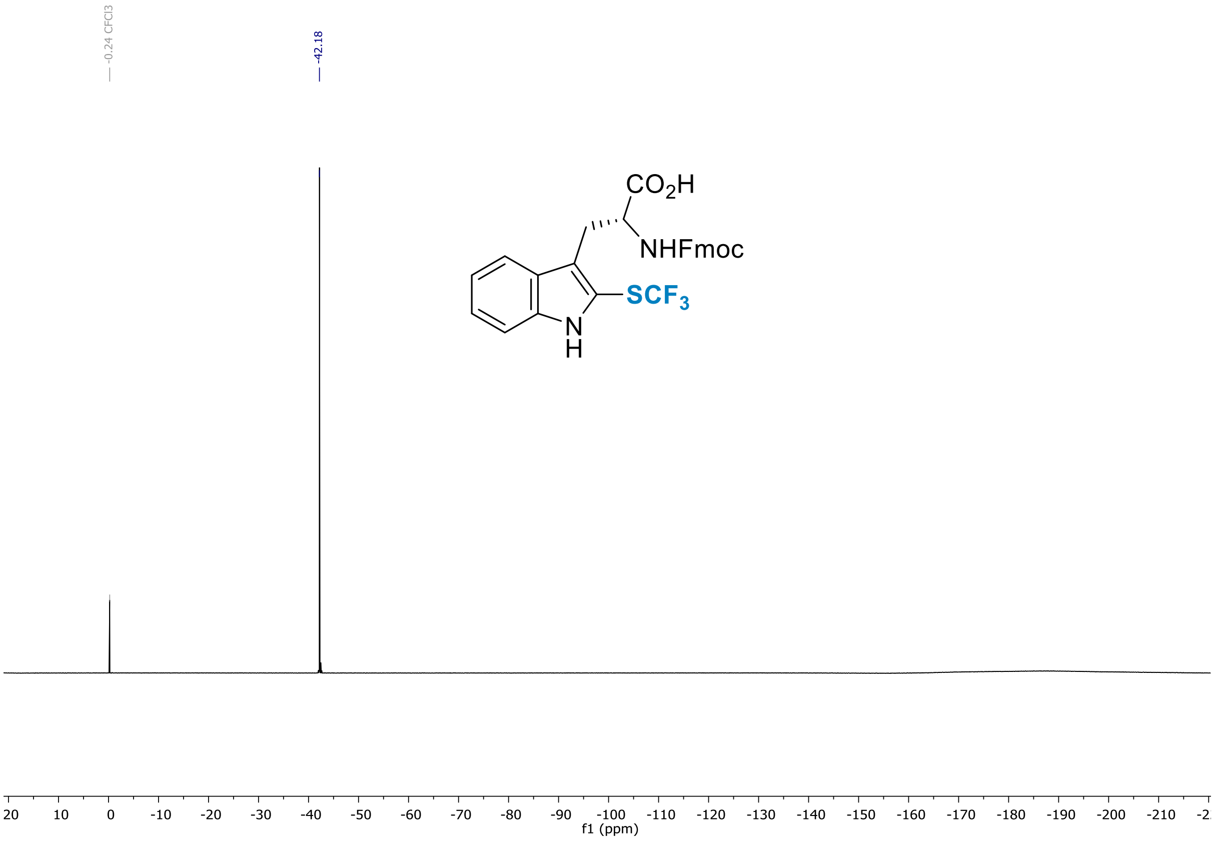

^{1}H NMR spectrum of ***(R)*-4b** in DMSO-d_6, 400 MHz

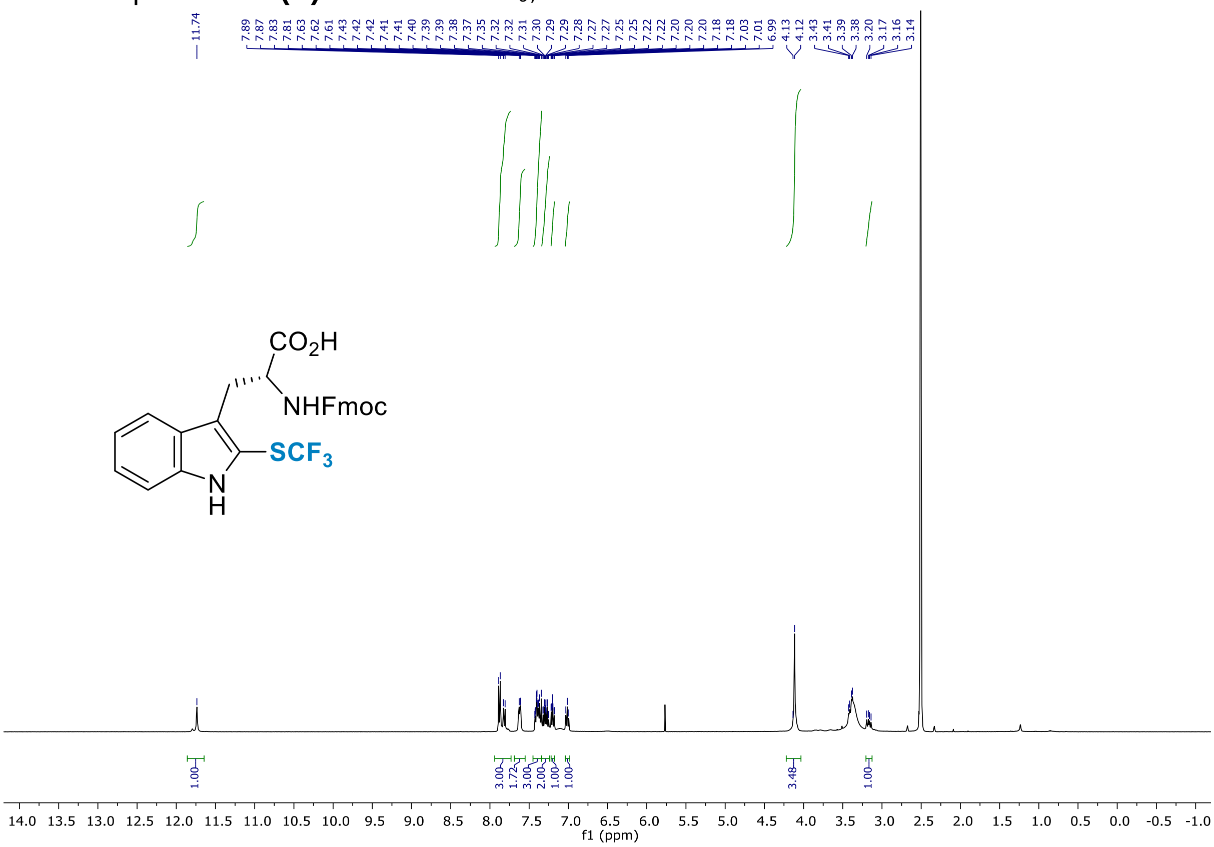

^{19}F NMR spectrum of **(*R*)-4b** in DMSO-d_6, 471 MHz (referenced to $CFCl_3$ at –0.24 ppm)

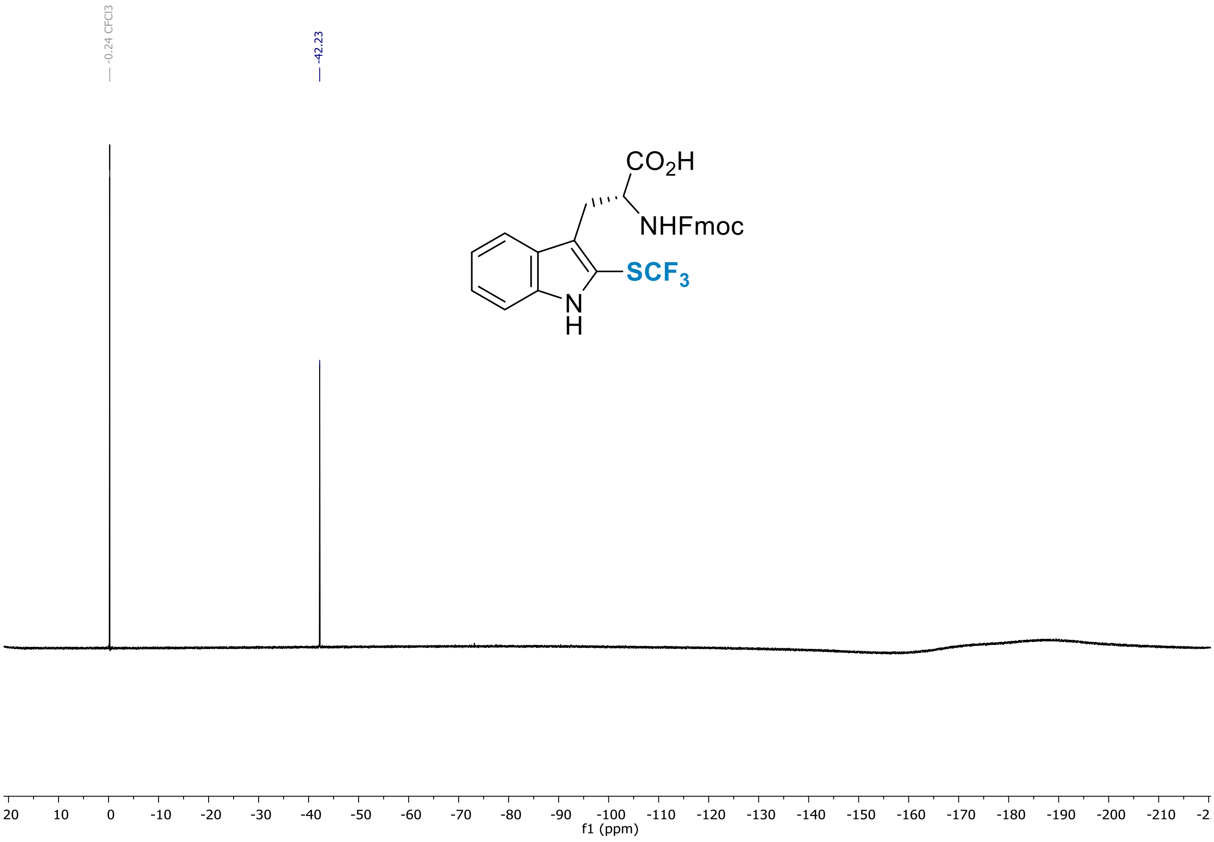

^{1}H NMR spectrum of **4c** in $CDCl_3$, 500 MHz

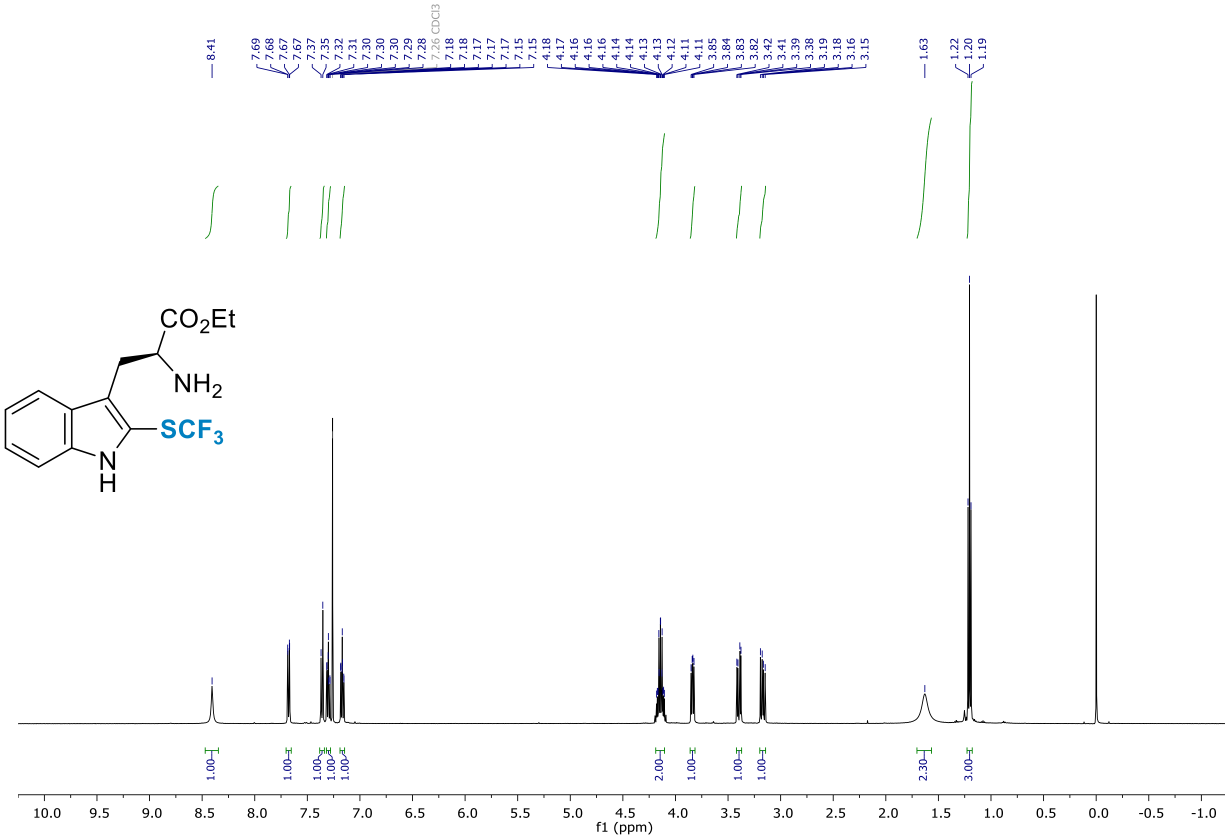

$^{13}C\{^{1}H\}$ NMR spectrum of **4c** in $CDCl_3$, 126 MHz

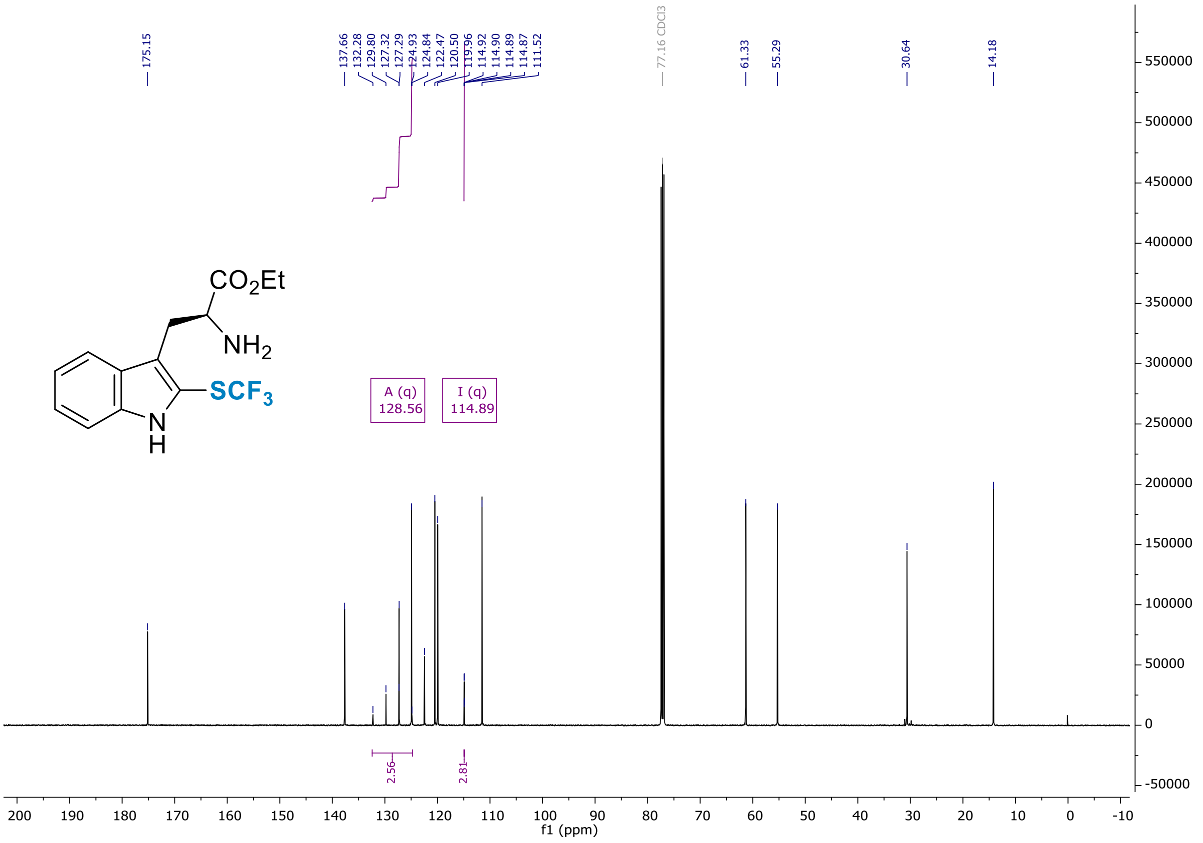

^{19}F NMR spectrum of **4c** in $CDCl_3$, 471 MHz (referenced to $CFCl_3$ at 0.65 ppm)

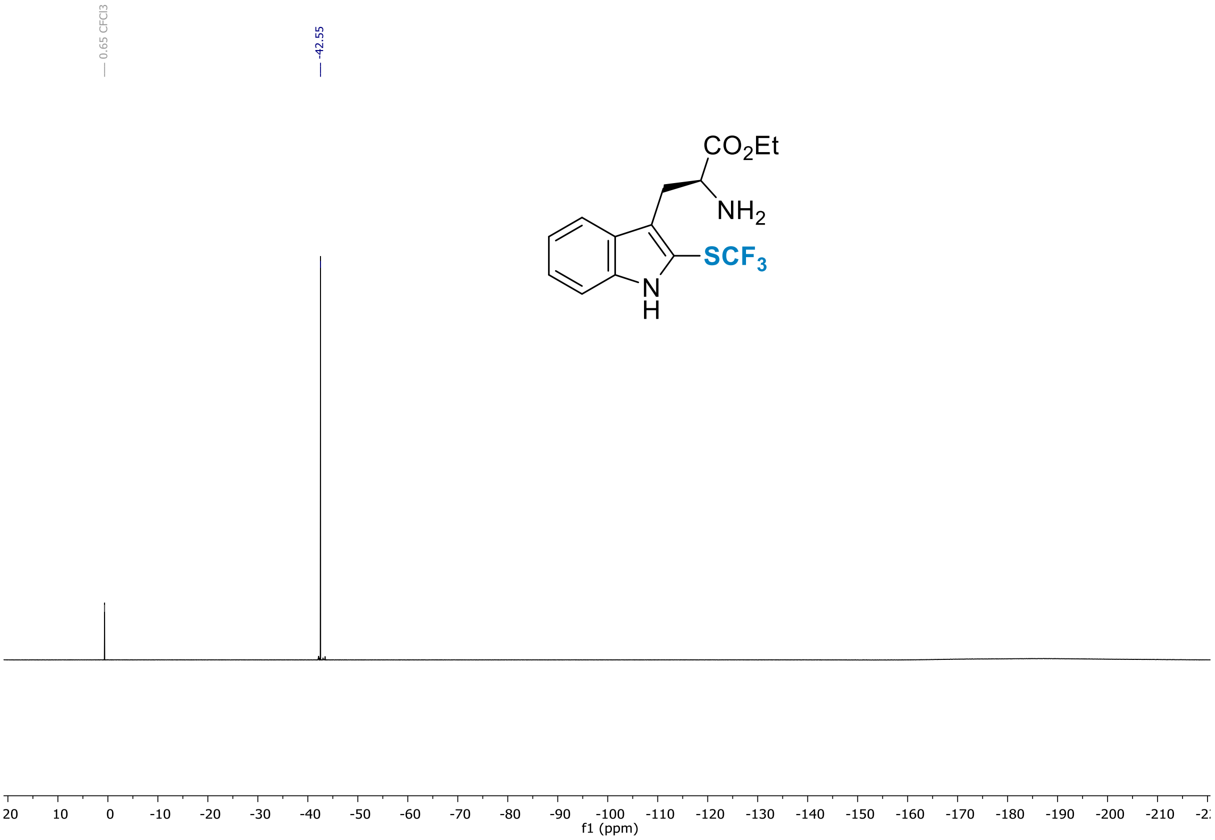

^{1}H NMR spectrum of **4d** in $CDCl_3$, 500 MHz

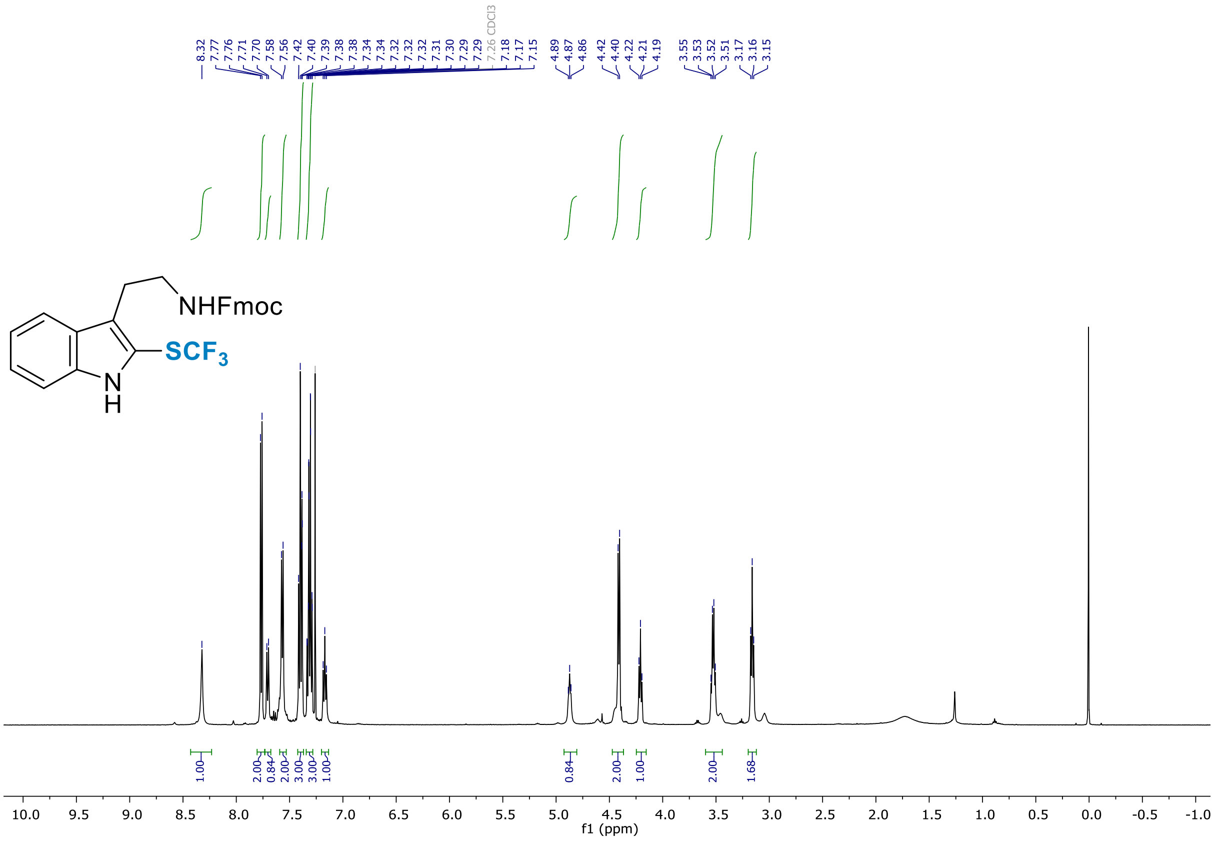

^{13}C{^{1}H} NMR spectrum of **4d** in $CDCl_3$, 126 MHz

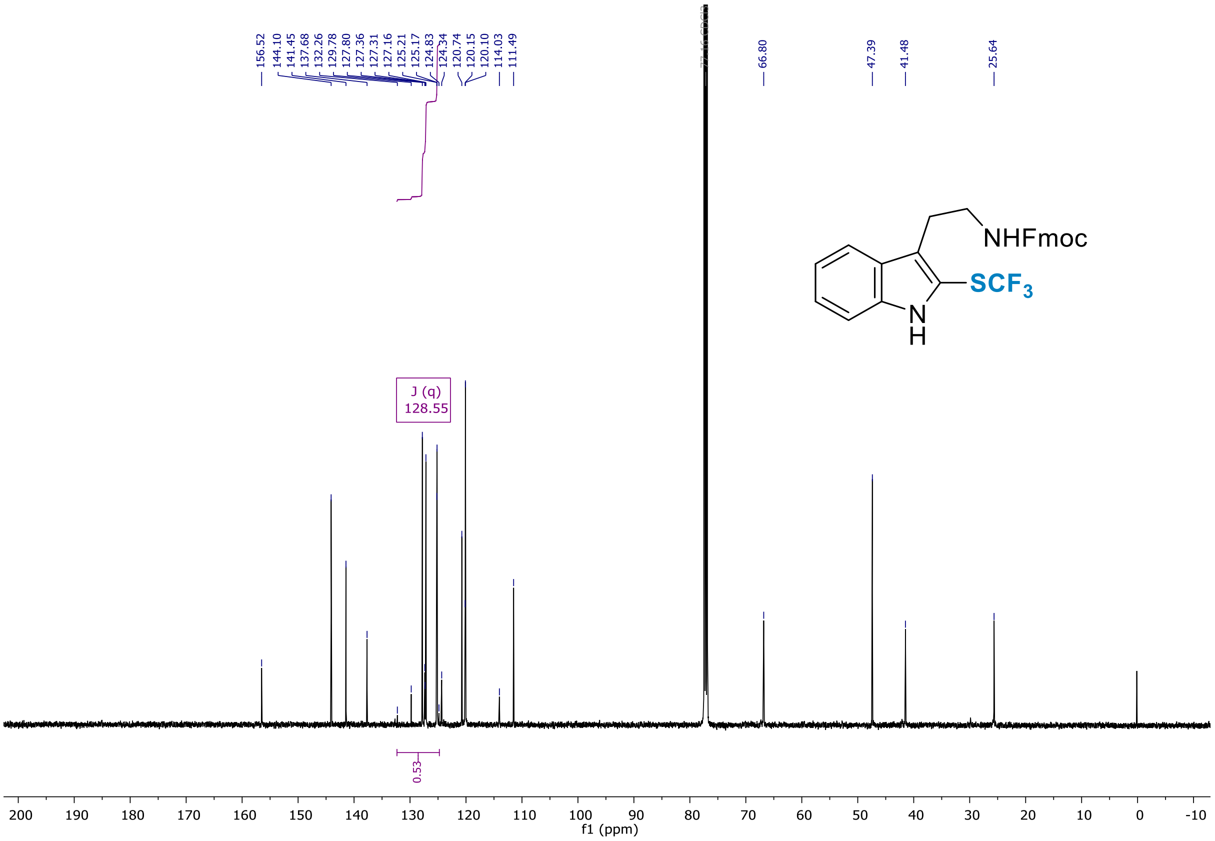

^{19}F NMR spectrum of **4d** in $CDCl_3$, 471 MHz (referenced to $CFCl_3$ at 0.65 ppm)

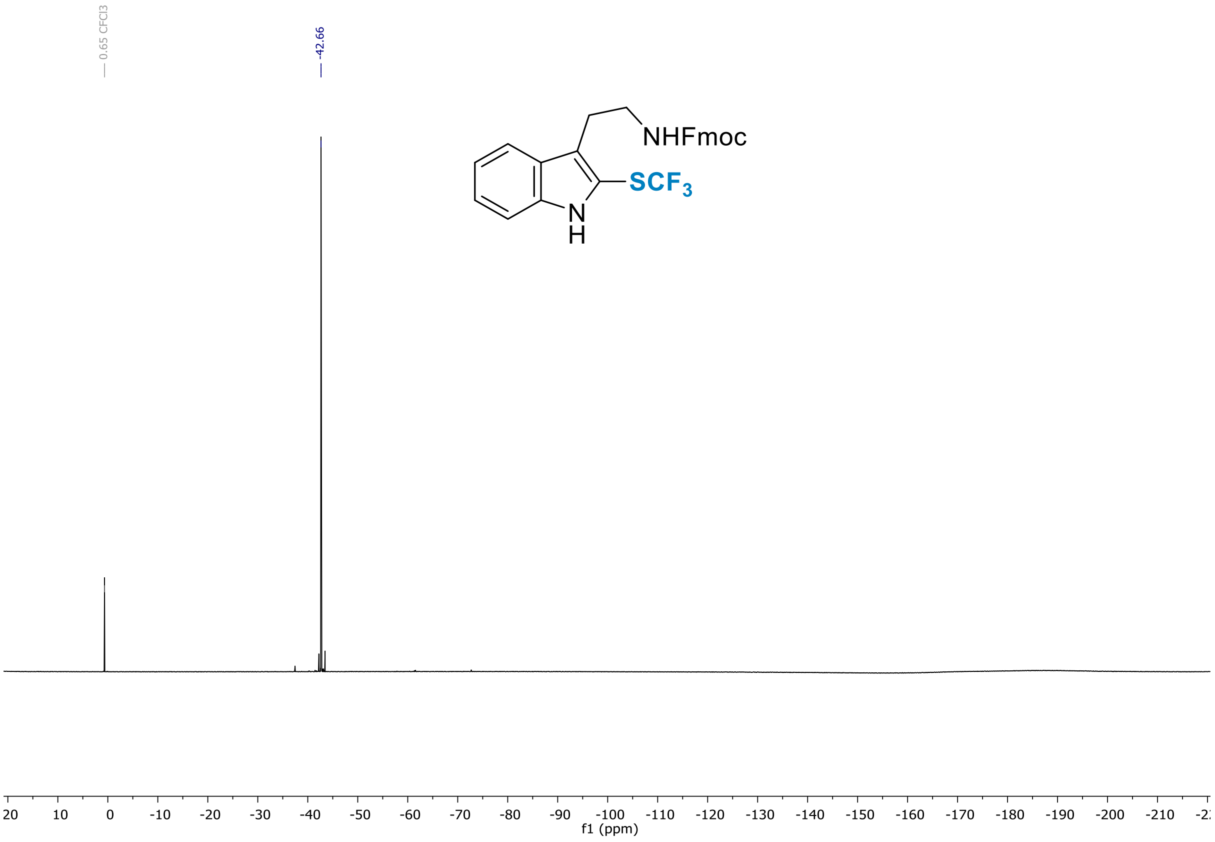

^{1}H NMR spectrum of **4e** in $CDCl_3$, 500 MHz

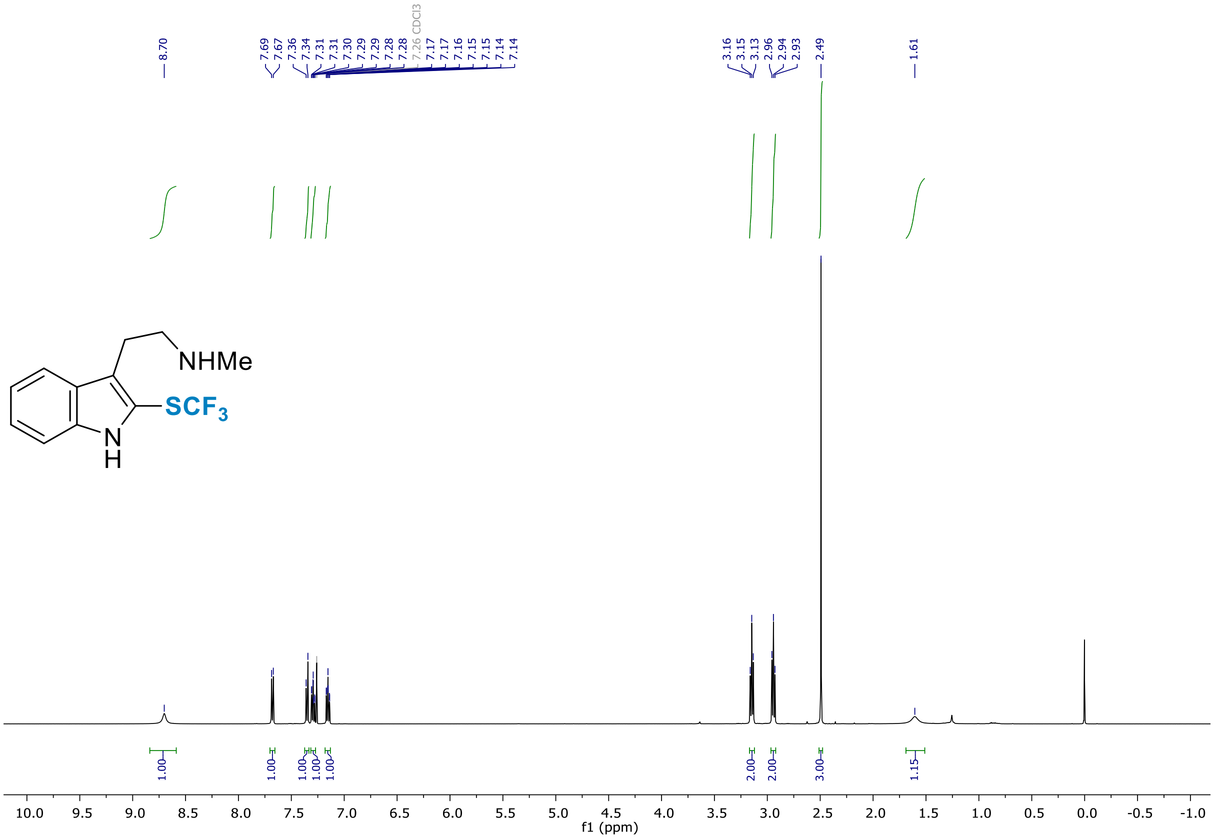

$^{13}C\{^{1}H\}$ NMR spectrum of **4e** in $CDCl_3$, 126 MHz

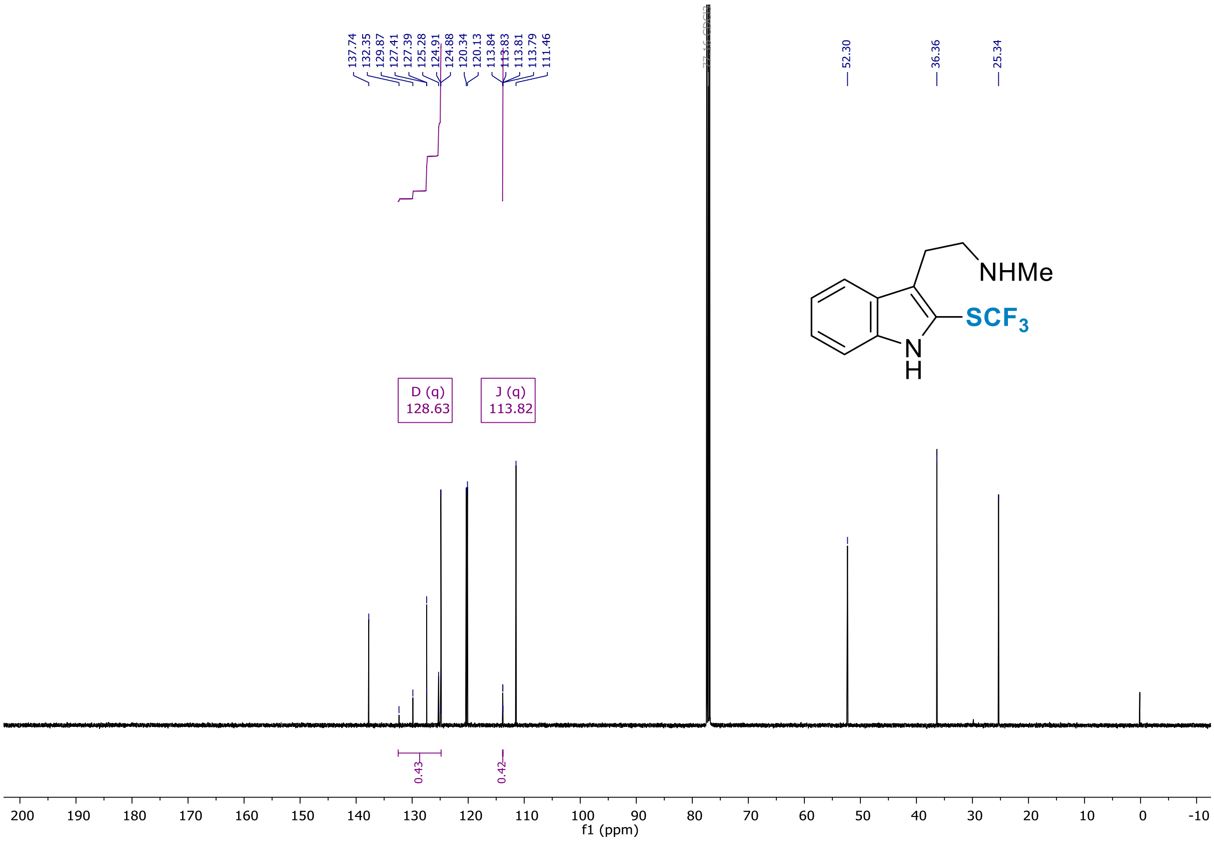

^{19}F NMR spectrum of **4e** in $CDCl_3$, 471 MHz (referenced to $CFCl_3$ at 0.65 ppm)

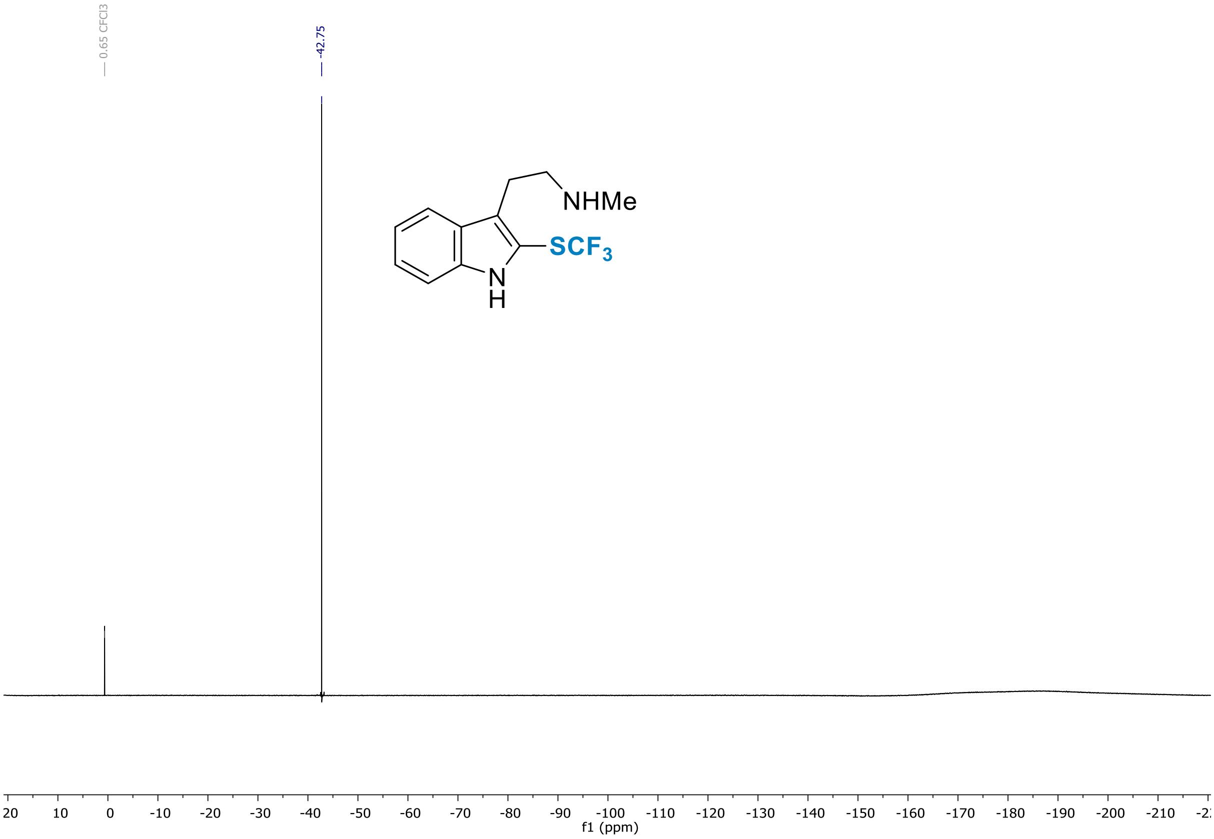

^{1}H NMR spectrum of **4f** in $CDCl_3$, 500 MHz

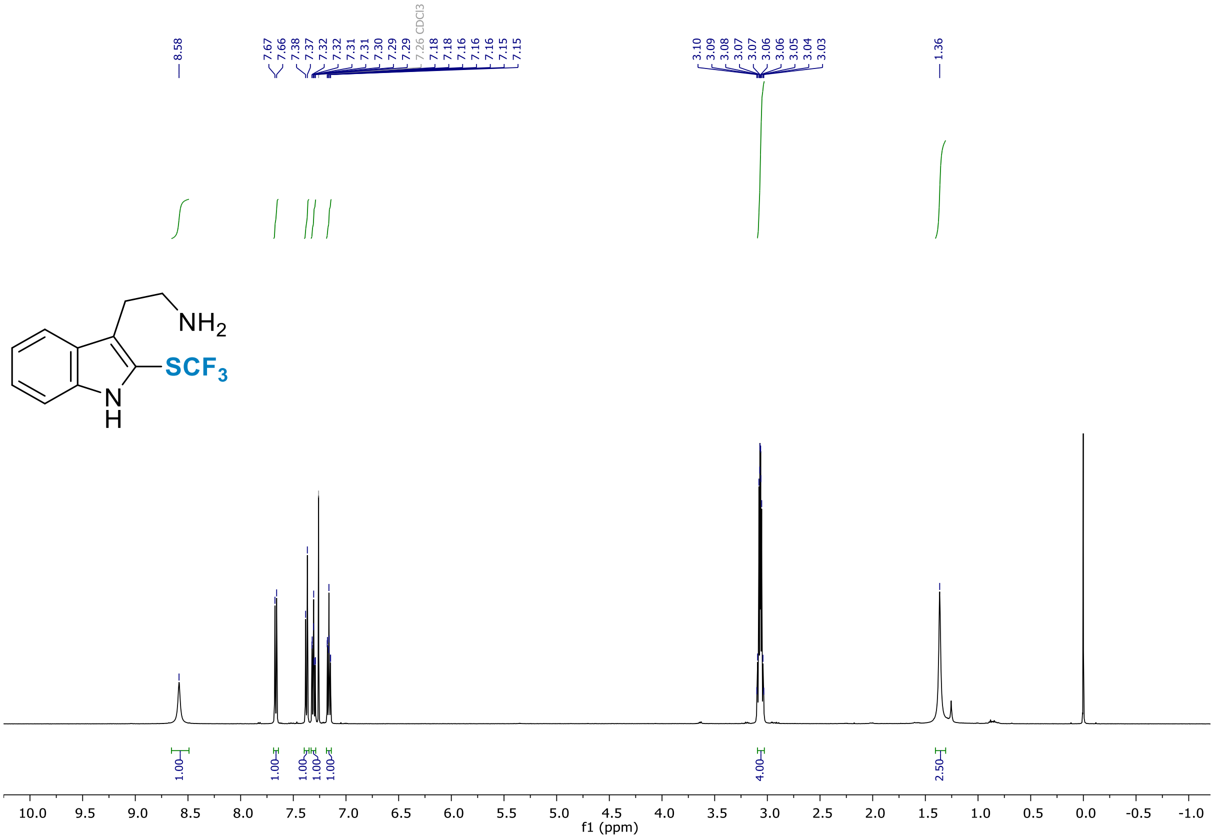

$^{13}C\{^{1}H\}$ NMR spectrum of **4f** in $CDCl_3$, 126 MHz

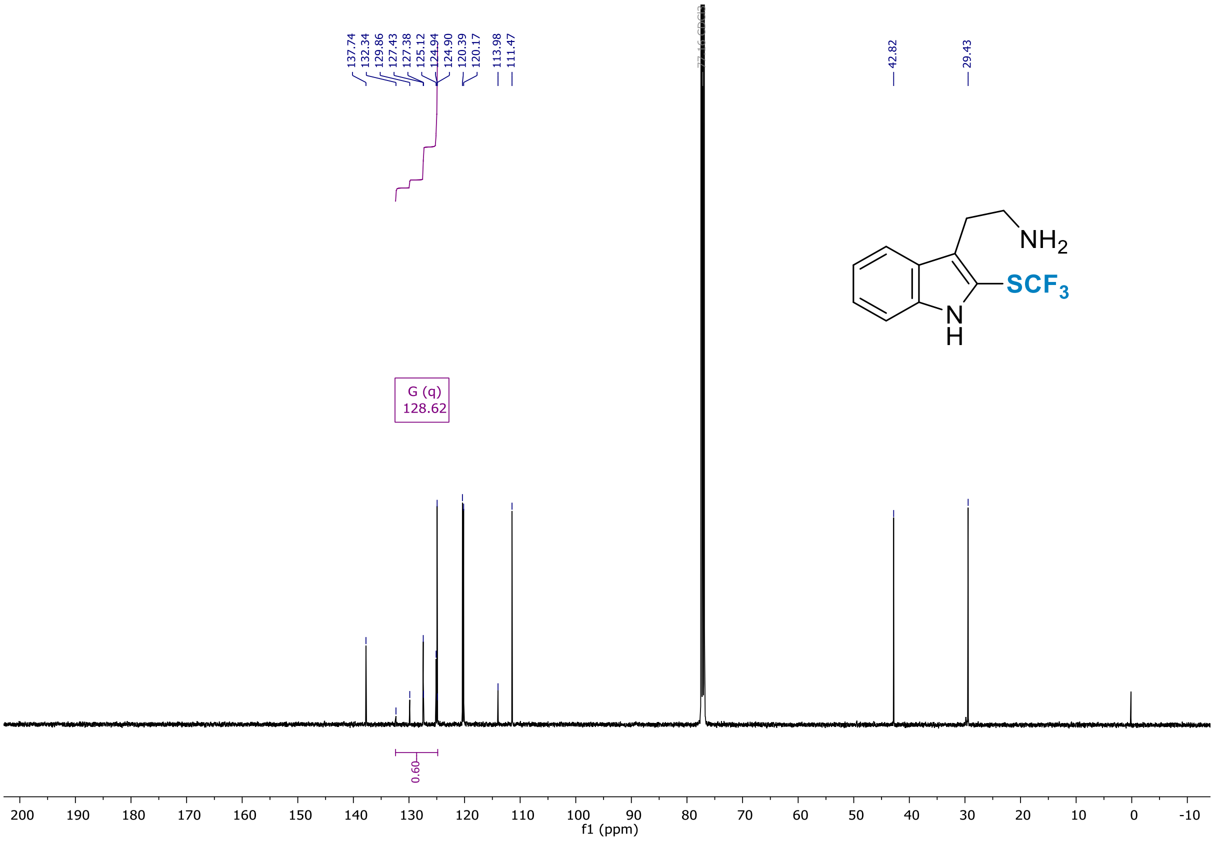

^{19}F NMR spectrum of **4f** in $CDCl_3$, 471 MHz (referenced to $CFCl_3$ at 0.65 ppm)

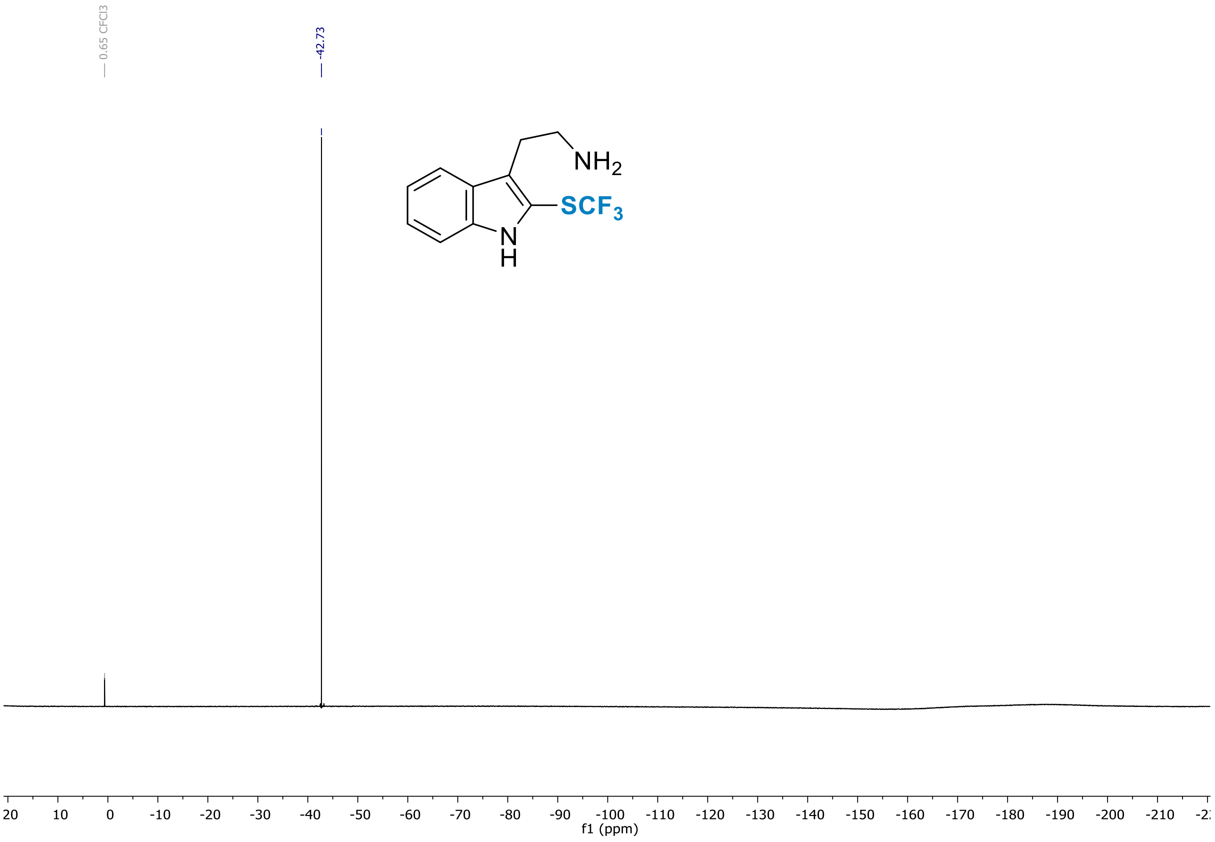

^{1}H NMR spectrum of **4h** in $CDCl_3$, 400 MHz

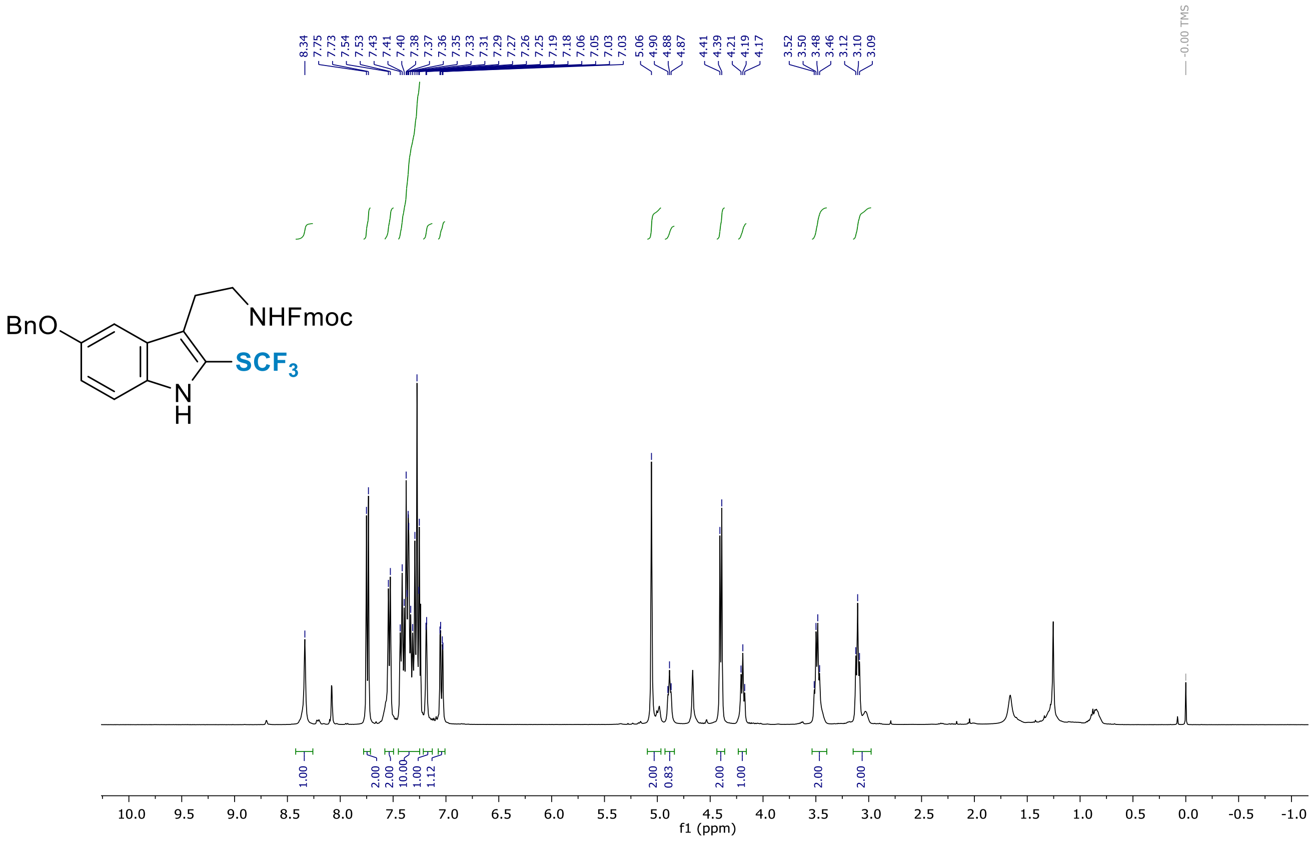

$^{13}C\{^{1}H\}$ NMR spectrum of **4h** in $CDCl_3$, 101 MHz

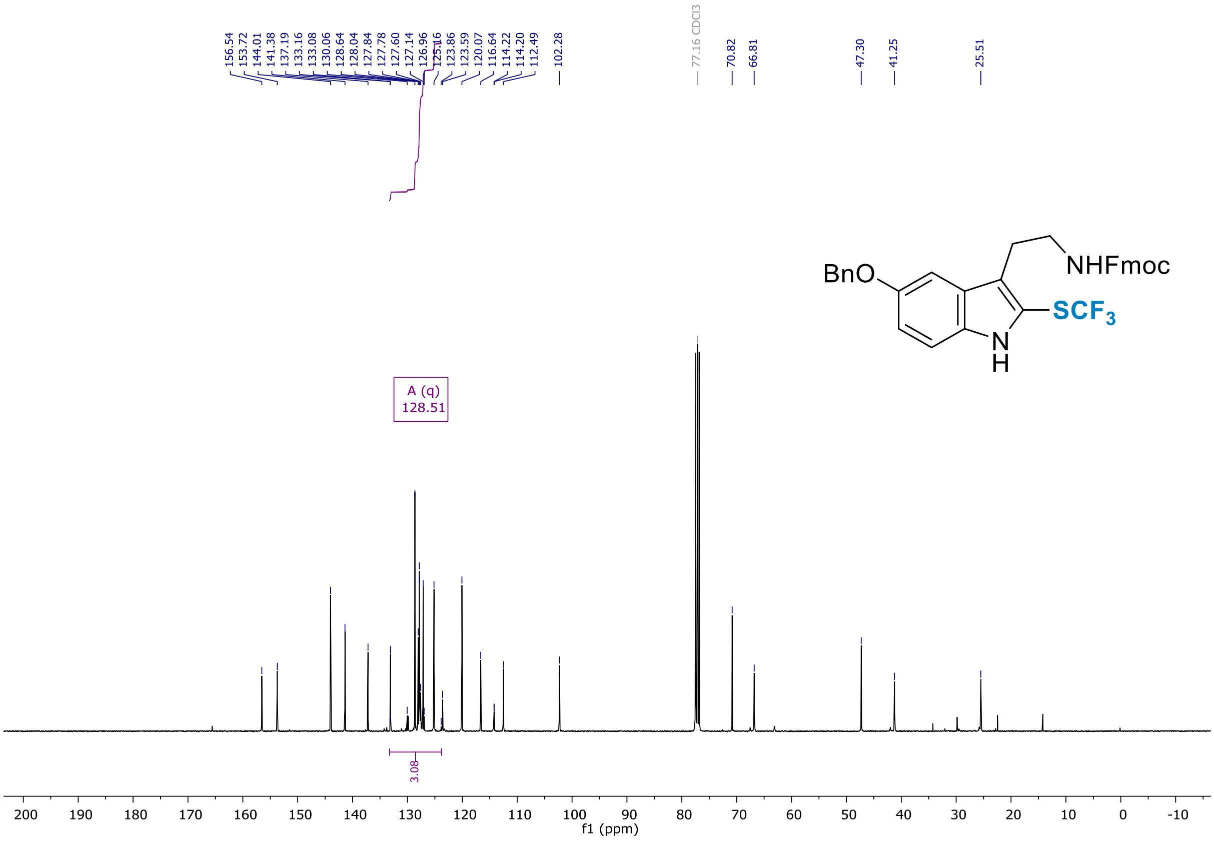

^{19}F NMR spectrum of **4h** in $CDCl_3$, 376 MHz (referenced to C_6F_6 at –161.64 ppm)

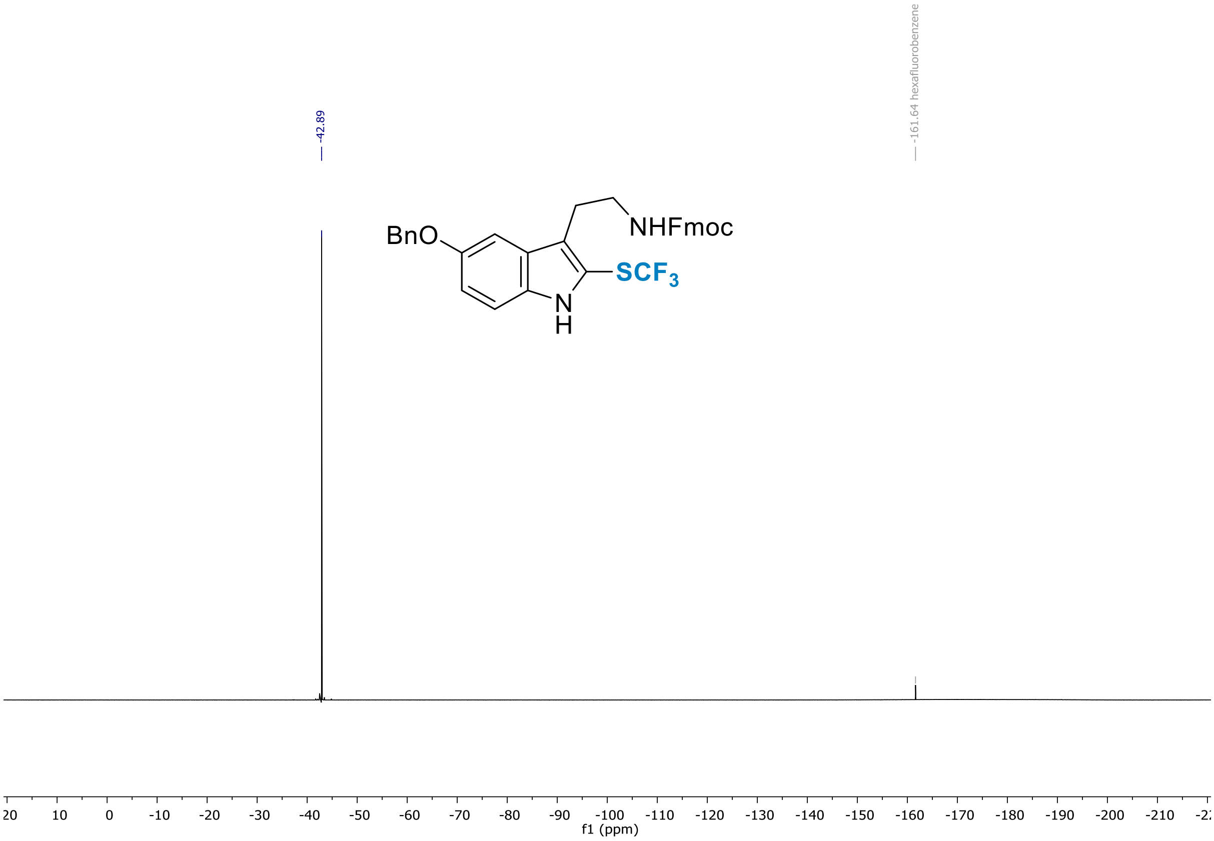

^{1}H NMR spectrum of **4i** in $CDCl_3$, 400 MHz

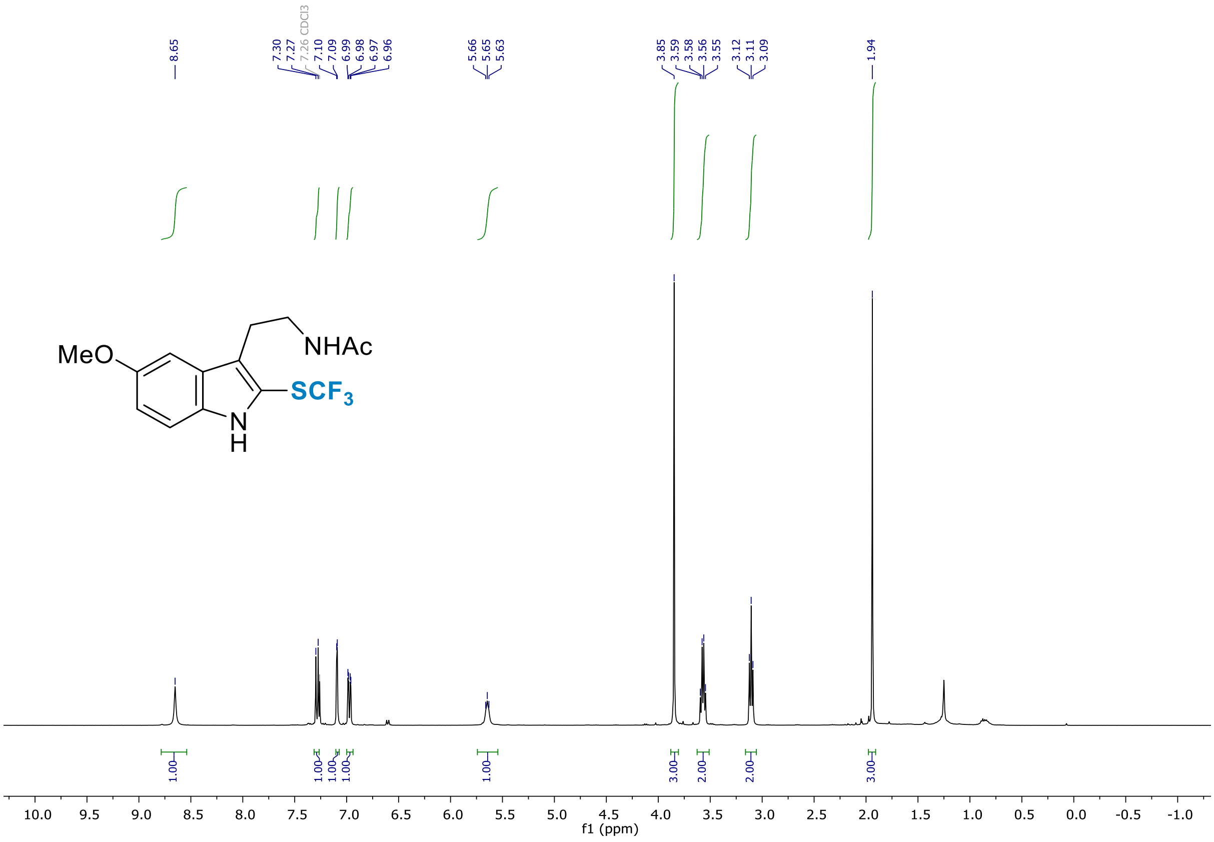

$^{13}C\{^{1}H\}$ NMR spectrum of **4i** in $CDCl_3$, 101 MHz

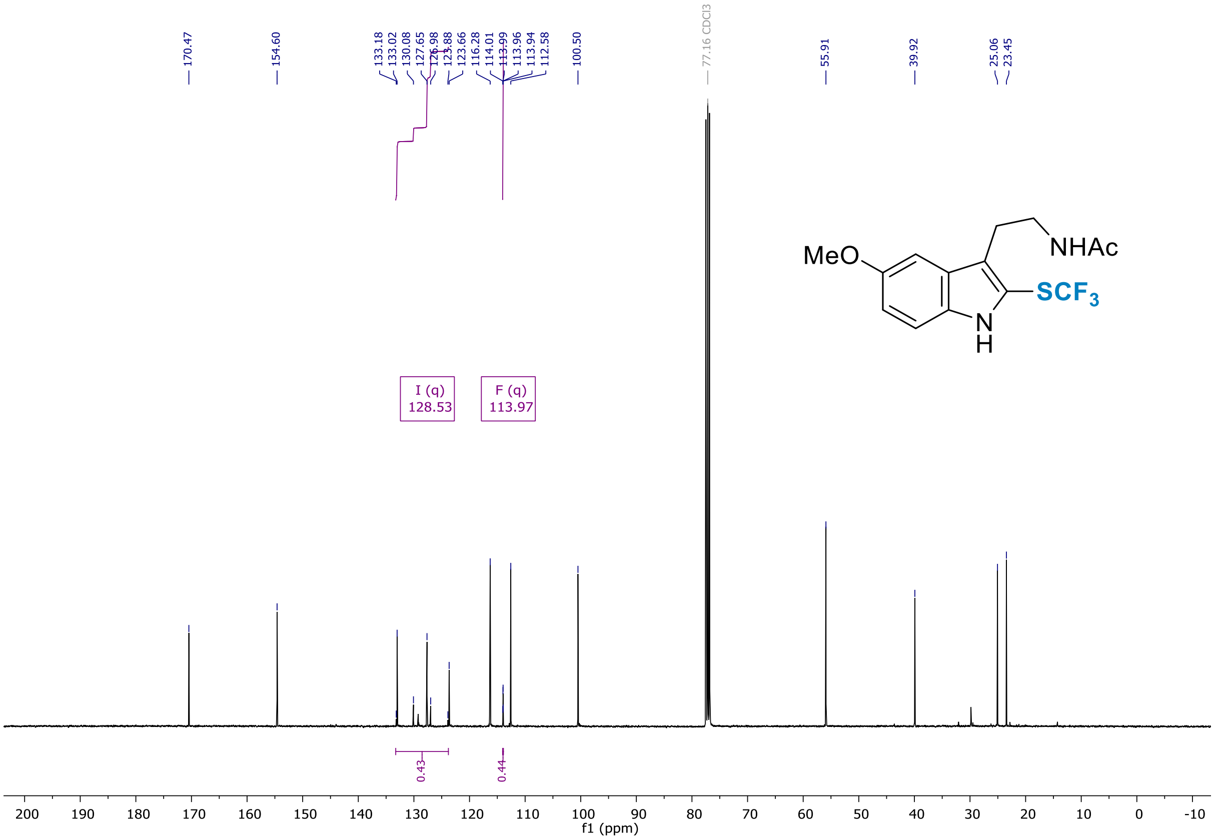

^{19}F NMR spectrum of **4i** in $CDCl_3$, 376 MHz (referenced to C_6F_6 at –161.64 ppm)

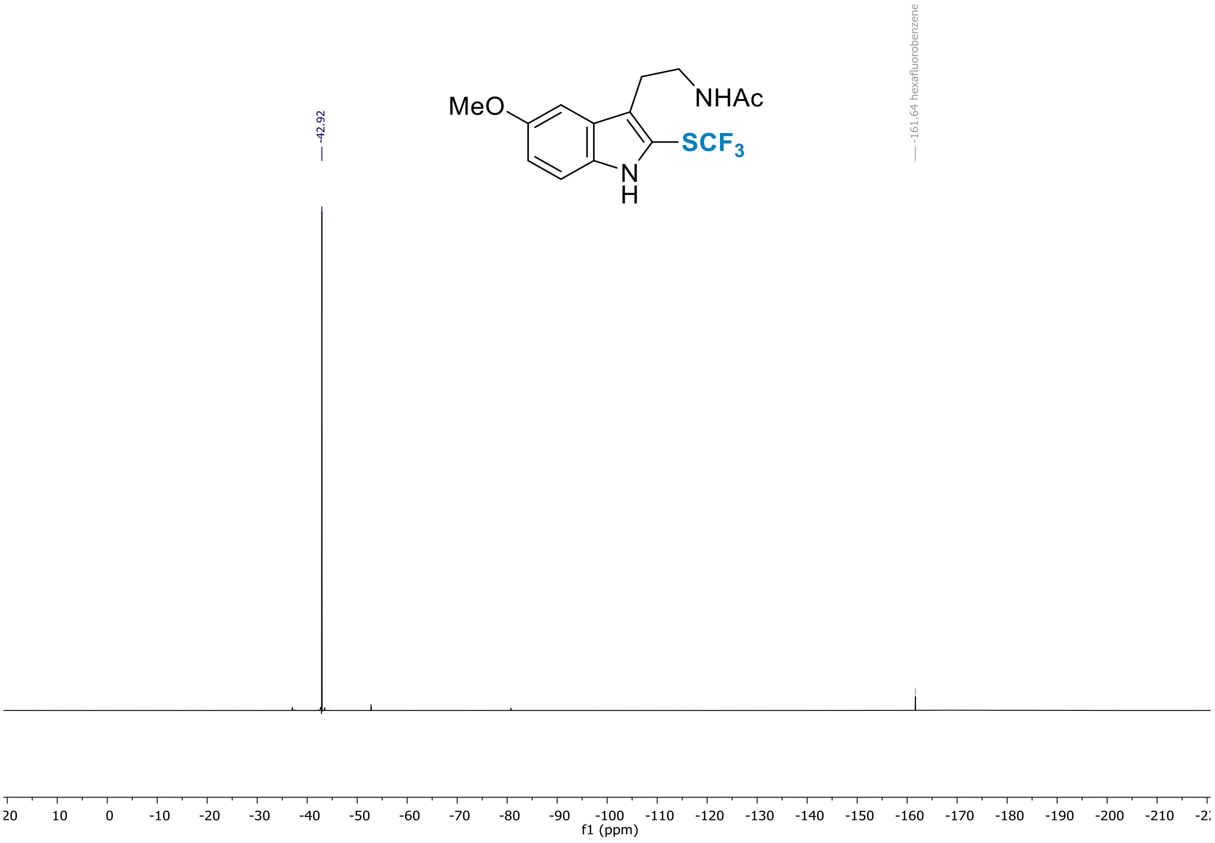

^{1}H NMR spectrum of **6a** in DMSO-d_6, 500 MHz

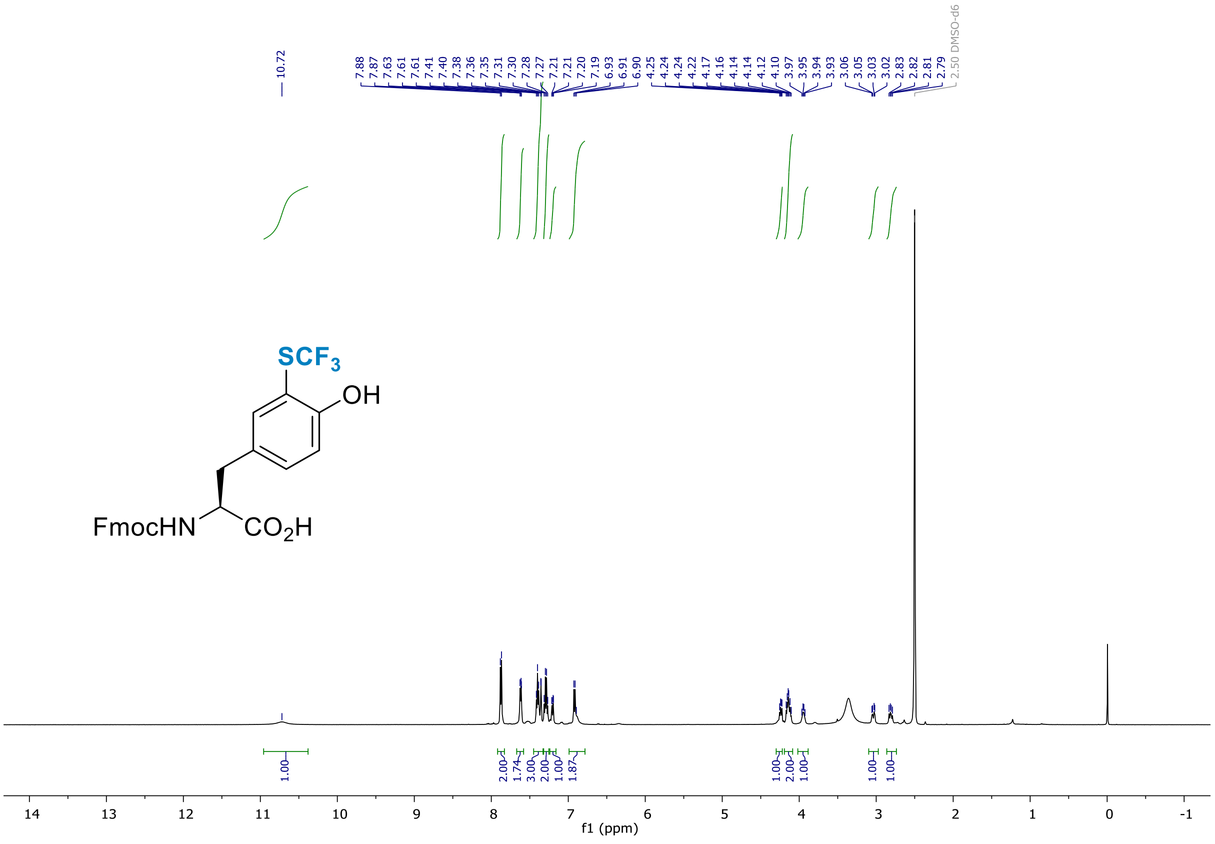

$^{13}C\{^{1}H\}$ NMR spectrum of **(*S*)-6a** in DMSO-*d*$_6$, 126 MHz

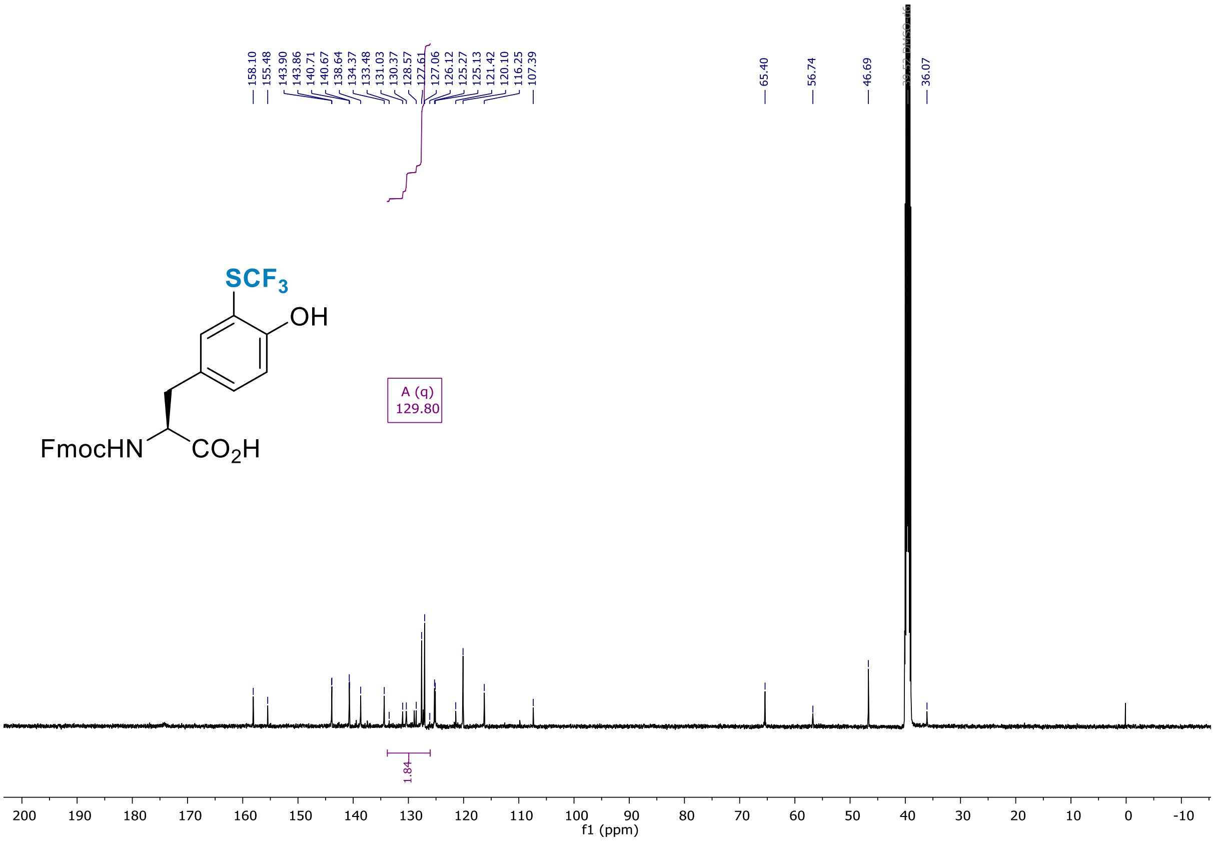

^{19}F NMR spectrum of **(*S*)-6a** in DMSO-*d*$_6$, 471 MHz (referenced to $CFCl_3$ at –0.24 ppm)

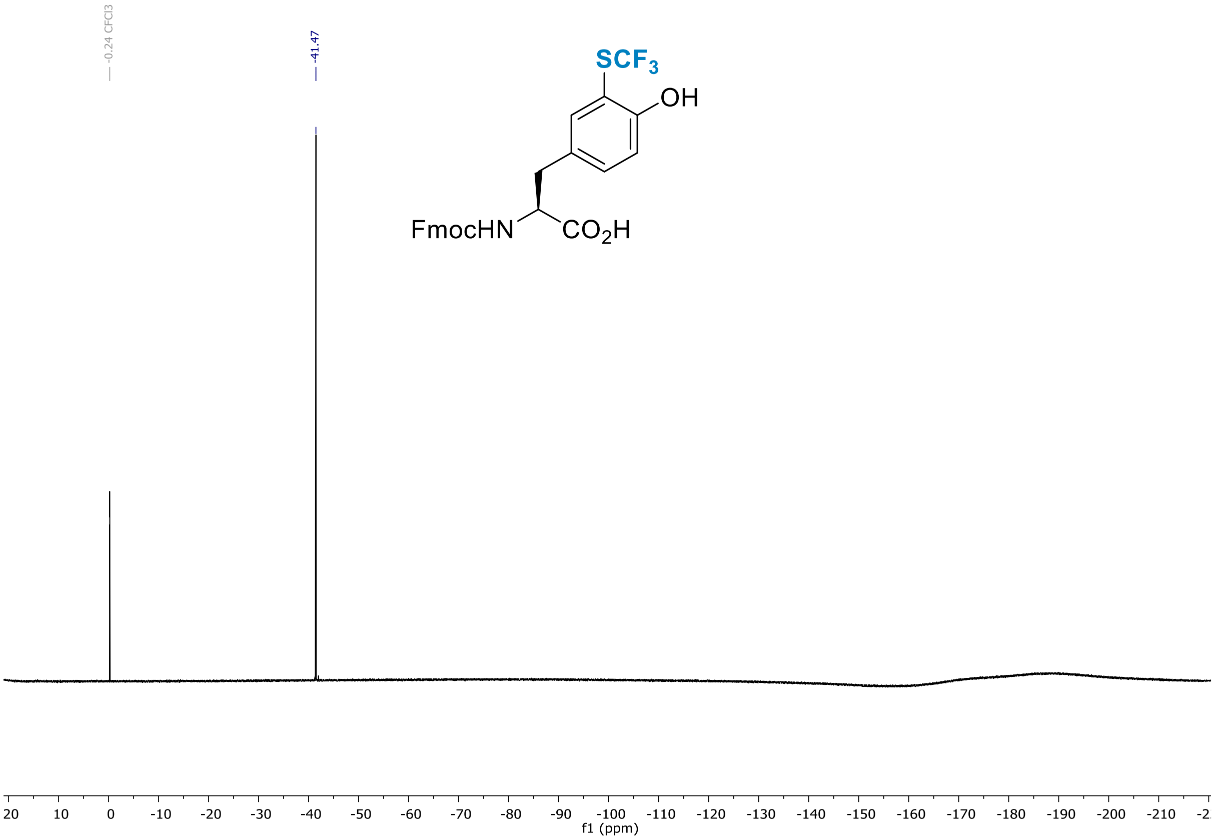

^{1}H NMR spectrum of ***(R)*-6a** in MeOD-d_4, 400 MHz

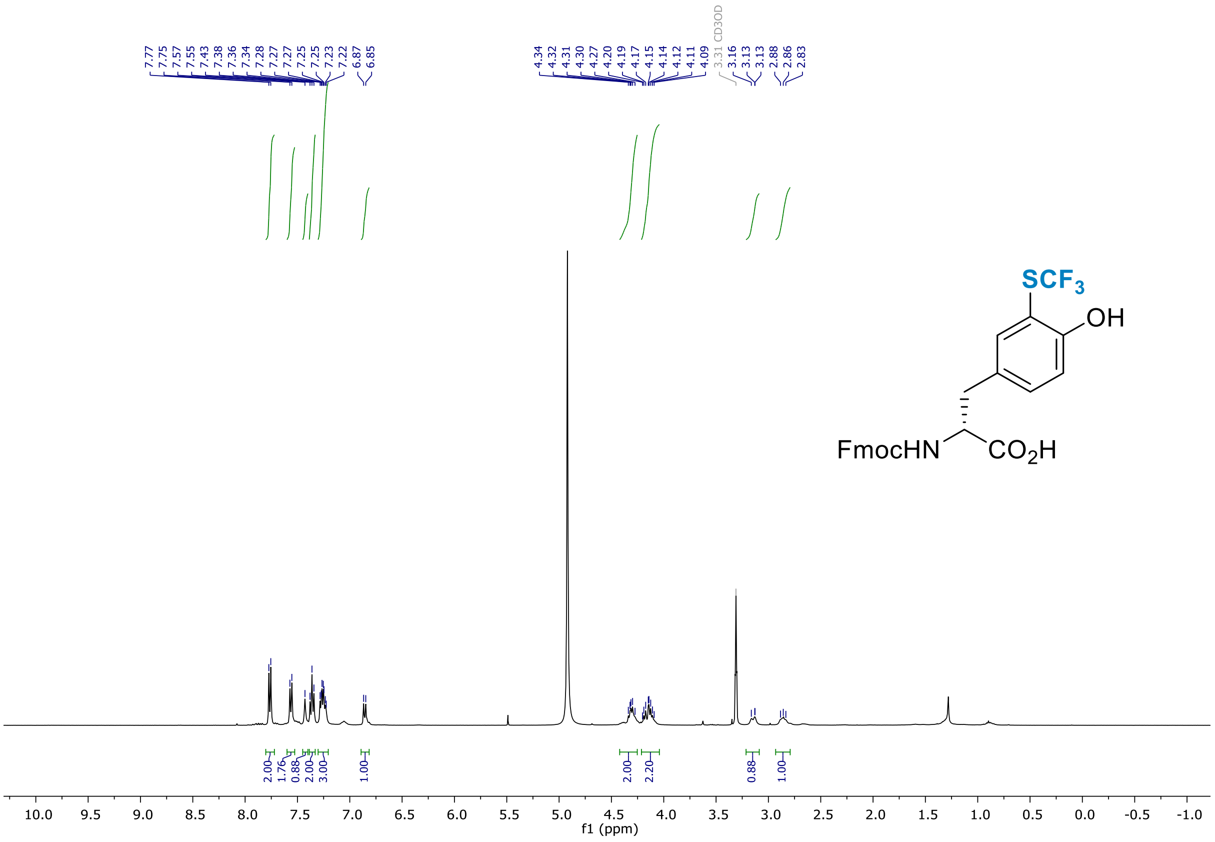

^{19}F NMR spectrum of ***(R)*-6a** in MeOD-d_4, 376 MHz (referenced to C_6F_6 at –165.37 ppm)

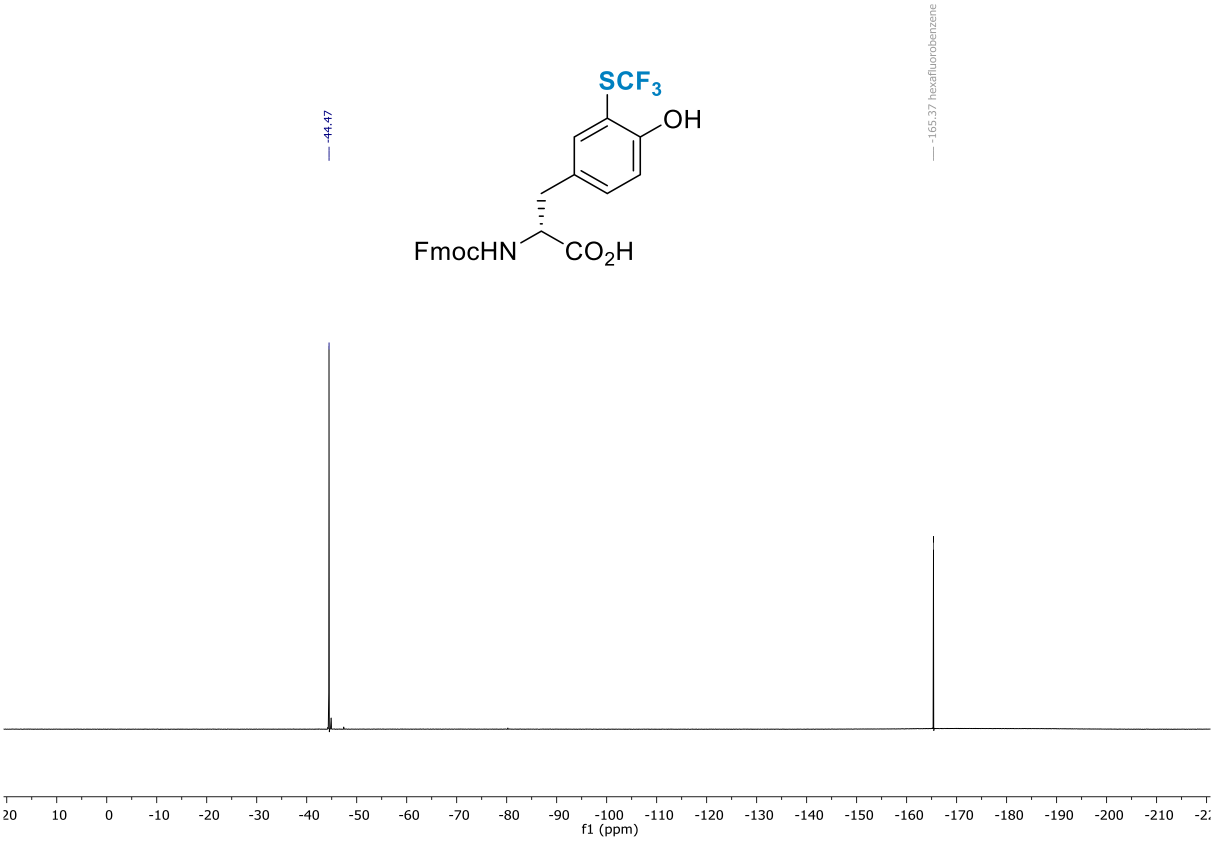

^{1}H NMR spectrum of **6b** in $CDCl_3$, 500 MHz

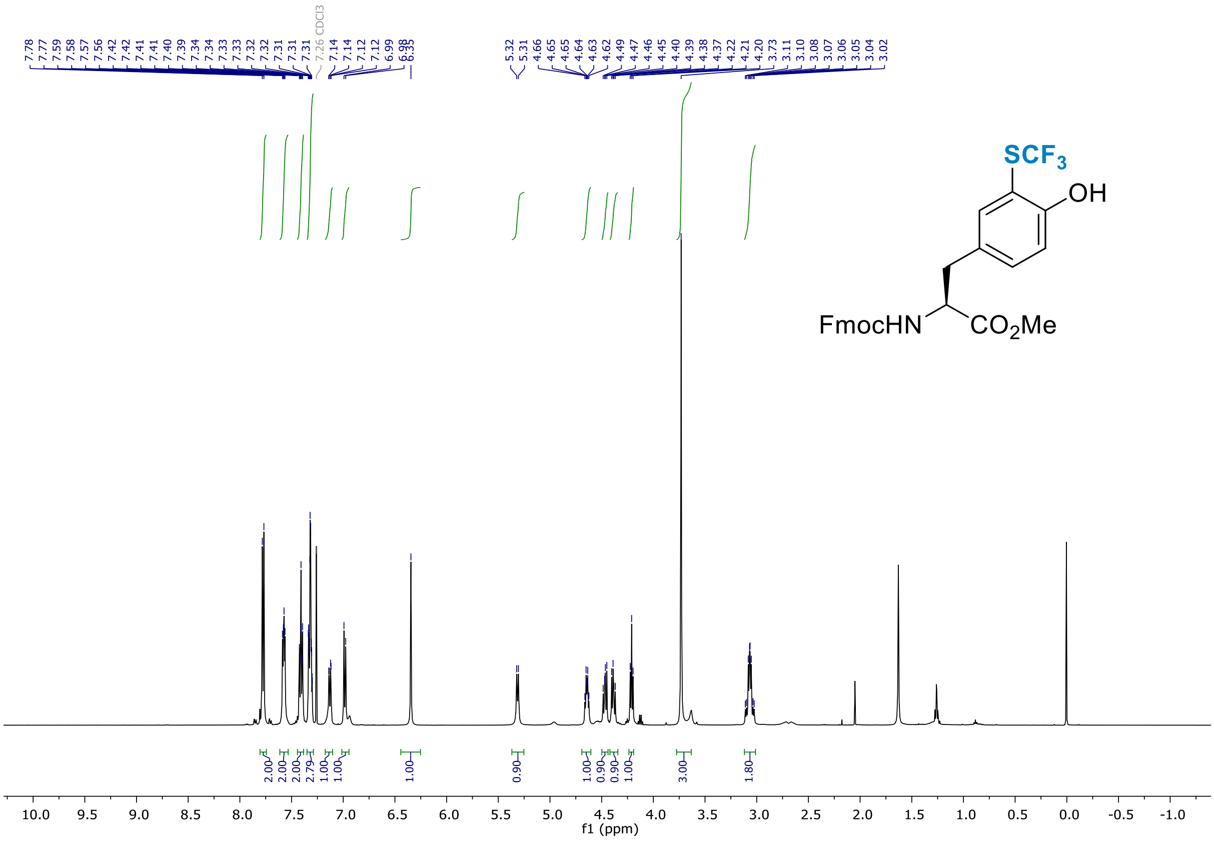

$^{13}C\{^1H\}$ NMR spectrum of **6b** in $CDCl_3$, 126 MHz

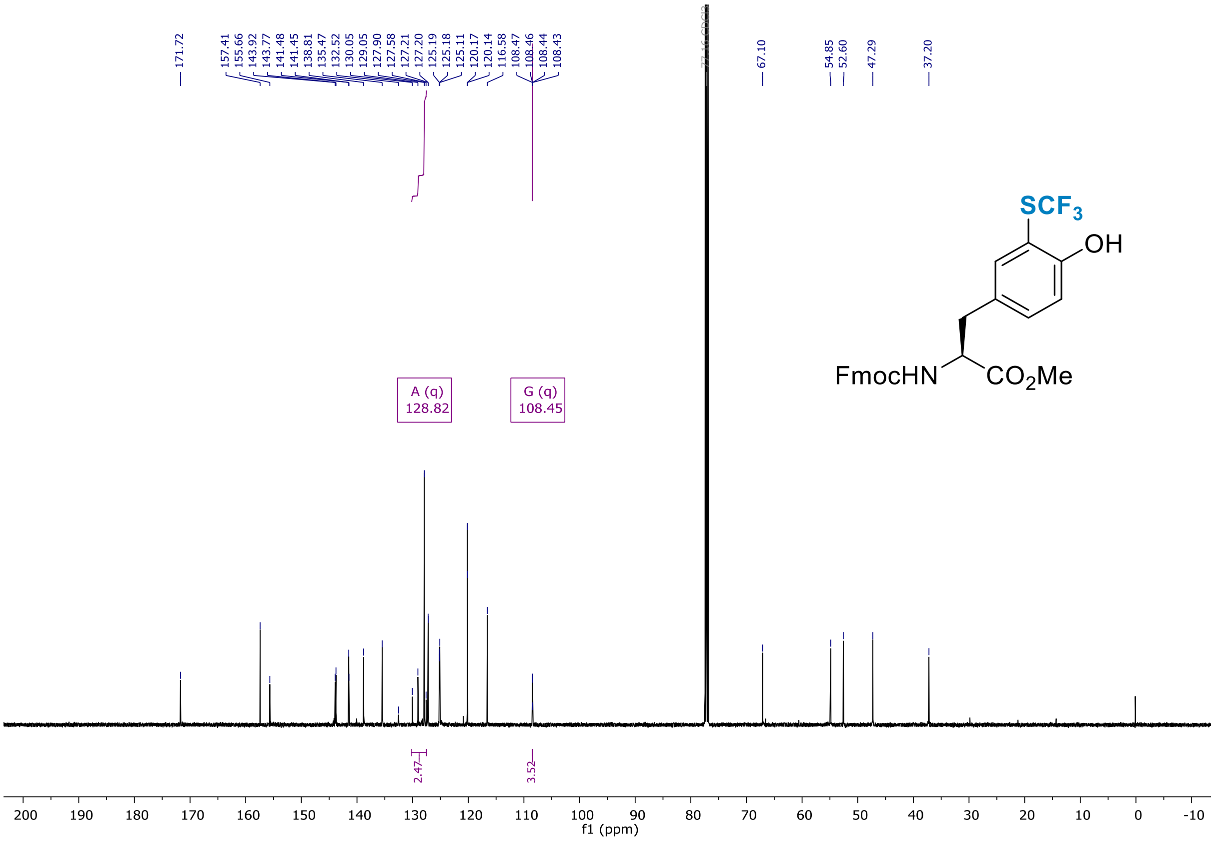

^{19}F NMR spectrum of **6b** in $CDCl_3$, 471 MHz (referenced to $CFCl_3$ at 0.65 ppm)

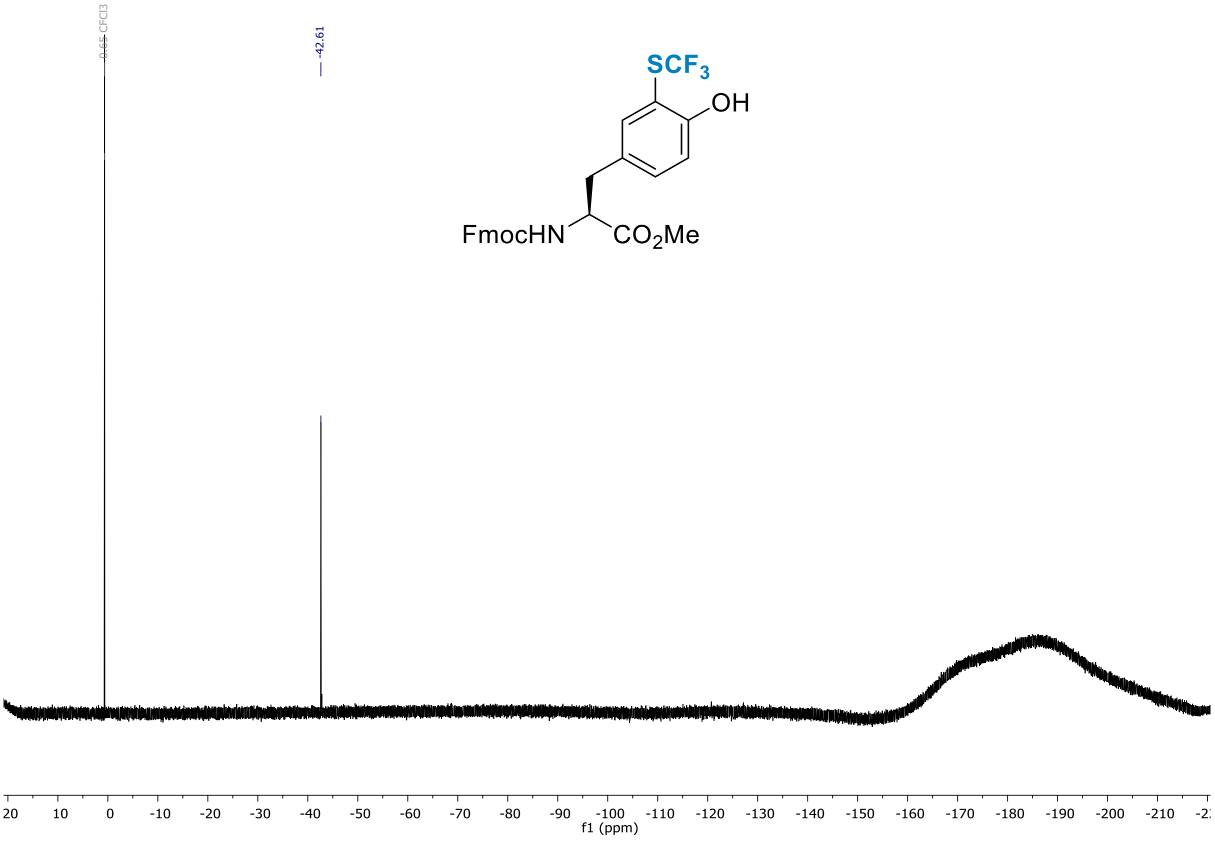

^{1}H NMR spectrum of **6d** in $CDCl_3$, 500 MHz

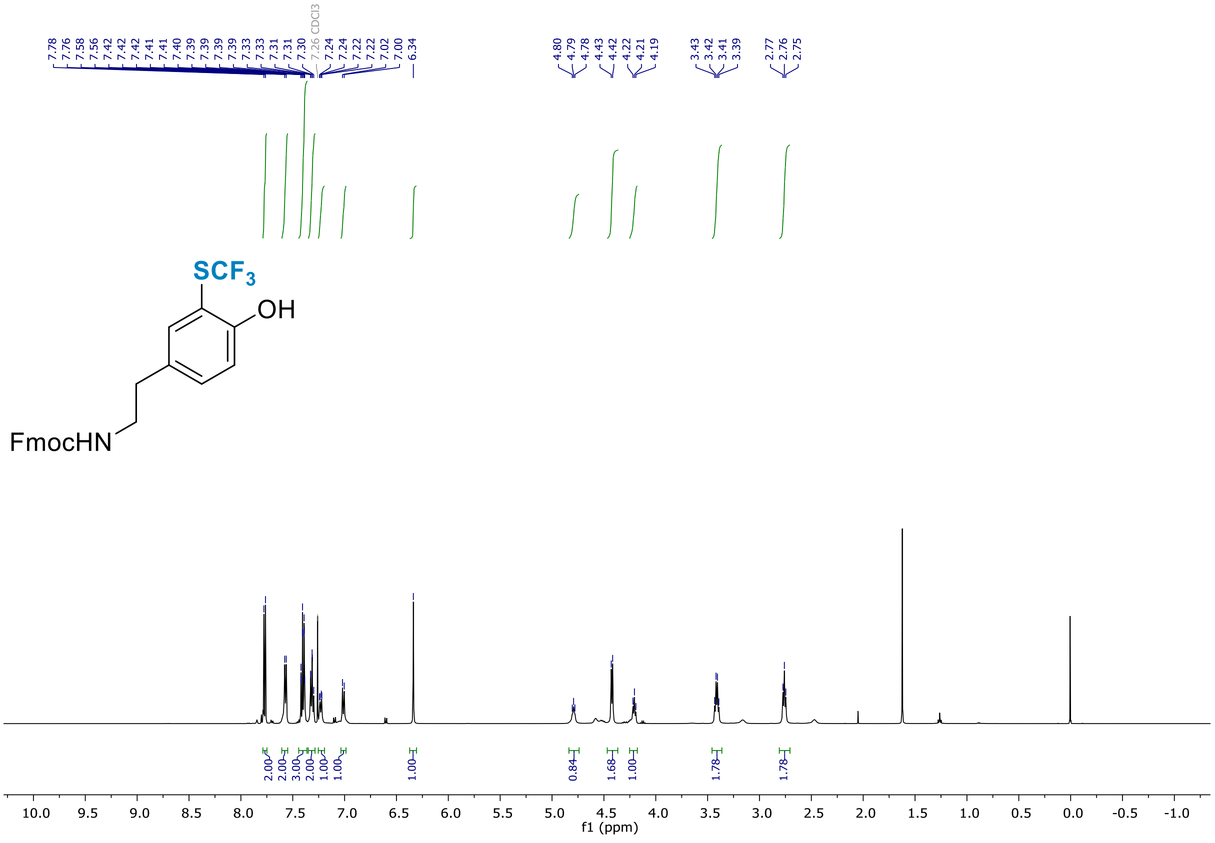

$^{13}C\{^1H\}$ NMR spectrum of **6d** in $CDCl_3$, 126 MHz

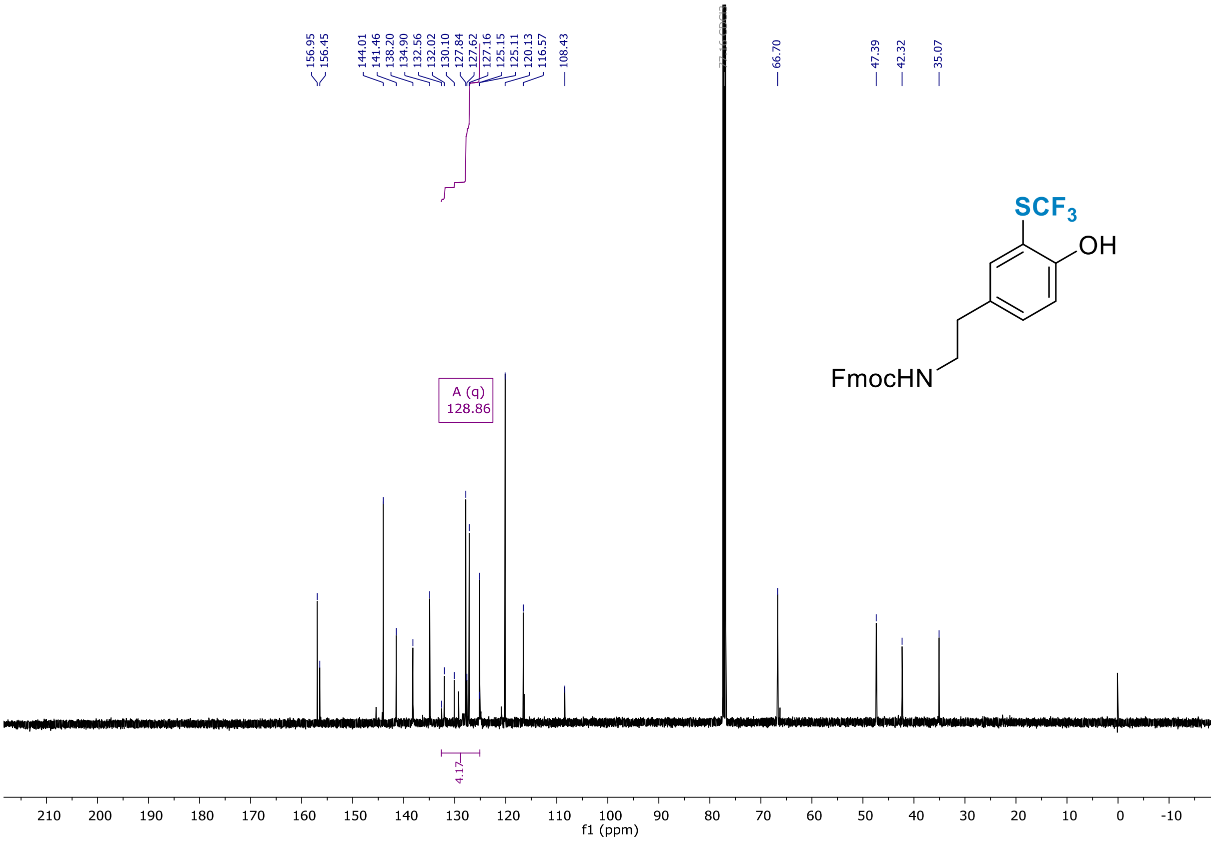

^{19}F NMR spectrum of **6d** in $CDCl_3$, 376 MHz (referenced to C_6F_6 at –161.64 ppm)

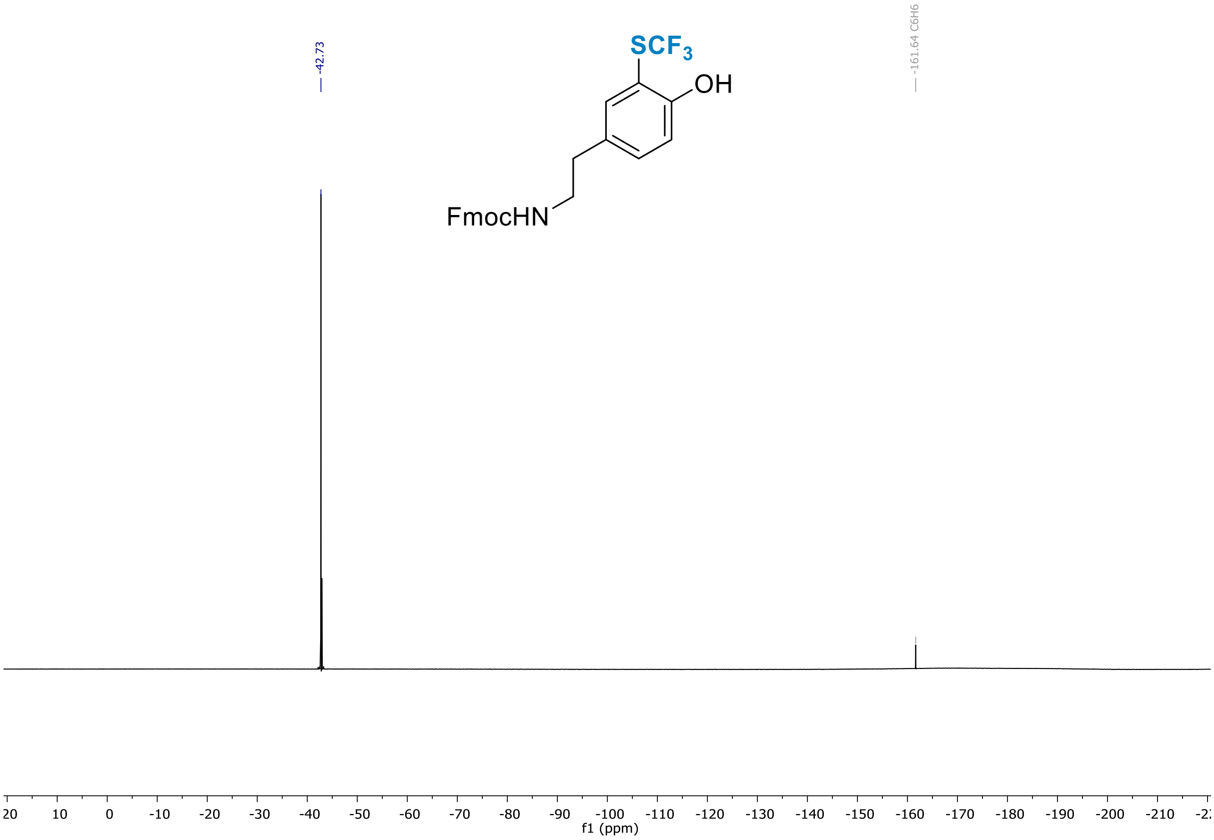

^{1}H NMR spectrum of **6e** in MeOD-d_4, 400 MHz

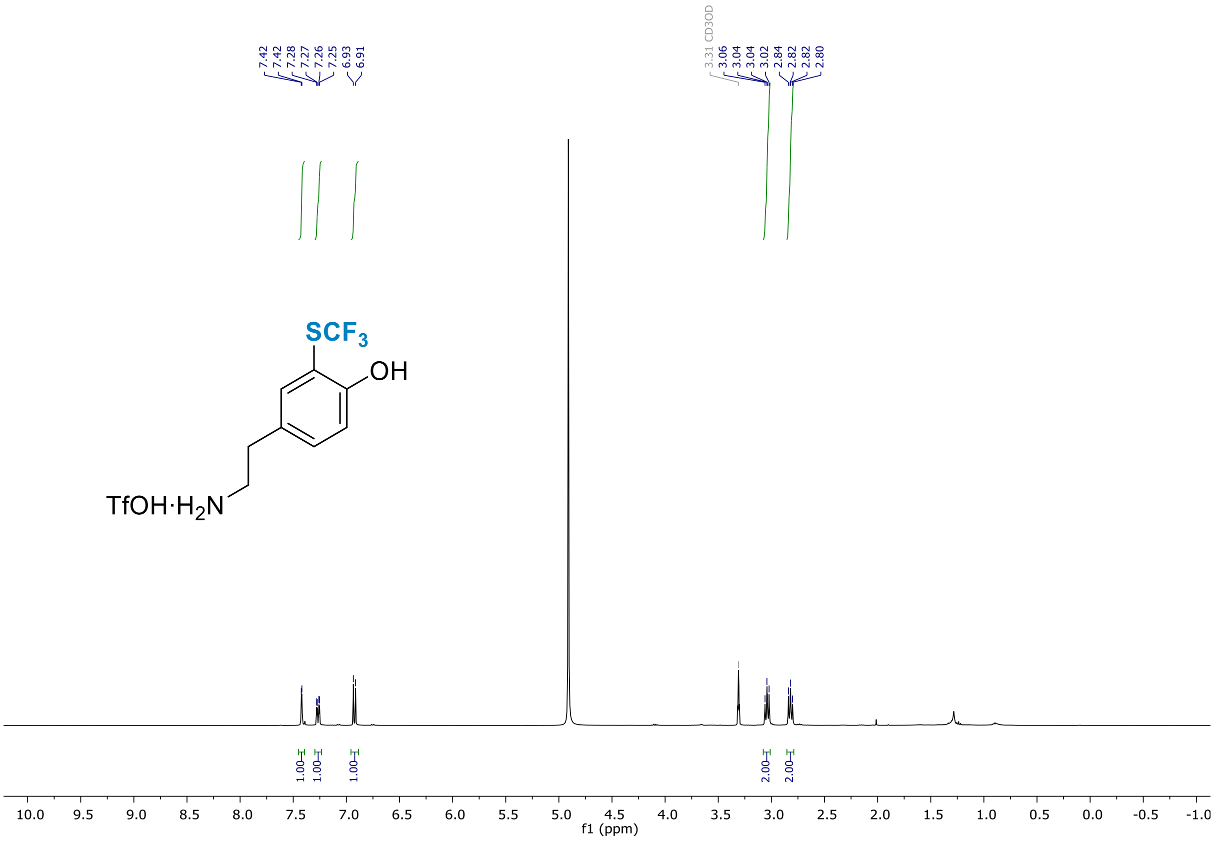

^{13}C{^{1}H} NMR spectrum of **6e** in MeOD-d_4, 101 MHz

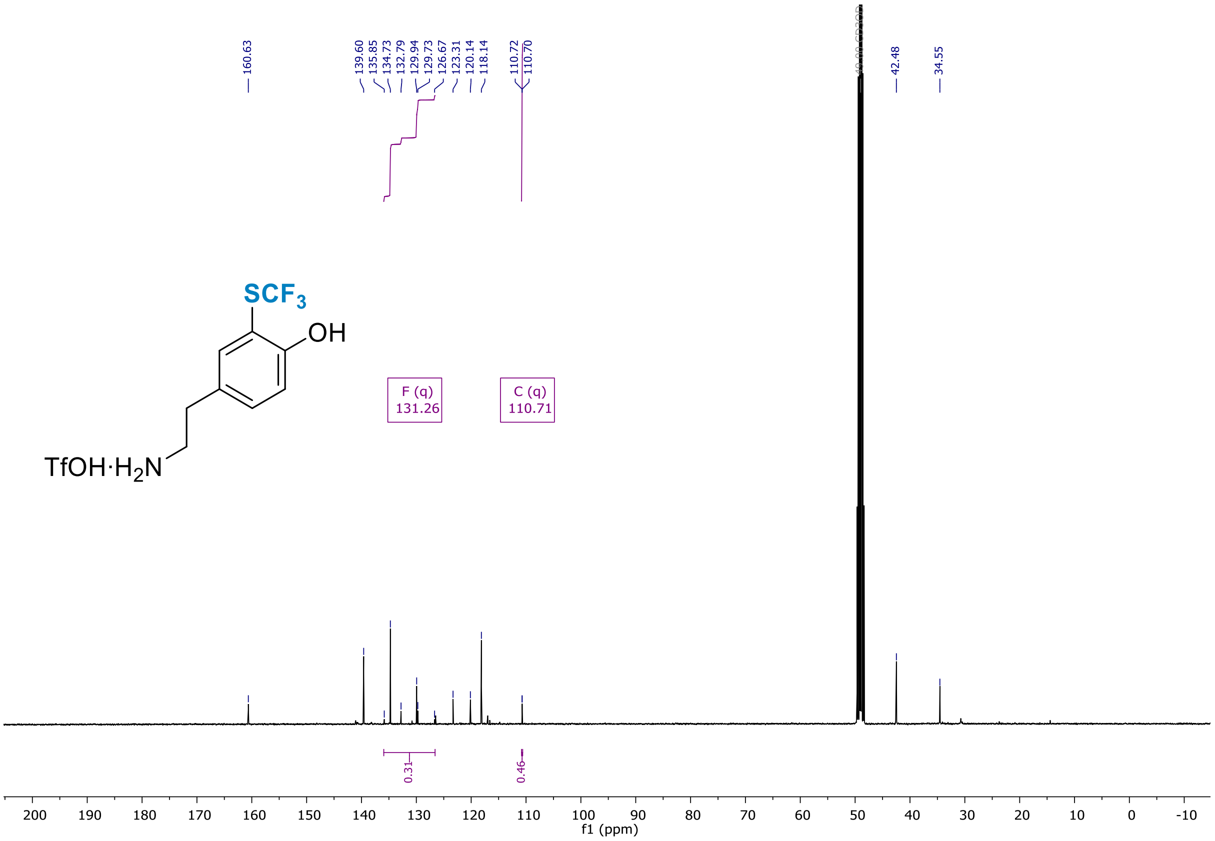

^{19}F NMR spectrum of **6e** in MeOD-d_4, 376 MHz (referenced to C_6F_6 at –165.37 ppm)

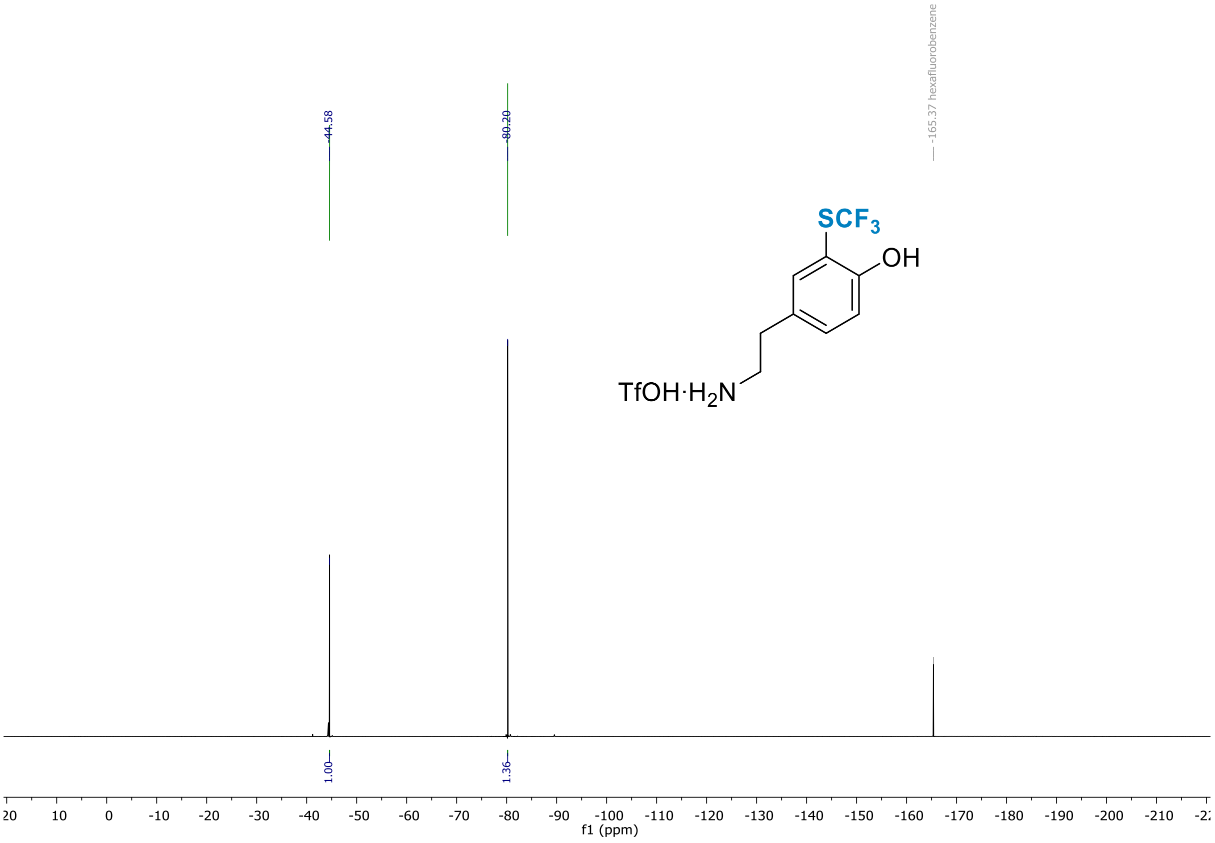

^{1}H NMR spectrum of **6f** in MeOD-d_4, 400 MHz

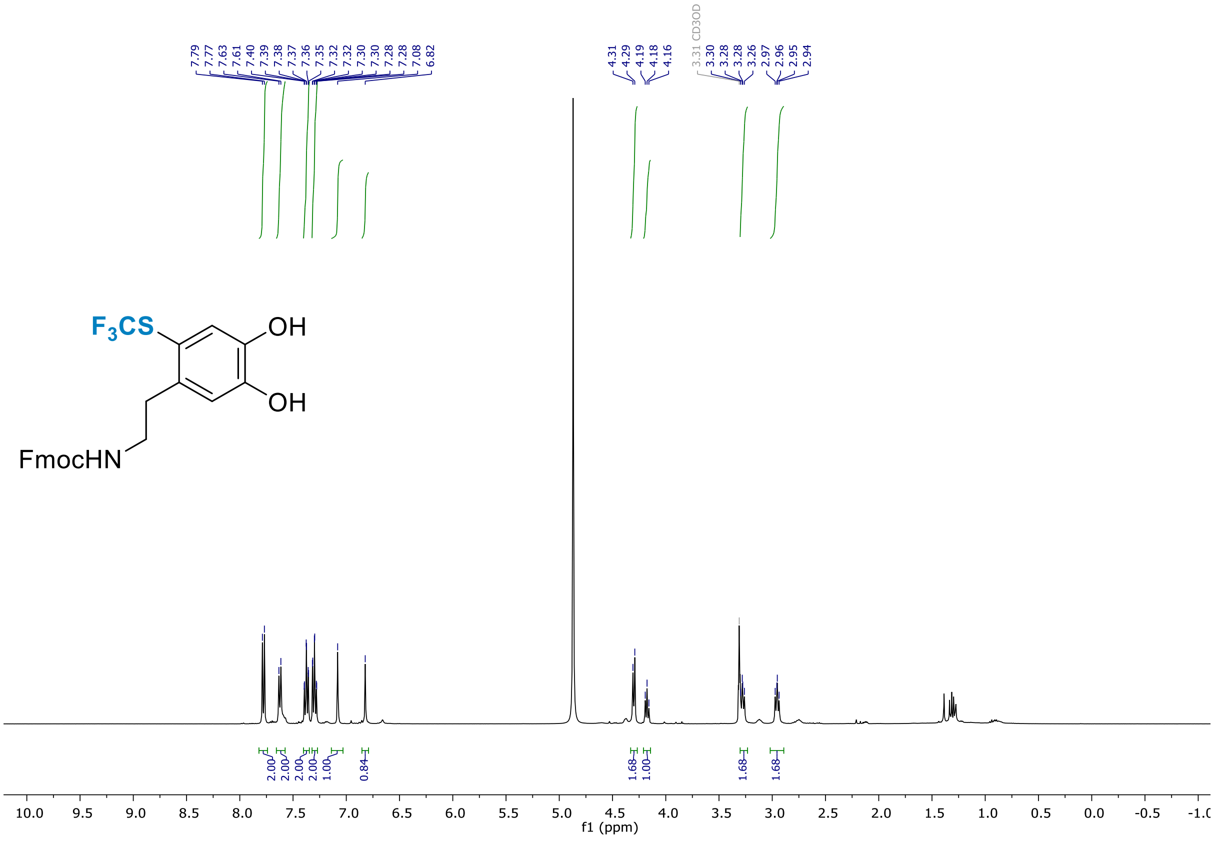

$^{13}C\{^{1}H\}$ NMR spectrum of **6f** in MeOD-d_4, 101 MHz

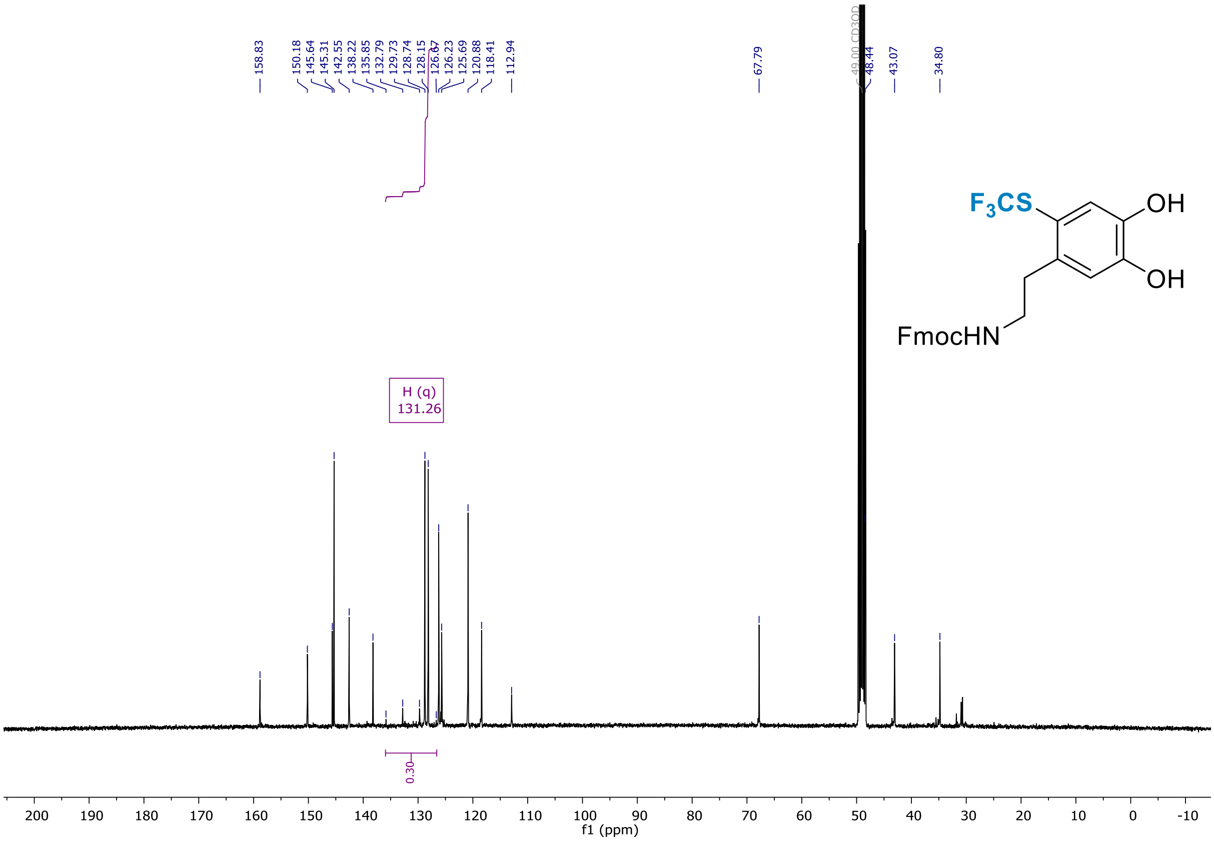

^{19}F NMR spectrum of **6f** in MeOD-d_4, 376 MHz (referenced to C_6F_6 at –165.37 ppm)

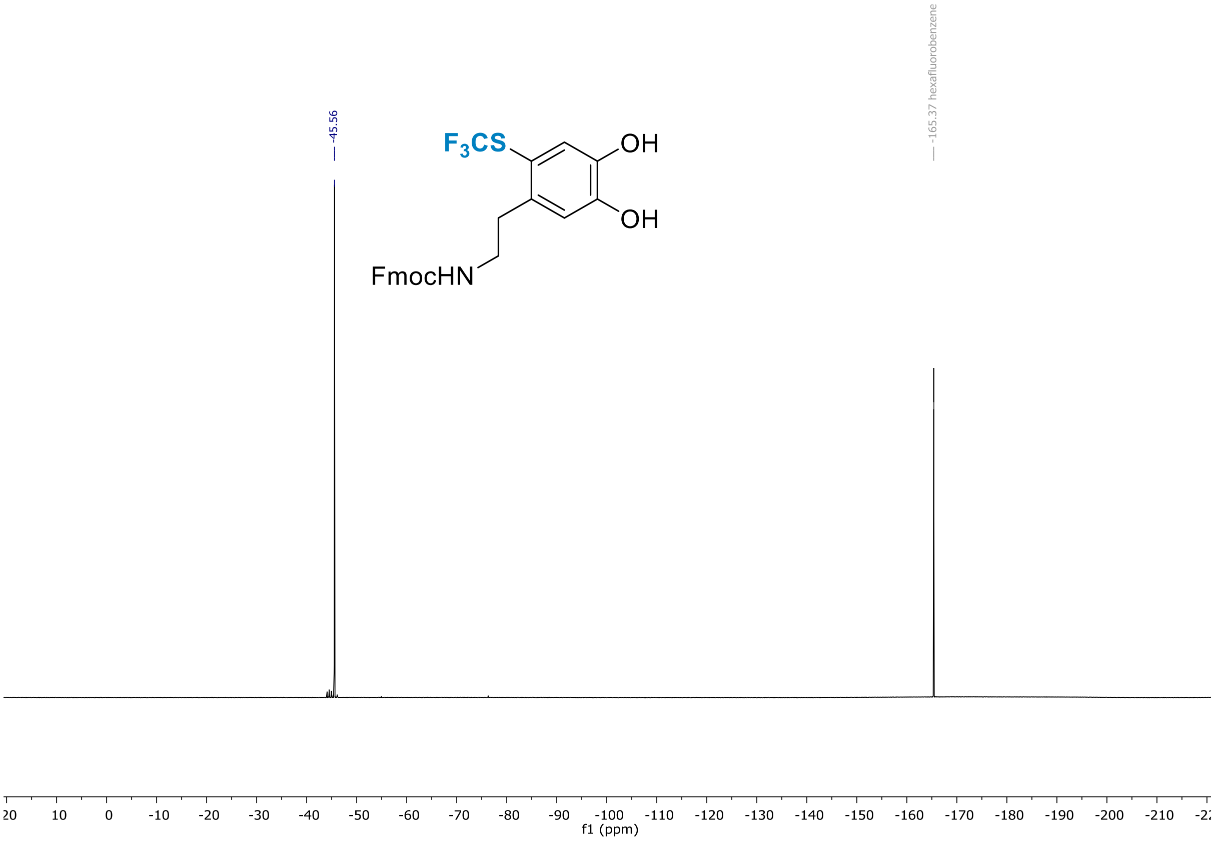

^{1}H NMR spectrum of **6g** in MeOD-d_4, 400 MHz

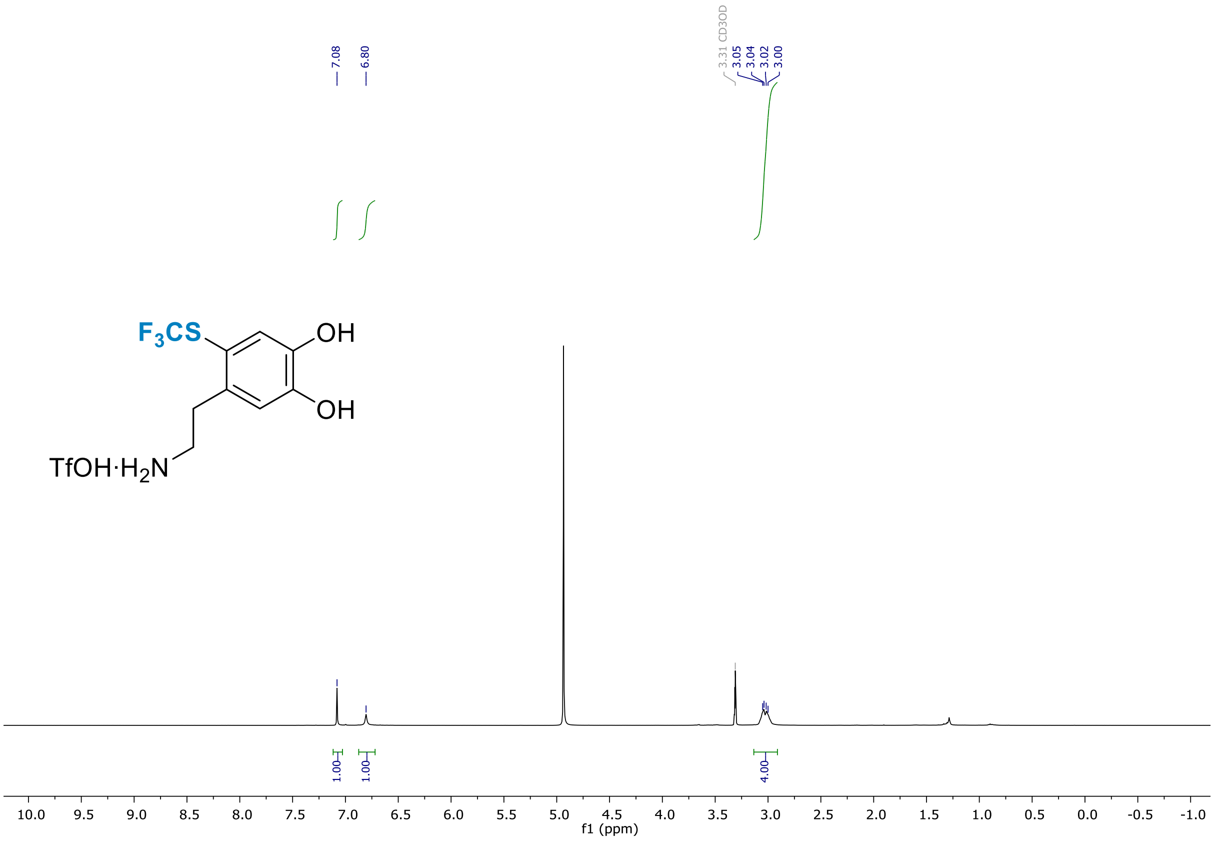

^{13}C{^{1}H} NMR spectrum of **6g** in MeOD-d_4, 101 MHz

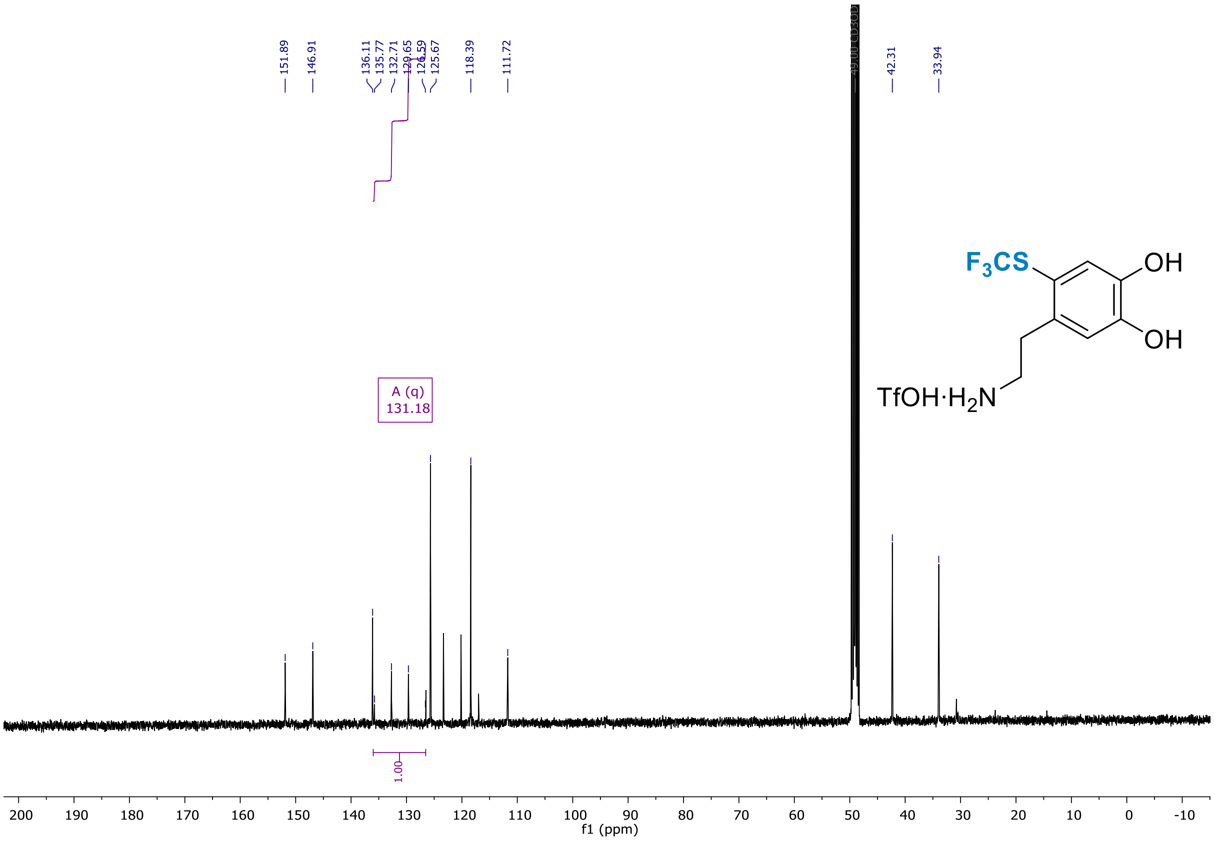

^{19}F NMR spectrum of **6g** in MeOD-*d*$_4$, 376 MHz (referenced to C_6F_6 at –165.37 ppm)

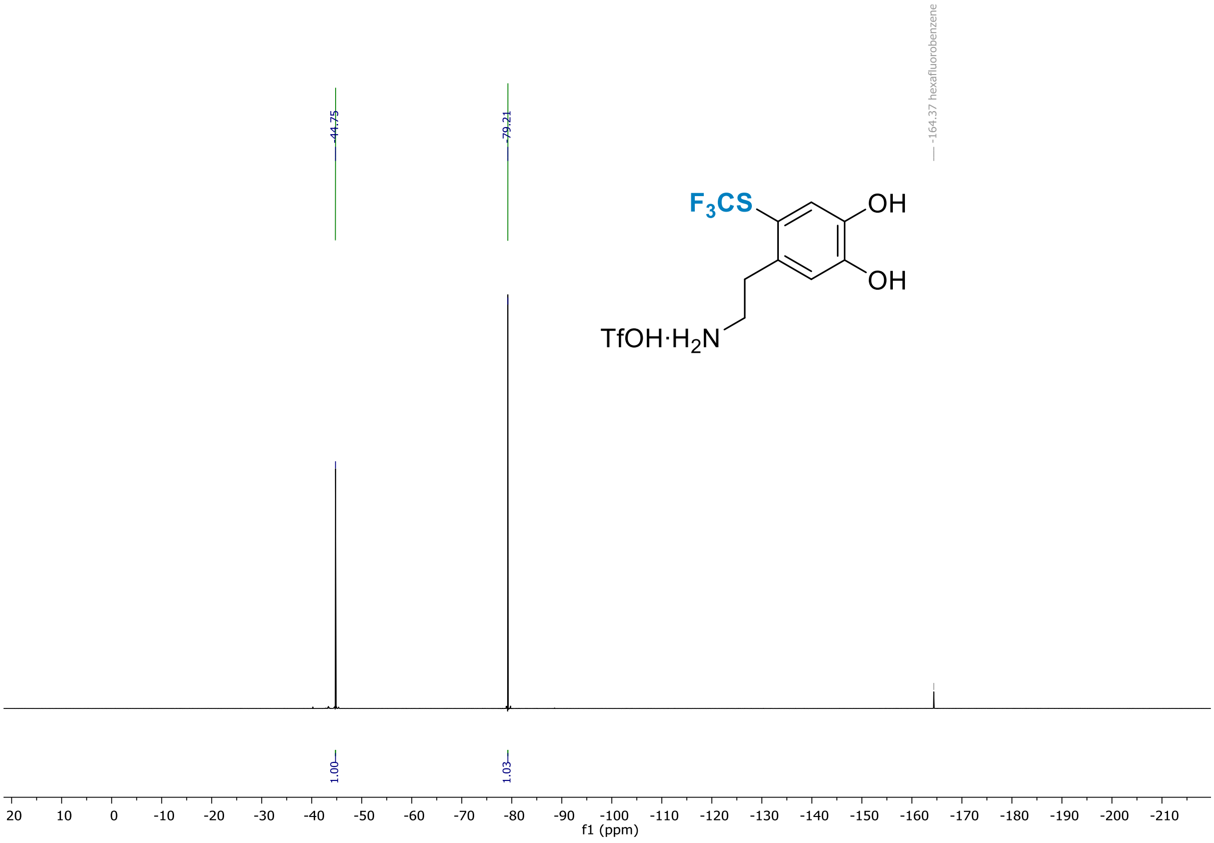

^{1}H NMR spectrum of **6h** in DMSO-*d*$_6$, 400 MHz

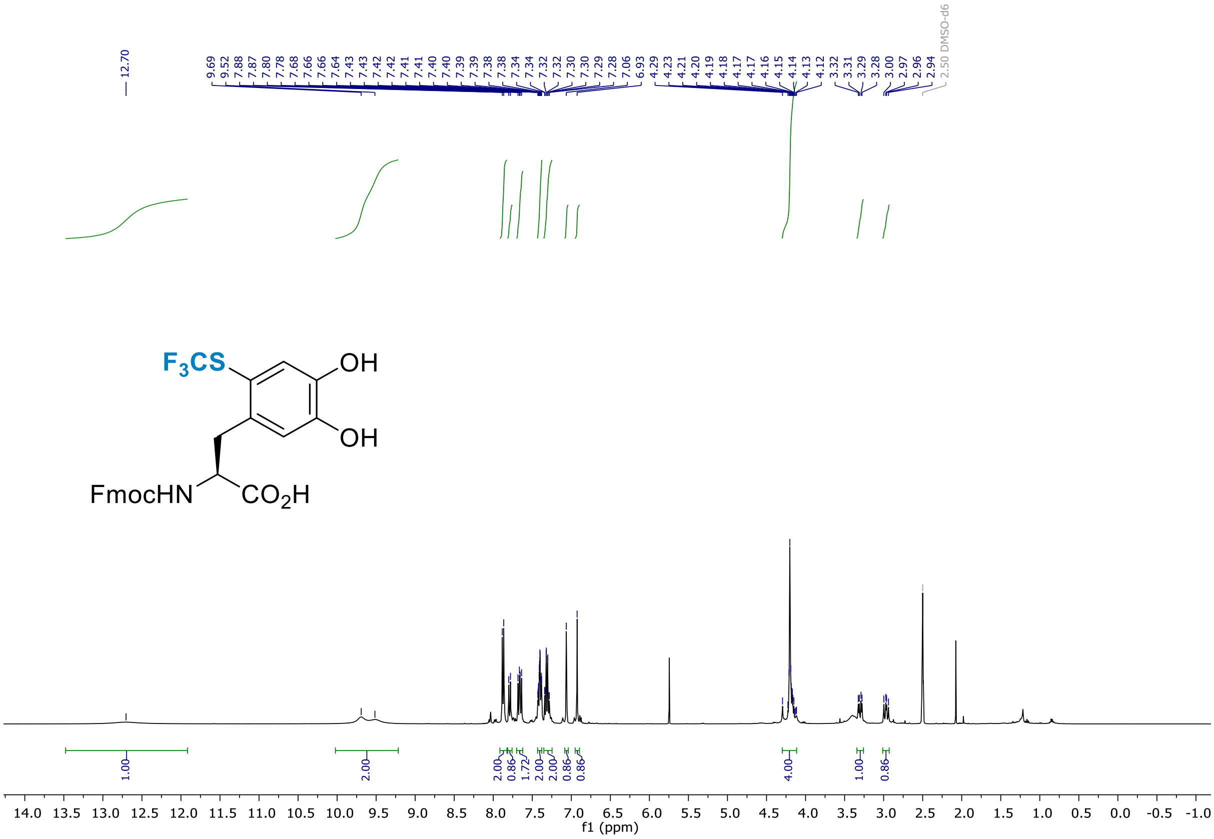

$^{13}C\{^{1}H\}$ NMR spectrum of **6h** in DMSO-d_6, 101 MHz

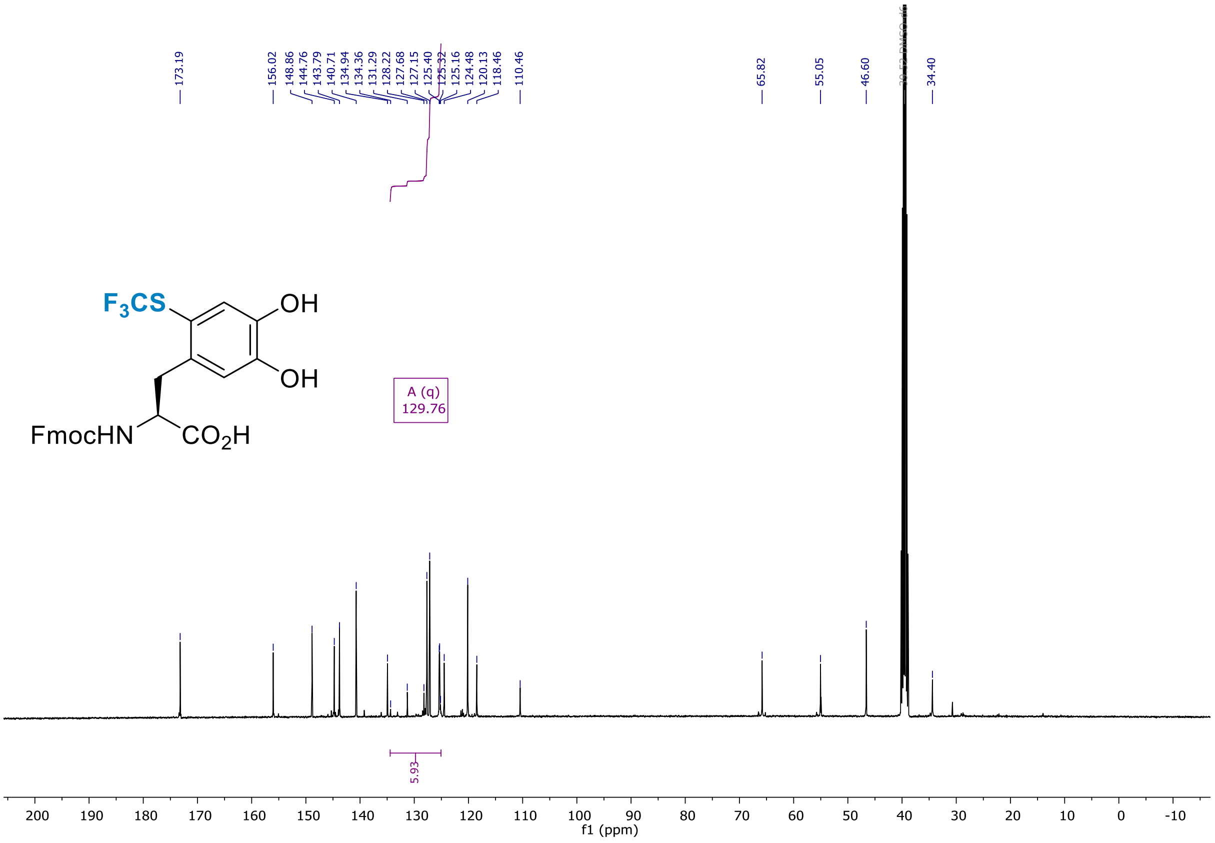

^{19}F NMR spectrum of **6h** in DMSO-d_6, 376 MHz (referenced to C_6F_6 at –162.45 ppm)

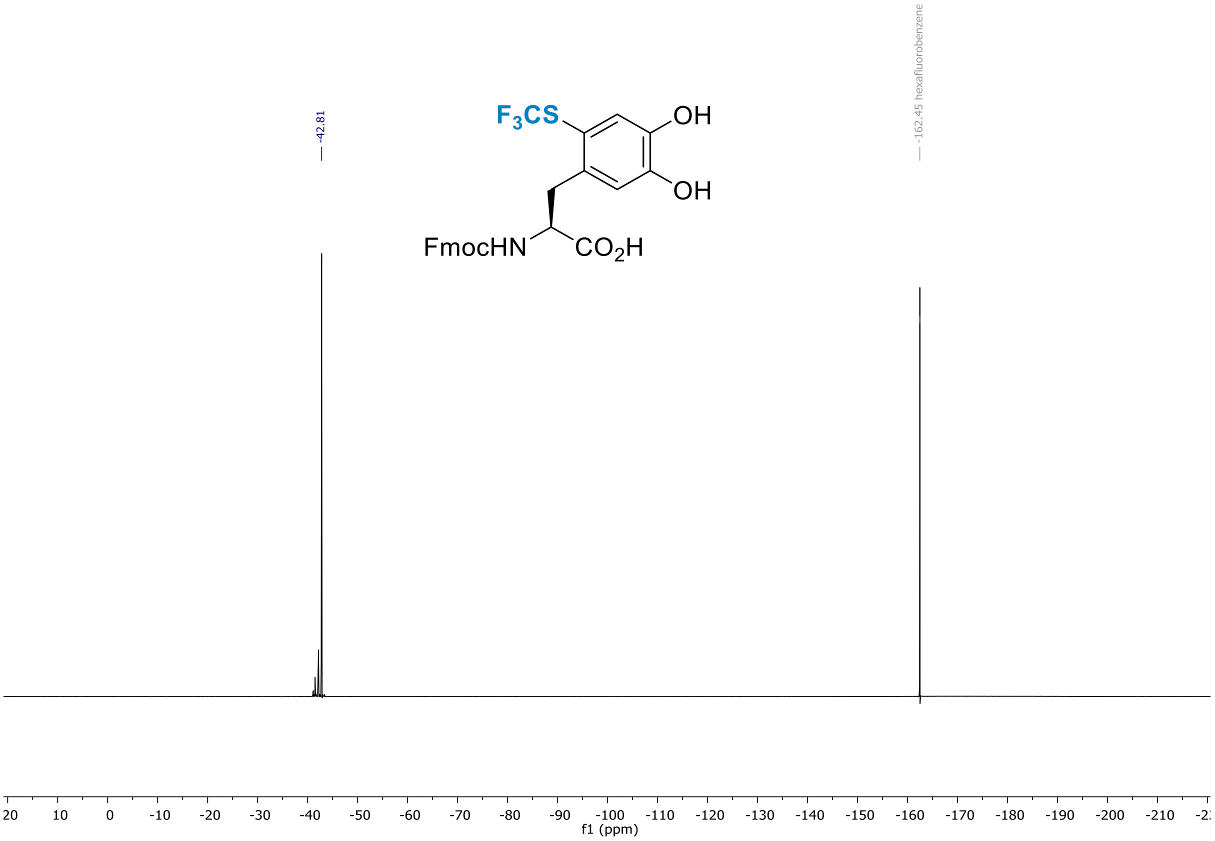

^{1}H NMR spectrum of **6i** in MeOD-d_4, 400 MHz

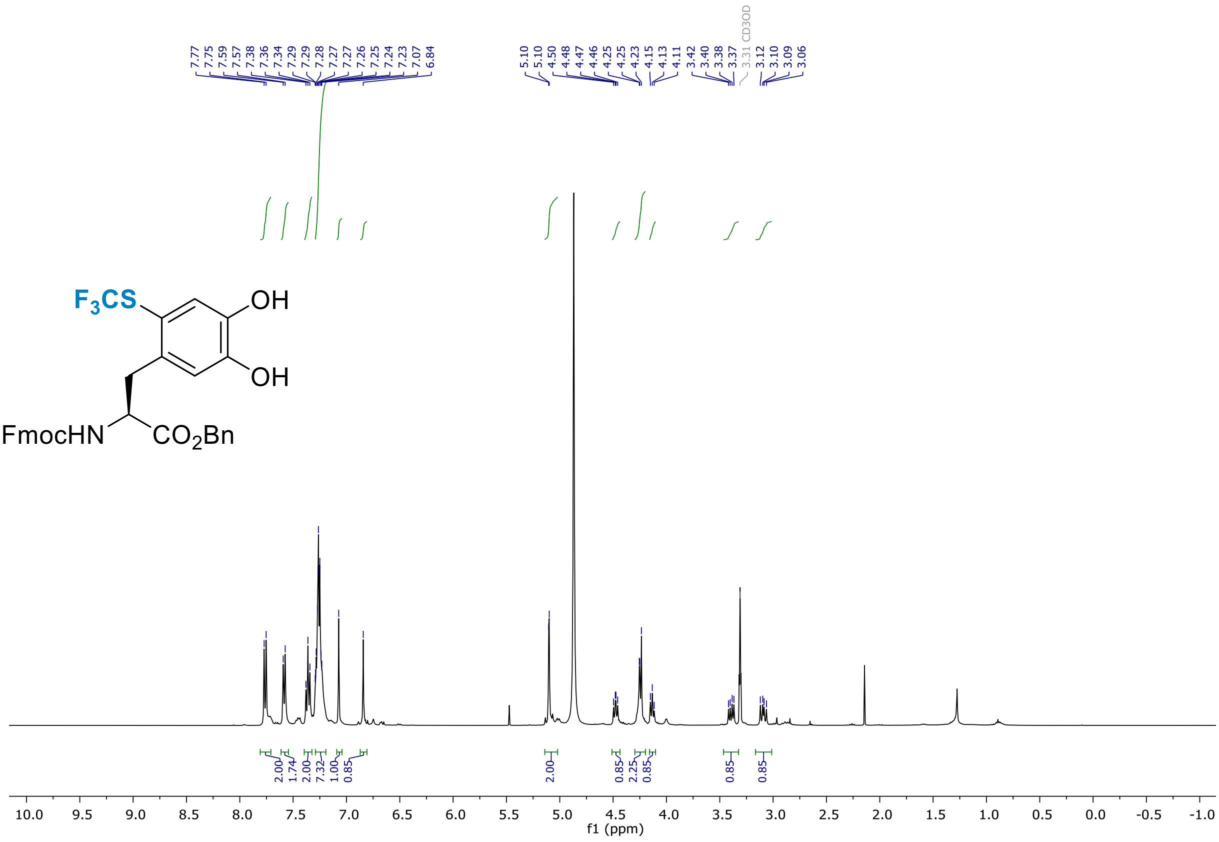

^{13}C{^{1}H} NMR spectrum of **6i** in MeOD-d_4, 101 MHz

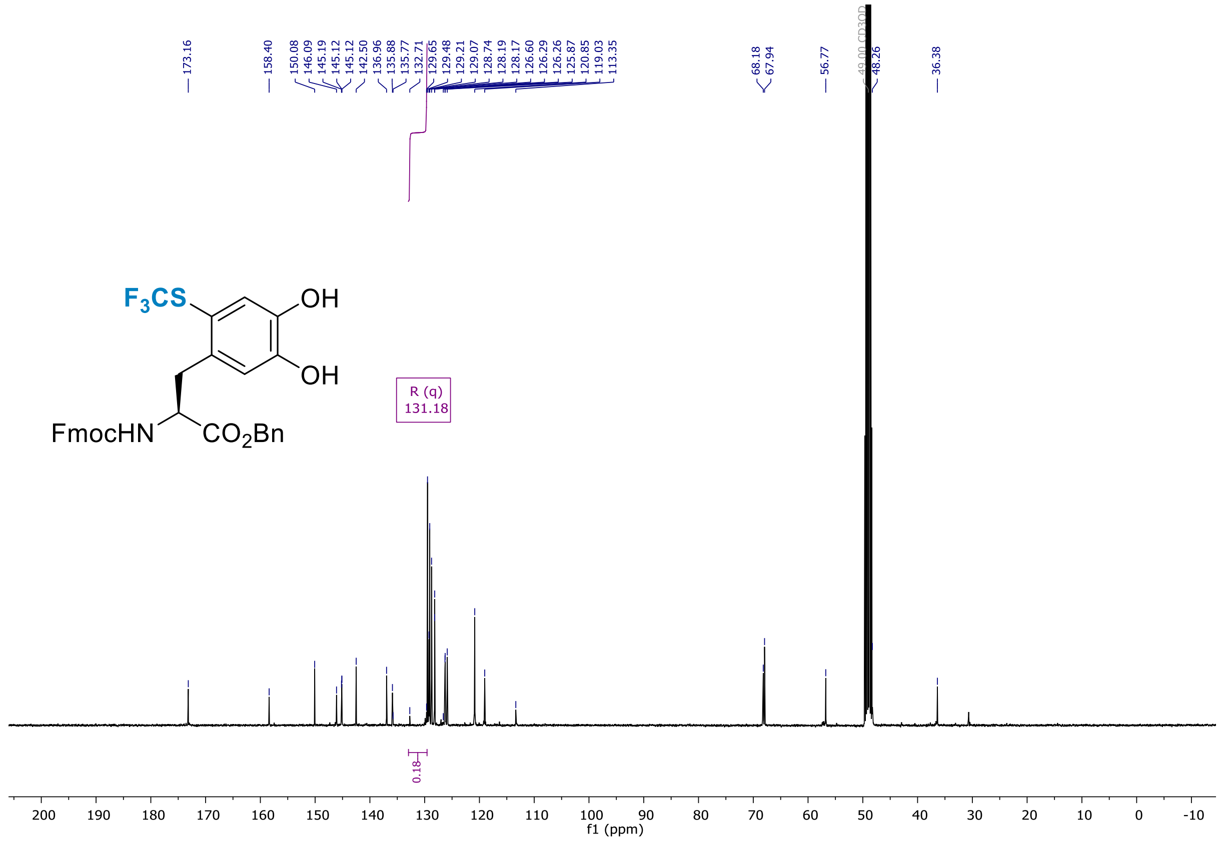

^{19}F NMR spectrum of **6i** in MeOD-d_4, 376 MHz (referenced to C_6F_6 at –165.37 ppm)

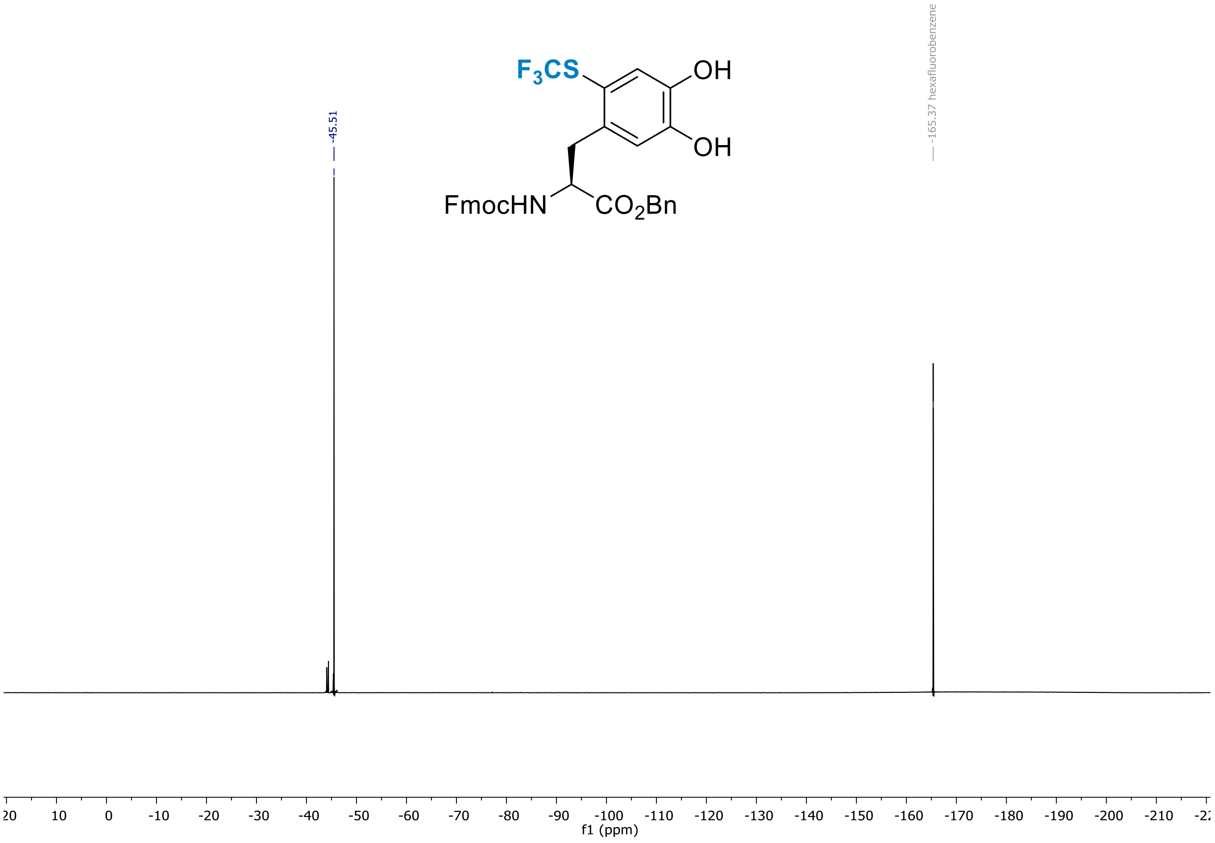

^{1}H NMR spectrum of **8a** in $CDCl_3$, 500 MHz

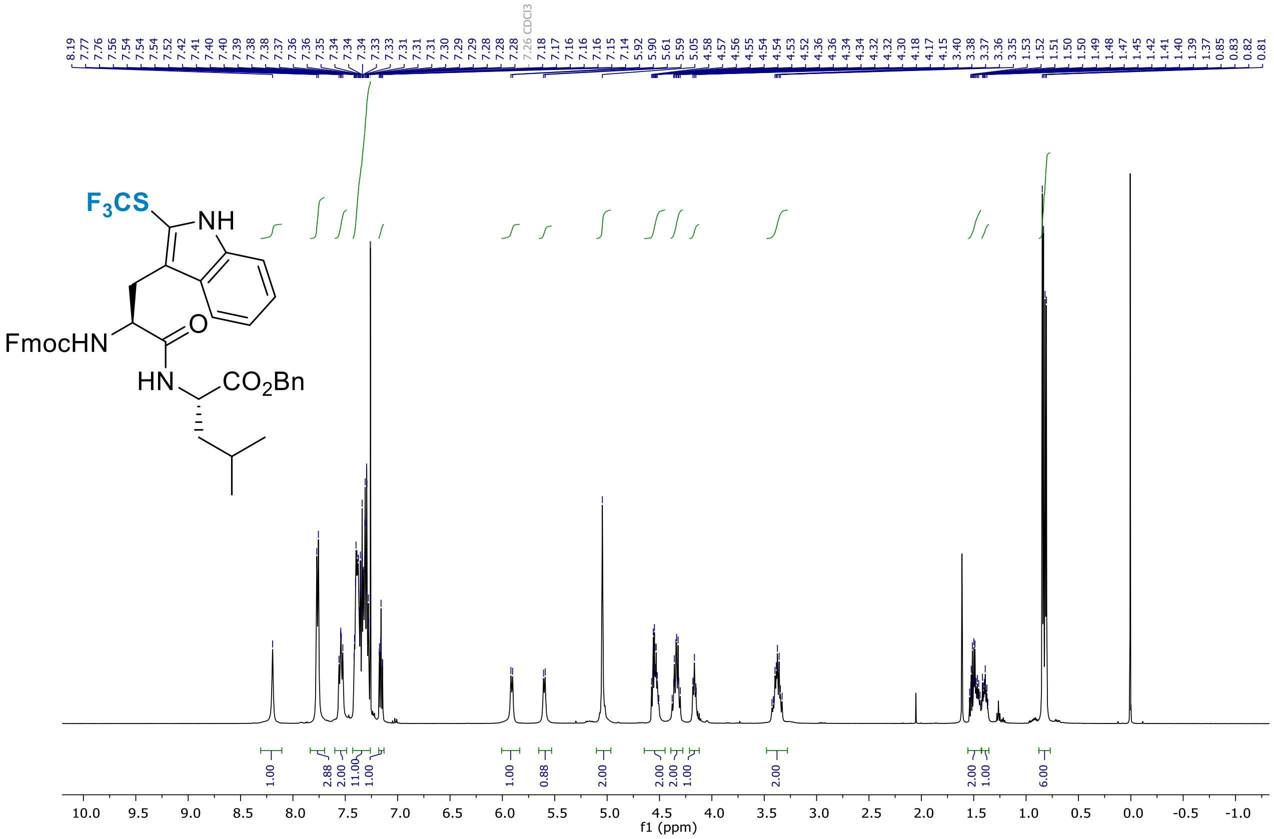

$^{13}C\{^1H\}$ NMR spectrum of **8a** in $CDCl_3$, 126 MHz

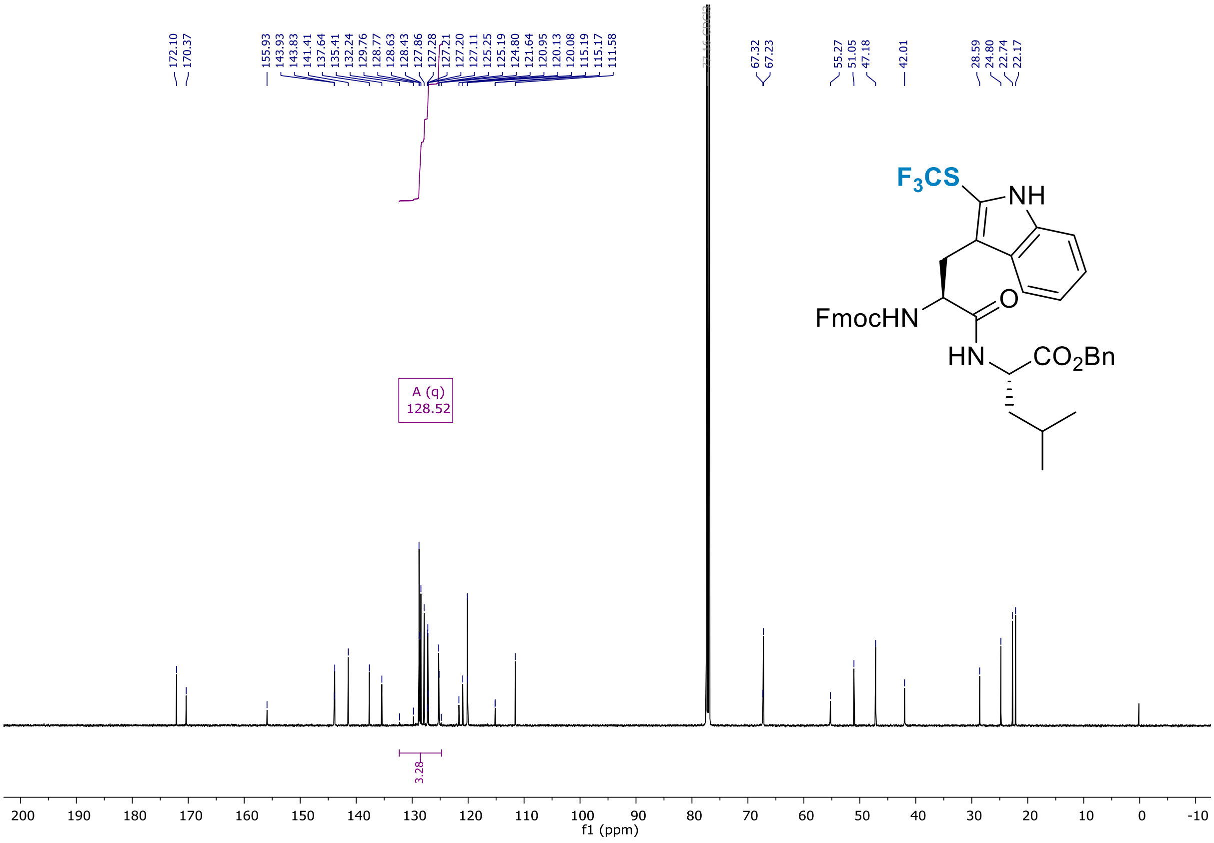

^{19}F NMR spectrum of **8a** in $CDCl_3$, 471 MHz (referenced to $CFCl_3$ at 0.65 ppm)

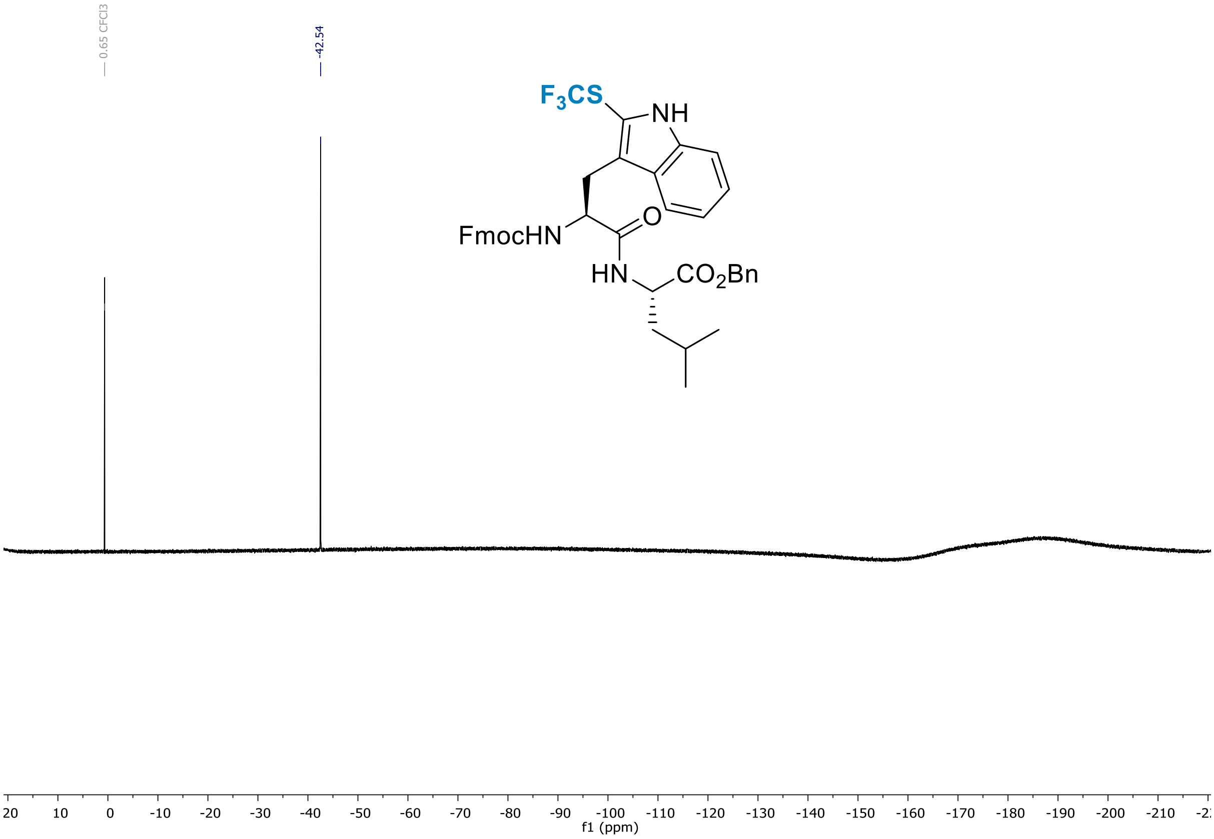

^{1}H NMR spectrum of **8b** in $CDCl_3$, 500 MHz

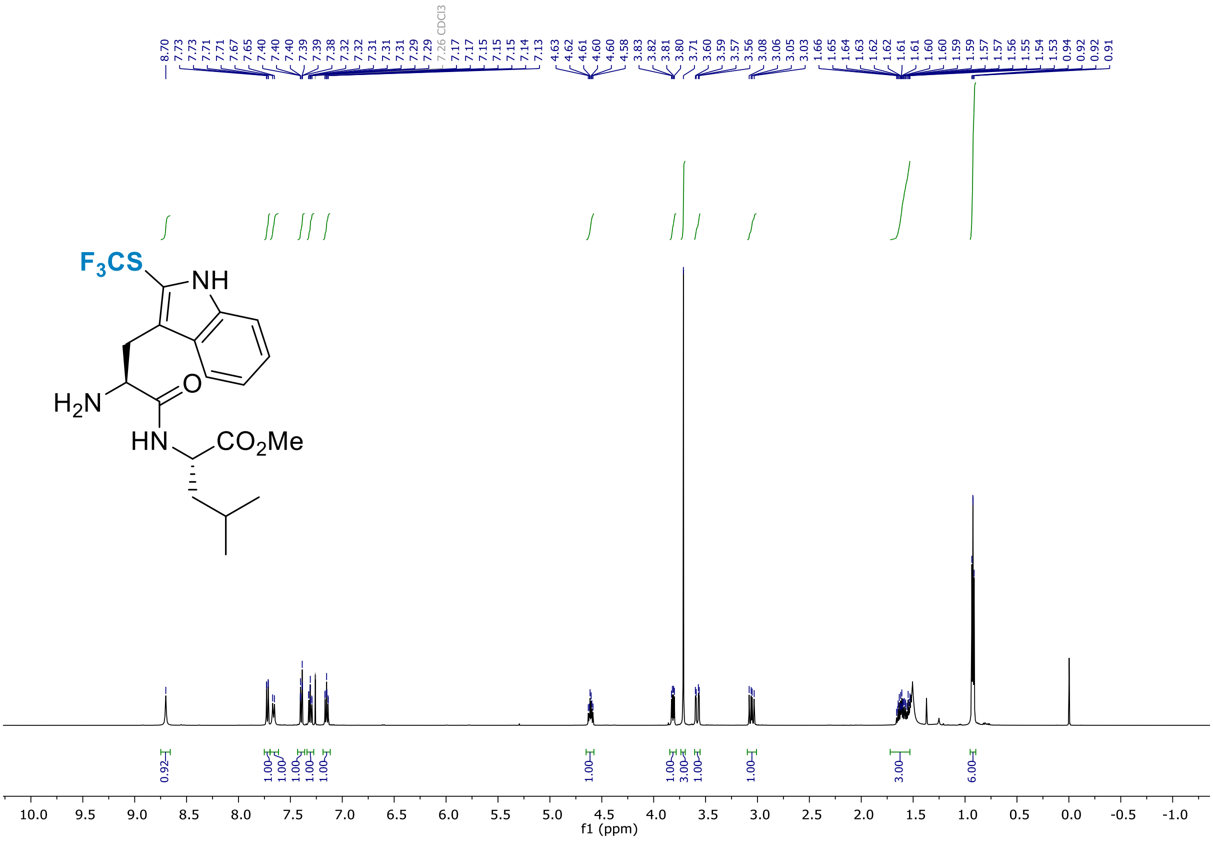

$^{13}C\{^1H\}$ NMR spectrum of **8b** in $CDCl_3$, 126 MHz

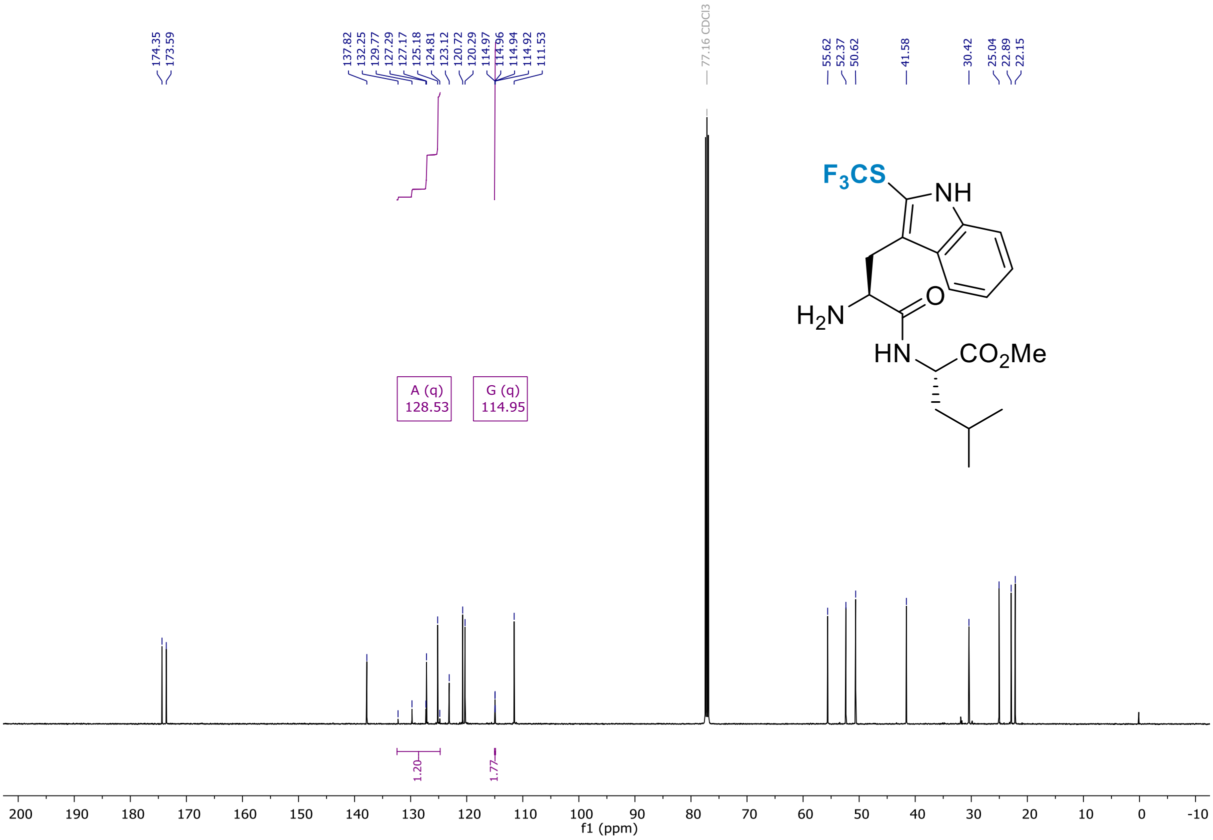

^{19}F NMR spectrum of **8b** in $CDCl_3$, 471 MHz (referenced to $CFCl_3$ at 0.65 ppm)

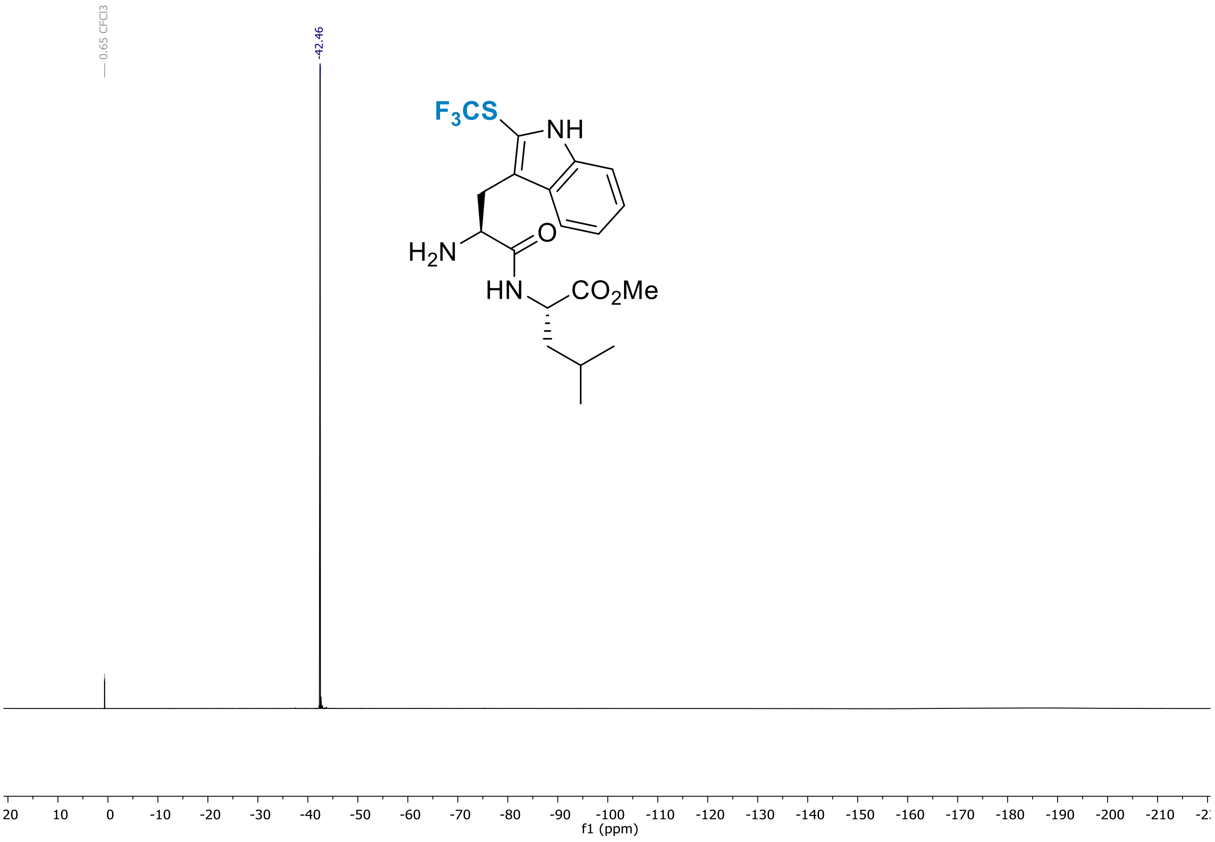

^{1}H NMR spectrum of **8c** in $CDCl_3$, 400 MHz

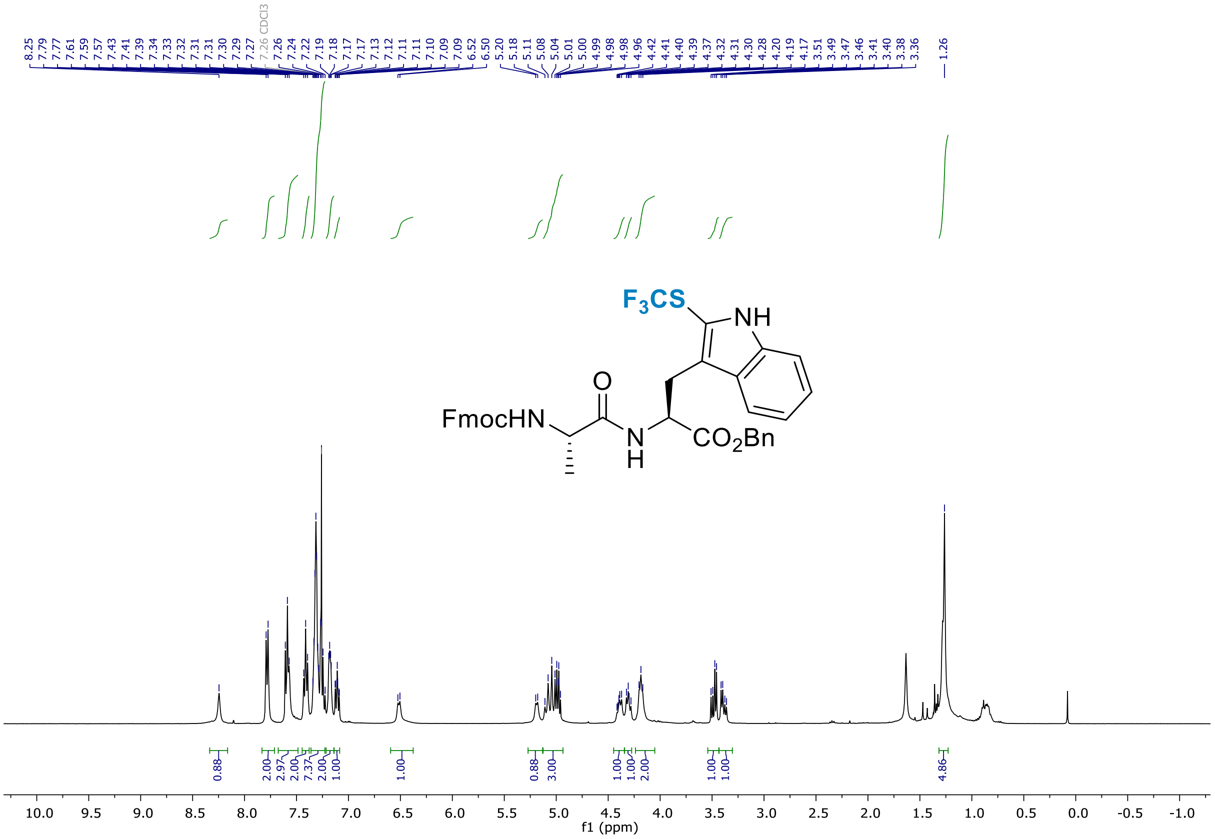

$^{13}C\{^1H\}$ NMR spectrum of **8c** in $CDCl_3$, 101 MHz

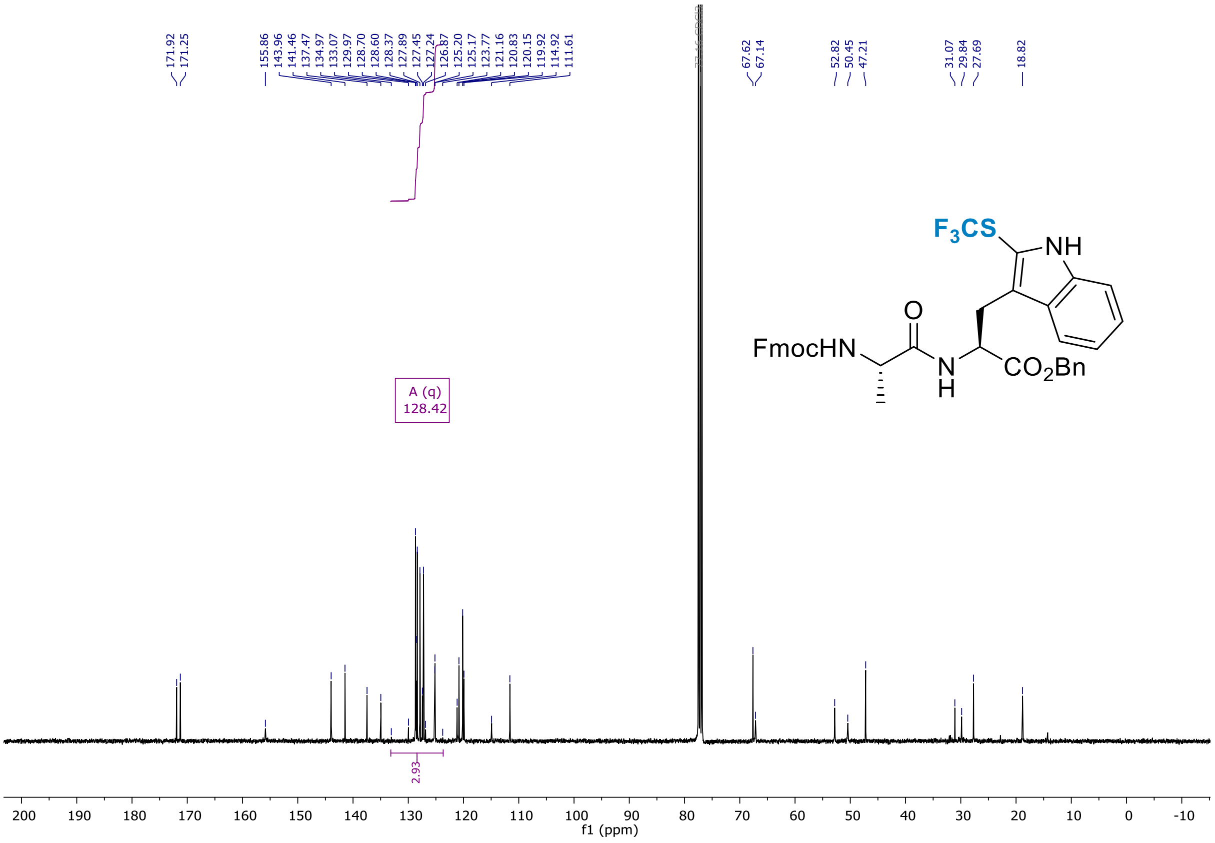

^{19}F NMR spectrum of **8c** in $CDCl_3$, 376 MHz (referenced to C_6F_6 at –161.64 ppm)

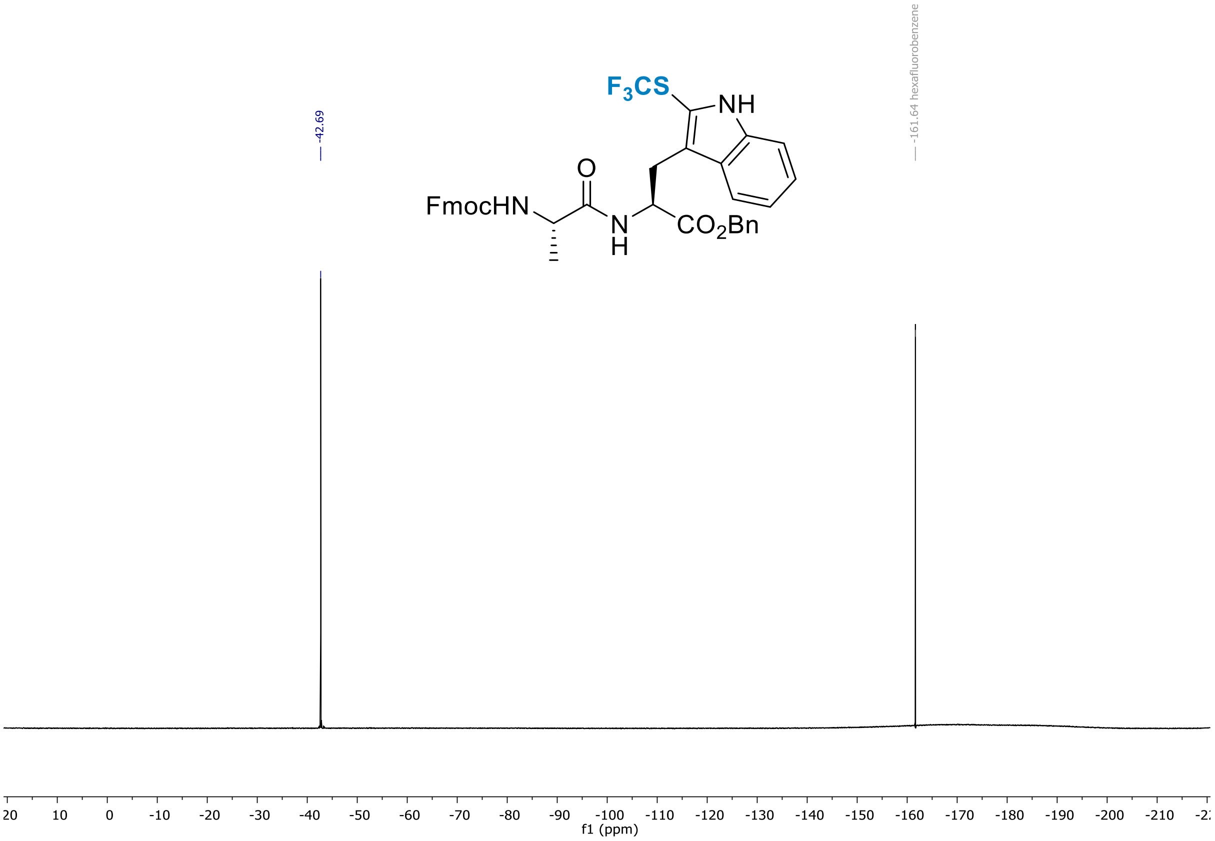

^{1}H NMR spectrum of **8d** in DMSO-d_6, 500 MHz

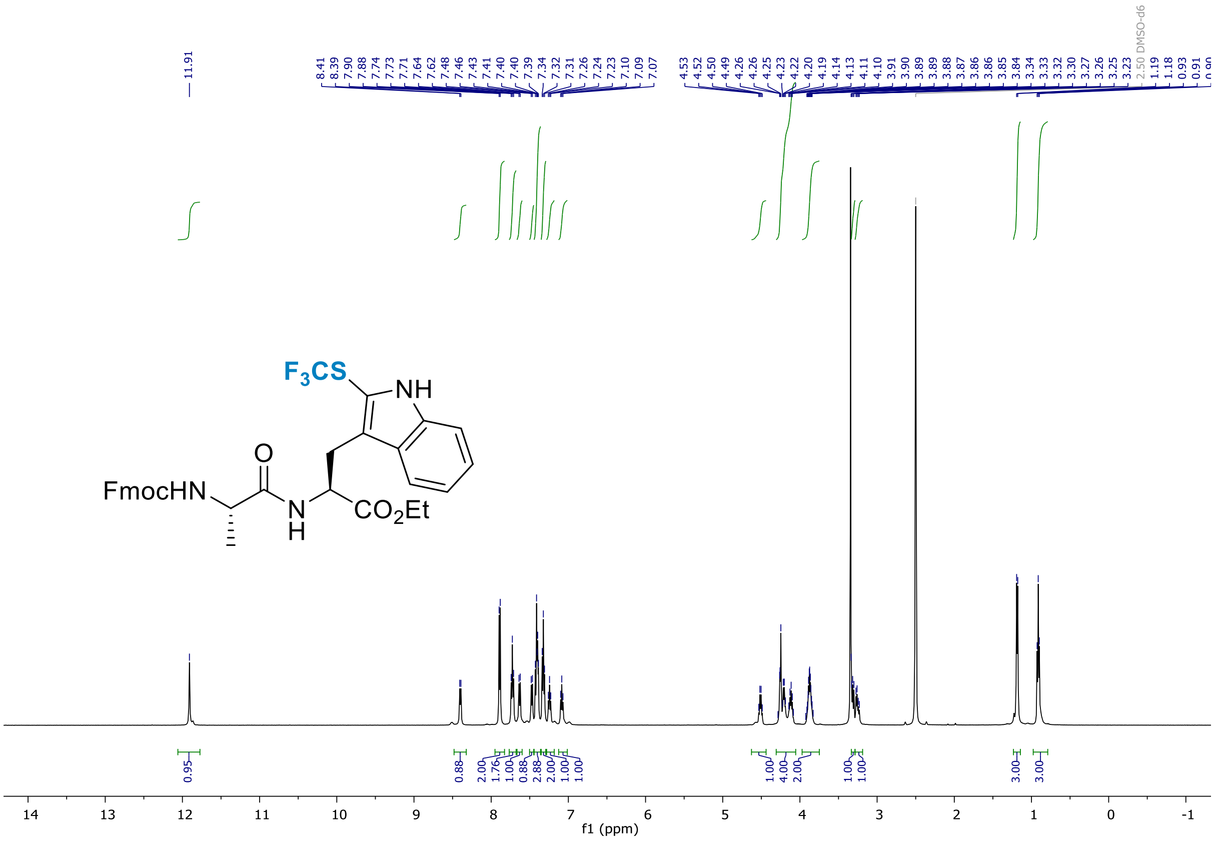

$^{13}C\{^{1}H\}$ NMR spectrum of **8d** in DMSO-d_6, 126 MHz

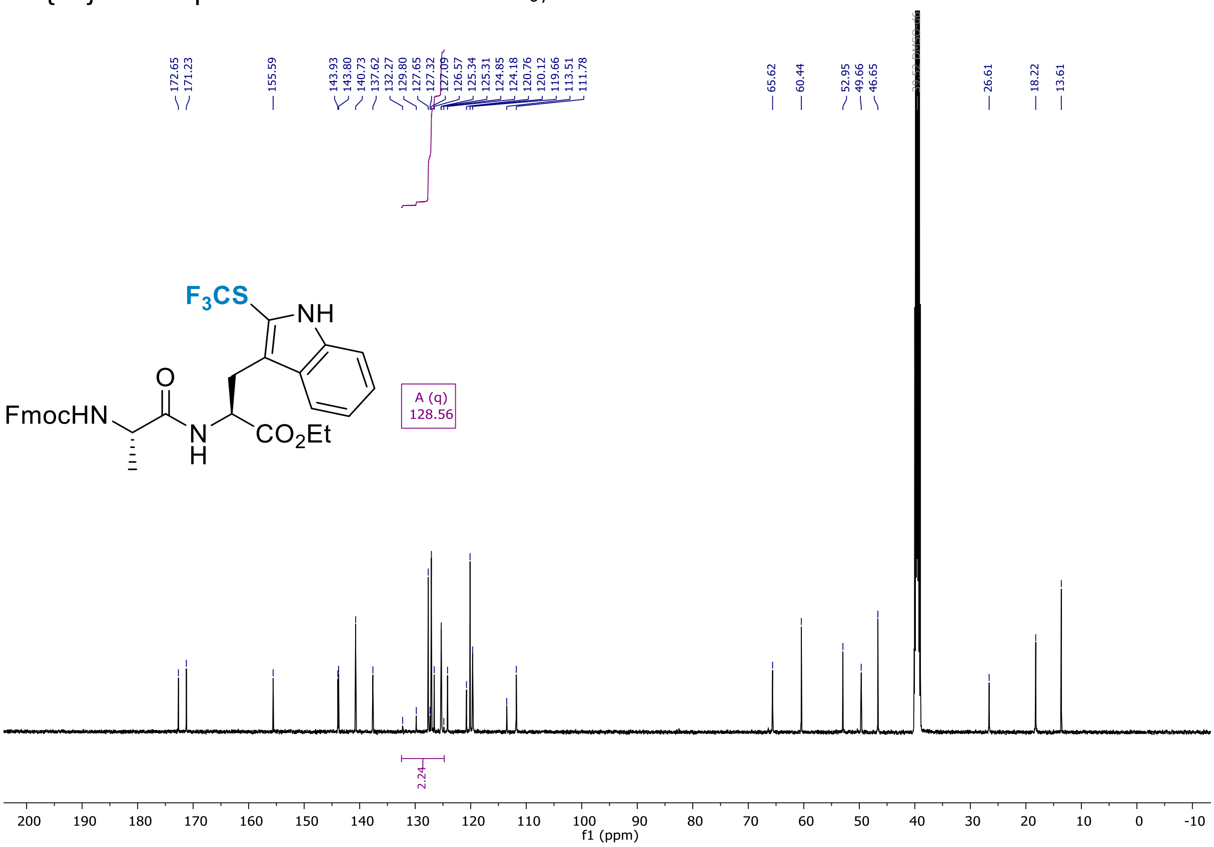

^{19}F NMR spectrum of **8d** in DMSO-d_6, 471 MHz (referenced to $CFCl_3$ at –0.24 ppm)

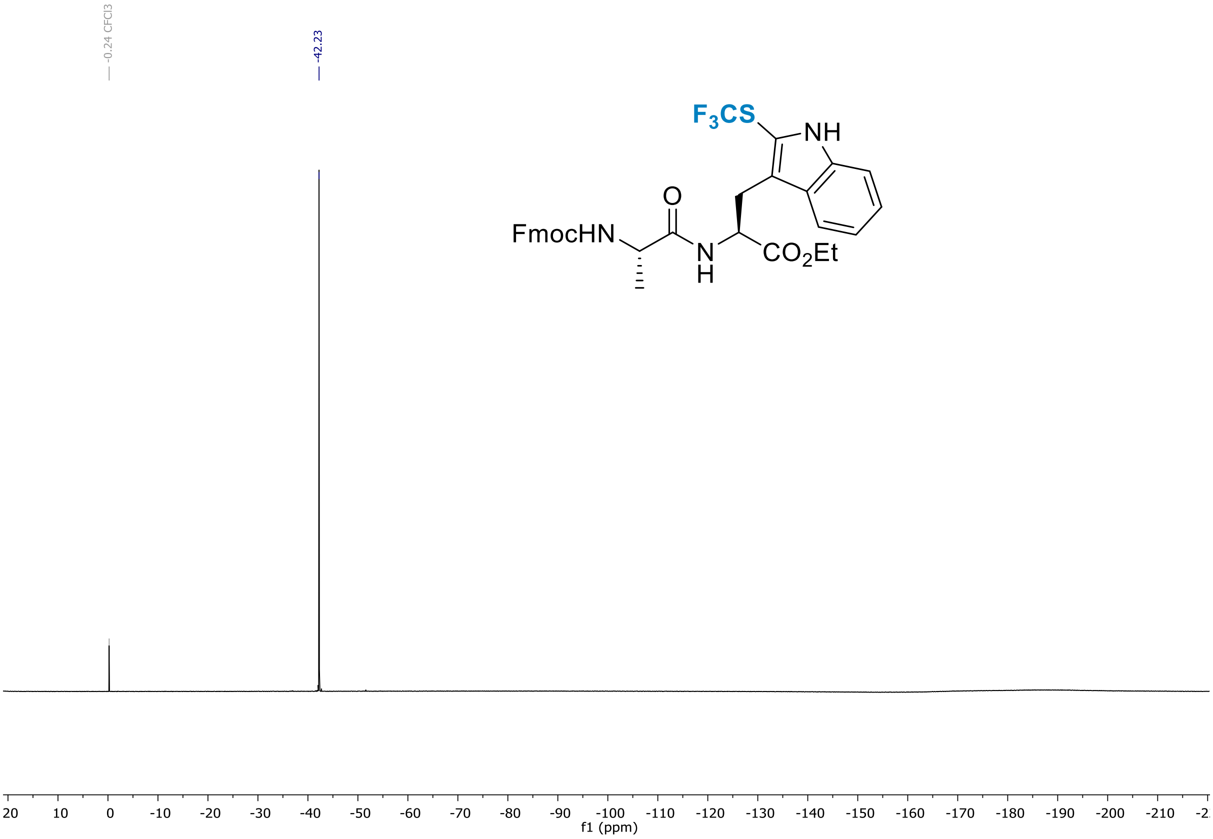

^{1}H NMR spectrum of **8e** in DMSO-d_6, 500 MHz

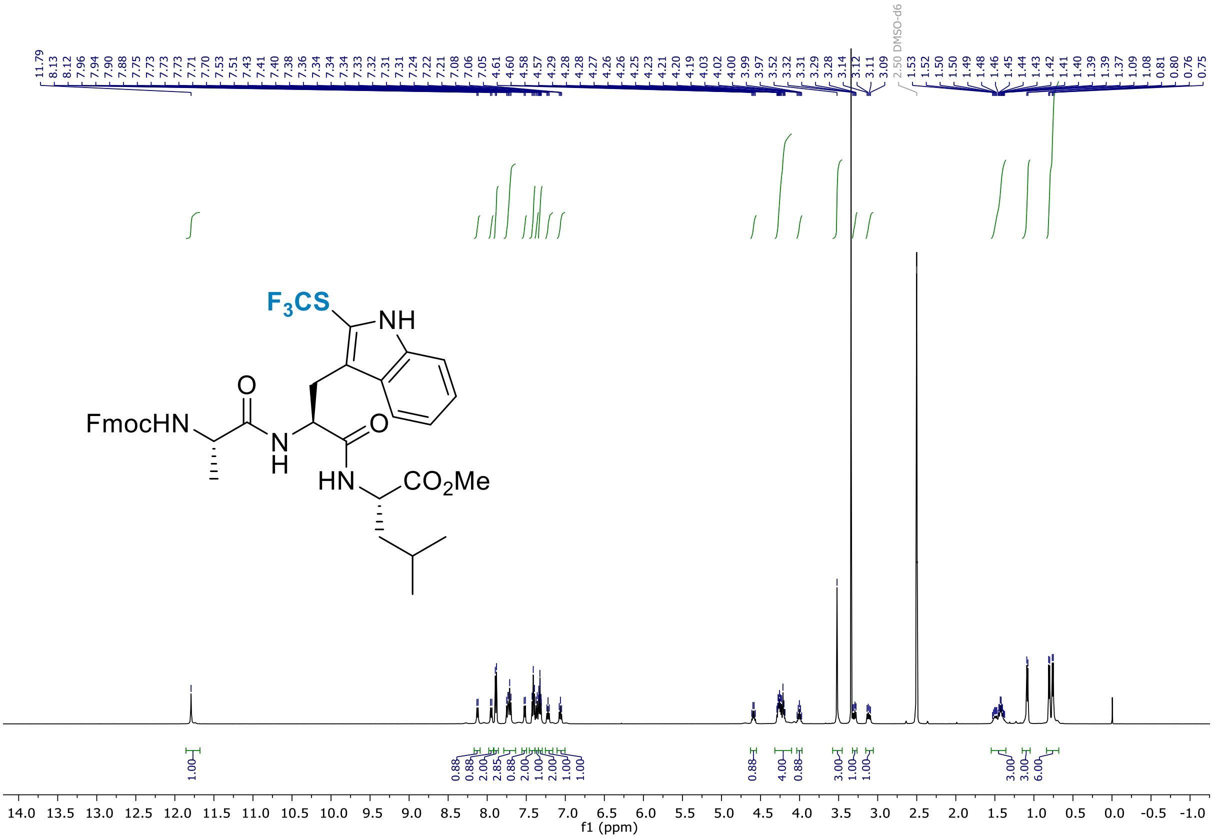

$^{13}C\{^{1}H\}$ NMR spectrum of **8e** in DMSO-d_6, 126 MHz

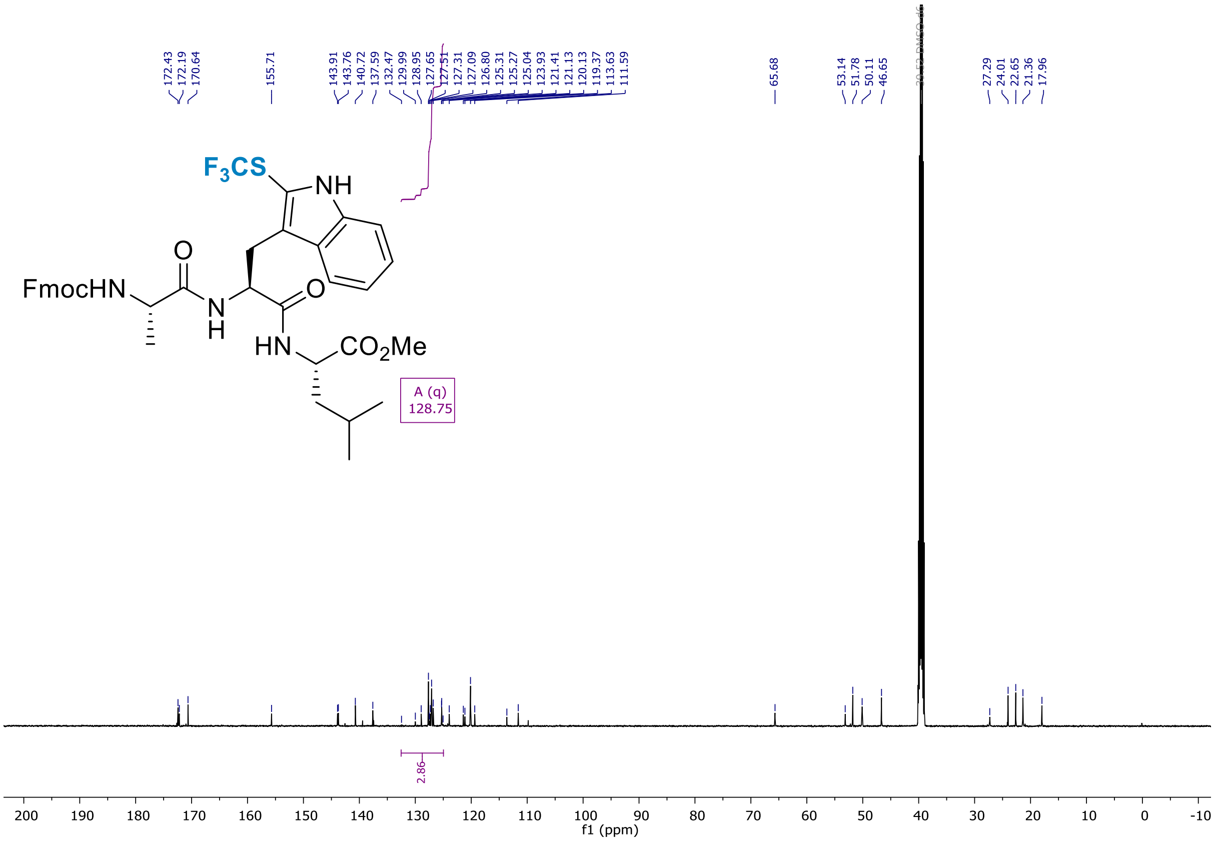

^{19}F NMR spectrum of **8e** in DMSO-d_6, 471 MHz (referenced to $CFCl_3$ at –0.24 ppm)

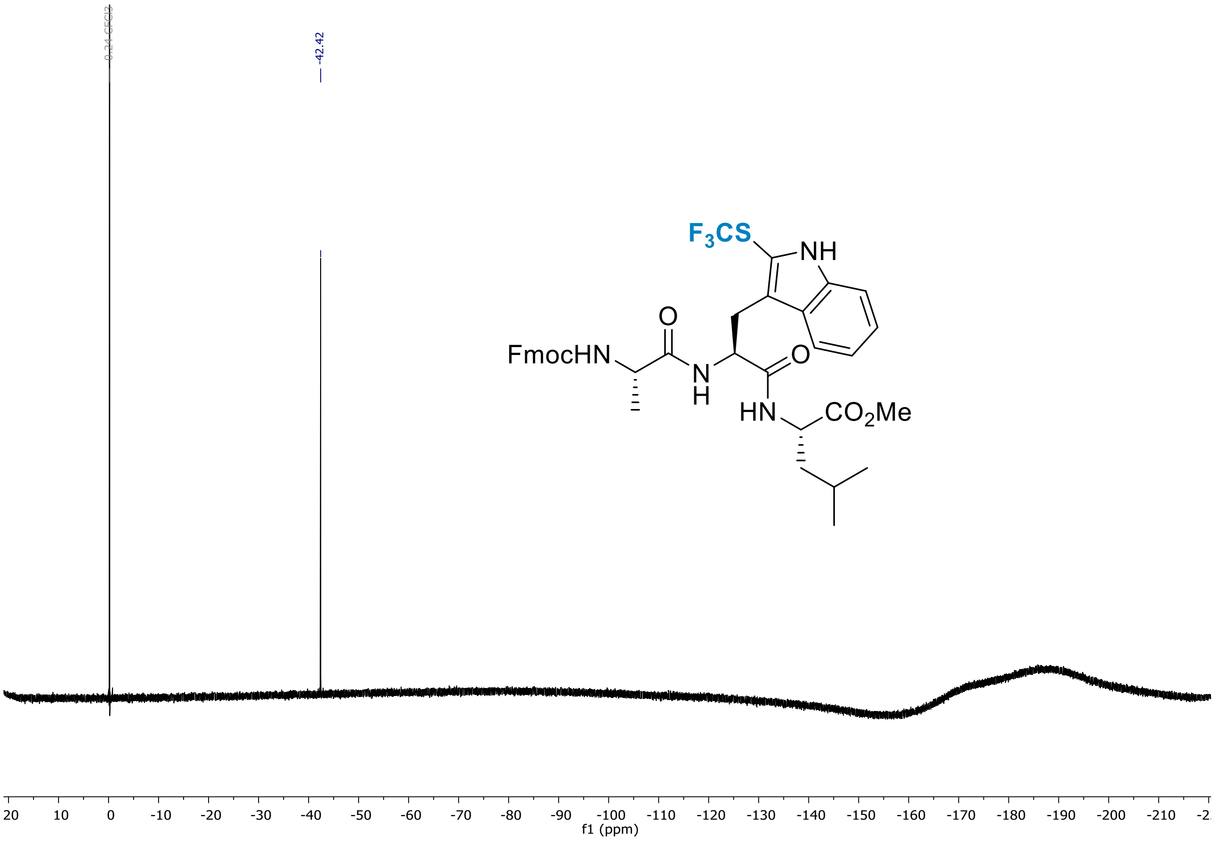

^{1}H NMR spectrum of **8f** in DMSO-d_6, 400 MHz

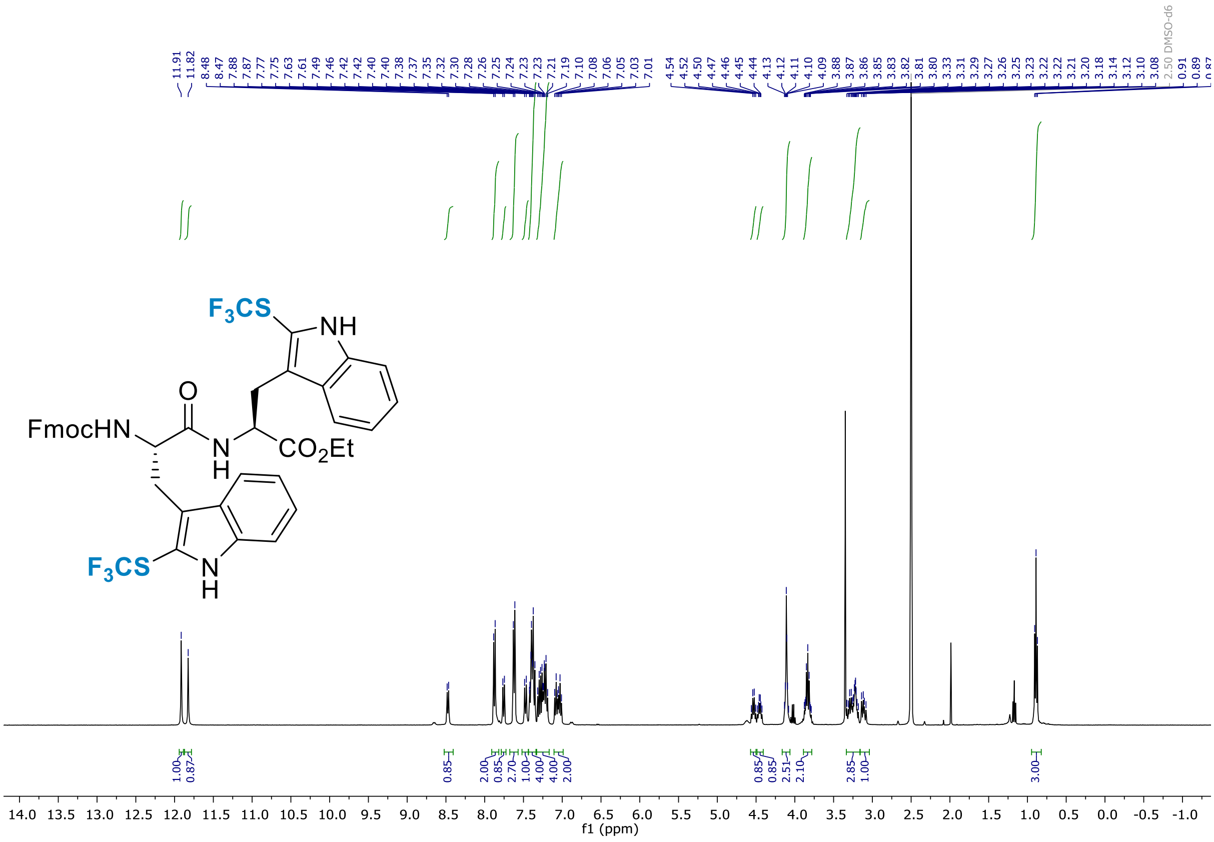

^{13}C{^{1}H} NMR spectrum of **8f** in DMSO-d_6, 101 MHz

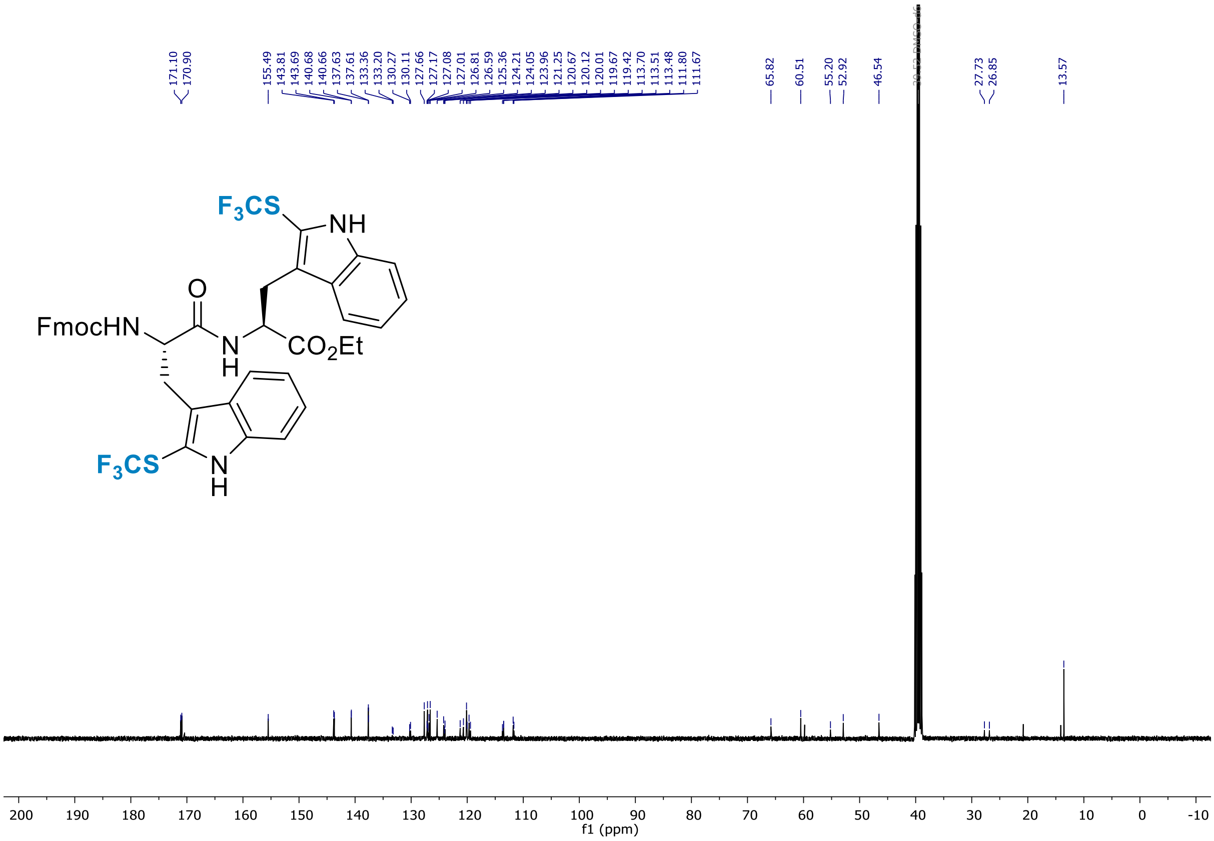

^{19}F NMR spectrum of **8f** in DMSO-d_6, 376 MHz (referenced to C_6F_6 at –162.45 ppm)

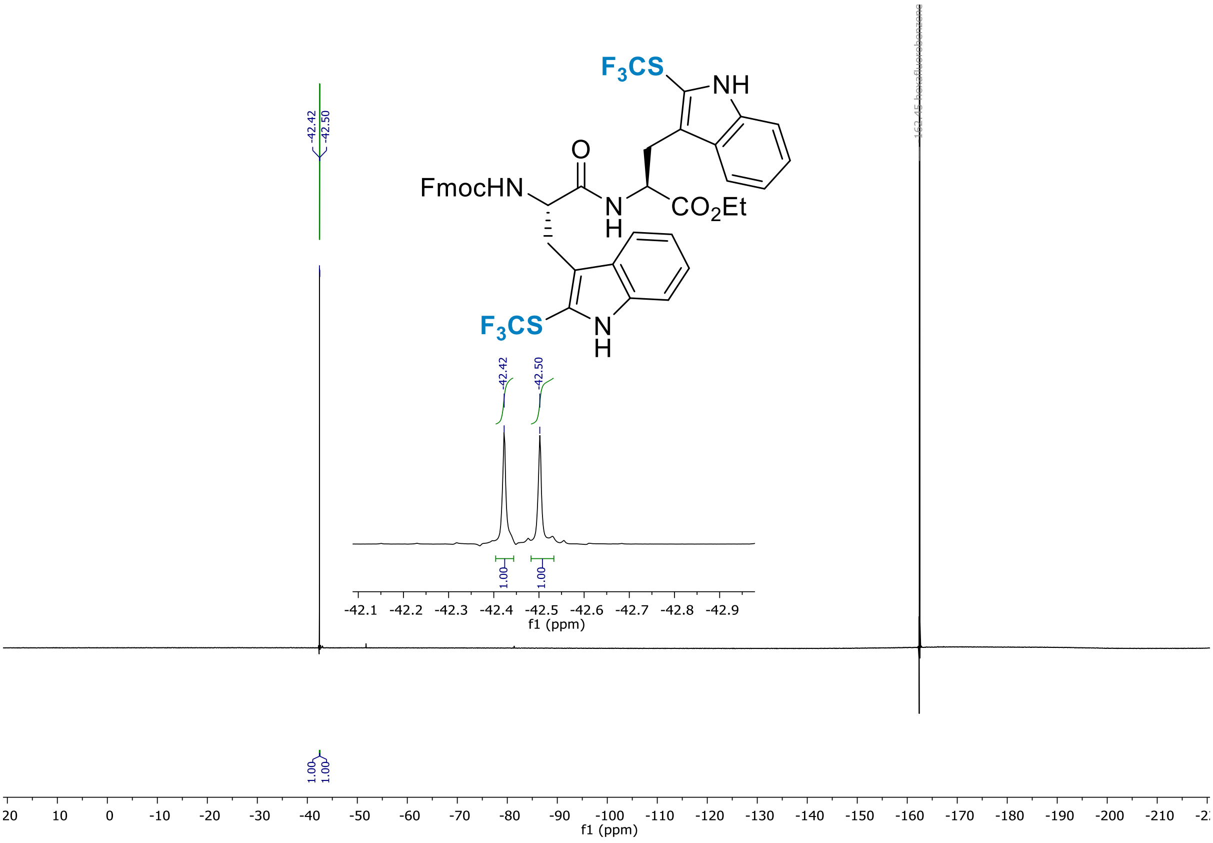

^{1}H NMR spectrum of **8g** in DMSO-d_6, 500 MHz

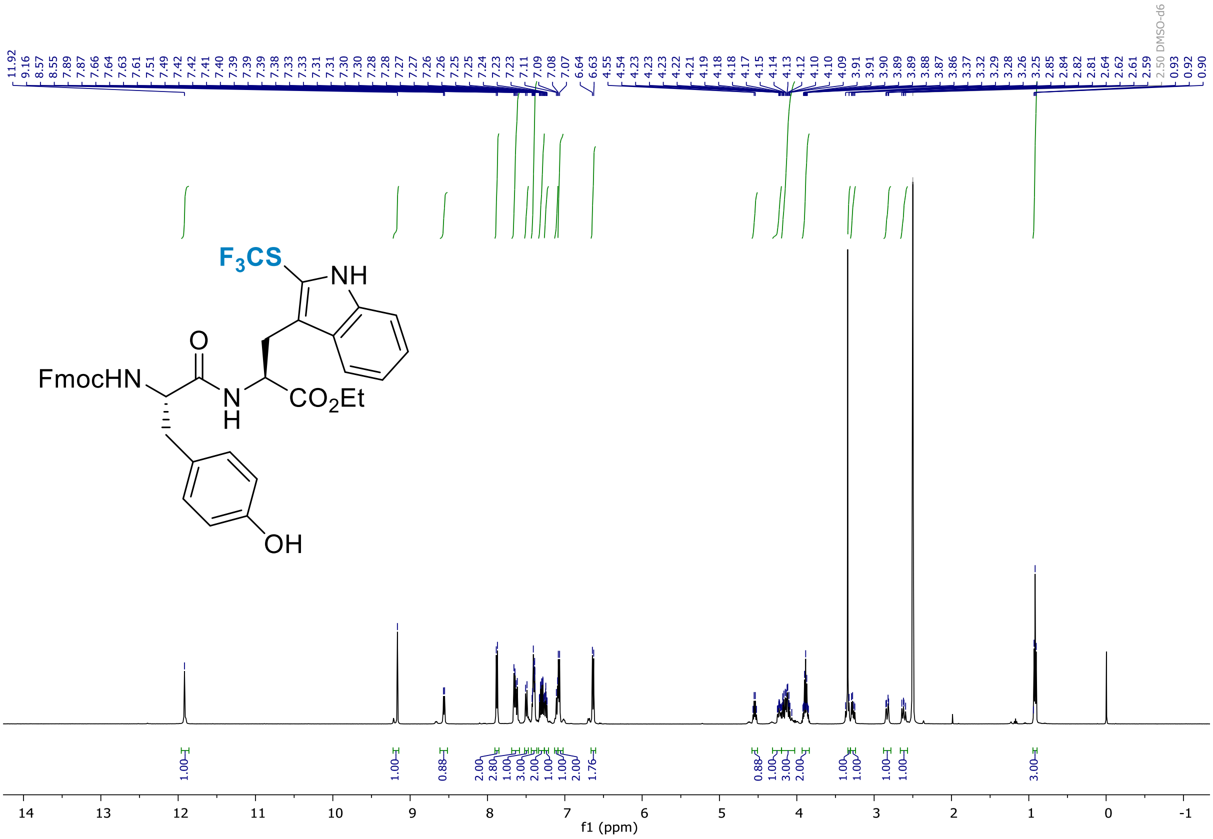

$^{13}C\{^{1}H\}$ NMR spectrum of **8g** in DMSO-d_6, 126 MHz

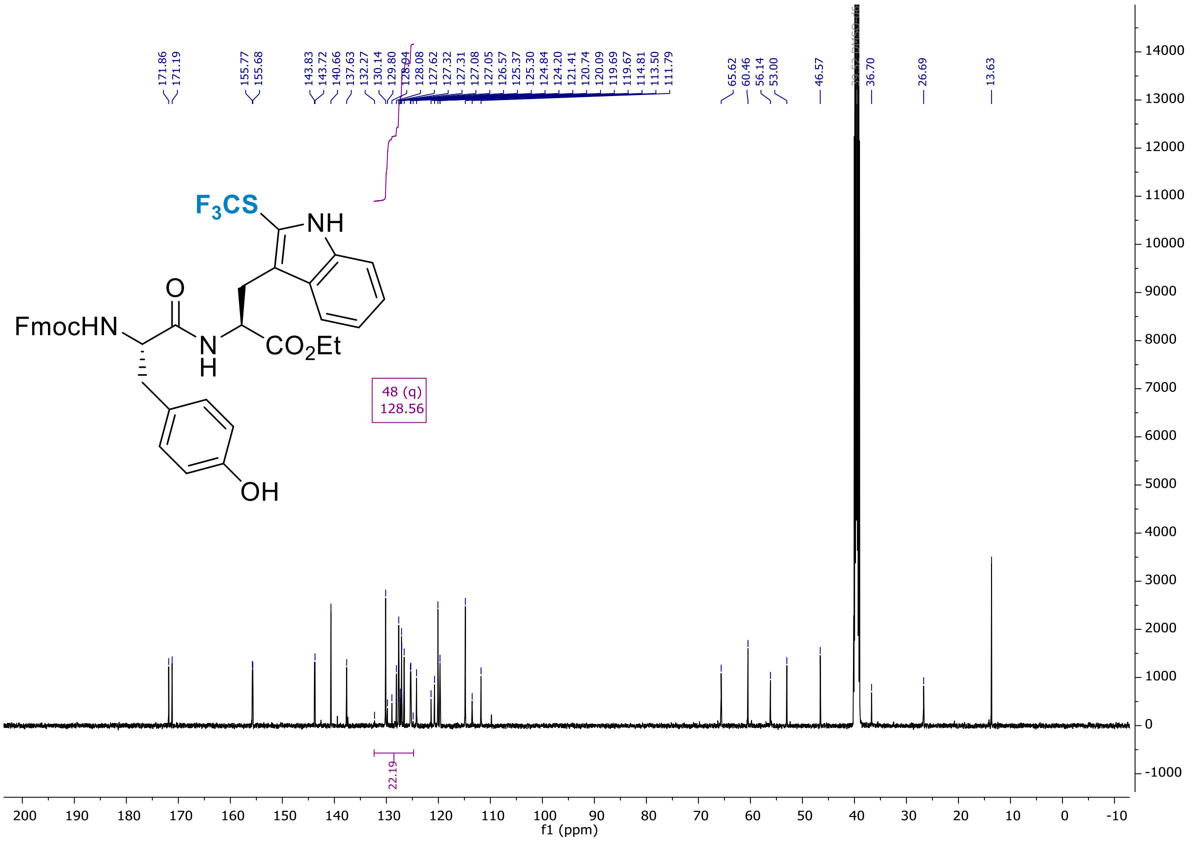

^{19}F NMR spectrum of **8g** in DMSO-d_6, 471 MHz (referenced to $CFCl_3$ at –0.24 ppm)

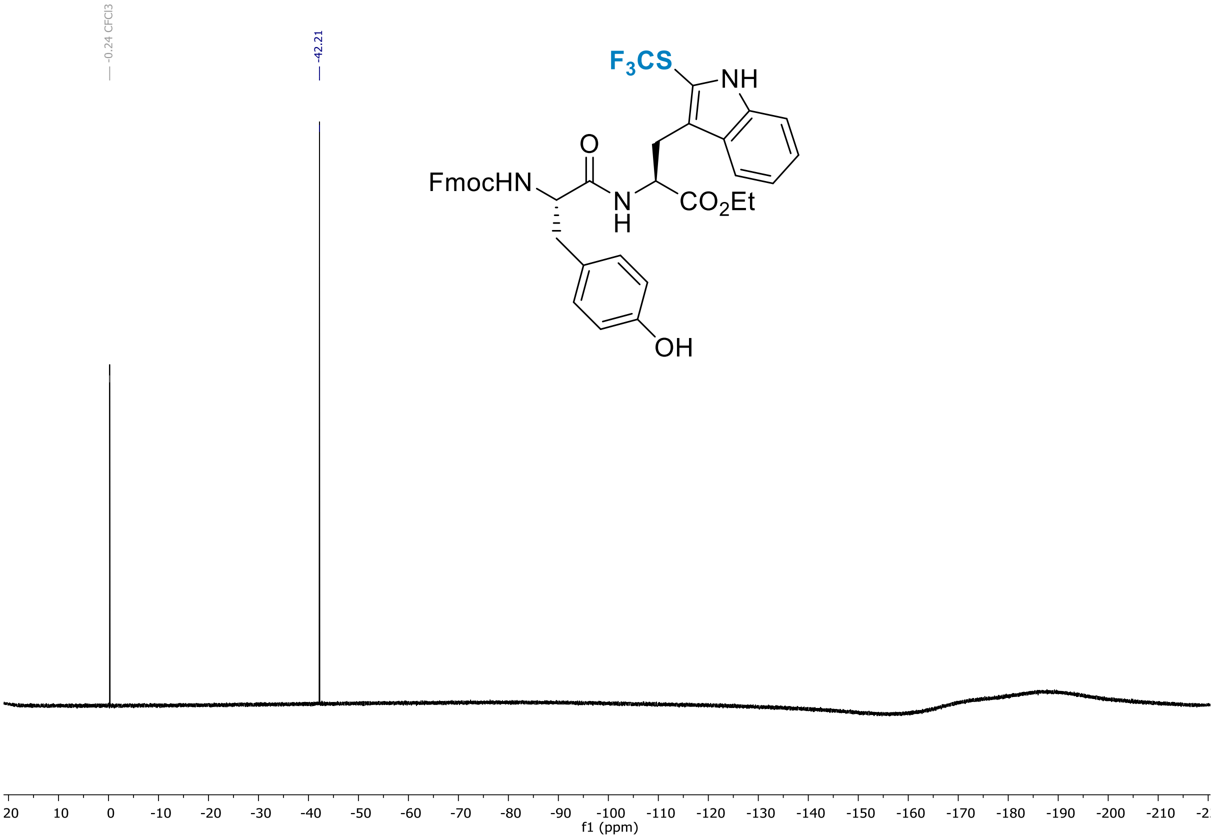

^{1}H NMR spectrum of **EM-1·TFA** in MeOD-d_3, 400 MHz (20 mM)

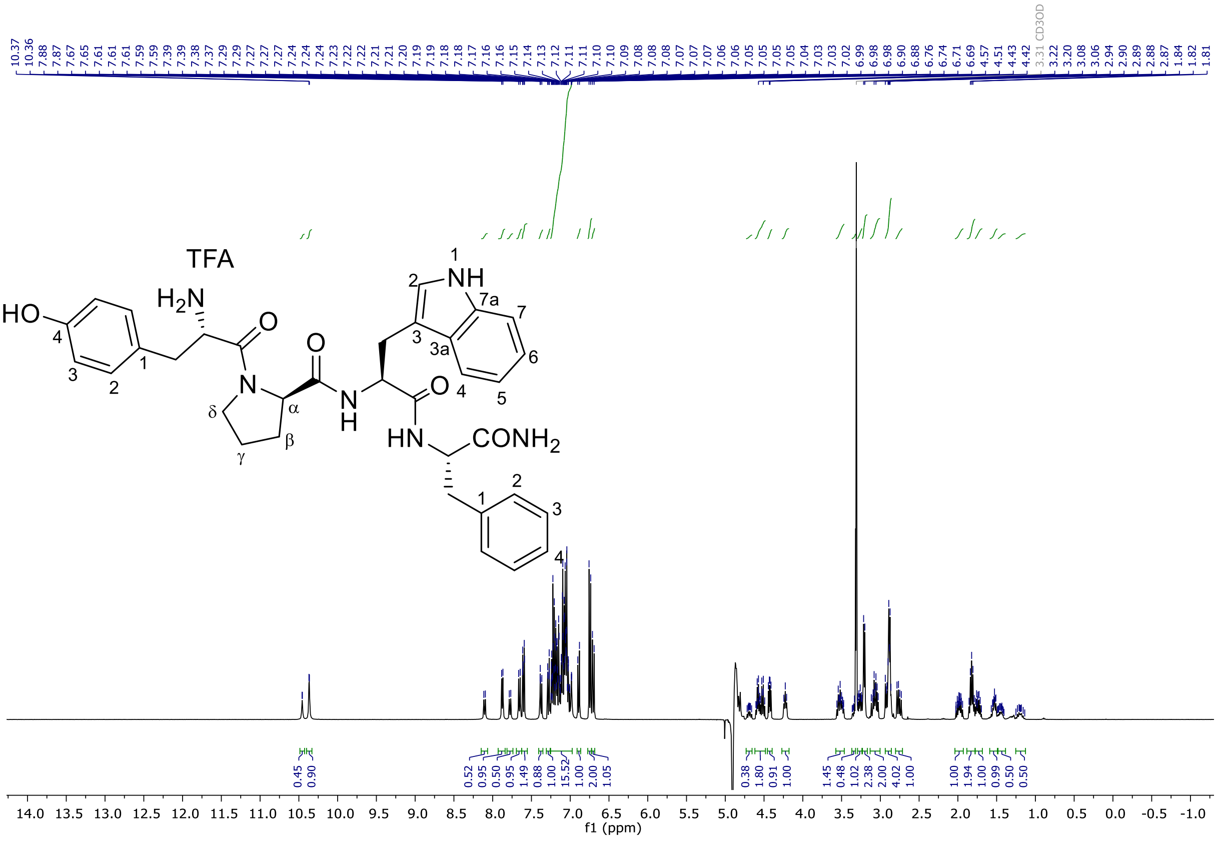

$^{13}C\{^{1}H\}$ NMR spectrum of **EM-1·TFA** in MeOD-d_3, 101 MHz (20 mM)

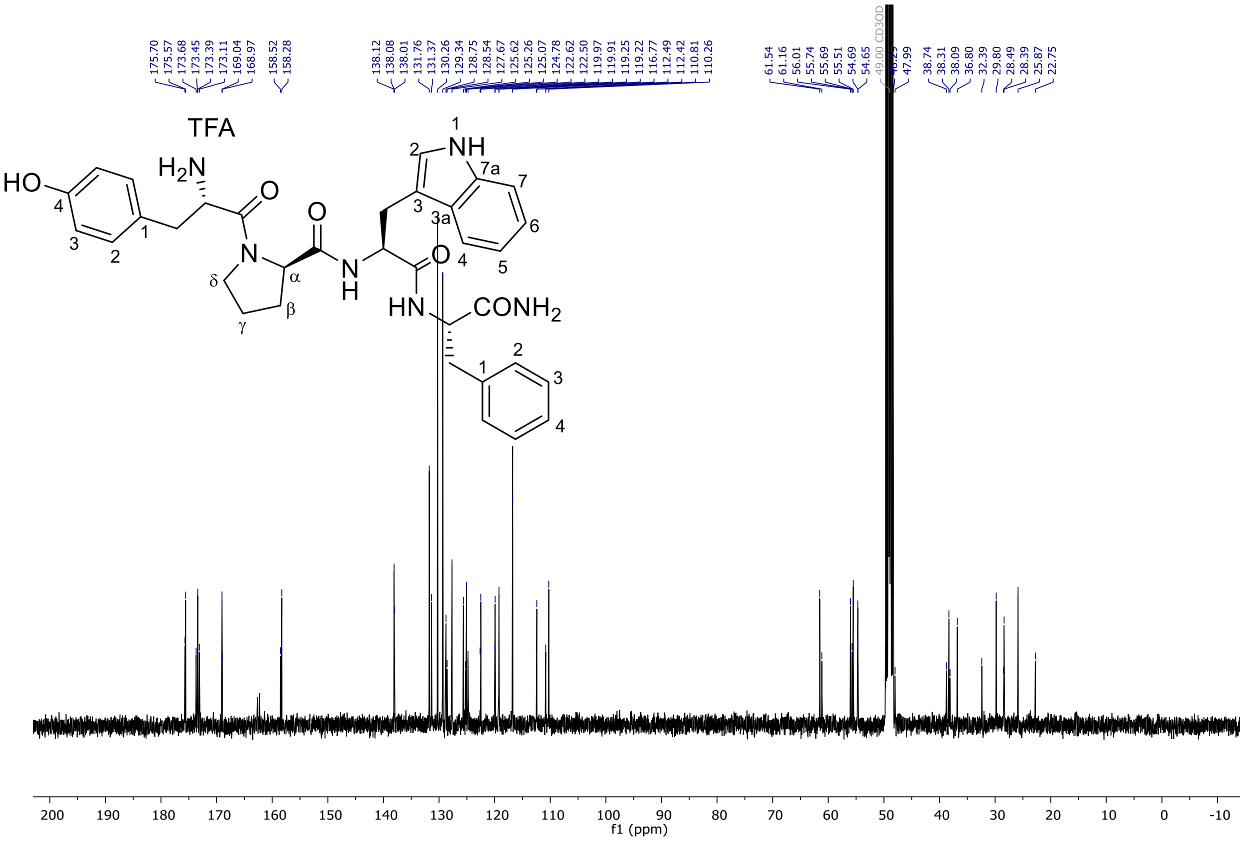

^{1}H NMR spectrum of **(CF$_3$S)Trp-EM-1·TFA** in MeOD-*d*$_3$, 400 MHz (20 mM)

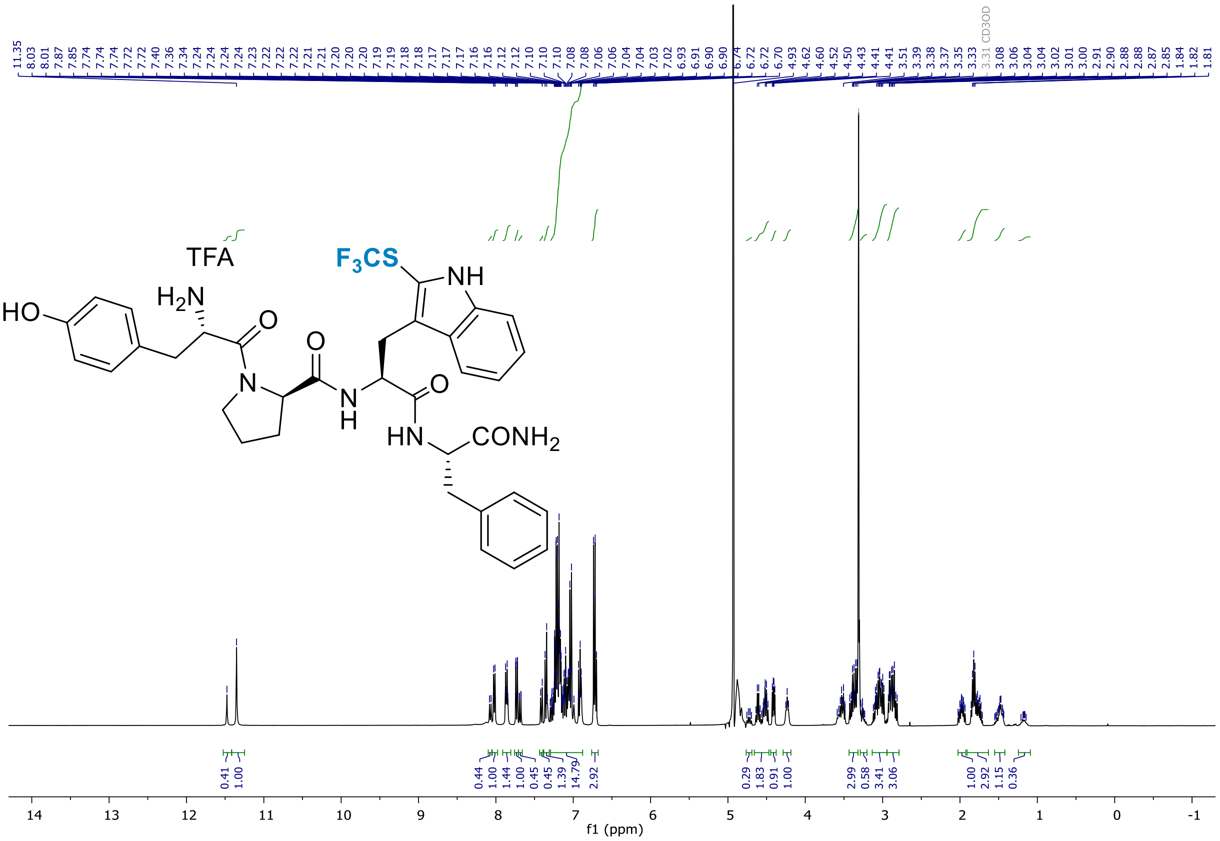

^{13}C{^{1}H} NMR spectrum of **(CF$_3$S)Trp-EM-1·TFA** in MeOD-*d*$_3$, 101 MHz (20 mM)

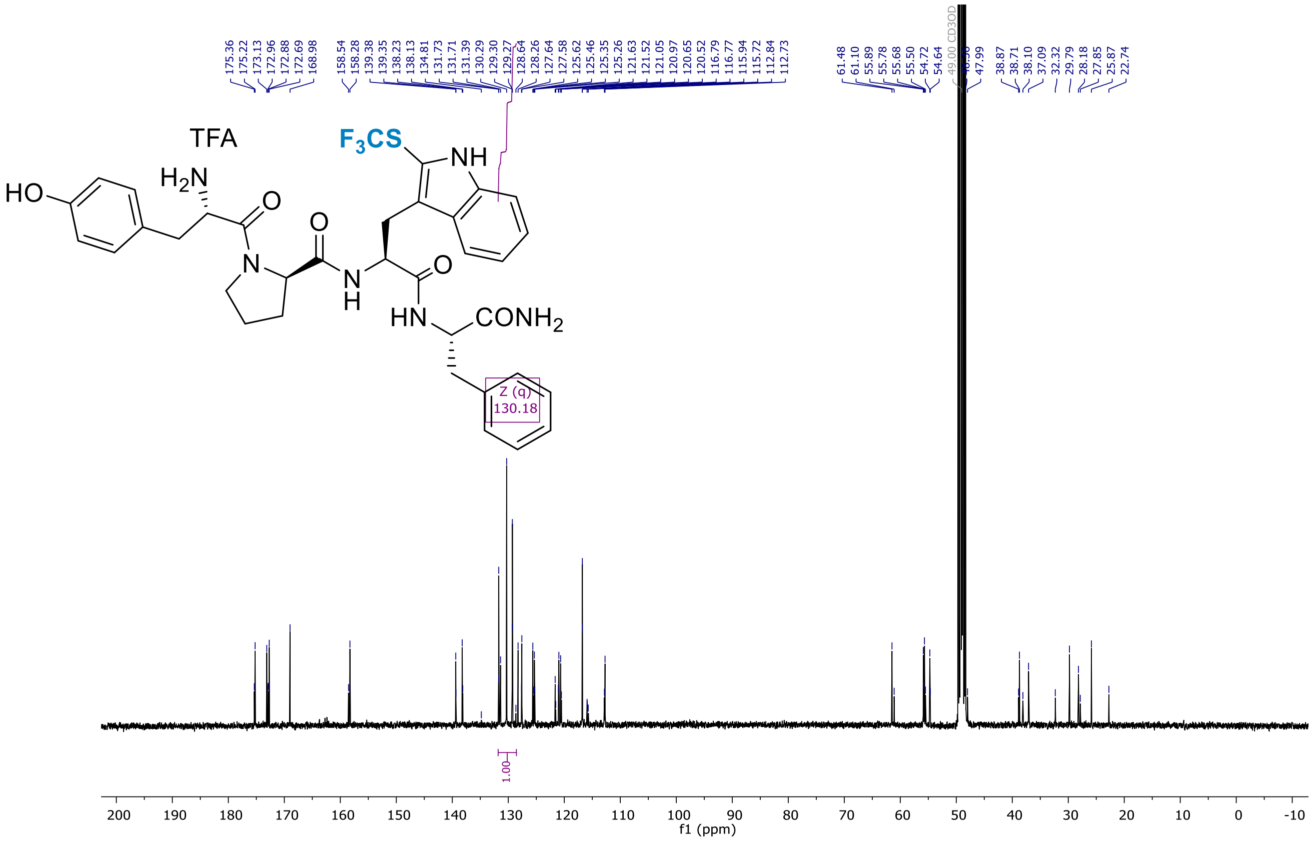

^{19}F NMR spectrum of **(CF_3S)Trp-EM-1·TFA** in MeOD-d_3, 376 MHz (20 mM, C_6F_6 as ref.)

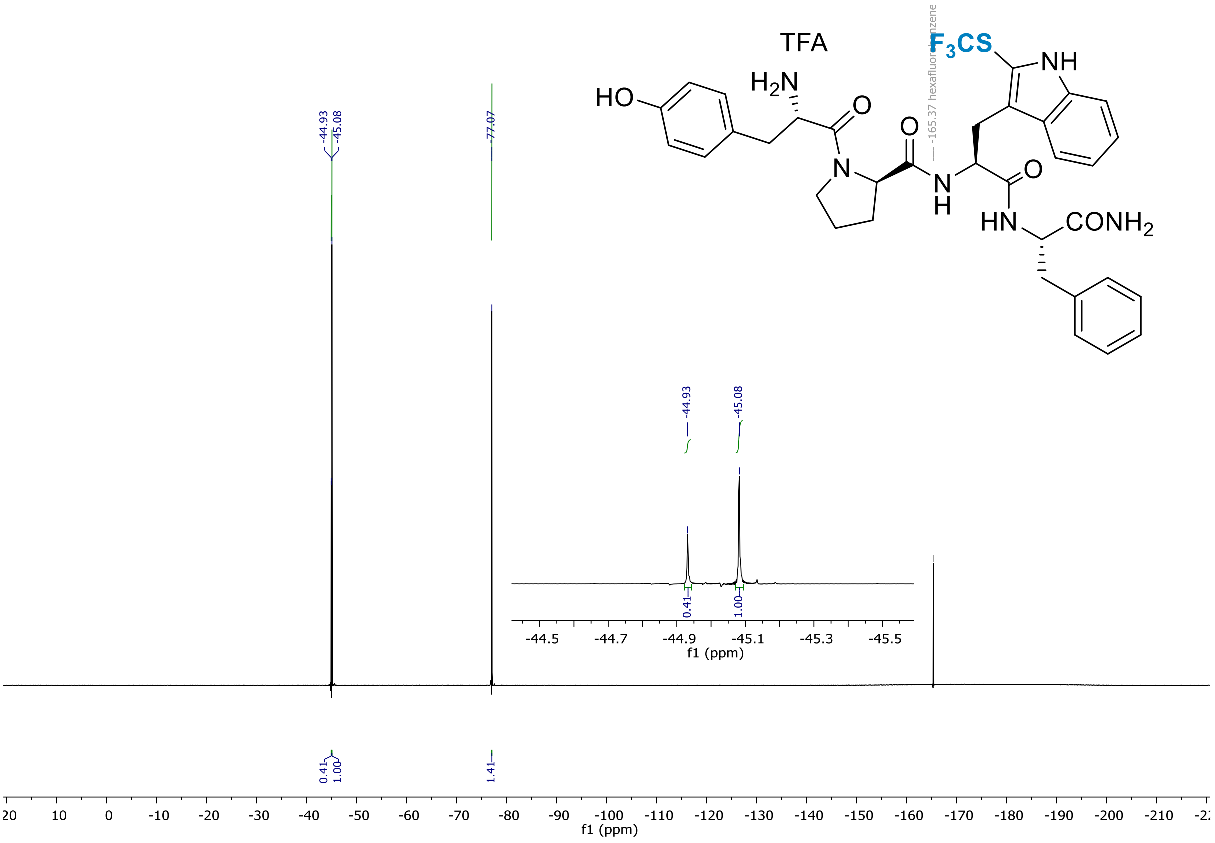

^{1}H NMR spectrum of **(CF_3S)Tyr-EM-1·TFA** in MeOD-d_3, 400 MHz (20 mM)

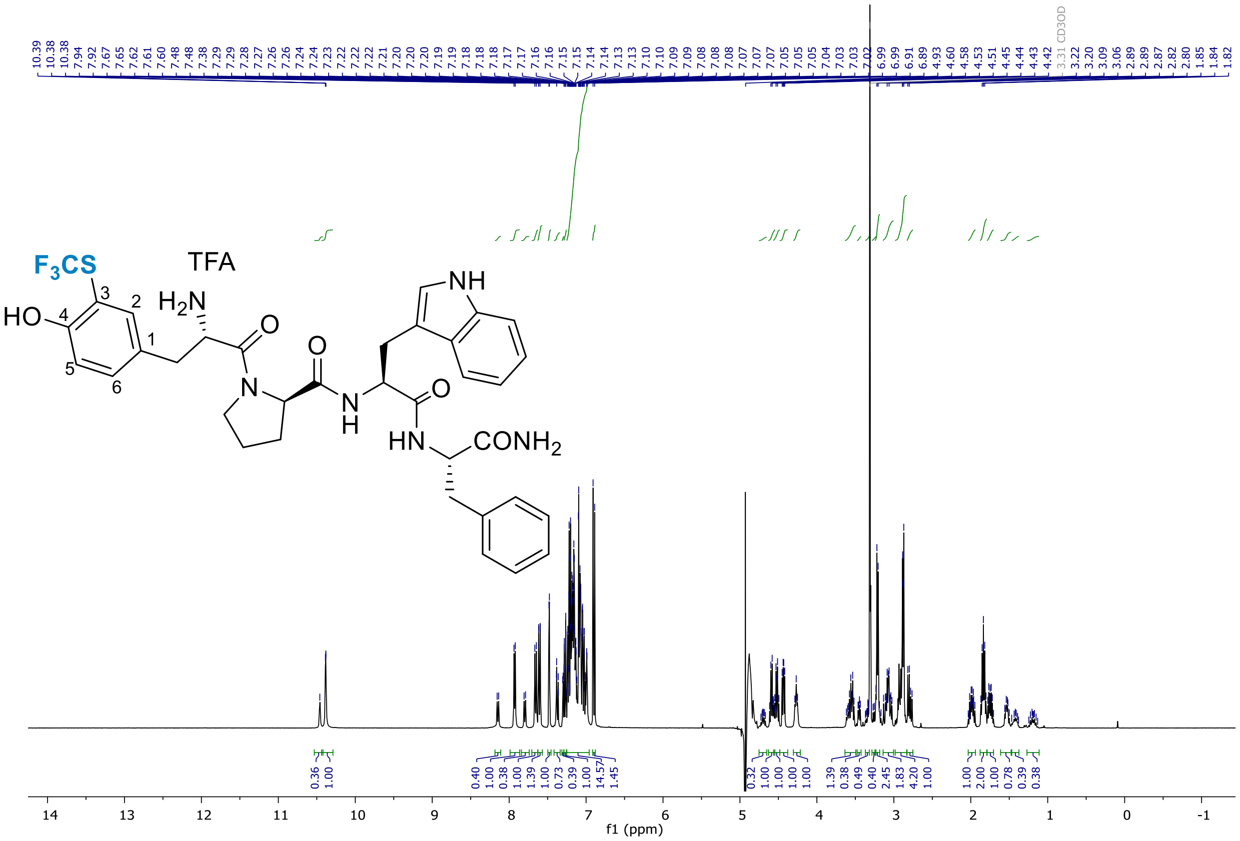

$^{13}C\{^1H\}$ NMR spectrum of **(CF_3S)Tyr-EM-1·TFA** in MeOD-d_3, 101 MHz (20 mM)

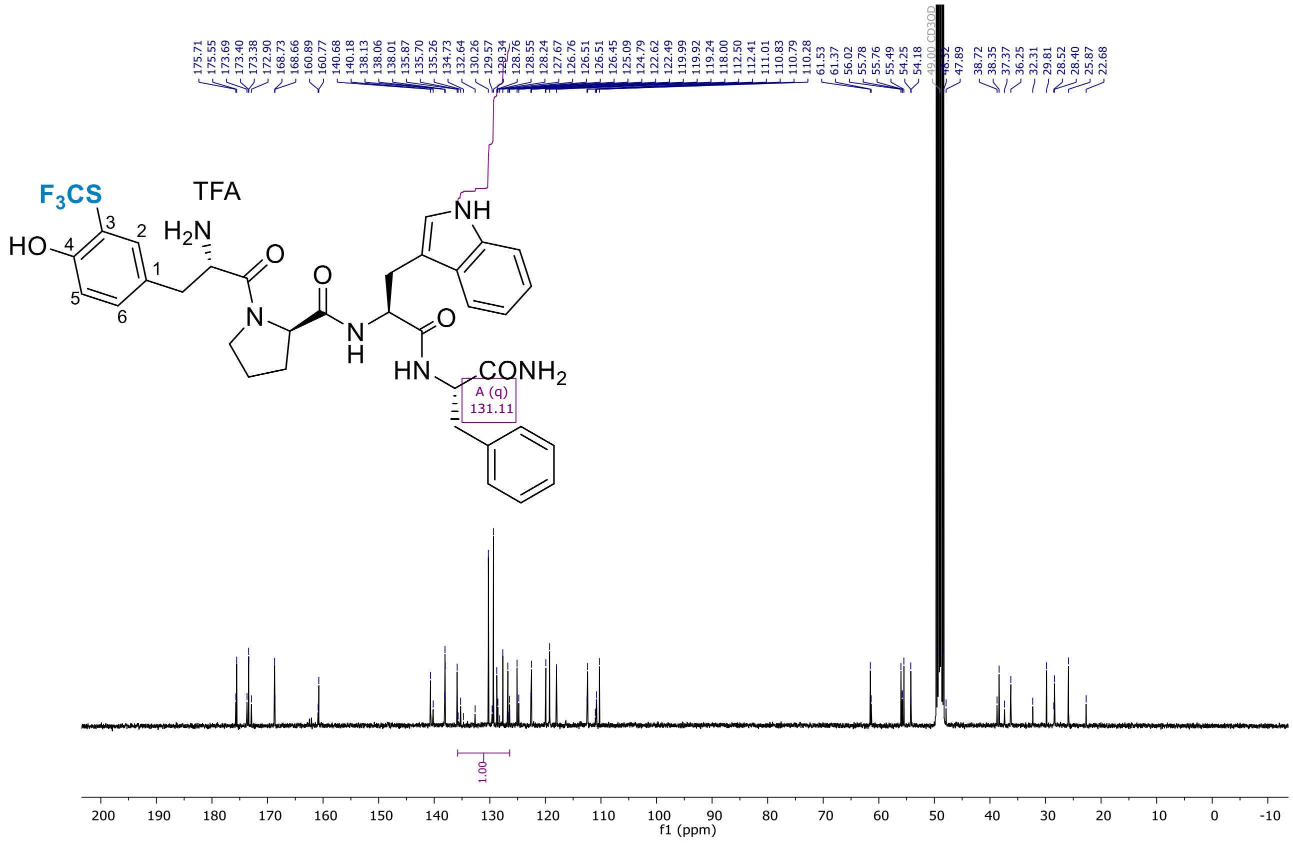

^{19}F NMR spectrum of **(CF_3S)Tyr-EM-1·TFA** in MeOD-d_3, 376 MHz (20 mM, C_6F_6 as ref.)

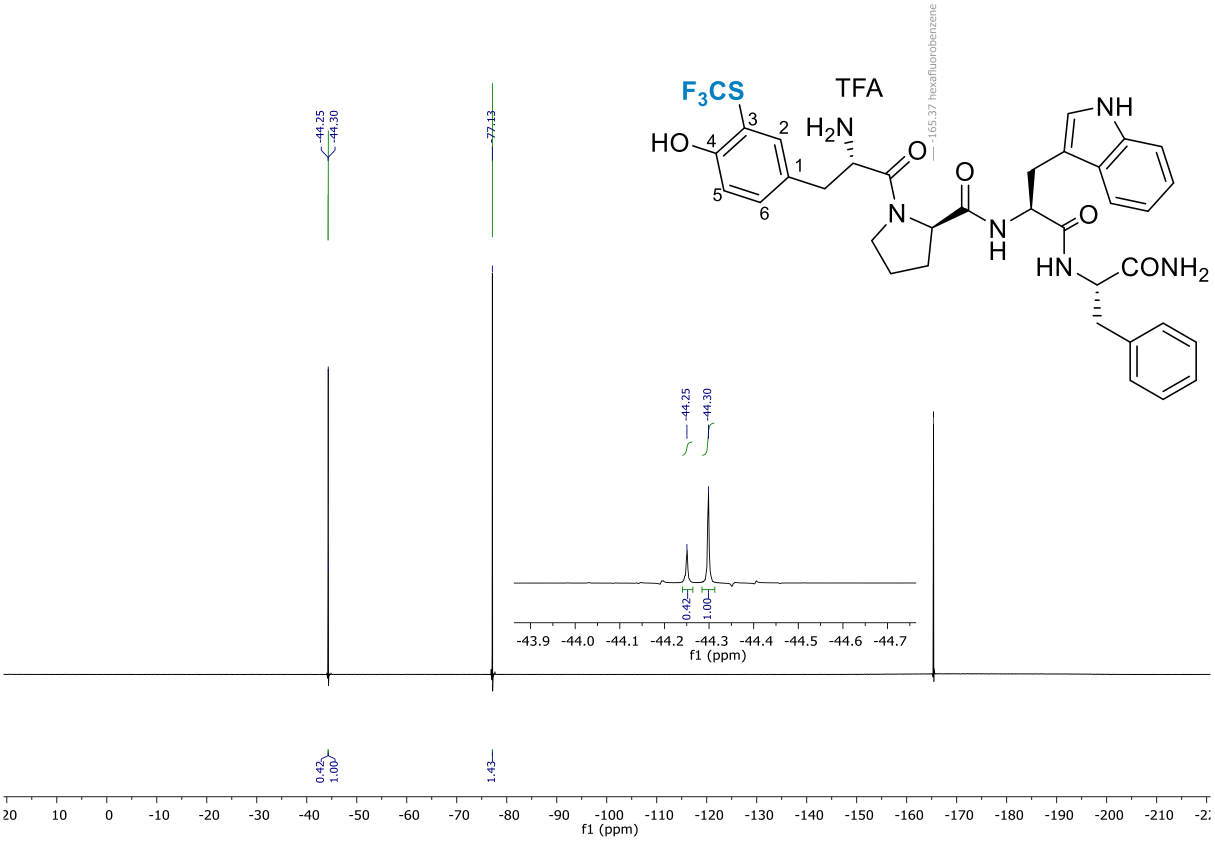

^{1}H NMR spectrum of **9** in MeOD-d_3, 400 MHz (22 mM)

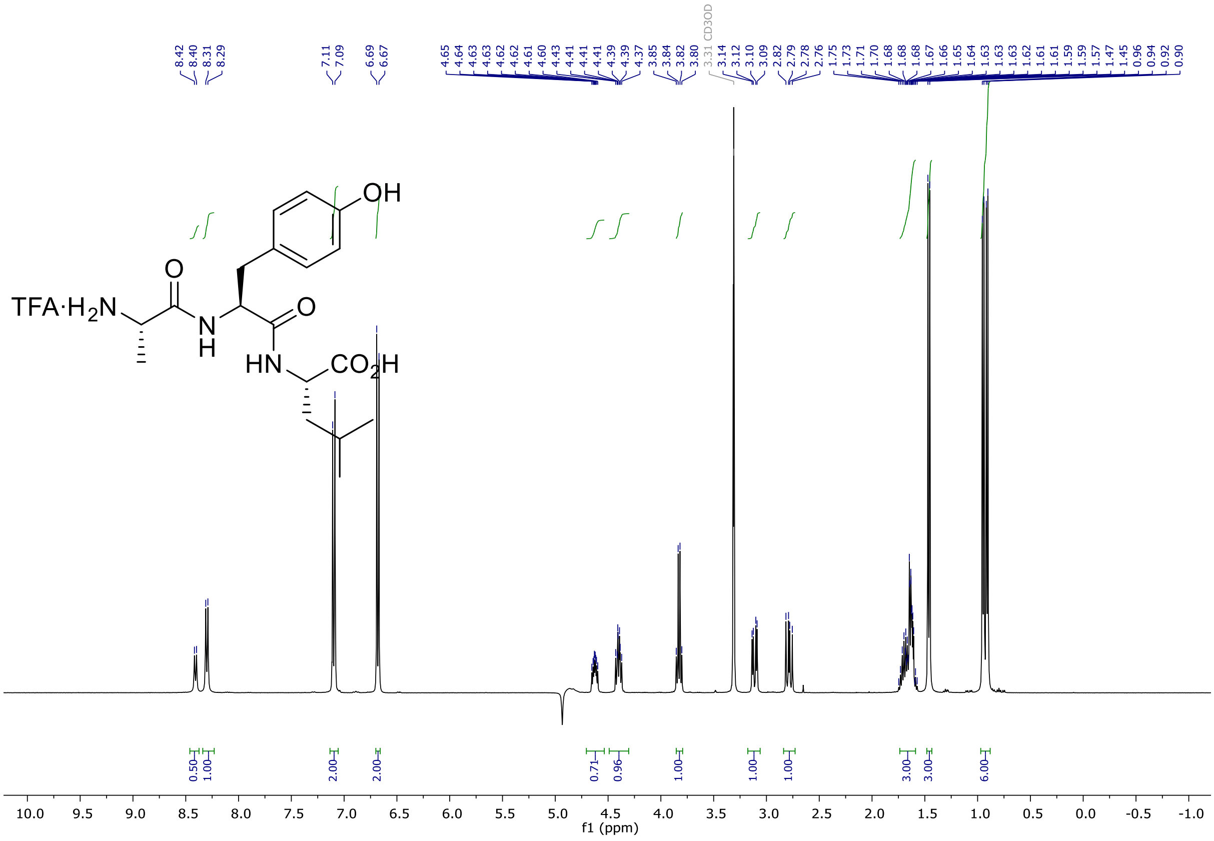

^{13}C{^{1}H} NMR spectrum of **9** in MeOD-d_3, 101 MHz (22 mM)

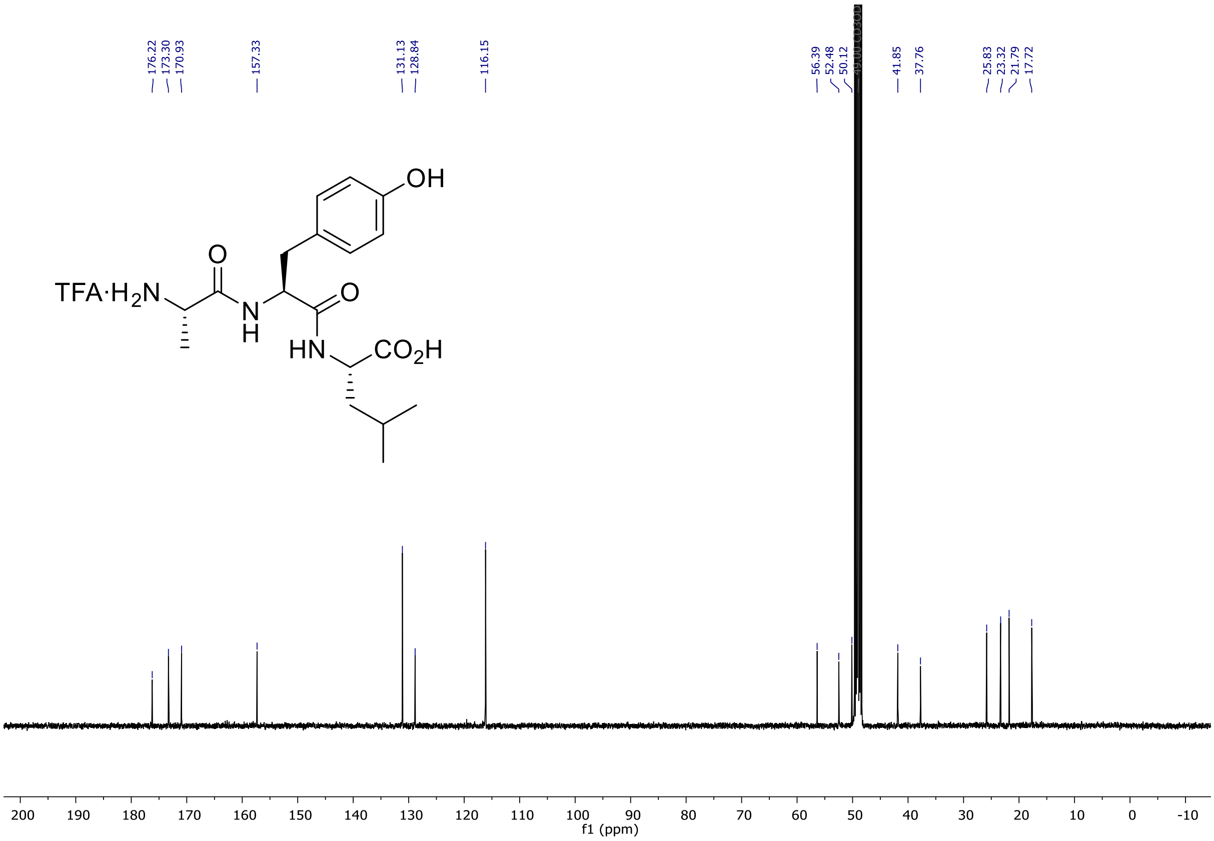

^{1}H NMR spectrum of **10** in MeOD-d_3, 400 MHz (18 mM)

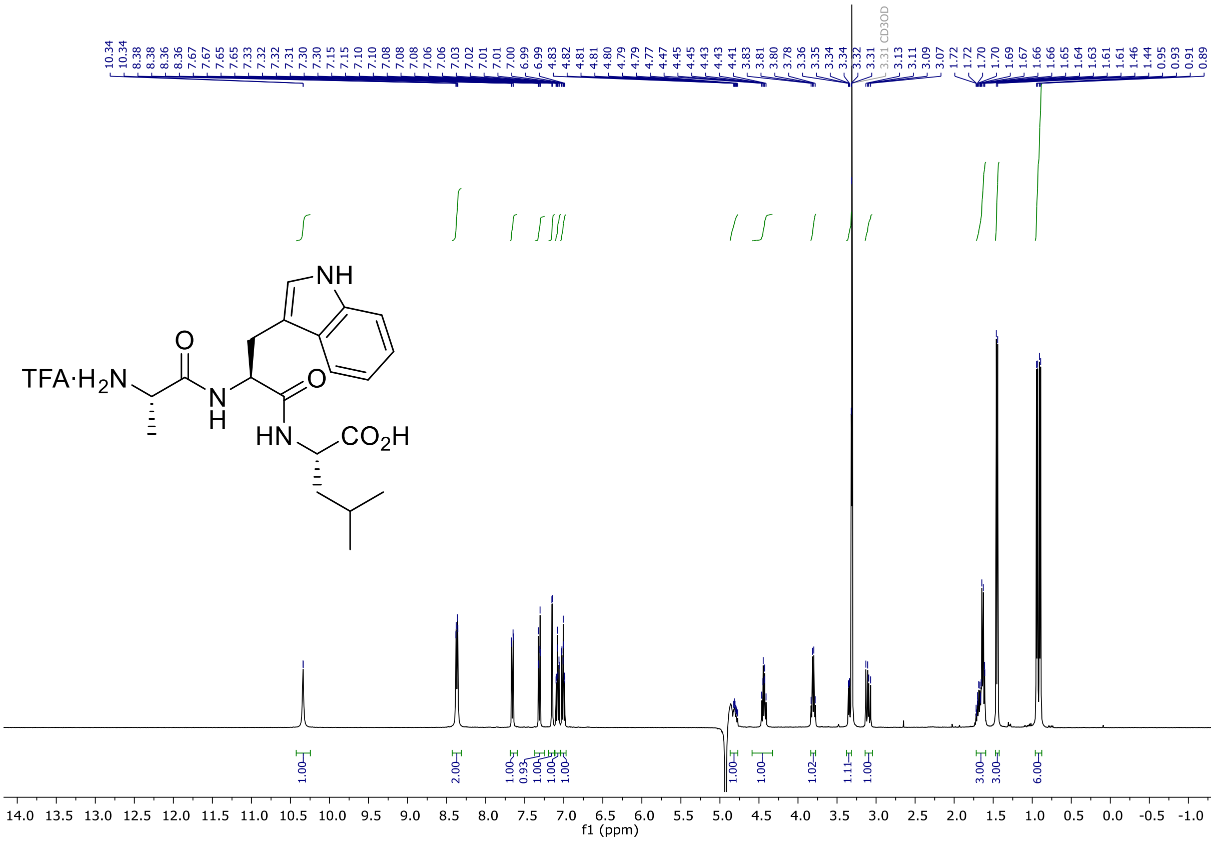

^{13}C{^{1}H} NMR spectrum of **10** in MeOD-d_3, 101 MHz (18 mM)

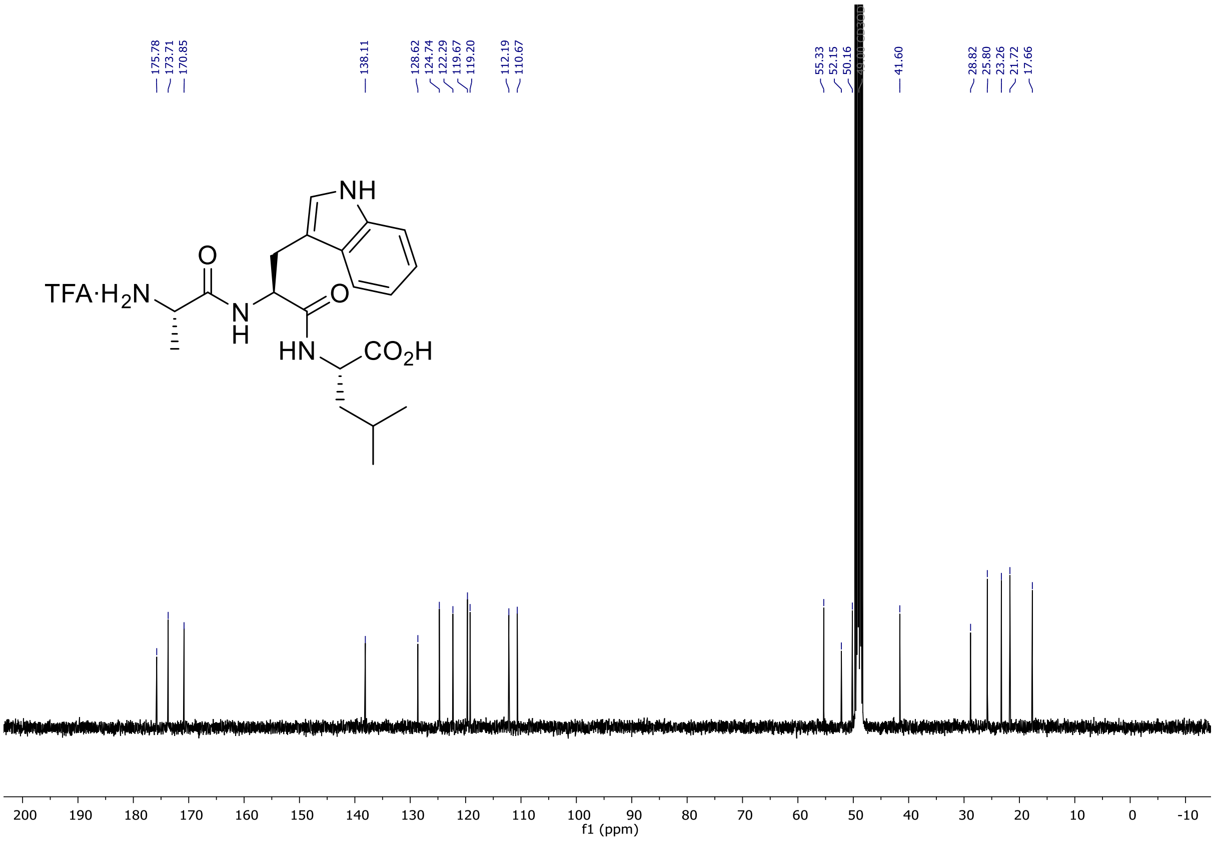

^{1}H NMR spectrum of **11** in MeOD-d_3, 400 MHz (20 mM)

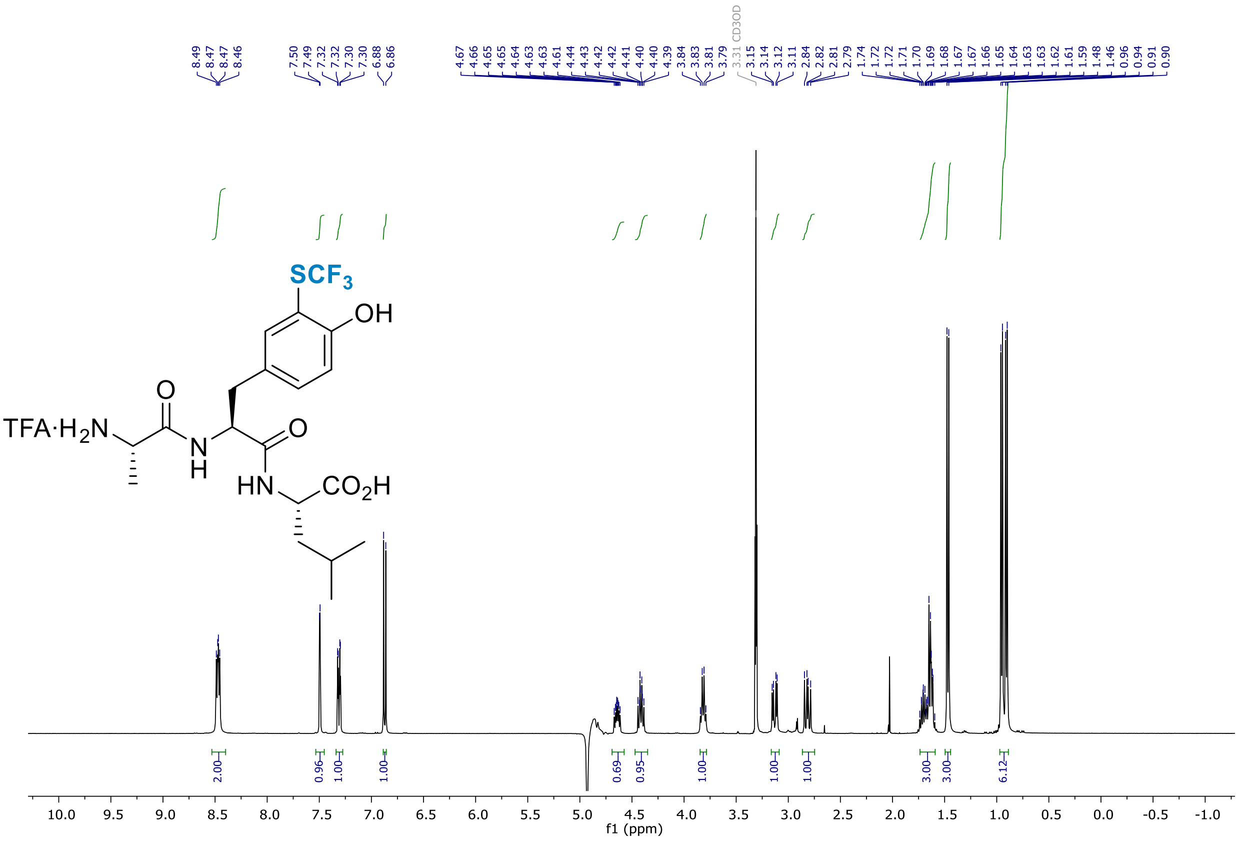

^{13}C{^{1}H} NMR spectrum of **11** in MeOD-d_3, 101 MHz (20 mM)

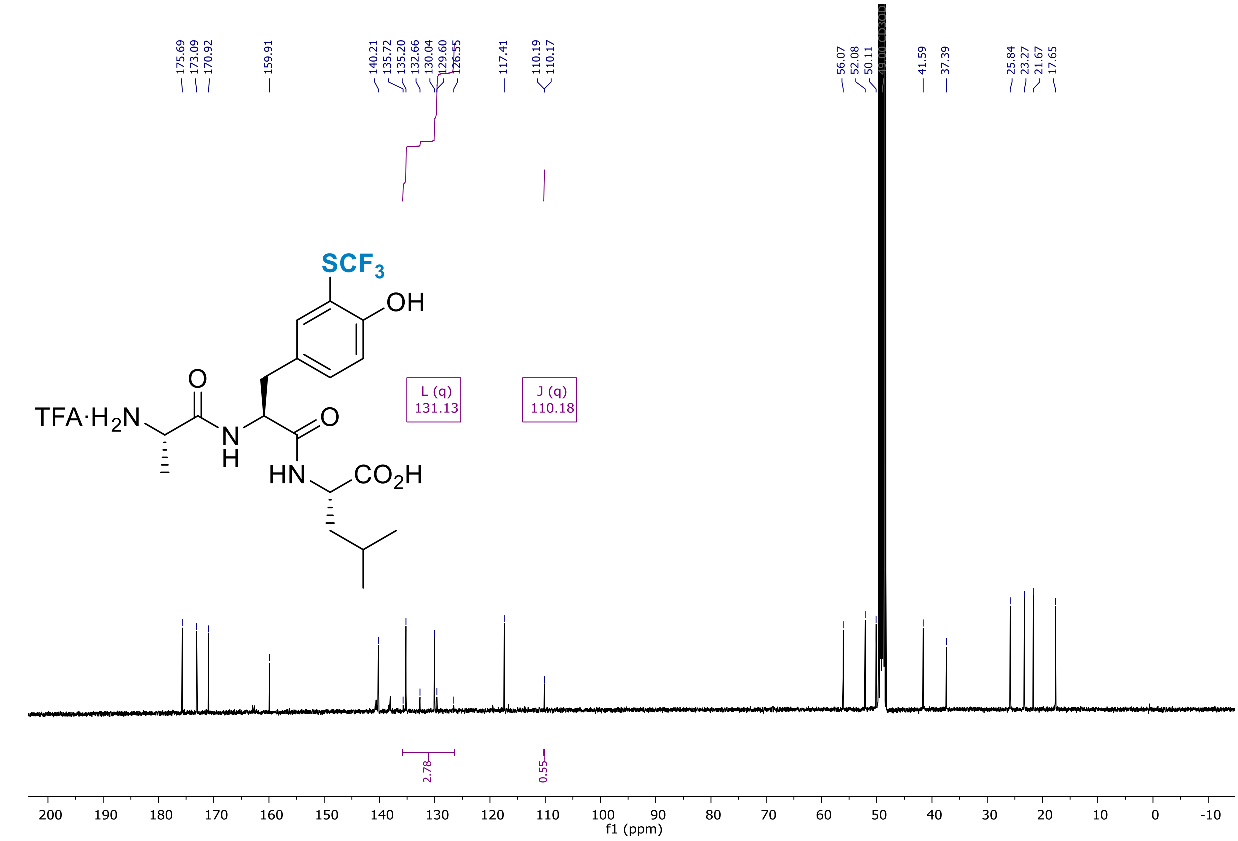

^{19}F NMR spectrum of **11** in MeOD-d_3, 376 MHz (20 mM, referenced to C_6F_6 at –165.37 ppm)

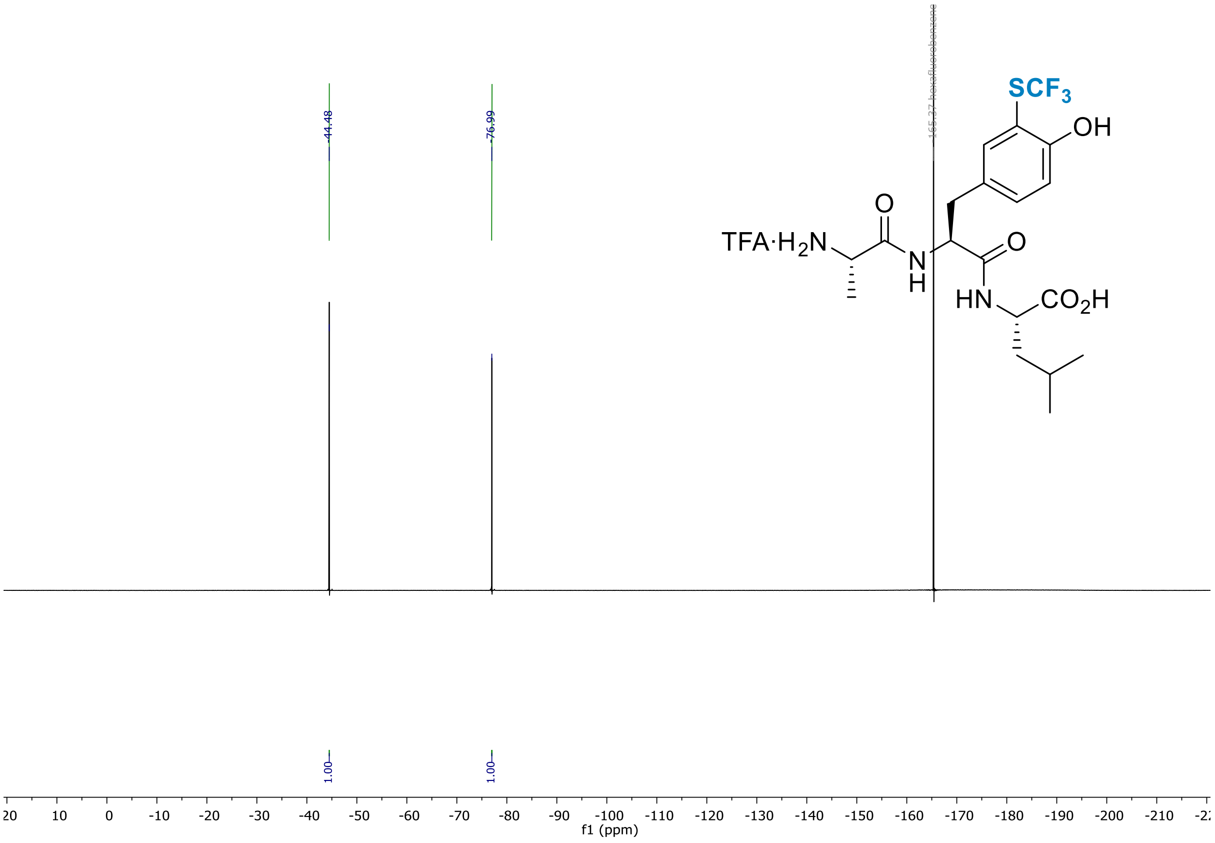

^{1}H NMR spectrum of **12** in MeOD-d_3, 400 MHz (12 mM)

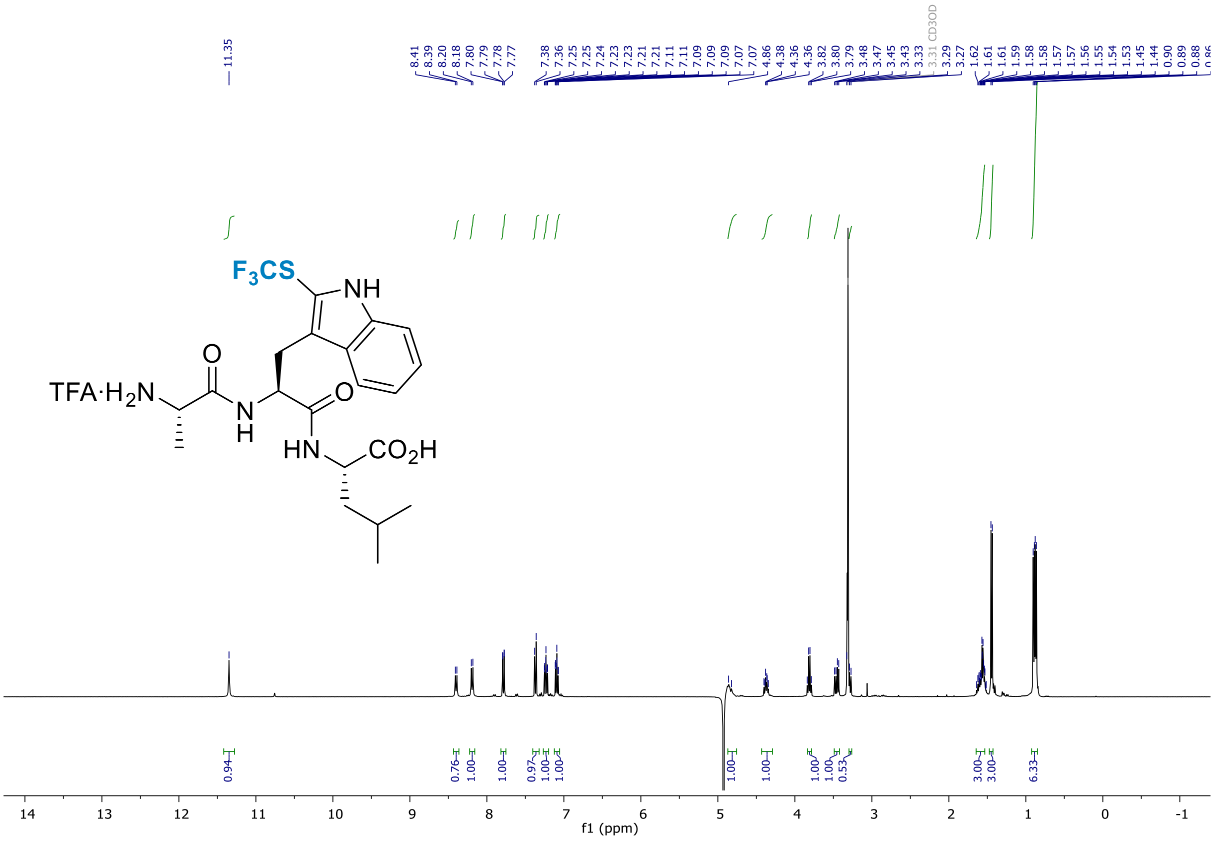

$^{13}C\{^1H\}$ NMR spectrum of **12** in MeOD-d_3, 101 MHz (12 mM)

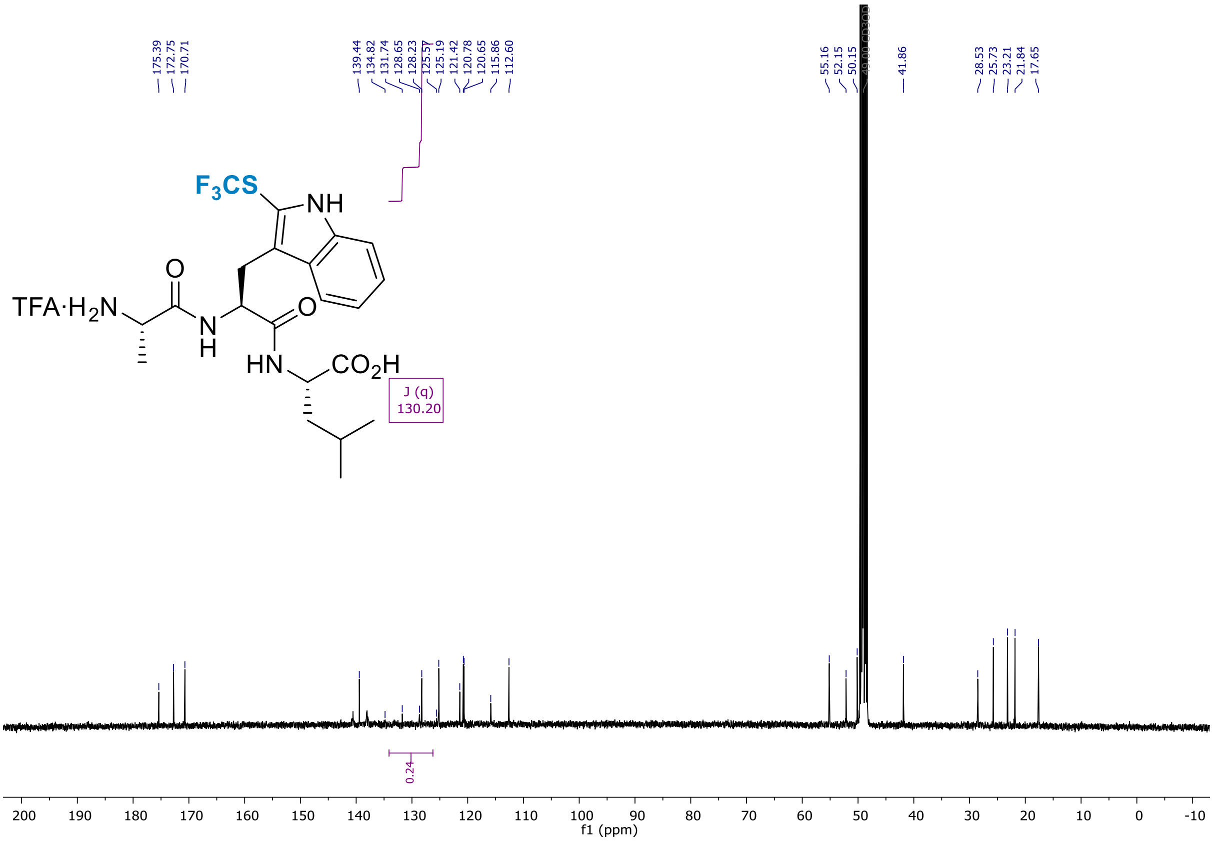

^{19}F NMR spectrum of **12** in MeOD-d_3, 376 MHz (12 mM, referenced to C_6F_6 at –165.37 ppm)

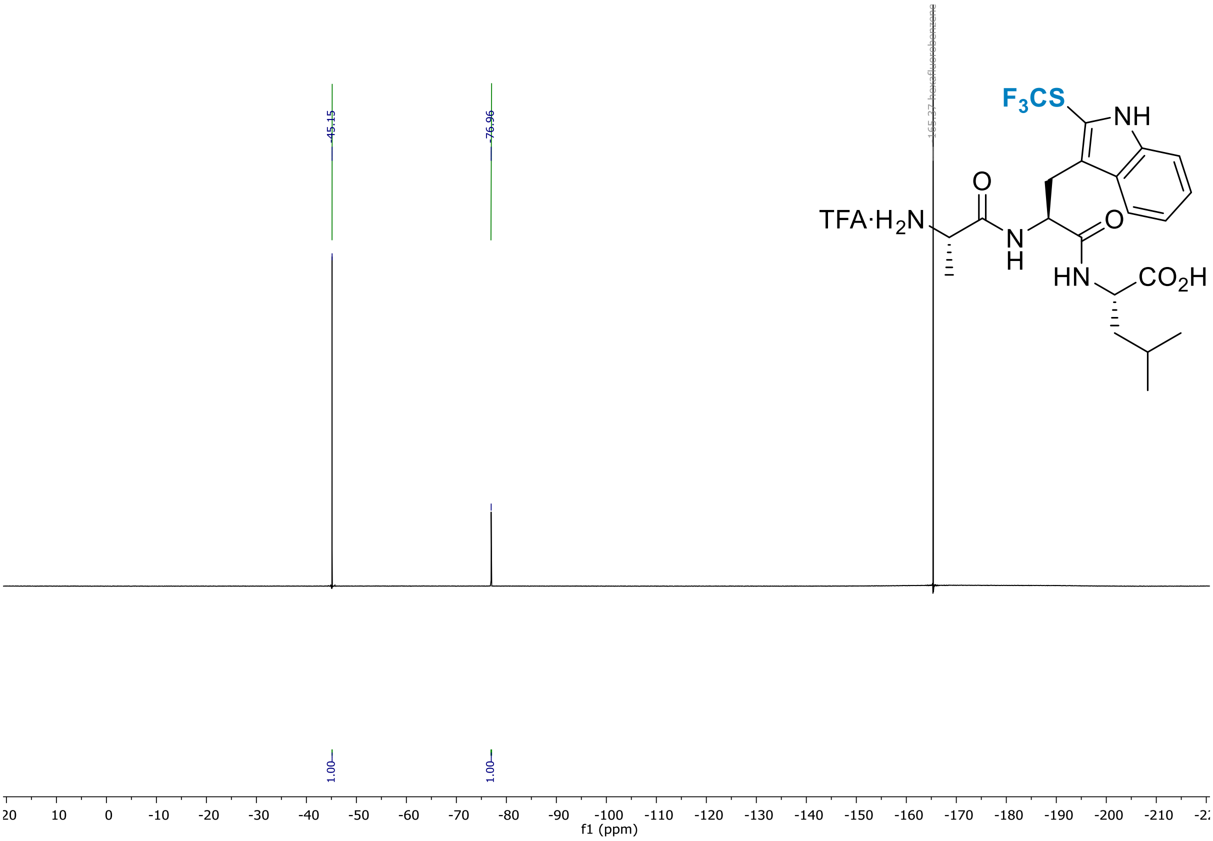

9. References

1. Rosenau, C. P.; Jelier, B. J.; Gossert, A. D.; Togni, A. Exposing the Origins of Irreproducibility in Fluorine NMR Spectroscopy. *Angew. Chem. Int. Ed.* **2018,** *57*, 9528–9533.

2. Ferry, A.; Billard, T.; Langlois, B. R.; Bacqué, E. Synthesis of Trifluoromethanesulfinamidines and -sulfanylamides. *J. Org. Chem.* **2008,** *73*, 9362–9365.

3. Torres-García, C.; Díaz, M.; Blasi, D.; Farràs, I.; Fernández, I.; Ariza, X.; Farràs, J.; Lloyd-Williams, P.; Royo, M.; Nicolás, E. Side Chain Anchoring of Tryptophan to Solid Supports Using a Dihydropyranyl Handle: Synthesis of Brevianamide F. *Int. J. Pept. Res. Ther.* **2012,** *18*, 7–19.

4. Wei, Q.; Wang, Y.-Y.; Du, Y.-L.; Gong, L.-Z. Organocatalytic asymmetric selenofunctionalization of tryptamine for the synthesis of hexahydropyrrolo[2,3-b]indole derivatives. *Beilstein J. Org. Chem.* **2013,** *9*, 1559–1564.

5. Yang, Y.; Jiang, X.; Qing, F.-L. Sequential Electrophilic Trifluoromethanesulfanylation–Cyclization of Tryptamine Derivatives: Synthesis of C(3)-Trifluoromethanesulfanylated Hexahydropyrrolo[2,3-*b*]indoles. *J. Org. Chem.* **2012,** *77*, 7538–7547.

6. Wang, S.; Dupin, L.; Noël, M.; Carroux, C. J.; Renaud, L.; Géhin, T.; Meyer, A.; Souteyrand, E.; Vasseur, J.-J.; Vergoten, G.; Chevolot, Y.; Morvan, F.; Vidal, S. Toward the Rational Design of Galactosylated Glycoclusters That Target Pseudomonas aeruginosa Lectin A (LecA): Influence of Linker Arms That Lead to Low-Nanomolar Multivalent Ligands. *Chem. Eur. J.* **2016,** *22*, 11785–11794.

7. Rosés, C.; Camó, C.; Oliveras, À.; Moll, L.; López, N.; Feliu, L.; Planas, M. Total Solid-Phase Synthesis of Dehydroxy Fengycin Derivatives. *J. Org. Chem.* **2018,** *83*, 15297–15311.

8. Zajdel, P.; Nomezine, G.; Masurier, N.; Amblard, M.; Pawłowski, M.; Martinez, J.; Subra, G. A New Highly Versatile Handle for Chemistry on a Solid Support: The Pipecolic Linker. *Chem. Eur. J.* **2010,** *16*, 7547–7553.

9. Spicer, C. D.; Pujari-Palmer, M.; Autefage, H.; Insley, G.; Procter, P.; Engqvist, H.; Stevens, M. M. Synthesis of Phospho-Amino Acid Analogues as Tissue Adhesive Cement Additives. *ACS Cent. Sci.* **2020,** *6*, 226–231.

10. Di Gioia, M. L.; Gagliardi, A.; Leggio, A.; Leotta, V.; Romio, E.; Liguori, A. *N*-Urethane protection of amines and amino acids in an ionic liquid. *RSC Adv.* **2015,** *5*, 63407–63420.

11. Elumalai, N.; Berg, A.; Natarajan, K.; Scharow, A.; Berg, T. Nanomolar Inhibitors of the Transcription Factor STAT5b with High Selectivity over STAT5a. *Angew. Chem. Int. Ed.* **2015,** *54*, 4758–4763.

12. Song, Y.-L.; Peach, M. L.; Roller, P. P.; Qiu, S.; Wang, S.; Long, Y.-Q. Discovery of a Novel Nonphosphorylated Pentapeptide Motif Displaying High Affinity for Grb2-SH2 Domain by the Utilization of 3'-Substituted Tyrosine Derivatives. *J. Med. Chem* **2006,** *49*, 1585–1596.

13. St-Georges, C.; Désilets, A.; Béliveau, F.; Ghinet, M.; Dion, S. P.; Colombo, É.; Boudreault, P.-L.; Najmanovich, R. J.; Leduc, R.; Marsault, É. Modulating the selectivity of matriptase-2 inhibitors with unnatural amino acids. *Eur. J. Med. Chem.* **2017,** *129*, 110–123.

14. (a) Cozett, R. E.; Venter, G. A.; Gokada, M. R.; Hunter, R. Catalytic enantioselective acyl transfer: the case for 4-PPY with a C-3 carboxamide peptide auxiliary based on synthesis and modelling studies. *Org. Biomol. Chem.* **2016,** *14*, 10914–10925. (b) Xue, F.; Seto, C. T. Selective Inhibitors of the Serine Protease Plasmin: Probing the S3 and S3' Subsites Using a Combinatorial Library. *J. Med. Chem* **2005,** *48*, 6908–6917.

15. Crich, D.; Banerjee, A. Chemistry of the Hexahydropyrrolo[2,3-*b*]indoles: Configuration, Conformation, Reactivity, and Applications in Synthesis. *Acc. Chem. Res.* **2007,** *40*, 151–161.

16. Pryyma, A.; Bu, Y. J.; Wai, Y.; Patrick, B. O.; Perrin, D. M. Synthesis and Activation of Bench-Stable 3a-Fluoropyrroloindolines as Latent Electrophiles for the Synthesis of C-2-Thiol-Substituted Tryptophans and C-3a-Substituted Pyrroloindolines. *Org. Lett.* **2019,** *21*, 8234–8238.

17. Gadais, C.; Devillers, E.; Gasparik, V.; Chelain, E.; Pytkowicz, J.; Brigaud, T. Probing the Outstanding Local Hydrophobicity Increases in Peptide Sequences Induced by Incorporation of Trifluoromethylated Amino Acids. *ChemBioChem* **2018,** *19*, 1026–1030.

18. Bezençon, J.; Wittwer, M. B.; Cutting, B.; Smieško, M.; Wagner, B.; Kansy, M.; Ernst, B. pK_a determination by ^1H NMR spectroscopy – An old methodology revisited. *J. Pharm. Biomed. Anal.* **2014,** *93*, 147–155.

19. Krężel, A.; Bal, W. A formula for correlating pK_a values determined in D_2O and H_2O. *J. Inorg. Biochem.* **2004,** *98*, 161–166.

20. Tejwani, R. W.; Stouch, T. R.; Anderson, B. D. Substituent effects on the ionization and partitioning of *p*-(aminoethyl)phenols and structurally related compounds: Electrostatic effects dependent on conformation. *J. Pharm. Sci.* **2009,** *98*, 4534–4544.
